# Diversity Scaling Analysis of Chinese Gut Microbiomes Across Ethnicities and Lifestyles

**DOI:** 10.3389/fmicb.2021.736393

**Published:** 2021-12-08

**Authors:** Wanmeng Xiao, Depei Gao, Hongju (Daisy) Chen, Yuting Qiao, Zhanshan (Sam) Ma, Lincan Duan

**Affiliations:** ^1^Computational Biology and Medical Ecology Lab, State Key Laboratory of Genetic Resources and Evolution, Kunming Institute of Zoology, Chinese Academy of Sciences, Kunming, China; ^2^Kunming College of Life Sciences, University of Chinese Academy of Sciences, Kunming, China; ^3^Radiology Department, The 3rd-Affiliated Hospital of Kunming Medical University, Kunming, China; ^4^Center for Excellence in Animal Evolution and Genetics, Chinese Academy of Sciences, Kunming, China; ^5^The 2nd Thoracic Surgery Department, The 3rd-Affiliated Hospital of Kunming Medical University, Kunming, China

**Keywords:** Chinese gut microbiomes, diversity scaling, diversity–area relationship (DAR), ethnicity, lifestyle

## Abstract

Diversity scaling (changes) of human gut microbiome is important because it measures the inter-individual heterogeneity of diversity and other important parameters of population-level diversity. Understanding the heterogeneity of microbial diversity can be used as a reference for the personalized medicine of microbiome-associated diseases. Similar to diversity *per se*, diversity scaling may also be influenced by host factors, especially lifestyles and ethnicities. Nevertheless, this important topic regarding Chinese populations has not been addressed, to our best knowledge. Here, we fill the gap by applying a recent extension to the classic species–area relationship (SAR), i.e., diversity–area relationship (DAR), to reanalyze a large dataset of Chinese gut microbiomes covering the seven biggest Chinese ethnic groups (covering > 95% Chinese) living rural and urban lifestyles. Four DAR profiles were constructed to investigate the diversity scaling, diversity overlap, potential maximal diversity, and the ratio of local to global diversity of Chinese gut microbiomes. We discovered the following: (*i*) The diversity scaling parameters (*z*) at various taxon levels are little affected by either ethnicity or lifestyles, as exhibited by less than 0.5% differences in pairwise comparisons. (*ii*) The maximal accrual diversity (potential diversity) exhibited difference in only about 5% of pairwise comparisons, and all of the differences occurred in ethnicity comparisons (i.e., lifestyles had no effects). (*iii*) Ethnicity seems to have stronger effects than lifestyles across all taxon levels, and this may reflect the reality that China has been experiencing rapid urbanization in the last few decades, while the ethnic-related genetic background may change relatively little during the same period.

## Introduction

The microbes that inhabit in and on the human body constitute the human microbiota. Thanks to the development of the Human Microbiome Project (HMP), we have opened up a new era in the study of human microbes (Turnbaugh et al., 2007). As a vital component of the human microbiome, the human gut microbiome has been extensively studied. Many studies exposed the truth that the gut microbiome has an important influence on the occurrence or development of inflammatory bowel disease, type 2 diabetes, obesity, epilepsy, and other diseases (Hollister et al., 2014; Ni et al., 2017; Heintz-Buschart and Wilmes, 2018; Canfora et al., 2019; Dahlin and Prast-Nielsen, 2019; Gurung et al., 2020; Rackaityte and Lynch, 2020). As far as we know, inter-individual heterogeneities in diversity scaling and abundance also existed in healthy populations (Arumugam et al., 2011; Human Microbiome Project Consortium, 2012; Zhang et al., 2015). Since we were born, we have been constantly building our own microbial network. Factors such as diet, lifestyle, genetics, geography, and ethnic origin all play significant roles in shaping the human gut microbiomes (Yatsunenko et al., 2012; Miller et al., 2016; De Filippo et al., 2017; Kabwe et al., 2020).

A typical analysis of unweighted UniFrac principal coordinates for 314 individuals showed that individual microbial communities were clustered mainly based on their ethnicity/geography rather than lifestyle, and the principal component analysis and principal coordinates analysis (PCoA) revealed that there is a large inter-individual compositional variation (Zhang et al., 2015). In the process of identifying the relative contributions of various factors, a recent study has found that, compared with host genetics, “environment” has a stronger ability to shape the human gut microbiome, and more than 20% of the inter-individual heterogeneities in microorganisms can be attributed to diet, drugs, and anthropometric measurements (Rothschild et al., 2018). By analyzing the gut microbiome of 7,009 individuals from 14 districts in one Chinese province, He et al. (2018) found that the geographical location of the host had the strongest correlation with gut microbiota variation. Researchers investigated the fecal microbiome of 2,084 different ethnic individuals and found that individuals living in the same city tended to have similar gut microbiome characteristics, regardless of ethnic origins (Deschasaux et al., 2018). Similar to bacterial communities, fungi in urban and rural South African individuals also exhibit different community structures (Kabwe et al., 2020). The human gut microbiome has been extensively and intensively studied from various aspects in the last decade, especially the ethnic differences among different races/ethnicities (Yap et al., 2011; Yatsunenko et al., 2012; Li et al., 2014; Obregon-Tito et al., 2015; Gomez et al., 2016; Gupta et al., 2017; Gaulke and Sharpton, 2018; Lin et al., 2020; Zuo et al., 2020; Sun et al., 2021). A recent study found that 20 host factors were significantly associated with human enterovirus variation, with geographic factors having the greatest impact and ethnically diverse diets associated with specific viral species (Zuo et al., 2020).

Understanding the relationship between human microbiome and various factors has far-reaching significance such as promoting personalized precision medicine (Herd et al., 2018). With the deepening of our understanding of the microbiome and advances in science and technology, microbiome-based diagnostic applications have been proposed in the diagnosis and prognosis of inflammatory bowel disease, pre-screening of colorectal cancer, treatment selection of melanoma, and early diagnosis and risk assessment of metabolic and cardiovascular diseases (Konstantinov et al., 2013; Tang et al., 2013; Dubinsky and Braun, 2015; Bouter et al., 2017; Versalovic et al., 2017; Routy et al., 2018). Based on metagenomic analysis, Yu et al. (2017) have even explored the potential of fecal microbiome as biomarkers for early diagnosis of colorectal cancer.

The objective of this study is to investigate the effects of ethnicities and lifestyles on the diversity scaling of the Chinese gut microbiomes. To achieve this objective, we choose to reanalyze a large dataset of over 300 Chinese covering the top seven most populous Chinese ethnic groups living rural or urban lifestyles, which was originally published by Zhang et al. (2015). Methodically, we apply the diversity–area relationship (DAR) approach extended by Ma (2018, 2019b) to reanalyze the Zhang et al. (2015) datasets. As an extension to the classic species–area relationship (SAR), the DAR model adopted Hill numbers as diversity measures to compensate for the limitation that SAR considers only species richness but ignores species richness in practical application.

## Materials and Methods

### The Chinese Gut Microbiome Dataset

We performed a secondary analysis of the Chinese gut dataset of 314 healthy young volunteers published by Zhang et al. (2015). The unrelated volunteers came from urban (145 samples) and rural (169 samples) areas in nine provinces of China, covering seven ethnic groups, namely, the Bai, Han, Kazakh, Mongol, Tibetan, Uyghur, and Zhuang, aged between 18 and 35 years. Pyrosequencing was performed on the V5–V6 region of the 16S ribosomal RNA (rRNA) gene of bacteria and archaea from 314 fecal samples, and a total of 5,102,015 high-quality sequences were generated. After operational taxonomic unit (OTU) classification by QIIME v-1.2.1 (Quantitative Insights Into Microbial Ecology, Caporaso et al., 2010), the microbial abundance information at four levels of phylum, family, genus, and species was finally obtained.

### Analysis Design Schemes

**FIGURE 1:**
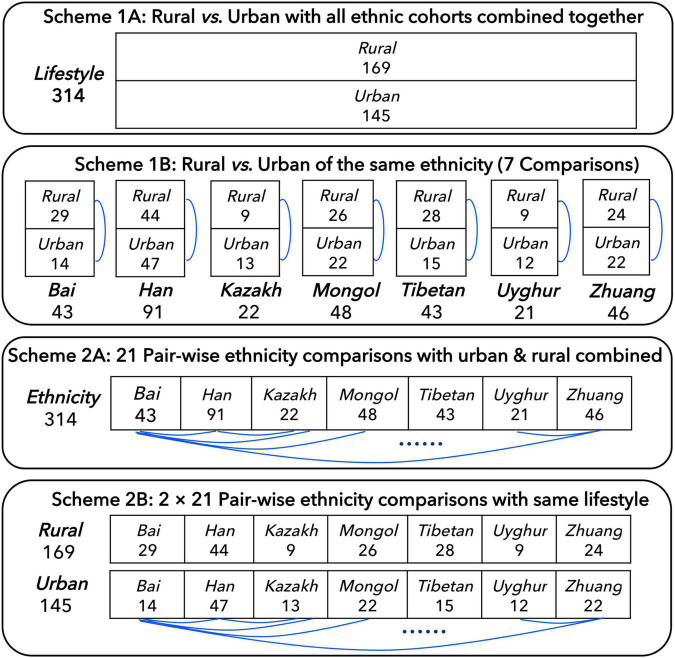
Four schemes for comparing the Chinese gut microbiome from seven major Chinese ethnic cohorts with different lifestyles, which followed Ma (2021).

In order to better compare the influence of ethnicities and lifestyles on individual gut microbiome, before the DAR analysis, we developed four schemes: (1A) the 314 individuals were divided into two cohorts according to their lifestyles (rural or urban) and compared; (1B) the 314 individuals were divided into 14 cohorts according to their ethnicities and lifestyles, and the urban and rural cohorts with the same ethnicity were compared; (2A) the 314 individuals were divided into seven cohorts according to their ethnicities with rural and urban combined and compared in pairs; and (2B) the 314 individuals were divided into 14 cohorts according to their ethnicities and lifestyles, and the different ethnic cohorts with the same lifestyle were compared (see Figure 1 for more details). In addition, we also integrated all the samples into one cohort (the total cohort) for model fitting.

### Diversity–Area Relationship Analysis

DAR analysis relies primarily on the DAR-PL (power law) and DAR-PLEC (power law with exponential cutoff) models (Ma, 2018, 2019b). Since there is no natural ordering among samples, in order to avoid the possible deviation caused by any sorting, we sorted all samples of each cohort, randomly selected 100 results for DAR model fitting, and finally calculated the mean value as the parameter of the DAR model. The DAR model used here is an extension of the classic SAR model (Plotkin et al., 2000; Ulrich and Buszko, 2003; Tjørve, 2009). Hill number diversity was introduced into the DAR model, which extended the traditional SAR model of species abundance to a more comprehensive level of diversity and quantified the variation of community diversity on the spatial scale (Chao et al., 2012, 2014a,b; Ma, 2018, 2019b). Diversity and area conform to the power law model:

(1) Dq=c⁢Az

where *^q^D* means the diversity of order *q*, *A* is the area, and *c* and *z* are heterogeneity parameters. And on that basis, Ma further extended the general power law to the PLEC model (Ma, 2018, 2019b):

(2) Dq=c⁢Az⁢exp⁢(d⁢A)

where *d* is a parameter that is usually negative in the DAR models and exp(*dA*) is responsible for the exponential decay of the PLEC model.

In order to estimate the parameters of the DAR model, linear transformation was made above the power law equation:

(3) l⁢n⁢(D)=l⁢n⁢(c)+z⁢l⁢n⁢(A)

(4) l⁢n⁢(D)=l⁢n⁢(c)+z⁢l⁢n⁢(A)+d⁢A

The linear correlation coefficient *R* and *p*-value can be used to evaluate the efficacy of the fitting of the model. Based on above models, four diversity scaling profiles were defined for characterizing the biogeography maps of the microbial community:

(*i*) The *DAR* profile describes the relationship between diversity scaling parameter (*z*) and diversity order (*q*) of the DAR model. The *DAR* profile can be used to quantify the changes of diversity scaling under different diversity order (*q*).

(*ii*) The *PDO* (pairwise diversity overlap) profile represents the relationship between the diversity overlap parameter *g* and diversity order (*q*) of the DAR-PL model.

(5) $$g=2\,\left(D_{A}-D_{2\,A}\right)/D_{A}=2-2^{z}$$

where *D*_*A*_ is the diversity of area A, *D*_2A_ is the diversity of area 2*A*, and *z* is the heterogeneity parameter of the DAR model.

(*iii*) The *MAD* (maximum accrual diversity) profile describes the relationship between maximum accrual diversity and the diversity order (*q*) and can be used to estimate the regional or global maximum theoretical value of microbial community diversity:

(6) M⁢a⁢x⁢(Dq)=Dmaxq=c⁢(-zd)z⁢exp⁢(-z)=c⁢Amaxz⁢exp⁢(-z)

where *A_max =–z/d_*, which represents the area when the diversity is maximized.

(*iv*) The *LGD* (the ratio of local diversity to global accrual diversity) profile represents the ratio of sample diversity to global diversity and it can be used to estimate the proportion of the microbial community within a region on a global scale (Li and Ma, 2019):

(7) $$L\,G\,D=c/D_{m\,a\,x}$$

where *c* is the parameter of the DAR-PL model at the diversity order of *q* and *D*_*max*_ corresponding to the diversity order can be generated according to Equation (6). After the relevant parameters of all profiles were obtained, we used a permutation (randomization) test to verify the difference of these parameters among all cohorts (Ma, 2019b; Li and Ma, 2019; Ma and Li, 2019).

## Results

### Diversity–Area Relationship Model Fitting

After fitting the DAR-PL and DAR-PLEC models for each cohort of the Chinese gut microbiome dataset, we got all parameters of the DAR analysis, including the *DAR* (*z*), *PDO* (*g*), *MAD* (*D*_*max*_), and *LGD* profiles. Table 1 shows the DAR parameters of all cohorts at the species level (for the parameters of other levels, see Supplementary Table 1). We observed that all cohorts fitted the DAR-PL and DAR-PLEC models well, with a *p*-value < 0.05. In the 100 times of reordering model fitting, especially when the diversity order *q* is 0, the 100 times of DAR-PL model fitting were all successful, with a *p*-value of 0.000. However, with the increase of the order of diversity, some model fittings failed. Similar situations also appeared in DAR-PLEC model fitting. Table 2 lists the results (percentages with significant differences) of the permutation tests for the differences in the parameters of the DAR models for the pairwise comparisons of the Chinese gut microbiome dataset (see Supplementary Table 2 for specific results).

**TABLE 1:** Fitting the DAR models for all cohorts of the Chinese gut microbiome datasets (with 100 times of random permutations of microbiome samples) (see Supplementary Table 1 for the phylum-, family-, and genus-level counterpart results; only species-level results are shown here).

Cohort	Diversity order	Power law (PL)						PL with exponential cutoff (PLEC)								
		*z*	ln(*c*)	*R*	*p*-value	*g*	*N*	*z*	*d*	ln(*c*)	*R*	*p*-value	*N*	*A*_*max*_	*D*_*max*_	*LGD*
Total	*q* = 0	0.309	6.019	0.994	0.000	0.761	100	0.351	0.000	5.896	0.997	0.000	93	1,373	2,781.4	15.2
	*q* = 1	0.065	4.423	0.745	0.000	0.954	100	0.134	-0.001	4.222	0.854	0.000	95	230	118.5	70.6
	*q* = 2	0.069	3.613	0.674	0.000	0.950	99	0.153	-0.001	3.368	0.802	0.000	94	4,503	54.5	68.6
	*q* = 3	0.076	3.231	0.683	0.000	0.946	99	0.166	-0.001	2.965	0.808	0.000	92	196	38.4	66.3
Rural	*q* = 0	0.322	6.010	0.993	0.000	0.750	100	0.370	-0.001	5.891	0.995	0.000	91	1,850	2,654.7	16.5
	*q* = 1	0.085	4.358	0.741	0.000	0.938	100	0.179	-0.002	4.136	0.852	0.000	90	107	118.1	66.8
	*q* = 2	0.095	3.501	0.681	0.000	0.931	95	0.190	-0.002	3.290	0.789	0.000	94	199	52.7	66.9
	*q* = 3	0.104	3.107	0.688	0.001	0.924	96	0.204	-0.002	2.878	0.796	0.000	90	145	37.0	63.7
Urban	*q* = 0	0.324	5.908	0.993	0.000	0.748	100	0.374	-0.001	5.794	0.995	0.000	84	1,065	2,215.8	17.3
	*q* = 1	0.089	4.328	0.813	0.000	0.936	99	0.172	-0.002	4.148	0.907	0.000	98	102	114.6	67.0
	*q* = 2	0.096	3.544	0.735	0.000	0.930	99	0.192	-0.002	3.335	0.845	0.000	97	154	54.5	65.7
	*q* = 3	0.103	3.162	0.729	0.000	0.925	98	0.199	-0.002	2.957	0.826	0.000	96	137	38.3	64.7
Bai	*q* = 0	0.226	5.869	0.952	0.000	0.830	100	0.323	-0.007	5.763	0.971	0.000	88	82	815.3	44.3
	*q* = 1	0.099	4.053	0.776	0.001	0.928	97	0.202	-0.008	3.939	0.853	0.000	81	50	82.3	71.6
	*q* = 2	0.101	3.225	0.741	0.001	0.927	87	0.212	-0.009	3.115	0.801	0.003	72	42	36.2	73.4
	*q* = 3	0.096	2.914	0.711	0.002	0.930	88	0.204	-0.008	2.797	0.807	0.001	70	64	26.2	73.1
Han	*q* = 0	0.349	5.904	0.992	0.000	0.726	100	0.413	-0.002	5.782	0.996	0.000	87	499	2,029.9	18.8
	*q* = 1	0.100	4.228	0.781	0.000	0.928	96	0.186	-0.003	4.084	0.861	0.000	94	75	105.0	67.6
	*q* = 2	0.112	3.330	0.689	0.000	0.918	87	0.201	-0.004	3.205	0.764	0.001	90	118	45.6	68.8
	*q* = 3	0.122	2.910	0.695	0.001	0.910	87	0.214	-0.004	2.787	0.762	0.001	84	67	31.1	66.9
Kazakh	*q* = 0	0.373	5.862	0.984	0.000	0.703	100	0.505	-0.015	5.738	0.989	0.000	65	174	1,377.2	28.1
	*q* = 1	0.245	3.987	0.824	0.001	0.809	96	0.472	-0.032	3.866	0.902	0.001	86	21	109.8	55.7
	*q* = 2	0.304	3.108	0.801	0.002	0.754	91	0.567	-0.038	2.988	0.882	0.001	82	17	53.5	51.6
	*q* = 3	0.313	2.765	0.804	0.001	0.746	92	0.558	-0.034	2.628	0.887	0.000	82	22	39.4	48.4
Mongol	*q* = 0	0.362	5.962	0.983	0.000	0.713	100	0.468	-0.006	5.800	0.987	0.000	69	179	1,724.9	22.5
	*q* = 1	0.129	4.299	0.774	0.001	0.905	95	0.226	-0.007	4.192	0.851	0.000	90	34	116.3	66.8
	*q* = 2	0.131	3.464	0.719	0.001	0.903	91	0.233	-0.007	3.358	0.810	0.001	87	37	51.5	67.1
	*q* = 3	0.128	3.056	0.693	0.002	0.905	86	0.230	-0.008	2.961	0.788	0.001	83	42	34.6	67.8
Tibetan	*q* = 0	0.325	5.838	0.982	0.000	0.747	100	0.413	-0.006	5.736	0.988	0.000	78	282	1,306.3	28.0
	*q* = 1	0.163	3.993	0.780	0.000	0.878	96	0.294	-0.010	3.856	0.858	0.000	90	42	96.5	60.4
	*q* = 2	0.163	3.033	0.659	0.003	0.875	77	0.264	-0.010	2.996	0.743	0.002	89	28	37.7	68.6
	*q* = 3	0.134	2.675	0.632	0.006	0.895	70	0.228	-0.010	2.659	0.727	0.003	81	26	24.7	77.0
Uyghur	*q* = 0	0.363	5.970	0.993	0.000	0.713	100	0.451	-0.010	5.886	0.996	0.000	67	113	1,465.8	27.5
	*q* = 1	0.175	4.276	0.890	0.000	0.869	99	0.344	-0.022	4.146	0.954	0.000	85	27	118.7	60.5
	*q* = 2	0.223	3.387	0.841	0.001	0.829	93	0.431	-0.029	3.250	0.910	0.001	85	23	55.2	56.5
	*q* = 3	0.234	2.984	0.814	0.001	0.820	90	0.462	-0.031	2.833	0.898	0.001	81	16	37.6	55.3
Zhuang	*q* = 0	0.354	5.803	0.991	0.000	0.722	100	0.428	-0.005	5.703	0.994	0.000	74	186	1,480.7	23.3
	*q* = 1	0.150	4.122	0.888	0.000	0.889	100	0.272	-0.009	3.978	0.953	0.000	97	47	105.3	59.6
	*q* = 2	0.156	3.293	0.798	0.000	0.884	100	0.327	-0.012	3.084	0.895	0.000	86	43	47.6	57.8
	*q* = 3	0.155	2.908	0.752	0.001	0.885	98	0.318	-0.012	2.721	0.849	0.001	87	52	32.8	59.1
Bai-Rural	*q* = 0	0.217	5.857	0.940	0.000	0.837	100	0.349	-0.014	5.748	0.974	0.000	92	45	707.7	50.5
	*q* = 1	0.122	3.985	0.768	0.001	0.910	89	0.236	-0.013	3.914	0.842	0.001	83	26	79.4	72.3
	*q* = 2	0.129	3.156	0.766	0.001	0.904	80	0.238	-0.013	3.100	0.827	0.001	80	34	35.3	72.7
	*q* = 3	0.123	2.860	0.766	0.001	0.910	80	0.237	-0.013	2.783	0.819	0.002	75	23	25.6	72.7
Bai-Urban	*q* = 0	0.317	5.720	0.938	0.000	0.751	100	0.512	-0.037	5.668	0.967	0.000	76	84	799.9	46.2
	*q* = 1	0.213	3.856	0.841	0.002	0.836	88	0.385	-0.034	3.807	0.914	0.001	87	20	81.4	63.9
	*q* = 2	0.294	2.814	0.852	0.003	0.771	60	0.462	-0.041	2.834	0.912	0.003	65	13	33.8	58.8
	*q* = 3	0.282	2.472	0.834	0.003	0.781	58	0.432	-0.041	2.543	0.893	0.004	61	13	23.9	62.6
Han-Rural	*q* = 0	0.375	5.860	0.991	0.000	0.703	100	0.449	-0.005	5.768	0.994	0.000	78	1,852	2,027.2	21.6
	*q* = 1	0.170	4.072	0.823	0.000	0.873	98	0.321	-0.011	3.894	0.883	0.000	89	46	107.6	56.9
	*q* = 2	0.197	3.116	0.722	0.002	0.850	91	0.359	-0.012	2.945	0.805	0.002	87	37	45.1	56.5
	*q* = 3	0.206	2.699	0.726	0.001	0.842	91	0.364	-0.012	2.531	0.804	0.001	81	35	30.6	55.3
Han-Urban	*q* = 0	0.354	5.866	0.991	0.000	0.721	100	0.434	-0.005	5.745	0.994	0.000	75	1,023	1,744.4	22.2
	*q* = 1	0.113	4.173	0.791	0.002	0.918	97	0.210	-0.007	4.061	0.865	0.001	91	47	98.0	68.9
	*q* = 2	0.122	3.308	0.682	0.001	0.909	89	0.237	-0.009	3.197	0.779	0.001	80	35	42.8	70.6
	*q* = 3	0.131	2.896	0.676	0.002	0.902	85	0.237	-0.009	2.811	0.768	0.002	77	72	29.5	70.8
Kazakh-Rural	*q* = 0	0.389	5.834	0.978	0.000	0.687	100	0.644	-0.058	5.757	0.987	0.000	55	20	885.0	38.3
	*q* = 1	0.262	3.910	0.838	0.010	0.787	89	0.555	-0.075	3.875	0.923	0.007	69	8	84.2	64.7
	*q* = 2	0.341	2.966	0.843	0.009	0.707	71	0.618	-0.076	2.969	0.907	0.011	63	8	38.0	61.6
	*q* = 3	0.372	2.588	0.837	0.011	0.680	70	0.641	-0.069	2.543	0.918	0.009	55	11	27.8	57.1
Kazakh-Urban	*q* = 0	0.398	5.833	0.978	0.000	0.681	100	0.555	-0.031	5.779	0.986	0.000	74	35	1,061.5	33.8
	*q* = 1	0.326	3.950	0.882	0.001	0.738	99	0.627	-0.059	3.847	0.940	0.000	79	23	123.0	47.4
	*q* = 2	0.384	3.133	0.861	0.002	0.680	98	0.671	-0.059	3.062	0.925	0.001	75	13	56.5	48.6
	*q* = 3	0.384	2.811	0.859	0.003	0.679	97	0.611	-0.046	2.750	0.915	0.002	76	17	42.2	48.5
Mongol-Rural	*q* = 0	0.336	6.199	0.990	0.000	0.737	100	0.428	-0.009	6.099	0.994	0.000	66	81	1,671.5	29.1
	*q* = 1	0.129	4.438	0.708	0.004	0.904	83	0.204	-0.010	4.414	0.818	0.002	78	16	122.9	75.0
	*q* = 2	0.158	3.521	0.687	0.006	0.879	62	0.228	-0.014	3.574	0.773	0.003	75	65	57.2	75.2
	*q* = 3	0.128	3.174	0.672	0.006	0.900	60	0.193	-0.013	3.233	0.756	0.004	74	20	37.5	82.0
Mongol-Urban	*q* = 0	0.362	5.609	0.994	0.000	0.715	100	0.424	-0.008	5.562	0.997	0.000	80	371	1,222.5	26.4
	*q* = 1	0.192	3.975	0.918	0.000	0.855	100	0.319	-0.017	3.889	0.955	0.000	91	30	93.5	58.2
	*q* = 2	0.205	3.163	0.870	0.002	0.843	96	0.346	-0.020	3.085	0.913	0.001	85	64	43.5	58.7
	*q* = 3	0.211	2.781	0.854	0.000	0.839	92	0.361	-0.021	2.701	0.897	0.001	78	32	30.0	58.8
Tibetan-Rural	*q* = 0	0.375	5.734	0.987	0.000	0.702	100	0.492	-0.011	5.615	0.992	0.000	74	73	1,168.1	26.3
	*q* = 1	0.238	3.768	0.819	0.000	0.816	98	0.426	-0.021	3.627	0.895	0.001	92	54	92.0	52.1
	*q* = 2	0.247	2.703	0.756	0.002	0.806	74	0.427	-0.026	2.688	0.819	0.002	80	19	33.3	61.9
	*q* = 3	0.207	2.334	0.730	0.003	0.836	68	0.389	-0.025	2.311	0.811	0.002	72	21	21.2	68.8
Tibetan-Urban	*q* = 0	0.284	5.874	0.924	0.000	0.781	100	0.298	-0.004	5.890	0.952	0.000	64	41	779.5	53.9
	*q* = 1	0.193	3.997	0.756	0.006	0.848	78	0.301	-0.020	3.960	0.862	0.003	64	9	83.8	72.5
	*q* = 2	0.257	3.057	0.726	0.009	0.790	65	0.318	-0.014	3.061	0.840	0.005	62	12	38.2	69.0
	*q* = 3	0.266	2.699	0.716	0.011	0.781	61	0.291	-0.008	2.701	0.832	0.007	63	11	27.2	68.7
Uyghur-Rural	*q* = 0	0.380	5.872	0.995	0.000	0.698	100	0.485	-0.024	5.837	0.997	0.000	59	57	1,083.5	34.5
	*q* = 1	0.280	4.122	0.905	0.003	0.779	98	0.489	-0.051	4.071	0.964	0.002	85	10	108.0	58.9
	*q* = 2	0.494	2.971	0.917	0.003	0.577	71	0.733	-0.078	3.043	0.952	0.005	71	8	50.9	49.1
	*q* = 3	0.553	2.509	0.924	0.002	0.517	67	0.823	-0.083	2.558	0.958	0.003	64	18	43.4	42.8
Uyghur-Urban	*q* = 0	0.377	6.006	0.990	0.000	0.700	100	0.497	-0.024	5.960	0.995	0.000	71	36	1,182.1	34.9
	*q* = 1	0.208	4.244	0.915	0.001	0.841	94	0.416	-0.044	4.181	0.954	0.002	80	16	113.2	62.7
	*q* = 2	0.246	3.374	0.852	0.005	0.808	86	0.476	-0.050	3.318	0.918	0.005	81	11	49.9	61.9
	*q* = 3	0.264	2.937	0.845	0.005	0.794	75	0.519	-0.057	2.888	0.912	0.005	73	9	33.9	59.6
Zhuang-Rural	*q* = 0	0.368	5.826	0.989	0.000	0.708	100	0.475	-0.011	5.723	0.993	0.000	71	128	1,301.7	26.9
	*q* = 1	0.178	3.993	0.903	0.000	0.867	100	0.333	-0.019	3.872	0.947	0.000	86	27	92.2	59.5
	*q* = 2	0.192	3.090	0.856	0.001	0.855	98	0.387	-0.023	2.925	0.923	0.001	78	50	39.1	56.8
	*q* = 3	0.195	2.691	0.826	0.000	0.852	96	0.398	-0.023	2.521	0.905	0.000	72	25	26.5	56.6
Zhuang-Urban	*q* = 0	0.370	5.707	0.991	0.000	0.707	100	0.449	-0.010	5.655	0.994	0.000	65	597	1,266.4	27.0
	*q* = 1	0.232	4.024	0.934	0.000	0.823	100	0.394	-0.022	3.925	0.973	0.000	89	57	112.7	52.0
	*q* = 2	0.271	3.200	0.880	0.000	0.787	100	0.495	-0.031	3.083	0.946	0.000	79	26	54.5	50.3
	*q* = 3	0.275	2.822	0.844	0.001	0.783	99	0.489	-0.031	2.730	0.912	0.001	78	59	39.4	50.7

**TABLE 2:** The results (percentages with significant differences) of the permutation tests for the differences in the parameters of the DAR models for the pairwise comparisons of Chinese gut microbiome datasets [see Supplementary Table 2 for specific results (*p*-value) of the four schemes (1A–2B)].

Scheme 1A									
**Diversity order**	**Rural vs. Urban with all 7 ethnicity combined**	**PL**		**PLEC**					
		***z***	**ln(*c*)**	***z***	***d***	**ln(*c*)**	***A*_*max*_**	***D*_*max*_**	***LGD***
*q* = 0	Four taxon levels (%) with significant differences	0	0	0	0	0	25% (1/4)	0	0
*q* = 1	Four taxon levels (%) with significant differences	0	0	0	0	0	0	0	0
*q* = 2	Four taxon levels (%) with significant differences	0	0	0	0	0	25% (1/4)	0	0
*q* = 3	Four taxon levels (%) with significant differences	0	0	0	0	0	0	0	0
**Scheme 1B**									

**Diversity order**	**Rural vs. urban of each ethnicity**	**PL**		**PLEC**					
		***z***	**ln(*c*)**	***z***	***d***	**ln(*c*)**	***A*_*max*_**	***D*_*max*_**	***LGD***

*q* = 0	Four taxon levels (%) with significant differences	0	0	0	0	0	3.5% (1/28)	0	3.5% (1/28)
*q* = 1	Four taxon levels (%) with significant differences	0	0	0	0	0	3.5% (1/28)	0	0
*q* = 2	Four taxon levels (%) with significant differences	0	0	0	0	0	3.5% (1/28)	0	0
*q* = 3	Four taxon levels (%) with significant differences	0	0	0	0	0	0	0	0
**Scheme 2A**									

**Diversity order**	**Pairwise ethnicity comparisons with rural and urban combined**	**PL**		**PLEC**					
		***z***	**ln(*c*)**	***z***	***d***	**ln(*c*)**	***A*_*max*_**	***D*_*max*_**	***LGD***

*q* = 0	Four taxon levels (%) with significant differences	2.3% (2/84)	0	0	0	0	2.3% (2/84)	0	3.6% (3/84)
*q* = 1	Four taxon levels (%) with significant differences	0	0	0	0	0	2.3% (2/84)	9.5% (8/84)	0
*q* = 2	Four taxon levels (%) with significant differences	0	0	0	0	0	7.1% (6/84)	13.1% (11/84)	0
*q* = 3	Four taxon levels (%) with significant differences	0	0	0	0	0	0	8.3% (7/84)	0
**Scheme 2B**									

**Diversity order**	**Pairwise comparison of each ethnicity for rural lifestyle**	**PL**		**PLEC**					
		***z***	**ln(*c*)**	***z***	***d***	**ln(*c*)**	***A*_*max*_**	***D*_*max*_**	***LGD***

*q* = 0	Four taxon levels (%) with significant differences	2.3% (2/84)	0	0	0	0	7.1% (6/84)	1.2% (1/84)	3.6% (3/84)
*q* = 1	Four taxon levels (%) with significant differences	0	0	0	0	0	7.1% (6/84)	6.0% (5/84)	0
*q* = 2	Four taxon levels (%) with significant differences	0	0	0	0	0	0	7.1% (6/84)	0
*q* = 3	Four taxon levels (%) with significant differences	0	0	0	0	0	0	9.5% (8/84)	0

**Diversity order**	**Pairwise comparison of each ethnicity for urban lifestyle**	**PL**		**PLEC**					
		***z***	**ln(*c*)**	***z***	***d***	**ln(*c*)**	***A*_*max*_**	***D*_*max*_**	***LGD***

*q* = 0	Four taxon levels (%) with significant differences	0	0	0	0	0	7.1% (6/84)	0	2.3% (2/84)
*q* = 1	Four taxon levels (%) with significant differences	0	0	0	0	0	1.2% (1/84)	3.6% (3/84)	0
*q* = 2	Four taxon levels (%) with significant differences	0	0	0	0	0	0	6.0% (5/84)	0
*q* = 3	Four taxon levels (%) with significant differences	0	0	0	0	0	4.8% (4/84)	7.1% (6/84)	0

### Four Diversity–Area Relationship Profiles

**FIGURE 2:**
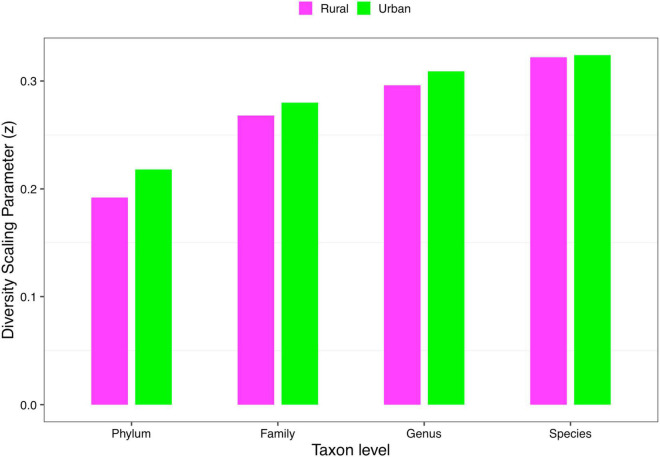
The DAR profiles of the rural and urban cohorts at phylum, family, genus, and species levels when diversity order *q* = 0 (Scheme 1A).

(*i*) *DAR* profile: The diversity scaling parameter (*z*) was defined as the *DAR* profile, which quantitatively describes community similarity. The higher the diversity scaling parameter *z* is, the greater are the differences between individuals in the community. With the gradual refinement of the taxonomy level, the inter-individual heterogeneity in almost all the cohorts of four schemes progressively increased, as well as the total cohort (phylum: *z* = 0.169; family: *z* = 0.255; genus: *z* = 0.288; species: *z* = 0.309, when diversity order *q* = 0). Based on Scheme 1A, for the rural and urban cohorts, from the phylum level to the species level, the urban individuals had a higher diversity heterogeneity at diversity order *q* = 0. The parameter of diversity scaling (*z*) of each cohort increased gradually and always kept the trend of the rural cohort being smaller than the urban cohort at *q* = 0 (Figure 2). We then used Scheme 1B to further determine the influence of lifestyles on the diversity scaling of Chinese gut microbiome. We compared the *DAR* profiles of urban and rural individuals in the same ethnic group, and there was no statistically significant difference.

**FIGURE 3:**
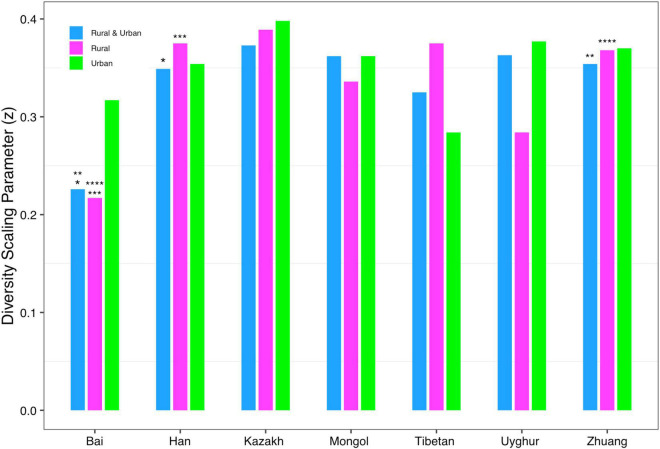
The DAR profiles of all cohorts from Schemes 2A and 2B at the species level when diversity order *q* = 0 (the same number of “*, **, ***, ****” indicates a significant difference in pairwise comparison).

**FIGURE 4:**
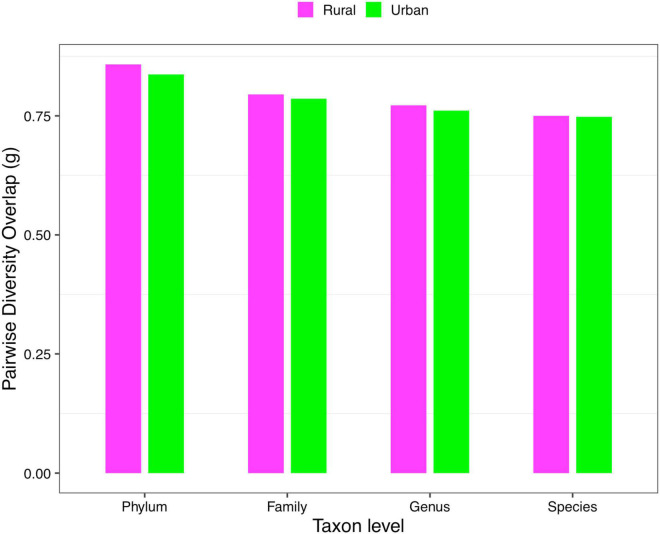
The PDO profiles of the rural and urban cohorts at phylum, family, genus, and species levels when diversity order *q* = 0 (Scheme 1A).

**FIGURE 5:**
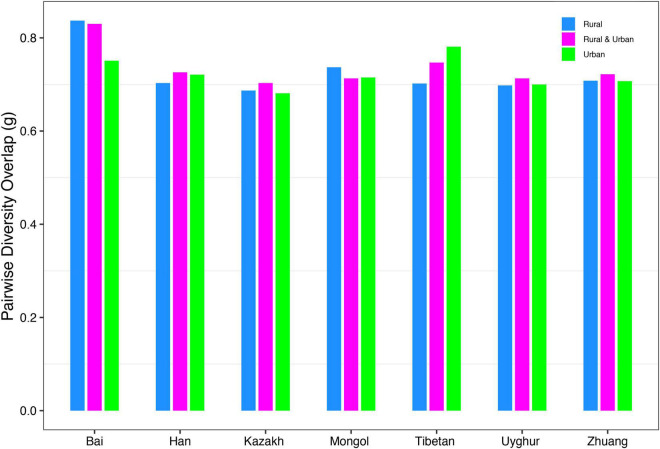
The PDO profiles of all cohorts from Schemes 2A and 2B at the species level when diversity order *q* = 0.

**FIGURE 6:**
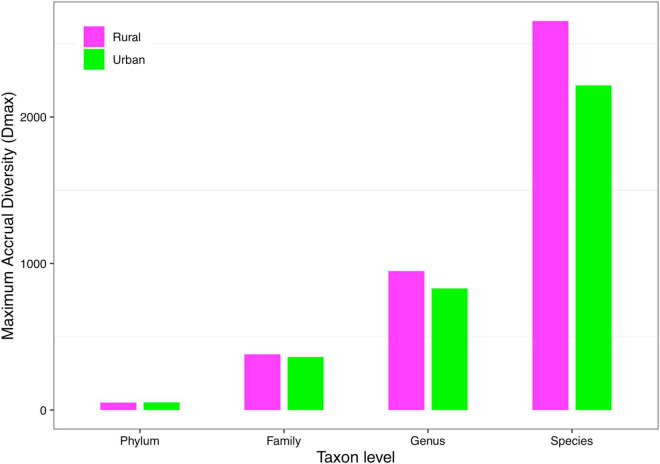
The MAD profiles of the rural and urban cohorts at phylum, family, genus, and species levels when diversity order *q* = 0 (Scheme 1A).

**FIGURE 7:**
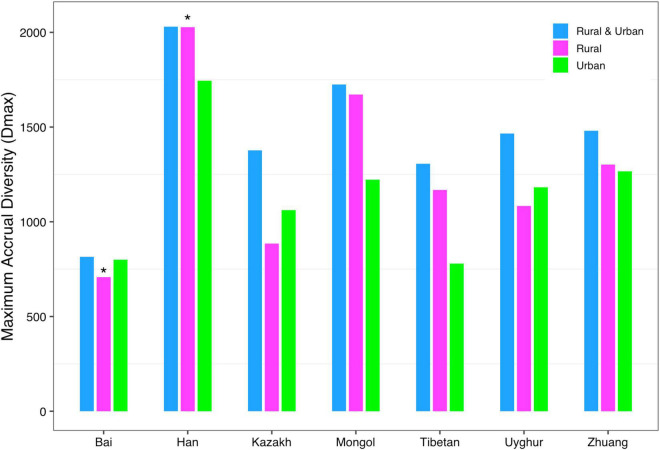
The MAD profiles of all cohorts from Schemes 2A and 2B at the species level when diversity order *q* = 0 (“*” indicated a significant difference between pairwise comparisons).

In order to detect the impact of ethnicities on the *DAR* profiles, we set up 1,008 pairwise comparisons from phylum to species levels when diversity order *q* = 0–3 according to Schemes 2A and 2B (Scheme 2A: seven ethnic cohorts with urban and rural combined, 336 pairwise comparisons; Scheme 2B: cohorts with the same lifestyles but different ethnicities, 672 pairwise comparisons). We noticed that there were four pairwise comparisons of the *DAR* profiles showing statistically significant differences at the species level when diversity order *q* = 0 (Bai vs. Han; Bai vs. Zhuang; Bai-Rural vs. Han-Rural; Bai-Rural vs. Zhuang-Rural cohorts). Figure 3 displays the *DAR* profiles of all cohorts from Schemes 2A and 2B at the species level when diversity order *q* = 0. Different from lifestyles, we found that ethnicities influenced the diversity heterogeneity of the Chinese gut microbiome significantly.

(*ii*) *PDO* profile: The parameter of pairwise diversity overlap (*g*) was defined as the *PDO* profile, which also quantitatively describes the community similarity. The larger the parameter *g* is, the higher the overlap is, that is, the smaller the diversity heterogeneity among individuals in the community. In contrast to the *DAR* profile, with the gradual refinement of the taxonomy level and the rise of the diversity order, the *PDO* profile of each cohort showed a general downward trend (Figure 4). According to Scheme 1A, we noticed that the *PDO* profile presented a trend of the rural cohort being higher than the urban cohort. As for Scheme 1B, we found the *PDO* profiles of urban and rural cohorts of the same ethnic group were similar at various taxonomy levels when diversity order *q* = 0, except for the Bai nationality. Based on Scheme 2A, we noticed that the *PDO* profiles of the other ethnic groups, except the Bai, were relatively close. Figure 5 displayed the *PDO* profiles of all cohorts from Schemes 2A and 2B at the species level when diversity order *q* = 0.

(*iii*) *MAD* profile: The maximum accrual diversity (*D*_*max*_) was defined as the *MAD* profile. When diversity order *q* = 0, *D*_*max*_ is the maximum estimated diversity within a community, which means the maximum number of phylum, family, genus, and species. The *D*_*max*_ of the total cohort was always the largest in all cohorts at the four taxonomy levels when diversity order *q* = 0. According to Scheme 1A, we found that the *MAD* profile of the rural cohort was lower than that of the urban cohort at the phylum level, but the opposite trend was shown at other taxonomy levels (Figure 6). The *MAD* profiles of the rural and urban cohorts of each ethnic group did not change as consistently as the rise of the taxonomy level based on Scheme 1B. In addition, we found that the potential maximum species number of the Bai nationality was lowest in seven ethnic groups according to Schemes 2A and 2B, although the numbers of potential maximum phyla, families, and genera of the Bai nationality were similar to those of other ethnic groups. Figure 7 shows the *MAD* profiles of seven ethnic groups at the species level when diversity order *q* = 0 (Schemes 2A and 2B).

**FIGURE 8:**
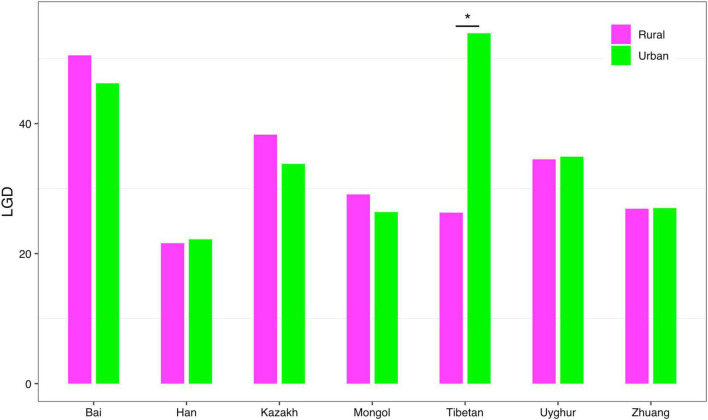
The LGD profiles of all cohorts from Scheme 1B at the species level when diversity order *q* = 0 (“*” indicated a significant difference between pairwise comparisons).

By doing permutation tests for 1,136 pairwise comparisons based on four schemes (1A–2B), we found that only one pairwise comparison (the *D*_*max*_ of the Bai-Rural vs. Han-Rural: 815.3 vs. 2,029.9) differed significantly at the species level when diversity order *q* = 0. It was undeniable that ethnicity had a statistically significant impact on the potential maximal species richness in the Bai-Rural cohort and the Han-Rural cohorts. Especially with the increase of the diversity order, significant differences were found in more than 50 pairwise comparisons of two schemes (2A and 2B) according to the results of Table 2. These findings suggested that although ethnicities did not influence the potential maximal species richness of most communities, it significantly affected the potential maximal diversity of dominant phylum/family/genus/species. Because when *q* > 0, the *MAD* profile described the diversity of dominant phylum/family/genus/species. Interestingly, we did not observe similar effects of lifestyles on the *MAD* profile.

(*iv*) *LGD* profile: The *LGD* profile estimated the ratio of the microbiome diversity within the local scale on a global scale, that is, the ratio of human gut microbiome diversity in the global ecosystem. In Scheme 1A, the *LGD* profile of the rural cohort is higher than that of the urban cohort at the first three taxonomy levels. According to Scheme 1B, we did not observe similar trends. Figure 8 exhibited the *LGD* ratio of urban and rural cohorts for each race at the species level when diversity order *q* = 0. At the species level, except for the Bai, Kazakh, and Mongol nationalities, the *LGD* profiles of other ethnic groups were smaller in the rural areas than in the urban areas; especially for the Tibetans, we have found a significant difference between Tibetan-Rural and Tibetan-Urban cohorts. Based on Scheme 2A, we found that the *LGD* profiles of all cohorts decreased with the refinement of taxonomy level, except for the Bai cohort.

**FIGURE 9:**
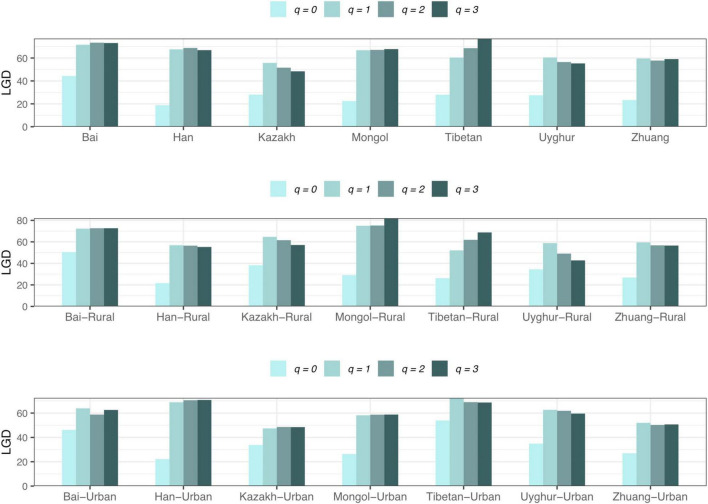
The LGD profiles of all cohorts from Schemes 2A and 2B at the species level.

For the *LGD* profile, significant differences were found in 9 of the 1,136 pairwise comparisons for Schemes 1A–2B (Tibetan-Rural vs. Tibetan-Urban; Bai vs. Han; Bai vs. Mongol; Bai vs. Zhuang; Bai-Rural vs. Han-Rural; Bai-Rural vs. Tibetan-Rural; Bai-Rural vs. Zhuang-Rural; Han-Urban vs. Tibetan-Urban; and Tibetan-Urban vs. Zhuang-Urban). Figure 9 displayed the *LGD* ratio of all cohorts for Schemes 2A and 2B at the species level.

## Conclusions and Discussion

We applied the DAR model to explore the influence of ethnicity and lifestyle on the Chinese gut microbiome, by extending the SAR model from species richness to general biodiversity metrics (in Hill numbers) and providing tools to estimate some key biodiversity parameters such as maximal accrual diversity or potential diversity (also known as dark diversity), which includes diversities that are absent locally but present in the regional species pool (so that they may appear at some time point) (Ma, 2019a). Our study tested whether ethnicities and lifestyles influence the DAR parameters based on four design schemes (Figure 1).

Firstly, we found that there were significant differences in diversity scaling (*DAR* profile) in only 0.40% (4/1,008) of pairwise comparisons on ethnic groups (i.e., Bai vs. Han; Bai vs. Zhuang; Bai-Rural vs. Han-Rural; and Bai-Rural vs. Zhuang-Rural). The 0.40% pairwise comparisons with significant differences all occurred when diversity order *q* = 0, but there was no significant difference when *q* > 0. In contrast, lifestyles had no significant effect on *DAR* profiles at all diversity orders. In general, ethnicity and lifestyle have no effect on DAR profiles. These results also suggested that the structure of the dominant species in the Chinese gut microbiome might be stable and not affected by ethnicities or lifestyles, possibly because these species (i.e., *Eubacterium rectale*, *Faecalibacterium prausnitzii*, and *Veillonella atypical*) are related to important functions (i.e., metabolism) (Sokol et al., 2008; Berni Canani et al., 2012; Han et al., 2020). Moreover, there were significant differences only at the species level and none at higher taxonomy levels. These findings indicated that compared with the species level, higher taxonomy levels of Chinese gut microbiome were more stable without the influences of ethnicities and lifestyles. The *PDO* profiles estimated the pairwise diversity overlaps between two communities. In the comparison of the *PDO* and *DAR* profiles, it is not hard to see that the two profiles show reciprocal patterns (Figures 4–7). This is because the *PDO* profile is a precise function of the *DAR* profile (Equation 5), and the *PDO* profile provides a more intuitive and convenient measure of community overlap than the *DAR* profile.

Secondly, for the *MAD* profile, only one pairwise comparison (Bai-Rural vs. Han-Rural) differed significantly at the species level when diversity order *q* = 0, and 60 pairwise comparisons differed significantly when diversity order *q* > 0. A total of 1,008 pairwise comparisons were used to detect the impact of ethnicities, and 6.1% (61/1,008) of the comparisons showed significant differences. The numbers of pairwise comparisons with significant differences at phylum, family, genus, and species levels were 37, 4, 8, and 10, respectively. Similar to findings in the *DAR* profiles, lifestyles had no significant effect on *MAD* profiles at all diversity orders. We speculated that the main reason for the difference between Bai-Rural and Han-Rural might be that the individuals of the Han cohort came from six countries in four provinces, while the individuals of the Bai cohort came from only two countries in one province. This results in a significant difference of *A*_*max*_ between Bai-Rural and Han-rural in the DAR models, which in turn affects the *MAD* profile. In addition, Li et al. (2015) found that the infection risk of *Toxoplasma gondii* in the Bai nationality of Dali was much higher than the national average, and they believed that the habit of eating raw pork and/or liver might be a potential risk of infection with *T. gondii*. We considered that this special dietary habit might also affect gut microbes. Of course, gut microbes are affected by many factors, and the specific reasons may need further investigation.

In addition, for the *LGD* profile, we noted that 0.79% (8/1,008) pairwise comparisons showed that ethnicities had a significant influence on *LGD* profiles (Bai vs. Han; Bai vs. Mongol; Bai vs. Zhuang; Bai-Rural vs. Han-Rural; Bai-Rural vs. Tibetan-Rural; Bai-Rural vs. Zhuang-Rural; Han-Urban vs. Tibetan-Urban; and Tibetan-Urban vs. Zhuang-Urban). The 0.78% (1/128) pairwise comparisons showed that lifestyles had a significant impact on *LGD* profile (Tibetan-Rural vs. Tibetan-Urban). Same as *DAR* profiles, there were significant differences on *LGD* profiles at the species level when diversity order *q* = 0, and there is no significant difference when *q* > 0 or at other taxonomy levels.

Across the three profiles, lifestyle affected only 0.78% of pairwise comparisons in the *LGD* profiles and not the other two profiles, while ethnicity affected 0.40, 6.1, and 0.79% pairwise comparisons in *DAR*, *MAD*, and *LGD*, respectively. These findings suggested that the influence of lifestyle on DAR parameters was negligible, and the influence of ethnicity was only reflected in few pairwise comparisons. Ethnicity seems to have stronger effects on DAR parameters than lifestyle, which is also consistent with the research of Zhang et al. (2015) that subjects mainly clustered by their ethnicity/geography, not lifestyle. These might be the result of China’s rapid urbanization during the last few decades, which has brought rural and urban lifestyles closer together. The influence of ethnicity on these profiles may be attributed to its genetic effect. Existing studies have also shown that racial/ethnic background strongly influences metabolism and microbes (Human Microbiome Project Consortium, 2012; Dehingia et al., 2017). A study in the United Kingdom involving 416 pairs of twins also suggested that host genes determine gut microbiome and obesity phenotype (Goodrich et al., 2014). In addition, the effect on DAR parameters was mainly concentrated when diversity order *q* > 0, and we deduced that this is due to the small sample size of this study or other confounding factors, such as geography. Therefore, the future studies may try to collect more samples of the Chinese gut microbiome in more areas to verify and expand our conclusions.

## Data Availability Statement

Publicly available datasets were analyzed in this study. This data can be found here: https://www.mg-rast.org/, mgp1538.

## Author Contributions

ZM and LD designed the study and revised the manuscript. WX and DG performed the data analysis and wrote the draft. HC and YQ performed the data curation. All authors approved the submission.

## Conflict of Interest

The authors declare that the research was conducted in the absence of any commercial or financial relationships that could be construed as a potential conflict of interest.

## Publisher’s Note

All claims expressed in this article are solely those of the authors and do not necessarily represent those of their affiliated organizations, or those of the publisher, the editors and the reviewers. Any product that may be evaluated in this article, or claim that may be made by its manufacturer, is not guaranteed or endorsed by the publisher.
