# Supplementary material for: Diversity Scaling Analysis of Chinese Gut Microbiomes Across Ethnicities and Lifestyles
Source: Front Microbiol. 2021 Dec 8;12:736393. doi: 10.3389/fmicb.2021.736393 (PMC8692740; doi:10.3389/fmicb.2021.736393)
Supplement: Supplementary file 1 [file Data_Sheet_1.ZIP › TableS1-S2/CGP-Tables S1-S2.pdf]

Tables S1-S2

Table S1. Fitting the DAR (diversity-area relationship) models for all cohorts of the Chinese gut microbiome datasets (with 100 times of random permutations of microbiome samples) for the phylum-, family- and genus-level counterpart results

Taxon	Cohort	Diversity Order	Power Law (PL)						PL with Exponential Cutoff (PLEC)								
			z	ln(c)	R	p-value	g	N	z	d	ln(c)	R	p-value	N	A_{max}	D_{max}	LGD
Phylum	Total	$q = 0$	0.169	3.002	0.966	0.000	0.876	100	0.228	-0.001	2.833	0.986	0.000	97	558	51.8	39.3
		$q = 1$	0.009	1.092	0.497	0.001	0.994	86	0.024	0.000	1.049	0.618	0.001	83	200	3.1	95.8
		$q = 2$	0.000	0.669	0.488	0.002	1.000	87	0.006	0.000	0.653	0.612	0.000	83	844	2.0	100.6
		$q = 3$	-0.001	0.542	0.502	0.002	1.001	84	0.002	0.000	0.529	0.610	0.001	80	200	1.7	100.6
	Rural	$q = 0$	0.192	2.914	0.977	0.000	0.858	100	0.238	-0.001	2.810	0.986	0.000	89	407	50.1	37.4
		$q = 1$	0.020	1.068	0.595	0.001	0.985	90	0.037	0.000	1.031	0.685	0.001	84	364	3.2	91.7
		$q = 2$	0.007	0.670	0.572	0.001	0.995	91	0.010	0.000	0.663	0.655	0.001	85	203	2.0	97.9
		$q = 3$	0.003	0.550	0.579	0.000	0.998	89	0.000	0.000	0.559	0.671	0.001	83	341	1.8	100.8
	Urban	$q = 0$	0.218	2.833	0.981	0.000	0.837	100	0.268	-0.001	2.730	0.989	0.000	77	344	51.8	33.5
		$q = 1$	0.018	1.009	0.581	0.003	0.987	90	0.033	0.000	0.986	0.668	0.001	81	90	3.0	93.9
		$q = 2$	0.005	0.605	0.584	0.002	0.996	84	0.006	0.000	0.601	0.668	0.001	82	247	1.9	100.0
		$q = 3$	0.002	0.489	0.596	0.001	0.999	82	0.001	0.000	0.490	0.667	0.001	83	123	1.6	100.4
	Bai	$q = 0$	0.242	2.763	0.964	0.000	0.817	100	0.288	-0.004	2.761	0.974	0.000	42	123	43.4	40.2
		$q = 1$	0.038	0.942	0.662	0.005	0.972	78	0.064	-0.003	0.945	0.721	0.002	71	32	3.0	93.2
		$q = 2$	0.009	0.606	0.673	0.003	0.992	76	0.017	-0.001	0.619	0.715	0.003	76	36	1.9	101.1
		$q = 3$	0.001	0.510	0.699	0.003	0.998	71	0.001	0.000	0.523	0.735	0.004	72	29	1.7	102.4
	Han	$q = 0$	0.186	2.905	0.968	0.000	0.862	100	0.243	-0.002	2.808	0.981	0.000	88	237	42.9	43.7
		$q = 1$	0.019	0.894	0.622	0.002	0.987	83	0.036	-0.001	0.866	0.695	0.001	74	73	2.6	94.2
		$q = 2$	0.001	0.513	0.601	0.002	0.999	83	0.000	0.000	0.519	0.682	0.001	80	113	1.7	102.8
		$q = 3$	-0.002	0.410	0.595	0.002	1.001	84	-0.006	0.000	0.421	0.686	0.001	79	87	1.5	102.7
	Kazakh	$q = 0$	0.219	2.814	0.974	0.000	0.835	100	0.292	-0.009	2.768	0.983	0.000	74	55	34.3	49.7
		$q = 1$	0.076	1.130	0.765	0.002	0.943	73	0.151	-0.011	1.095	0.817	0.004	75	17	3.9	84.0
		$q = 2$	0.026	0.764	0.769	0.003	0.979	79	0.069	-0.006	0.733	0.830	0.003	74	19	2.3	95.7
		$q = 3$	0.008	0.644	0.774	0.003	0.992	80	0.031	-0.003	0.627	0.844	0.002	75	20	2.0	101.5
	Mongol	$q = 0$	0.219	2.887	0.978	0.000	0.836	100	0.272	-0.003	2.821	0.985	0.000	73	259	47.1	39.6
		$q = 1$	0.018	1.101	0.636	0.005	0.987	75	0.025	-0.001	1.097	0.722	0.003	83	35	3.2	95.3
		$q = 2$	0.001	0.714	0.662	0.002	0.999	71	-0.006	0.001	0.715	0.714	0.002	83	64	2.0	100.3
		$q = 3$	-0.003	0.590	0.635	0.005	1.002	78	-0.013	0.001	0.598	0.717	0.002	84	46	1.8	102.1
	Tibetan	$q = 0$	0.238	2.681	0.976	0.000	0.821	100	0.299	-0.004	2.599	0.984	0.000	50	348	42.9	34.9
		$q = 1$	0.053	1.044	0.705	0.002	0.961	82	0.090	-0.003	1.024	0.777	0.001	78	62	3.5	86.3
		$q = 2$	0.028	0.757	0.629	0.006	0.979	74	0.040	-0.002	0.771	0.723	0.002	80	26	2.4	95.5
		$q = 3$	0.021	0.669	0.632	0.004	0.984	69	0.026	-0.001	0.683	0.708	0.003	76	38	2.1	96.8
	Uyghur	$q = 0$	0.205	2.955	0.971	0.000	0.847	100	0.286	-0.011	2.906	0.985	0.000	75	97	37.3	52.5
		$q = 1$	0.042	1.210	0.748	0.003	0.969	73	0.063	-0.004	1.214	0.823	0.003	74	19	3.8	92.5
		$q = 2$	0.003	0.819	0.730	0.004	0.995	79	-0.009	0.001	0.846	0.822	0.004	73	16	2.3	105.5
		$q = 3$	-0.010	0.690	0.731	0.005	1.005	78	-0.038	0.003	0.727	0.822	0.004	74	18	2.0	108.9
	Zhuang	$q = 0$	0.240	2.762	0.980	0.000	0.819	100	0.302	-0.004	2.680	0.986	0.000	68	944	45.6	36.6
		$q = 1$	0.039	0.936	0.635	0.004	0.972	76	0.066	-0.003	0.941	0.731	0.001	74	242	3.0	93.0
		$q = 2$	0.008	0.568	0.631	0.003	0.994	73	0.014	-0.001	0.576	0.733	0.002	70	25	1.8	100.7
		$q = 3$	0.000	0.471	0.629	0.003	0.999	74	0.002	0.000	0.474	0.734	0.002	68	49	1.6	102.4
	Bai-Rural	$q = 0$	0.227	2.756	0.955	0.000	0.829	100	0.304	-0.010	2.744	0.965	0.000	49	47	34.2	49.8
		$q = 1$	0.059	0.873	0.704	0.004	0.957	77	0.117	-0.008	0.859	0.780	0.002	78	20	2.9	88.1
		$q = 2$	0.021	0.548	0.710	0.002	0.984	73	0.058	-0.004	0.524	0.789	0.001	72	31	1.9	95.7
		$q = 3$	0.010	0.457	0.702	0.003	0.992	77	0.038	-0.003	0.431	0.794	0.002	73	33	1.7	97.4
	Bai-Urban	$q = 0$	0.240	2.785	0.960	0.000	0.818	100	0.310	-0.013	2.768	0.972	0.000	47	81	33.6	52.4
		$q = 1$	0.078	0.890	0.780	0.006	0.943	52	0.119	-0.012	0.930	0.837	0.007	62	9	3.0	88.5
		$q = 2$	0.041	0.599	0.771	0.008	0.969	55	0.069	-0.006	0.606	0.848	0.005	62	13	2.0	94.2
		$q = 3$	0.023	0.526	0.789	0.006	0.981	53	0.035	-0.004	0.545	0.844	0.006	68	18	1.8	99.2

	Han-Rural	$q = 0$	0.197	2.899	0.967	0.000	0.854	100	0.279	-0.006	2.800	0.983	0.000	84	68	37.5	48.5
		$q = 1$	0.028	0.935	0.662	0.004	0.979	68	0.043	-0.002	0.950	0.694	0.004	71	45	2.9	95.6
		$q = 2$	-0.006	0.593	0.628	0.003	1.003	74	-0.002	0.000	0.578	0.693	0.002	74	59	1.8	101.2
		$q = 3$	-0.012	0.489	0.618	0.004	1.007	77	-0.016	0.001	0.484	0.696	0.001	72	45	1.6	103.8
	Han-Urban	$q = 0$	0.221	2.801	0.972	0.000	0.835	100	0.292	-0.005	2.709	0.981	0.000	63	271	40.9	41.0
		$q = 1$	0.017	0.824	0.674	0.003	0.988	75	0.026	-0.001	0.822	0.762	0.001	79	39	2.4	96.5
		$q = 2$	0.001	0.455	0.655	0.002	0.999	74	0.001	0.000	0.456	0.748	0.001	80	32	1.6	100.7
		$q = 3$	-0.001	0.359	0.657	0.001	1.000	73	-0.003	0.000	0.362	0.754	0.001	78	34	1.4	101.0
	Kazakh-Rural	$q = 0$	0.208	2.846	0.949	0.000	0.844	100	0.348	-0.038	2.860	0.974	0.000	60	23	28.3	63.2
		$q = 1$	0.051	1.069	0.839	0.011	0.959	49	0.108	-0.013	1.046	0.899	0.012	50	6	3.2	91.7
		$q = 2$	0.002	0.663	0.852	0.011	0.994	46	0.032	0.000	0.601	0.899	0.012	41	13	1.9	99.0
		$q = 3$	-0.012	0.544	0.855	0.011	1.004	45	-0.010	0.005	0.501	0.905	0.011	41	11	1.7	103.1
	Kazakh-Urban	$q = 0$	0.243	2.781	0.967	0.000	0.816	100	0.324	-0.017	2.773	0.982	0.000	59	116	32.3	53.9
		$q = 1$	0.124	1.125	0.815	0.004	0.907	54	0.164	-0.016	1.199	0.862	0.006	65	13	4.1	85.1
		$q = 2$	0.050	0.816	0.789	0.007	0.959	54	0.077	-0.009	0.835	0.859	0.006	64	9	2.6	95.5
		$q = 3$	0.012	0.729	0.781	0.009	0.988	60	0.034	-0.003	0.694	0.868	0.005	62	8	2.1	98.8
	Mongol-Rural	$q = 0$	0.223	2.938	0.976	0.000	0.833	100	0.278	-0.006	2.914	0.984	0.000	46	135	43.4	45.6
		$q = 1$	0.015	1.189	0.675	0.006	0.989	71	0.041	-0.003	1.170	0.772	0.002	78	326	3.5	96.5
		$q = 2$	0.002	0.771	0.679	0.005	0.998	72	0.018	-0.001	0.752	0.768	0.002	75	26	2.2	98.7
		$q = 3$	-0.003	0.642	0.694	0.004	1.002	68	0.013	-0.001	0.616	0.754	0.003	72	28	1.9	99.2
	Mongol-Urban	$q = 0$	0.238	2.726	0.977	0.000	0.820	100	0.296	-0.007	2.692	0.985	0.000	46	144	37.1	43.0
		$q = 1$	0.026	0.987	0.700	0.006	0.981	74	0.036	-0.002	0.993	0.775	0.006	72	15	2.9	96.0
		$q = 2$	-0.001	0.645	0.728	0.004	1.000	62	-0.004	0.000	0.644	0.790	0.003	74	16	1.9	101.7
		$q = 3$	-0.007	0.537	0.727	0.005	1.004	65	-0.014	0.001	0.531	0.802	0.003	73	16	1.7	102.6
	Tibetan-Rural	$q = 0$	0.196	2.763	0.961	0.000	0.854	100	0.268	-0.008	2.713	0.975	0.000	75	75	31.5	51.9
		$q = 1$	0.059	1.093	0.763	0.002	0.957	75	0.106	-0.006	1.087	0.807	0.002	66	14	3.6	88.1
		$q = 2$	0.039	0.809	0.697	0.005	0.972	72	0.071	-0.004	0.788	0.794	0.003	76	18	2.5	92.4
		$q = 3$	0.041	0.701	0.698	0.003	0.970	61	0.064	-0.004	0.704	0.778	0.003	75	27	2.3	93.2
	Tibeitan-Urban	$q = 0$	0.223	2.723	0.964	0.000	0.832	100	0.321	-0.016	2.677	0.979	0.000	50	30	28.8	53.1
		$q = 1$	0.061	0.963	0.790	0.005	0.953	60	0.150	-0.019	0.986	0.833	0.005	58	9	3.2	91.7
		$q = 2$	0.029	0.673	0.780	0.006	0.975	51	0.085	-0.013	0.690	0.822	0.007	58	38	2.2	102.2
		$q = 3$	0.009	0.615	0.761	0.007	0.990	57	0.062	-0.012	0.622	0.825	0.006	63	11	2.0	101.2
	Uyghur-Rural	$q = 0$	0.244	2.901	0.979	0.000	0.815	100	0.337	-0.021	2.877	0.991	0.000	52	47	34.7	54.4
		$q = 1$	0.179	1.157	0.862	0.006	0.861	49	0.343	-0.057	1.245	0.928	0.006	48	6	4.8	77.8
		$q = 2$	0.065	0.931	0.832	0.010	0.942	67	0.196	-0.038	0.944	0.914	0.009	57	8	3.1	91.9
		$q = 3$	0.012	0.856	0.834	0.010	0.981	71	0.081	-0.020	0.853	0.913	0.009	62	15	2.5	103.8
	Uyghur-Urban	$q = 0$	0.201	2.981	0.967	0.000	0.850	100	0.299	-0.021	2.963	0.979	0.000	59	48	33.5	60.3
		$q = 1$	0.044	1.117	0.787	0.007	0.968	70	0.071	-0.007	1.124	0.860	0.006	64	10	3.4	92.7
		$q = 2$	0.003	0.720	0.764	0.012	0.996	56	-0.013	0.003	0.730	0.863	0.008	62	8	2.1	102.2
		$q = 3$	-0.009	0.598	0.762	0.014	1.005	52	-0.030	0.005	0.598	0.857	0.009	63	7	1.8	103.3
	Zhuang-Rural	$q = 0$	0.228	2.788	0.963	0.000	0.828	100	0.303	-0.009	2.750	0.977	0.000	46	82	37.0	46.0
		$q = 1$	0.009	1.005	0.664	0.004	0.992	58	0.030	-0.002	0.992	0.769	0.002	70	29	2.9	97.5
		$q = 2$	-0.017	0.649	0.650	0.007	1.010	60	-0.003	-0.001	0.630	0.751	0.004	71	14	1.9	103.9
		$q = 3$	-0.019	0.536	0.643	0.009	1.012	62	-0.013	-0.001	0.529	0.751	0.005	72	20	1.6	107.0
	Zhuang-Urban	$q = 0$	0.252	2.761	0.974	0.000	0.809	100	0.326	-0.010	2.727	0.981	0.000	68	95	37.4	44.5
		$q = 1$	0.091	0.851	0.703	0.006	0.933	64	0.160	-0.014	0.895	0.732	0.007	62	20	3.2	83.9
		$q = 2$	0.025	0.525	0.689	0.007	0.980	57	0.062	-0.007	0.549	0.735	0.006	54	12	1.9	97.9
		$q = 3$	0.012	0.429	0.711	0.004	0.989	53	0.038	-0.004	0.429	0.741	0.006	53	12	1.6	99.0
Family	Total	$q = 0$	0.255	4.524	0.992	0.000	0.806	100	0.291	0.000	4.420	0.996	0.000	89	7279	467.8	20.7
		$q = 1$	0.024	2.813	0.574	0.000	0.983	95	0.052	0.000	2.736	0.687	0.000	92	164	19.1	88.2
		$q = 2$	0.026	2.398	0.504	0.001	0.982	93	0.057	0.000	2.311	0.636	0.000	93	174	12.7	88.0
		$q = 3$	0.028	2.216	0.480	0.001	0.980	86	0.061	0.000	2.132	0.613	0.000	91	162	10.8	88.1
	Rural	$q = 0$	0.268	4.494	0.989	0.000	0.795	100	0.316	-0.001	4.381	0.993	0.000	88	499	379.9	24.0
		$q = 1$	0.042	2.750	0.651	0.001	0.970	95	0.084	-0.001	2.657	0.747	0.000	89	147	19.2	82.5
		$q = 2$	0.053	2.288	0.611	0.001	0.962	90	0.107	-0.001	2.172	0.720	0.001	83	123	12.8	79.3
		$q = 3$	0.062	2.072	0.595	0.001	0.956	85	0.118	-0.001	1.959	0.705	0.001	80	256	10.8	77.8

Urban	$q = 0$	0.280	4.394	0.993	0.000	0.786	100	0.326	-0.001	4.290	0.996	0.000	91	764	361.8	22.9
	$q = 1$	0.050	2.699	0.721	0.000	0.965	96	0.097	-0.001	2.598	0.813	0.000	89	105	18.8	79.9
	$q = 2$	0.057	2.264	0.661	0.000	0.959	93	0.114	-0.001	2.141	0.734	0.001	87	88	12.6	78.1
	$q = 3$	0.062	2.080	0.631	0.001	0.955	92	0.123	-0.002	1.953	0.718	0.000	88	92	10.8	77.0
Bai	$q = 0$	0.325	4.271	0.981	0.000	0.747	100	0.397	-0.006	4.216	0.985	0.000	49	748	310.0	27.0
	$q = 1$	0.079	2.565	0.763	0.002	0.943	92	0.153	-0.006	2.497	0.846	0.000	82	81	17.4	77.0
	$q = 2$	0.101	2.095	0.694	0.001	0.927	85	0.196	-0.008	2.024	0.789	0.001	78	25	11.7	75.1
	$q = 3$	0.106	1.894	0.653	0.002	0.922	79	0.195	-0.008	1.854	0.753	0.001	81	39	10.0	76.3
Han	$q = 0$	0.276	4.439	0.991	0.000	0.789	100	0.328	-0.002	4.335	0.995	0.000	80	435	335.7	25.5
	$q = 1$	0.048	2.623	0.725	0.001	0.966	93	0.088	-0.002	2.561	0.808	0.001	85	69	17.0	82.8
	$q = 2$	0.053	2.166	0.649	0.000	0.962	90	0.094	-0.002	2.110	0.748	0.000	83	60	11.0	83.0
	$q = 3$	0.057	1.981	0.602	0.001	0.959	89	0.098	-0.002	1.929	0.716	0.001	81	76	9.3	82.7
Kazakh	$q = 0$	0.313	4.364	0.983	0.000	0.757	100	0.415	-0.012	4.285	0.987	0.000	52	113	238.6	34.8
	$q = 1$	0.135	2.615	0.755	0.004	0.899	96	0.307	-0.023	2.507	0.880	0.001	75	21	20.6	69.6
	$q = 2$	0.205	2.024	0.701	0.005	0.841	80	0.364	-0.025	1.997	0.831	0.002	77	28	13.6	67.3
	$q = 3$	0.239	1.709	0.690	0.008	0.811	65	0.374	-0.027	1.790	0.811	0.004	75	13	11.3	68.7
Mongol	$q = 0$	0.285	4.504	0.982	0.000	0.780	100	0.384	-0.005	4.348	0.989	0.000	66	131	287.8	30.6
	$q = 1$	0.045	2.776	0.654	0.002	0.967	81	0.099	-0.004	2.710	0.760	0.001	79	32	18.9	87.2
	$q = 2$	0.047	2.318	0.607	0.004	0.965	78	0.110	-0.005	2.256	0.731	0.001	79	26	12.2	88.3
	$q = 3$	0.049	2.107	0.602	0.005	0.963	70	0.109	-0.005	2.059	0.716	0.002	81	24	10.0	89.7
Tibetan	$q = 0$	0.297	4.310	0.990	0.000	0.771	100	0.359	-0.004	4.227	0.993	0.000	73	200	264.3	28.7
	$q = 1$	0.101	2.526	0.730	0.001	0.926	83	0.195	-0.008	2.451	0.818	0.001	85	46	18.1	74.1
	$q = 2$	0.121	2.021	0.671	0.001	0.911	79	0.225	-0.010	1.970	0.765	0.001	84	26	11.7	74.7
	$q = 3$	0.126	1.800	0.627	0.004	0.906	78	0.224	-0.010	1.769	0.750	0.001	83	66	9.7	75.2
Uyghur	$q = 0$	0.276	4.503	0.989	0.000	0.789	100	0.351	-0.009	4.441	0.993	0.000	59	1488	254.2	38.0
	$q = 1$	0.088	2.812	0.818	0.002	0.936	85	0.178	-0.013	2.769	0.901	0.001	78	16	21.3	80.0
	$q = 2$	0.124	2.364	0.780	0.002	0.909	85	0.252	-0.019	2.297	0.880	0.001	81	14	15.0	73.7
	$q = 3$	0.141	2.168	0.753	0.003	0.895	85	0.290	-0.021	2.087	0.866	0.001	80	14	12.9	70.9
Zhuang	$q = 0$	0.287	4.324	0.988	0.000	0.780	100	0.353	-0.004	4.237	0.993	0.000	82	445	264.0	30.6
	$q = 1$	0.084	2.605	0.760	0.001	0.939	94	0.179	-0.007	2.504	0.854	0.000	82	103	18.7	75.1
	$q = 2$	0.102	2.134	0.732	0.001	0.926	92	0.210	-0.008	2.019	0.819	0.000	85	85	12.5	71.9
	$q = 3$	0.115	1.925	0.735	0.001	0.915	89	0.227	-0.009	1.813	0.808	0.001	81	76	10.7	69.5
Bai-Rural	$q = 0$	0.321	4.315	0.972	0.000	0.750	100	0.447	-0.013	4.226	0.980	0.000	45	74	235.8	33.4
	$q = 1$	0.085	2.560	0.731	0.002	0.938	86	0.150	-0.008	2.526	0.809	0.001	76	33	16.9	80.2
	$q = 2$	0.093	2.092	0.665	0.004	0.931	67	0.160	-0.008	2.066	0.748	0.003	67	21	11.0	81.1
	$q = 3$	0.077	1.920	0.644	0.004	0.941	61	0.138	-0.008	1.897	0.715	0.004	64	17	8.7	86.3
Bai-Urban	$q = 0$	0.297	4.267	0.985	0.000	0.771	100	0.377	-0.016	4.267	0.992	0.000	53	40	172.7	43.6
	$q = 1$	0.112	2.534	0.889	0.001	0.919	92	0.208	-0.019	2.507	0.934	0.001	78	13	16.5	77.3
	$q = 2$	0.165	2.074	0.880	0.000	0.877	92	0.336	-0.031	1.998	0.934	0.001	73	13	11.9	66.7
	$q = 3$	0.199	1.877	0.874	0.000	0.850	92	0.383	-0.034	1.805	0.928	0.001	75	26	11.0	61.7
Han-Rural	$q = 0$	0.298	4.398	0.989	0.000	0.770	100	0.359	-0.004	4.321	0.993	0.000	83	227	291.7	29.1
	$q = 1$	0.081	2.584	0.755	0.001	0.942	85	0.146	-0.005	2.523	0.822	0.001	78	46	17.7	78.2
	$q = 2$	0.095	2.111	0.679	0.004	0.930	81	0.157	-0.005	2.070	0.758	0.002	76	36	11.5	77.6
	$q = 3$	0.104	1.920	0.639	0.005	0.923	81	0.155	-0.005	1.891	0.736	0.002	76	29	9.7	77.3
Han-Urban	$q = 0$	0.299	4.371	0.988	0.000	0.769	100	0.364	-0.004	4.285	0.992	0.000	71	167	282.8	28.6
	$q = 1$	0.058	2.572	0.738	0.002	0.959	87	0.110	-0.004	2.520	0.793	0.001	79	51	16.3	82.9
	$q = 2$	0.072	2.080	0.676	0.002	0.947	79	0.129	-0.005	2.031	0.732	0.001	76	29	10.4	81.6
	$q = 3$	0.080	1.883	0.652	0.002	0.941	78	0.136	-0.005	1.839	0.728	0.002	75	188	9.0	79.8
Kazakh-Rural	$q = 0$	0.278	4.391	0.969	0.000	0.786	100	0.458	-0.045	4.368	0.983	0.000	59	19	154.3	52.9
	$q = 1$	0.152	2.550	0.830	0.012	0.870	43	0.378	-0.064	2.559	0.904	0.010	56	6	17.5	81.5
	$q = 2$	0.158	2.024	0.852	0.007	0.841	32	0.449	-0.083	2.032	0.899	0.012	49	18	11.3	86.6
	$q = 3$	0.111	1.845	0.859	0.007	0.872	29	0.414	-0.082	1.806	0.895	0.013	47	25	9.6	86.0
Kazakh-Urban	$q = 0$	0.358	4.322	0.986	0.000	0.717	100	0.478	-0.020	4.260	0.990	0.000	46	68	239.3	34.2
	$q = 1$	0.180	2.633	0.864	0.001	0.863	96	0.374	-0.038	2.560	0.926	0.002	74	13	21.0	68.3

		q = 2	0.253	2.145	0.808	0.006	0.797	87	0.490	-0.045	2.039	0.900	0.004	70	13	15.0	61.5
		q = 3	0.304	1.891	0.803	0.005	0.753	75	0.486	-0.038	1.830	0.892	0.004	69	33	17.4	60.9
		q = 0	0.289	4.623	0.989	0.000	0.777	100	0.361	-0.007	4.549	0.993	0.000	65	120	309.6	34.1
	Mongol-Rural	q = 1	0.052	2.851	0.670	0.005	0.961	59	0.063	-0.003	2.882	0.765	0.004	77	11	20.2	92.7
		q = 2	0.044	2.408	0.659	0.005	0.965	65	0.070	-0.004	2.411	0.767	0.002	76	12	12.8	94.8
		q = 3	0.031	2.218	0.662	0.004	0.973	66	0.065	-0.004	2.195	0.772	0.002	75	15	10.3	97.1
		q = 0	0.306	4.163	0.992	0.000	0.763	100	0.371	-0.008	4.124	0.995	0.000	56	144	195.7	34.1
	Mongol-Urban	q = 1	0.095	2.499	0.784	0.002	0.930	81	0.180	-0.013	2.468	0.850	0.003	72	26	16.2	78.8
		q = 2	0.104	2.081	0.709	0.006	0.923	73	0.191	-0.014	2.069	0.810	0.004	71	56	10.9	80.6
		q = 3	0.129	1.857	0.711	0.005	0.903	61	0.196	-0.013	1.885	0.791	0.004	68	14	9.3	79.8
		q = 0	0.304	4.276	0.989	0.000	0.765	100	0.384	-0.008	4.199	0.993	0.000	67	101	219.9	33.1
	Tibetan-Rural	q = 1	0.130	2.426	0.770	0.002	0.904	80	0.224	-0.012	2.389	0.843	0.002	80	30	17.2	71.9
		q = 2	0.144	1.900	0.704	0.004	0.891	74	0.273	-0.018	1.880	0.807	0.002	75	23	10.9	72.9
		q = 3	0.144	1.664	0.683	0.005	0.891	70	0.278	-0.019	1.668	0.792	0.002	74	29	9.0	74.6
		q = 0	0.296	4.363	0.985	0.000	0.771	100	0.376	-0.013	4.324	0.990	0.000	55	177	226.9	38.9
	Tibeitan-Urban	q = 1	0.167	2.447	0.824	0.003	0.874	77	0.252	-0.019	2.463	0.878	0.003	75	19	17.6	73.5
		q = 2	0.218	1.958	0.800	0.004	0.830	74	0.391	-0.035	1.937	0.879	0.002	69	11	12.1	66.4
		q = 3	0.242	1.754	0.792	0.003	0.810	72	0.431	-0.038	1.728	0.866	0.003	69	10	10.4	64.4
		q = 0	0.295	4.409	0.987	0.000	0.772	100	0.407	-0.026	4.390	0.994	0.000	50	21	171.2	48.1
	Uyghur-Rural	q = 1	0.168	2.735	0.869	0.006	0.875	76	0.336	-0.046	2.735	0.945	0.004	65	11	21.6	73.8
		q = 2	0.242	2.261	0.849	0.008	0.813	76	0.487	-0.068	2.256	0.934	0.006	68	7	15.2	66.7
		q = 3	0.299	2.006	0.845	0.009	0.764	69	0.625	-0.089	1.991	0.940	0.004	62	7	13.3	59.8
		q = 0	0.287	4.500	0.988	0.000	0.779	100	0.379	-0.017	4.459	0.993	0.000	59	38	206.1	43.6
	Uyghur-Urban	q = 1	0.102	2.774	0.894	0.001	0.926	96	0.215	-0.024	2.742	0.951	0.000	85	12	20.2	79.0
		q = 2	0.139	2.327	0.852	0.003	0.898	93	0.289	-0.033	2.294	0.924	0.002	87	60	14.2	73.8
		q = 3	0.171	2.111	0.851	0.003	0.872	84	0.356	-0.041	2.076	0.926	0.002	76	12	12.3	70.1
		q = 0	0.297	4.331	0.986	0.000	0.771	100	0.379	-0.010	4.269	0.992	0.000	84	123	214.8	36.8
	Zhuang-Rural	q = 1	0.090	2.581	0.766	0.003	0.935	88	0.186	-0.013	2.528	0.866	0.001	83	18	17.2	79.3
		q = 2	0.125	2.090	0.797	0.001	0.908	84	0.235	-0.015	2.037	0.868	0.001	79	19	11.6	73.8
		q = 3	0.143	1.889	0.802	0.002	0.894	87	0.253	-0.014	1.823	0.867	0.001	77	20	10.0	69.7
		q = 0	0.310	4.258	0.989	0.000	0.760	100	0.386	-0.009	4.192	0.992	0.000	57	266	244.9	31.5
	Zhuang-Urban	q = 1	0.126	2.543	0.805	0.001	0.907	92	0.215	-0.013	2.516	0.875	0.001	77	24	18.6	72.7
		q = 2	0.149	2.037	0.749	0.003	0.889	82	0.236	-0.015	2.053	0.824	0.002	80	65	12.5	72.4
		q = 3	0.162	1.822	0.720	0.005	0.878	78	0.234	-0.014	1.874	0.809	0.003	80	15	10.0	73.7
Genus		q = 0	0.288	5.137	0.994	0.000	0.779	100	0.328	0.000	5.020	0.997	0.000	94	1093	984.5	17.6
	Total	q = 1	0.040	3.357	0.657	0.000	0.972	100	0.087	-0.001	3.223	0.791	0.000	96	183	35.8	80.9
		q = 2	0.045	2.831	0.631	0.000	0.968	99	0.101	-0.001	2.672	0.771	0.000	97	201	21.7	78.9
		q = 3	0.050	2.619	0.632	0.000	0.965	99	0.111	-0.001	2.446	0.772	0.000	98	215	18.1	76.9
		q = 0	0.296	5.125	0.992	0.000	0.772	100	0.341	-0.001	5.017	0.995	0.000	86	13123	947.7	19.4
	Rural	q = 1	0.051	3.324	0.712	0.000	0.964	97	0.101	-0.001	3.210	0.809	0.000	91	150	35.6	78.8
		q = 2	0.058	2.788	0.677	0.000	0.959	98	0.120	-0.001	2.648	0.779	0.000	89	1121	21.6	76.8
		q = 3	0.068	2.561	0.687	0.000	0.951	98	0.136	-0.002	2.410	0.783	0.000	90	566	18.1	73.2
		q = 0	0.309	5.023	0.994	0.000	0.761	100	0.353	-0.001	4.923	0.996	0.000	84	659	829.8	18.7
	Urban	q = 1	0.060	3.274	0.745	0.000	0.957	98	0.120	-0.002	3.148	0.851	0.000	96	117	35.1	76.3
		q = 2	0.067	2.743	0.712	0.001	0.952	95	0.142	-0.002	2.588	0.824	0.000	95	87	21.4	74.4
		q = 3	0.071	2.530	0.690	0.001	0.949	95	0.151	-0.002	2.365	0.810	0.000	96	89	17.6	73.4
		q = 0	0.340	4.927	0.986	0.000	0.733	100	0.420	-0.006	4.841	0.989	0.000	59	140	553.1	26.4
	Bai	q = 1	0.110	3.106	0.809	0.000	0.920	95	0.206	-0.008	3.007	0.872	0.000	88	48	32.9	70.2
		q = 2	0.134	2.545	0.808	0.000	0.901	95	0.260	-0.009	2.399	0.877	0.000	86	45	20.4	64.1
		q = 3	0.154	2.296	0.824	0.000	0.886	94	0.283	-0.010	2.147	0.885	0.000	85	73	17.0	60.5
		q = 0	0.312	5.048	0.992	0.000	0.758	100	0.364	-0.002	4.950	0.995	0.000	86	721	735.7	21.9
	Han	q = 1	0.068	3.213	0.788	0.000	0.951	98	0.138	-0.003	3.092	0.883	0.000	94	62	33.2	75.9
		q = 2	0.079	2.643	0.768	0.001	0.943	97	0.164	-0.003	2.497	0.858	0.001	91	57	19.7	72.9
		q = 3	0.087	2.409	0.763	0.000	0.937	96	0.179	-0.004	2.249	0.856	0.000	90	63	16.1	70.5

Kazakh	$q = 0$	0.351	4.966	0.986	0.000	0.724	100	0.433	-0.010	4.917	0.989	0.000	60	366	548.6	29.9
	$q = 1$	0.168	3.141	0.889	0.000	0.874	100	0.322	-0.020	3.043	0.936	0.000	86	28	37.8	63.0
	$q = 2$	0.219	2.587	0.873	0.000	0.832	100	0.441	-0.029	2.429	0.929	0.000	81	30	25.6	54.1
	$q = 3$	0.249	2.351	0.871	0.000	0.807	100	0.500	-0.032	2.171	0.928	0.000	75	24	21.9	50.2
Mongol	$q = 0$	0.337	5.073	0.984	0.000	0.736	100	0.426	-0.005	4.939	0.988	0.000	68	524	732.4	24.1
	$q = 1$	0.076	3.249	0.710	0.002	0.945	84	0.139	-0.005	3.187	0.801	0.000	84	47	33.9	79.3
	$q = 2$	0.083	2.695	0.705	0.001	0.939	84	0.166	-0.006	2.607	0.801	0.000	76	31	20.1	77.2
	$q = 3$	0.091	2.465	0.693	0.001	0.934	85	0.166	-0.006	2.397	0.773	0.002	82	33	16.3	77.1
Tibetan	$q = 0$	0.316	4.959	0.988	0.000	0.755	100	0.389	-0.005	4.873	0.994	0.000	80	485	553.8	28.1
	$q = 1$	0.118	2.955	0.712	0.002	0.913	88	0.213	-0.008	2.871	0.808	0.000	82	37	29.2	70.7
	$q = 2$	0.133	2.232	0.632	0.004	0.899	66	0.213	-0.009	2.223	0.728	0.002	74	371	15.5	75.1
	$q = 3$	0.101	2.022	0.595	0.006	0.921	67	0.180	-0.009	2.023	0.710	0.004	70	25	11.4	83.8
Uyghur	$q = 0$	0.317	5.124	0.993	0.000	0.754	100	0.386	-0.008	5.062	0.995	0.000	61	136	563.6	30.8
	$q = 1$	0.112	3.308	0.842	0.001	0.918	96	0.229	-0.016	3.238	0.911	0.001	85	25	37.6	73.7
	$q = 2$	0.140	2.749	0.825	0.001	0.897	95	0.320	-0.024	2.635	0.905	0.001	80	22	23.5	67.4
	$q = 3$	0.155	2.523	0.817	0.001	0.885	96	0.378	-0.030	2.371	0.912	0.000	77	19	19.5	63.7
Zhuang	$q = 0$	0.300	4.977	0.990	0.000	0.768	100	0.362	-0.004	4.898	0.993	0.000	88	203	507.3	29.2
	$q = 1$	0.097	3.153	0.768	0.000	0.930	99	0.191	-0.007	3.049	0.870	0.000	86	91	33.4	71.8
	$q = 2$	0.112	2.578	0.698	0.002	0.918	93	0.229	-0.009	2.465	0.829	0.001	80	37	20.0	70.1
	$q = 3$	0.122	2.334	0.676	0.002	0.910	88	0.235	-0.009	2.236	0.808	0.000	82	33	16.1	70.1
Bai-Rural	$q = 0$	0.333	4.962	0.978	0.000	0.739	100	0.446	-0.011	4.860	0.986	0.000	69	89	484.5	31.2
	$q = 1$	0.121	3.137	0.799	0.001	0.911	93	0.234	-0.013	3.057	0.878	0.000	84	31	34.1	70.4
	$q = 2$	0.138	2.599	0.806	0.001	0.898	93	0.273	-0.015	2.504	0.877	0.000	84	47	21.1	67.0
	$q = 3$	0.150	2.373	0.809	0.001	0.889	94	0.298	-0.016	2.256	0.884	0.000	80	29	17.3	64.2
Bai-Urban	$q = 0$	0.343	4.891	0.991	0.000	0.731	100	0.426	-0.015	4.862	0.994	0.000	61	105	418.9	34.3
	$q = 1$	0.139	3.004	0.863	0.002	0.898	86	0.203	-0.014	2.998	0.909	0.003	87	102	28.0	75.2
	$q = 2$	0.196	2.368	0.826	0.004	0.851	79	0.277	-0.019	2.367	0.881	0.004	76	12	16.5	70.6
	$q = 3$	0.225	2.099	0.815	0.004	0.826	78	0.297	-0.017	2.099	0.878	0.004	74	11	13.6	68.3
Han-Rural	$q = 0$	0.331	5.021	0.990	0.000	0.741	100	0.404	-0.005	4.926	0.993	0.000	74	304	638.7	25.6
	$q = 1$	0.105	3.163	0.830	0.000	0.924	99	0.203	-0.007	3.049	0.897	0.000	87	58	34.5	69.4
	$q = 2$	0.123	2.575	0.809	0.000	0.910	100	0.242	-0.009	2.438	0.884	0.000	88	48	20.5	65.6
	$q = 3$	0.136	2.330	0.802	0.000	0.900	100	0.259	-0.009	2.189	0.876	0.000	88	63	16.9	62.9
Han-Urban	$q = 0$	0.325	4.994	0.989	0.000	0.747	100	0.392	-0.004	4.896	0.992	0.000	78	909	668.6	24.6
	$q = 1$	0.084	3.141	0.776	0.001	0.939	97	0.174	-0.006	3.032	0.864	0.001	93	51	31.3	74.6
	$q = 2$	0.096	2.581	0.732	0.001	0.930	97	0.216	-0.008	2.432	0.833	0.001	89	37	18.8	71.1
	$q = 3$	0.104	2.353	0.728	0.001	0.924	96	0.231	-0.009	2.196	0.829	0.001	88	35	15.4	69.4
Kazakh-Rural	$q = 0$	0.322	5.016	0.973	0.000	0.747	100	0.488	-0.040	4.987	0.985	0.000	60	23	325.6	48.1
	$q = 1$	0.207	3.091	0.870	0.006	0.839	87	0.353	-0.040	3.094	0.935	0.005	71	9	32.8	72.1
	$q = 2$	0.269	2.531	0.852	0.008	0.782	80	0.432	-0.045	2.526	0.925	0.007	67	7	20.8	67.6
	$q = 3$	0.300	2.300	0.849	0.008	0.754	77	0.426	-0.037	2.308	0.916	0.009	69	16	17.9	64.8
Kazakh-Urban	$q = 0$	0.354	5.002	0.984	0.000	0.721	100	0.498	-0.023	4.913	0.988	0.000	52	58	450.9	34.2
	$q = 1$	0.215	3.110	0.935	0.000	0.836	100	0.415	-0.038	3.029	0.974	0.000	78	15	37.6	59.7
	$q = 2$	0.282	2.527	0.921	0.000	0.778	100	0.564	-0.054	2.419	0.966	0.000	77	18	24.4	52.2
	$q = 3$	0.317	2.278	0.913	0.000	0.747	100	0.613	-0.057	2.165	0.960	0.000	77	17	20.7	48.9
Mongol-Rural	$q = 0$	0.309	5.297	0.990	0.000	0.760	100	0.375	-0.007	5.238	0.992	0.000	60	193	677.3	30.6
	$q = 1$	0.092	3.303	0.697	0.005	0.932	68	0.165	-0.010	3.274	0.804	0.004	66	15	35.9	81.5
	$q = 2$	0.118	2.690	0.700	0.005	0.911	71	0.205	-0.012	2.665	0.787	0.005	74	16	21.1	78.6
	$q = 3$	0.146	2.401	0.720	0.003	0.889	69	0.225	-0.012	2.393	0.790	0.004	73	25	17.0	75.1
Mongol-Urban	$q = 0$	0.328	4.781	0.993	0.000	0.744	100	0.387	-0.007	4.744	0.996	0.000	63	143	411.9	30.8
	$q = 1$	0.127	2.994	0.852	0.001	0.907	95	0.231	-0.015	2.940	0.899	0.001	79	24	29.1	71.0
	$q = 2$	0.143	2.465	0.809	0.001	0.895	92	0.265	-0.017	2.403	0.880	0.001	87	25	17.9	69.3
	$q = 3$	0.156	2.241	0.785	0.002	0.884	90	0.297	-0.020	2.177	0.859	0.003	85	20	14.8	68.3
Tibetan-Rural	$q = 0$	0.338	4.855	0.987	0.000	0.735	100	0.446	-0.010	4.743	0.992	0.000	71	77	429.2	29.6
	$q = 1$	0.167	2.780	0.772	0.003	0.874	89	0.330	-0.019	2.683	0.849	0.001	81	20	27.5	64.8

	$q = 2$	0.176	2.020	0.716	0.003	0.864	77	0.340	-0.022	1.972	0.816	0.001	77	18	13.7	69.3
	$q = 3$	0.136	1.785	0.690	0.004	0.893	76	0.297	-0.022	1.763	0.792	0.002	76	15	10.1	77.7
	$q = 0$	0.357	4.952	0.984	0.000	0.718	100	0.469	-0.020	4.915	0.990	0.000	67	125	440.1	35.0
Tibeitan-Urban	$q = 1$	0.231	2.808	0.812	0.004	0.823	77	0.387	-0.034	2.807	0.887	0.003	68	20	29.6	63.7
	$q = 2$	0.305	2.045	0.792	0.005	0.756	64	0.469	-0.042	2.126	0.858	0.006	66	13	16.8	62.7
	$q = 3$	0.298	1.797	0.781	0.005	0.757	64	0.480	-0.044	1.868	0.862	0.004	59	10	13.1	64.6
	$q = 0$	0.320	5.060	0.994	0.000	0.751	100	0.413	-0.020	5.022	0.997	0.000	53	72	406.8	40.1
Uyghur-Rural	$q = 1$	0.210	3.181	0.894	0.003	0.842	77	0.381	-0.050	3.197	0.945	0.004	70	8	36.6	69.7
	$q = 2$	0.275	2.607	0.883	0.004	0.785	85	0.534	-0.071	2.599	0.948	0.004	72	22	24.0	60.8
	$q = 3$	0.318	2.353	0.889	0.003	0.747	89	0.567	-0.071	2.364	0.947	0.004	77	9	19.8	58.6
	$q = 0$	0.329	5.146	0.989	0.000	0.743	100	0.435	-0.021	5.109	0.995	0.000	68	53	446.6	39.8
Uyghur-Urban	$q = 1$	0.139	3.255	0.899	0.001	0.898	91	0.275	-0.030	3.229	0.944	0.001	85	13	35.8	73.8
	$q = 2$	0.172	2.675	0.860	0.003	0.872	89	0.356	-0.042	2.653	0.924	0.002	86	16	21.6	70.5
	$q = 3$	0.190	2.432	0.837	0.004	0.857	89	0.402	-0.049	2.408	0.910	0.003	88	24	17.7	68.2
	$q = 0$	0.328	4.952	0.987	0.000	0.744	100	0.421	-0.011	4.876	0.993	0.000	86	107	443.7	32.8
Zhuang-Rural	$q = 1$	0.121	3.035	0.802	0.001	0.912	88	0.248	-0.017	2.963	0.875	0.001	75	17	29.9	72.9
	$q = 2$	0.154	2.424	0.750	0.002	0.885	80	0.280	-0.018	2.390	0.821	0.003	78	18	17.9	71.1
	$q = 3$	0.179	2.154	0.746	0.002	0.865	75	0.302	-0.019	2.140	0.814	0.002	72	54	15.0	67.9
	$q = 0$	0.313	4.906	0.989	0.000	0.757	100	0.400	-0.010	4.837	0.992	0.000	61	1018	492.2	33.4
Zhuang-Urban	$q = 1$	0.151	3.117	0.905	0.000	0.889	100	0.271	-0.016	3.040	0.947	0.000	76	33	35.9	64.0
	$q = 2$	0.167	2.541	0.842	0.001	0.875	100	0.316	-0.019	2.446	0.905	0.000	81	34	21.0	62.3
	$q = 3$	0.179	2.295	0.810	0.001	0.866	98	0.337	-0.021	2.200	0.884	0.001	85	41	17.1	61.1

Table S2. The *p*-value of the permutation tests for the differences in the parameters of the DAR models for all pair-wise comparisons of Chinese gut microbiome datasets of four Schemes (1A-2B)

Scheme-1A

Diversity order	Taxon	Cohort	PL		PLEC					
			z	ln(c)	z	d	ln(c)	A_{max}	D_{max}	LGD
$q = 0$	Phylum	Rural *vs.* Urban	0.480	0.620	0.657	0.846	0.685	0.727	0.726	0.673
	Family		0.749	0.540	0.897	0.927	0.691	0.622	0.787	0.849
	Genus		0.727	0.567	0.872	0.940	0.683	0.034	0.488	0.900
	Species		0.964	0.600	0.961	0.861	0.698	0.390	0.371	0.860
	(%) With significant differences		0	0	0	0	0	25% (1/4)	0	0
$q = 1$	Phylum	Rural *vs.* Urban	0.970	0.805	0.964	0.972	0.871	0.160	0.342	0.919
	Family		0.844	0.773	0.853	0.838	0.813	0.532	0.699	0.856
	Genus		0.814	0.778	0.815	0.720	0.816	0.517	0.774	0.864
	Species		0.953	0.893	0.950	0.942	0.977	0.891	0.568	0.990
	(%) With significant differences		0	0	0	0	0	0	0	0
$q = 2$	Phylum	Rural *vs.* Urban	0.967	0.774	0.975	0.979	0.814	0.651	0.270	0.920
	Family		0.939	0.916	0.954	0.901	0.935	0.610	0.796	0.942
	Genus		0.860	0.840	0.843	0.733	0.841	0.024	0.850	0.896
	Species		0.993	0.918	0.985	0.897	0.932	0.417	0.622	0.959
	(%) With significant differences		0	0	0	0	0	25% (1/4)	0	0
$q = 3$	Phylum	Rural *vs.* Urban	0.976	0.763	0.996	0.991	0.789	0.198	0.298	0.981
	Family		0.999	0.978	0.969	0.935	0.992	0.218	0.969	0.971
	Genus		0.960	0.893	0.886	0.757	0.893	0.055	0.599	0.990
	Species		0.984	0.893	0.983	0.909	0.868	0.856	0.647	0.969
	(%) With significant differences		0	0	0	0	0	0	0	0

Scheme-1B

Diversity order	Taxon	Cohort	PL		PLEC					
			z	ln(c)	z	d	ln(c)	A_{max}	D_{max}	LGD
$q = 0$	Phylum	Bai-Rural *vs.* Bai-Urban	0.868	0.909	0.975	0.894	0.913	0.597	0.983	0.913
		Han-Rural *vs.* Han-Urban	0.712	0.618	0.902	0.830	0.664	0.262	0.558	0.509
		Kazakh-Rural *vs.* Kazakh-Urban	0.667	0.689	0.893	0.598	0.620	0.134	0.546	0.567
		Mongol-Rural *vs.* Mongol-Urban	0.846	0.303	0.907	0.948	0.356	0.901	0.454	0.871
		Tibetan-Rural *vs.* Tibetan-Urban	0.739	0.839	0.738	0.739	0.868	0.581	0.786	0.954
		Uyghur-Rural *vs.* Uyghur-Urban	0.657	0.672	0.852	1.000	0.657	0.970	0.826	0.715
		Zhuang-Rural *vs.* Zhuang-Urban	0.715	0.880	0.886	0.919	0.909	0.861	0.961	0.920
	(%) With significant differences		0	0	0	0	0	0	0	0
	Family	Bai-Rural *vs.* Bai-Urban	0.818	0.854	0.755	0.915	0.859	0.735	0.440	0.581
		Han-Rural *vs.* Han-Urban	0.979	0.910	0.973	0.974	0.898	0.677	0.829	0.935
		Kazakh-Rural *vs.* Kazakh-Urban	0.470	0.800	0.940	0.604	0.696	0.324	0.198	0.267
		Mongol-Rural *vs.* Mongol-Urban	0.870	0.121	0.958	0.945	0.223	0.713	0.114	0.993
		Tibetan-Rural *vs.* Tibetan-Urban	0.902	0.689	0.973	0.794	0.621	0.485	0.933	0.625
		Uyghur-Rural *vs.* Uyghur-Urban	0.929	0.636	0.873	0.817	0.735	0.724	0.492	0.781
		Zhuang-Rural *vs.* Zhuang-Urban	0.852	0.712	0.966	0.911	0.717	0.285	0.455	0.623
	(%) With significant differences		0	0	0	0	0	0	0	0
	Genus	Bai-Rural *vs.* Bai-Urban	0.908	0.789	0.931	0.905	0.996	0.873	0.759	0.847
		Han-Rural *vs.* Han-Urban	0.915	0.910	0.920	0.891	0.916	0.139	0.750	0.916
		Kazakh-Rural *vs.* Kazakh-Urban	0.807	0.962	0.961	0.745	0.806	0.426	0.297	0.404
		Mongol-Rural *vs.* Mongol-Urban	0.858	0.119	0.951	0.968	0.206	0.533	0.134	0.990
		Tibetan-Rural *vs.* Tibetan-Urban	0.844	0.699	0.907	0.719	0.543	0.622	0.962	0.689
		Uyghur-Rural *vs.* Uyghur-Urban	0.909	0.644	0.901	0.982	0.654	0.721	0.707	0.980
		Zhuang-Rural *vs.* Zhuang-Urban	0.832	0.818	0.904	0.952	0.864	0.041	0.539	0.931
	(%) With significant differences		0	0	0	0	0	14.3% (1/7)	0	0
	Species	Bai-Rural *vs.* Bai-Urban	0.360	0.567	0.464	0.460	0.750	0.384	0.584	0.798

		Han-Rural *vs.* Han-Urban	0.749	0.984	0.912	0.930	0.933	0.118	0.418	0.940
		Kazakh-Rural *vs.* Kazakh-Urban	0.942	0.998	0.796	0.660	0.945	0.716	0.515	0.750
		Mongol-Rural *vs.* Mongol-Urban	0.826	0.102	0.989	0.966	0.226	0.095	0.245	0.785
		Tibetan-Rural *vs.* Tibetan-Urban	0.349	0.580	0.404	0.758	0.366	0.670	0.192	0.040
		Uyghur-Rural *vs.* Uyghur-Urban	0.968	0.553	0.958	0.998	0.601	0.689	0.688	0.978
		Zhuang-Rural *vs.* Zhuang-Urban	0.982	0.602	0.874	0.941	0.806	0.100	0.885	0.994
	(%) With significant differences		0	0	0	0	0	0	0	14.3% (1/7)
		Bai-Rural *vs.* Bai-Urban	0.865	0.955	0.995	0.904	0.836	0.561	0.951	0.996
		Han-Rural *vs.* Han-Urban	0.878	0.704	0.921	0.891	0.700	0.825	0.274	0.971
		Kazakh-Rural *vs.* Kazakh-Urban	0.654	0.886	0.887	0.975	0.723	0.408	0.258	0.805
	Phylum	Mongol-Rural *vs.* Mongol-Urban	0.921	0.458	0.982	0.949	0.571	0.027	0.129	0.980
		Tibetan-Rural *vs.* Tibetan-Urban	0.989	0.713	0.854	0.711	0.771	0.747	0.608	0.886
		Uyghur-Rural *vs.* Uyghur-Urban	0.404	0.904	0.456	0.497	0.749	0.583	0.109	0.609
		Zhuang-Rural *vs.* Zhuang-Urban	0.453	0.596	0.588	0.661	0.743	0.506	0.613	0.582
	(%) With significant differences		0	0	0	0	0	14.3% (1/7)	0	0
		Bai-Rural *vs.* Bai-Urban	0.781	0.939	0.788	0.715	0.943	0.388	0.873	0.904
		Han-Rural *vs.* Han-Urban	0.730	0.955	0.794	0.843	0.989	0.790	0.285	0.826
		Kazakh-Rural *vs.* Kazakh-Urban	0.870	0.781	0.995	0.729	0.999	0.458	0.353	0.664
	Family	Mongol-Rural *vs.* Mongol-Urban	0.731	0.339	0.646	0.712	0.324	0.365	0.123	0.640
		Tibetan-Rural *vs.* Tibetan-Urban	0.781	0.957	0.909	0.841	0.859	0.514	0.889	0.955
		Uyghur-Rural *vs.* Uyghur-Urban	0.554	0.854	0.669	0.661	0.979	0.822	0.503	0.811
		Zhuang-Rural *vs.* Zhuang-Urban	0.674	0.872	0.877	0.999	0.967	0.722	0.658	0.780
$q = 1$	(%) With significant differences		0	0	0	0	0	0	0	0
		Bai-Rural *vs.* Bai-Urban	0.893	0.666	0.922	0.978	0.868	0.113	0.221	0.856
		Han-Rural *vs.* Han-Urban	0.742	0.918	0.836	0.885	0.958	0.690	0.130	0.753
		Kazakh-Rural *vs.* Kazakh-Urban	0.973	0.955	0.816	0.962	0.854	0.605	0.307	0.642
	Genus	Mongol-Rural *vs.* Mongol-Urban	0.781	0.412	0.794	0.848	0.467	0.546	0.129	0.730
		Tibetan-Rural *vs.* Tibetan-Urban	0.734	0.967	0.895	0.769	0.825	0.972	0.730	0.978
		Uyghur-Rural *vs.* Uyghur-Urban	0.605	0.815	0.738	0.721	0.925	0.611	0.820	0.903
		Zhuang-Rural *vs.* Zhuang-Urban	0.741	0.741	0.914	0.959	0.766	0.453	0.183	0.683
	(%) With significant differences		0	0	0	0	0	0	0	0
		Bai-Rural *vs.* Bai-Urban	0.512	0.712	0.655	0.593	0.769	0.771	0.896	0.761
		Han-Rural *vs.* Han-Urban	0.575	0.777	0.606	0.703	0.720	0.925	0.315	0.638
		Kazakh-Rural *vs.* Kazakh-Urban	0.802	0.953	0.901	0.877	0.972	0.332	0.233	0.662
	Species	Mongol-Rural *vs.* Mongol-Urban	0.698	0.335	0.761	0.850	0.375	0.450	0.135	0.606
		Tibetan-Rural *vs.* Tibetan-Urban	0.848	0.649	0.786	0.988	0.577	0.164	0.759	0.593
		Uyghur-Rural *vs.* Uyghur-Urban	0.711	0.752	0.862	0.936	0.801	0.579	0.663	0.895
		Zhuang-Rural *vs.* Zhuang-Urban	0.618	0.915	0.814	0.899	0.875	0.356	0.086	0.725
	(%) With significant differences		0	0	0	0	0	0	0	0
$q = 2$		Bai-Rural *vs.* Bai-Urban	0.874	0.886	0.951	0.946	0.822	0.389	0.639	0.955
		Han-Rural *vs.* Han-Urban	0.933	0.592	0.981	0.987	0.661	0.425	0.296	0.972
		Kazakh-Rural *vs.* Kazakh-Urban	0.786	0.720	0.924	0.903	0.591	0.517	0.257	0.917
	Phylum	Mongol-Rural *vs.* Mongol-Urban	0.964	0.615	0.874	0.893	0.680	0.531	0.212	0.893
		Tibetan-Rural *vs.* Tibetan-Urban	0.955	0.676	0.950	0.785	0.788	0.264	0.514	0.707
		Uyghur-Rural *vs.* Uyghur-Urban	0.749	0.620	0.650	0.630	0.618	0.938	0.073	0.761
		Zhuang-Rural *vs.* Zhuang-Urban	0.664	0.654	0.764	0.782	0.809	0.886	0.868	0.810
	(%) With significant differences		0	0	0	0	0	0	0	0
		Bai-Rural *vs.* Bai-Urban	0.632	0.969	0.595	0.623	0.879	0.680	0.722	0.632
		Han-Rural *vs.* Han-Urban	0.793	0.931	0.876	0.925	0.914	0.768	0.407	0.886
		Kazakh-Rural *vs.* Kazakh-Urban	0.741	0.833	0.954	0.786	0.986	0.460	0.449	0.568
	Family	Mongol-Rural *vs.* Mongol-Urban	0.682	0.480	0.703	0.763	0.494	0.133	0.441	0.657
		Tibetan-Rural *vs.* Tibetan-Urban	0.698	0.909	0.770	0.721	0.920	0.454	0.704	0.846
		Uyghur-Rural *vs.* Uyghur-Urban	0.517	0.847	0.652	0.643	0.924	0.040	0.650	0.795
		Zhuang-Rural *vs.* Zhuang-Urban	0.842	0.887	0.994	0.991	0.970	0.142	0.714	0.965
	(%) With significant differences		0	0	0	0	0	14.3% (1/7)	0	0

		Bai-Rural *vs.* Bai-Urban	0.696	0.559	0.986	0.925	0.754	0.235	0.201	0.902
		Han-Rural *vs.* Han-Urban	0.735	0.985	0.898	0.959	0.990	0.589	0.243	0.817
		Kazakh-Rural *vs.* Kazakh-Urban	0.948	0.991	0.778	0.920	0.815	0.420	0.337	0.652
	Genus	Mongol-Rural *vs.* Mongol-Urban	0.865	0.594	0.863	0.861	0.601	0.566	0.287	0.784
		Tibetan-Rural *vs.* Tibetan-Urban	0.623	0.977	0.791	0.788	0.840	0.723	0.551	0.866
		Uyghur-Rural *vs.* Uyghur-Urban	0.556	0.832	0.706	0.718	0.884	0.522	0.455	0.751
		Zhuang-Rural *vs.* Zhuang-Urban	0.927	0.729	0.911	0.978	0.885	0.370	0.444	0.765
	(%) With significant differences		0	0	0	0	0	0	0	0
		Bai-Rural *vs.* Bai-Urban	0.360	0.439	0.576	0.561	0.579	0.275	0.844	0.657
		Han-Rural *vs.* Han-Urban	0.609	0.717	0.707	0.807	0.705	0.898	0.748	0.698
		Kazakh-Rural *vs.* Kazakh-Urban	0.901	0.836	0.940	0.910	0.916	0.662	0.360	0.766
	Species	Mongol-Rural *vs.* Mongol-Urban	0.798	0.533	0.805	0.881	0.474	0.947	0.242	0.685
		Tibetan-Rural *vs.* Tibetan-Urban	0.966	0.627	0.857	0.866	0.632	0.677	0.775	0.899
		Uyghur-Rural *vs.* Uyghur-Urban	0.400	0.495	0.724	0.821	0.672	0.725	0.917	0.746
		Zhuang-Rural *vs.* Zhuang-Urban	0.622	0.811	0.762	0.810	0.761	0.371	0.066	0.822
	(%) With significant differences		0	0	0	0	0	0	0	0
		Bai-Rural *vs.* Bai-Urban	0.910	0.827	0.985	0.956	0.724	0.444	0.509	0.942
		Han-Rural *vs.* Han-Urban	0.857	0.542	0.918	0.933	0.609	0.675	0.319	0.862
		Kazakh-Rural *vs.* Kazakh-Urban	0.887	0.647	0.927	0.897	0.639	0.664	0.267	0.887
	Phylum	Mongol-Rural *vs.* Mongol-Urban	0.956	0.650	0.840	0.848	0.728	0.454	0.215	0.848
		Tibetan-Rural *vs.* Tibetan-Urban	0.795	0.780	0.991	0.801	0.812	0.315	0.484	0.750
		Uyghur-Rural *vs.* Uyghur-Urban	0.915	0.521	0.812	0.763	0.531	0.340	0.067	0.981
		Zhuang-Rural *vs.* Zhuang-Urban	0.726	0.666	0.777	0.865	0.727	0.577	0.999	0.704
	(%) With significant differences		0	0	0	0	0	0	0	0
		Bai-Rural *vs.* Bai-Urban	0.493	0.934	0.541	0.641	0.850	0.561	0.415	0.461
		Han-Rural *vs.* Han-Urban	0.828	0.924	0.927	0.981	0.889	0.067	0.638	0.934
		Kazakh-Rural *vs.* Kazakh-Urban	0.572	0.940	0.931	0.806	0.974	0.294	0.147	0.609
	Family	Mongol-Rural *vs.* Mongol-Urban	0.561	0.493	0.725	0.810	0.558	0.943	0.679	0.625
		Tibetan-Rural *vs.* Tibetan-Urban	0.659	0.869	0.734	0.737	0.923	0.283	0.660	0.809
		Uyghur-Rural *vs.* Uyghur-Urban	0.503	0.800	0.596	0.603	0.871	0.483	0.698	0.750
		Zhuang-Rural *vs.* Zhuang-Urban	0.892	0.888	0.937	0.997	0.913	0.784	0.997	0.886
$q = 3$	(%) With significant differences		0	0	0	0	0	0	0	0
		Bai-Rural *vs.* Bai-Urban	0.644	0.524	0.992	0.973	0.742	0.485	0.227	0.887
		Han-Rural *vs.* Han-Urban	0.723	0.933	0.882	0.969	0.993	0.392	0.270	0.809
		Kazakh-Rural *vs.* Kazakh-Urban	0.949	0.963	0.721	0.851	0.772	0.933	0.469	0.681
	Genus	Mongol-Rural *vs.* Mongol-Urban	0.954	0.720	0.829	0.796	0.677	0.745	0.410	0.844
		Tibetan-Rural *vs.* Tibetan-Urban	0.575	0.990	0.743	0.795	0.892	0.705	0.575	0.778
		Uyghur-Rural *vs.* Uyghur-Urban	0.504	0.832	0.757	0.812	0.924	0.265	0.527	0.757
		Zhuang-Rural *vs.* Zhuang-Urban	1.000	0.719	0.918	0.946	0.892	0.451	0.590	0.839
	(%) With significant differences		0	0	0	0	0	0	0	0
		Bai-Rural *vs.* Bai-Urban	0.385	0.403	0.638	0.577	0.618	0.580	0.735	0.749
		Han-Rural *vs.* Han-Urban	0.634	0.731	0.712	0.833	0.690	0.324	0.833	0.707
		Kazakh-Rural *vs.* Kazakh-Urban	0.977	0.784	0.971	0.889	0.794	0.537	0.358	0.834
	Species	Mongol-Rural *vs.* Mongol-Urban	0.666	0.504	0.722	0.851	0.425	0.477	0.407	0.601
		Tibetan-Rural *vs.* Tibetan-Urban	0.835	0.649	0.889	0.828	0.615	0.452	0.630	0.999
		Uyghur-Rural *vs.* Uyghur-Urban	0.363	0.484	0.725	0.855	0.638	0.340	0.261	0.702
		Zhuang-Rural *vs.* Zhuang-Urban	0.637	0.778	0.818	0.860	0.701	0.256	0.089	0.871
	(%) With significant differences		0	0	0	0	0	0	0	0

Scheme-2A

Diversity order	Taxon	Cohort	PL		PLEC					
			z	ln(c)	z	d	ln(c)	A_{max}	D_{max}	*LGD*
$q = 0$	Phylum	Bai *vs.* Han	0.306	0.469	0.641	0.723	0.830	0.637	0.975	0.768
		Bai *vs.* Kazakh	0.747	0.799	0.977	0.692	0.969	0.570	0.554	0.592
		Bai *vs.* Mongol	0.705	0.538	0.899	0.908	0.796	0.465	0.615	0.951
		Bai *vs.* Tibetan	0.946	0.697	0.942	0.935	0.433	0.292	0.958	0.665

	Bai *vs.* Uyghur	0.609	0.392	0.985	0.677	0.542	0.844	0.721	0.513
	Bai *vs.* Zhuang	0.974	0.994	0.895	1.000	0.692	0.110	0.806	0.783
	Han *vs.* Kazakh	0.619	0.661	0.677	0.547	0.852	0.416	0.642	0.667
	Han *vs.* Mongol	0.546	0.926	0.761	0.766	0.952	0.896	0.563	0.677
	Han *vs.* Tibetan	0.352	0.246	0.612	0.806	0.351	0.609	0.994	0.452
	Han *vs.* Uyghur	0.788	0.799	0.768	0.520	0.651	0.479	0.794	0.534
	Han *vs.* Zhuang	0.349	0.438	0.515	0.674	0.503	0.102	0.694	0.502
	Kazakh *vs.* Mongol	0.998	0.694	0.896	0.653	0.839	0.279	0.305	0.509
	Kazakh *vs.* Tibetan	0.771	0.480	0.961	0.631	0.422	0.168	0.419	0.352
	Kazakh *vs.* Uyghur	0.834	0.458	0.967	0.936	0.522	0.592	0.646	0.861
	Kazakh *vs.* Zhuang	0.729	0.764	0.946	0.673	0.636	0.067	0.271	0.369
	Mongol *vs.* Tibetan	0.758	0.282	0.844	0.982	0.332	0.653	0.585	0.687
	Mongol *vs.* Uyghur	0.865	0.726	0.929	0.564	0.711	0.339	0.504	0.438
	Mongol *vs.* Zhuang	0.737	0.517	0.791	0.905	0.520	0.100	0.837	0.755
	Tibetan *vs.* Uyghur	0.674	0.190	0.933	0.606	0.188	0.212	0.742	0.317
	Tibetan *vs.* Zhuang	0.967	0.671	0.972	0.942	0.711	0.118	0.689	0.871
	Uyghur *vs.* Zhuang	0.624	0.320	0.902	0.609	0.301	0.051	0.361	0.216
(%) With significant differences		0	0	0	0	0	0	0	0
	Bai *vs.* Han	0.361	0.384	0.516	0.474	0.580	0.410	0.841	0.884
	Bai *vs.* Kazakh	0.877	0.678	0.914	0.648	0.779	0.109	0.419	0.539
	Bai *vs.* Mongol	0.606	0.380	0.937	0.994	0.684	0.122	0.646	0.697
	Bai *vs.* Tibetan	0.650	0.851	0.729	0.846	0.963	0.164	0.428	0.892
	Bai *vs.* Uyghur	0.513	0.307	0.772	0.828	0.382	0.094	0.509	0.421
	Bai *vs.* Zhuang	0.556	0.801	0.695	0.874	0.929	0.315	0.418	0.733
	Han *vs.* Kazakh	0.621	0.737	0.549	0.410	0.856	0.345	0.573	0.541
	Han *vs.* Mongol	0.875	0.778	0.635	0.407	0.957	0.370	0.553	0.563
	Han *vs.* Tibetan	0.696	0.476	0.764	0.615	0.643	0.530	0.477	0.729
	Han *vs.* Uyghur	0.998	0.760	0.853	0.590	0.654	0.098	0.707	0.383
Family	Han *vs.* Zhuang	0.852	0.506	0.798	0.556	0.654	0.969	0.354	0.558
	Kazakh *vs.* Mongol	0.737	0.598	0.890	0.685	0.821	0.940	0.658	0.774
	Kazakh *vs.* Tibetan	0.789	0.775	0.707	0.513	0.821	0.668	0.829	0.666
	Kazakh *vs.* Uyghur	0.631	0.509	0.669	0.836	0.514	0.026	0.721	0.746
	Kazakh *vs.* Zhuang	0.683	0.849	0.620	0.486	0.846	0.244	0.802	0.720
	Mongol *vs.* Tibetan	0.862	0.448	0.873	0.856	0.681	0.717	0.650	0.821
	Mongol *vs.* Uyghur	0.909	0.999	0.836	0.811	0.744	0.056	0.814	0.588
	Mongol *vs.* Zhuang	0.984	0.476	0.811	0.870	0.687	0.269	0.575	0.998
	Tibetan *vs.* Uyghur	0.739	0.310	0.960	0.723	0.351	0.043	0.922	0.455
	Tibetan *vs.* Zhuang	0.841	0.943	0.946	0.972	0.958	0.396	1.000	0.807
	Uyghur *vs.* Zhuang	0.897	0.357	0.994	0.718	0.374	0.062	0.925	0.569
(%) With significant differences		0	0	0	0	0	9.5% (2/21)	0	0
Genus	Bai *vs.* Han	0.622	0.573	0.592	0.432	0.662	0.245	0.407	0.656
	Bai *vs.* Kazakh	0.912	0.889	0.928	0.770	0.757	0.319	0.989	0.804
	Bai *vs.* Mongol	0.970	0.627	0.978	0.915	0.775	0.201	0.276	0.813
	Bai *vs.* Tibetan	0.734	0.891	0.803	0.944	0.911	0.253	0.994	0.842
	Bai *vs.* Uyghur	0.793	0.428	0.826	0.879	0.448	0.986	0.975	0.701
	Bai *vs.* Zhuang	0.503	0.816	0.629	0.800	0.813	0.733	0.691	0.749
	Han *vs.* Kazakh	0.610	0.717	0.648	0.488	0.892	0.411	0.765	0.684
	Han *vs.* Mongol	0.693	0.911	0.606	0.532	0.978	0.536	0.995	0.780
	Han *vs.* Tibetan	0.950	0.692	0.811	0.496	0.769	0.476	0.356	0.452
	Han *vs.* Uyghur	0.964	0.755	0.888	0.622	0.664	0.211	0.791	0.555
	Han *vs.* Zhuang	0.829	0.698	0.982	0.611	0.834	0.303	0.287	0.376
	Kazakh *vs.* Mongol	0.882	0.736	0.975	0.760	0.944	0.386	0.428	0.700
	Kazakh *vs.* Tibetan	0.664	0.982	0.779	0.682	0.861	0.487	0.975	0.883
	Kazakh *vs.* Uyghur	0.659	0.455	0.756	0.893	0.543	0.204	0.873	0.946
	Kazakh *vs.* Zhuang	0.497	0.966	0.624	0.627	0.947	0.442	0.887	0.962
	Mongol *vs.* Tibetan	0.807	0.680	0.802	0.975	0.863	0.792	0.241	0.643

		Mongol *vs*. Uyghur	0.811	0.847	0.829	0.856	0.711	0.168	0.436	0.572
		Mongol *vs*. Zhuang	0.625	0.721	0.659	0.899	0.887	0.275	0.206	0.572
		Tibetan *vs*. Uyghur	0.995	0.458	0.980	0.847	0.492	0.192	0.968	0.826
		Tibetan *vs*. Zhuang	0.786	0.925	0.819	0.859	0.924	0.298	0.597	0.890
		Uyghur *vs*. Zhuang	0.820	0.482	0.872	0.768	0.503	0.735	0.822	0.904
	(%) With significant differences		0	0	0	0	0	0	0	0
		Bai *vs*. Han	0.041	0.873	0.449	0.391	0.954	0.311	0.108	0.002
		Bai *vs*. Kazakh	0.090	0.985	0.305	0.655	0.947	0.478	0.169	0.228
		Bai *vs*. Mongol	0.138	0.786	0.396	0.882	0.904	0.537	0.094	0.035
		Bai *vs*. Tibetan	0.130	0.879	0.508	0.920	0.916	0.327	0.135	0.103
		Bai *vs*. Uyghur	0.105	0.695	0.461	0.863	0.662	0.799	0.121	0.200
		Bai *vs*. Zhuang	0.040	0.745	0.377	0.676	0.786	0.533	0.115	0.020
		Han *vs*. Kazakh	0.749	0.855	0.565	0.364	0.880	0.412	0.642	0.539
		Han *vs*. Mongol	0.856	0.836	0.687	0.494	0.965	0.359	0.661	0.587
		Han *vs*. Tibetan	0.682	0.744	0.994	0.446	0.851	0.530	0.226	0.257
		Han *vs*. Uyghur	0.868	0.787	0.831	0.598	0.723	0.316	0.792	0.558
	Species	Han *vs*. Zhuang	0.924	0.615	0.888	0.610	0.748	0.423	0.344	0.508
		Kazakh *vs*. Mongol	0.915	0.774	0.860	0.650	0.897	0.981	0.622	0.653
		Kazakh *vs*. Tibetan	0.531	0.921	0.600	0.576	0.996	0.476	0.901	0.993
		Kazakh *vs*. Uyghur	0.896	0.648	0.775	0.769	0.610	0.564	0.719	0.962
		Kazakh *vs*. Zhuang	0.796	0.792	0.645	0.466	0.894	0.948	0.904	0.664
		Mongol *vs*. Tibetan	0.653	0.680	0.722	0.976	0.847	0.541	0.243	0.506
		Mongol *vs*. Uyghur	0.996	0.983	0.940	0.842	0.814	0.712	0.792	0.684
		Mongol *vs*. Zhuang	0.906	0.566	0.818	0.860	0.797	0.953	0.469	0.934
		Tibetan *vs*. Uyghur	0.606	0.559	0.826	0.820	0.588	0.346	0.792	0.967
		Tibetan *vs*. Zhuang	0.649	0.859	0.901	0.771	0.896	0.564	0.515	0.524
		Uyghur *vs*. Zhuang	0.926	0.480	0.880	0.706	0.527	0.692	0.988	0.687
	(%) With significant differences		9.5% (2/21)	0	0	0	0	0	0	14.3% (3/21)
q = 1		Bai *vs*. Han	0.799	0.862	0.836	0.701	0.805	0.451	0.336	0.972
		Bai *vs*. Kazakh	0.742	0.608	0.713	0.674	0.724	0.580	0.108	0.734
		Bai *vs*. Mongol	0.780	0.541	0.787	0.762	0.606	0.874	0.476	0.913
		Bai *vs*. Tibetan	0.885	0.757	0.875	0.967	0.832	0.383	0.297	0.769
		Bai *vs*. Uyghur	0.966	0.454	0.998	0.975	0.479	0.635	0.093	0.979
		Bai *vs*. Zhuang	0.997	0.985	0.991	0.998	0.993	0.061	0.958	0.988
		Han *vs*. Kazakh	0.585	0.477	0.517	0.533	0.529	0.339	0.012	0.697
		Han *vs*. Mongol	0.992	0.410	0.947	0.992	0.452	0.447	0.064	0.972
		Han *vs*. Tibetan	0.666	0.623	0.699	0.706	0.633	0.834	0.035	0.729
		Han *vs*. Uyghur	0.824	0.312	0.912	0.870	0.346	0.333	0.010	0.952
	Phylum	Han *vs*. Zhuang	0.792	0.857	0.837	0.718	0.812	0.120	0.370	0.946
		Kazakh *vs*. Mongol	0.504	0.931	0.478	0.451	0.995	0.468	0.138	0.619
		Kazakh *vs*. Tibetan	0.822	0.800	0.750	0.652	0.837	0.215	0.501	0.939
		Kazakh *vs*. Uyghur	0.779	0.841	0.681	0.728	0.763	0.830	0.885	0.777
		Kazakh *vs*. Zhuang	0.740	0.561	0.716	0.687	0.685	0.038	0.169	0.717
		Mongol *vs*. Tibetan	0.632	0.834	0.665	0.745	0.822	0.437	0.539	0.694
		Mongol *vs*. Uyghur	0.782	0.686	0.825	0.808	0.691	0.514	0.158	0.896
		Mongol *vs*. Zhuang	0.781	0.538	0.793	0.770	0.608	0.066	0.600	0.926
		Tibetan *vs*. Uyghur	0.926	0.621	0.887	0.983	0.601	0.236	0.541	0.792
		Tibetan *vs*. Zhuang	0.869	0.733	0.858	0.967	0.803	0.069	0.376	0.763
		Uyghur *vs*. Zhuang	0.967	0.392	0.992	0.974	0.443	0.034	0.199	0.974
	(%) With significant differences		0	0	0	0	0	9.5% (2/21)	14.3% (3/21)	0
	Family	Bai *vs*. Han	0.579	0.808	0.552	0.454	0.804	0.856	0.720	0.738
		Bai *vs*. Kazakh	0.573	0.873	0.431	0.354	0.973	0.169	0.174	0.773
		Bai *vs*. Mongol	0.689	0.468	0.753	0.788	0.581	0.214	0.394	0.686
		Bai *vs*. Tibetan	0.758	0.900	0.801	0.842	0.886	0.316	0.736	0.901
		Bai *vs*. Uyghur	0.928	0.345	0.883	0.653	0.379	0.149	0.066	0.893

	Bai *vs.* Zhuang	0.945	0.868	0.830	0.877	0.978	0.466	0.517	0.934
	Han *vs.* Kazakh	0.368	0.985	0.273	0.239	0.872	0.395	0.052	0.590
	Han *vs.* Mongol	0.962	0.553	0.930	0.681	0.636	0.454	0.154	0.839
	Han *vs.* Tibetan	0.393	0.654	0.408	0.349	0.679	0.669	0.418	0.621
	Han *vs.* Uyghur	0.636	0.466	0.608	0.496	0.490	0.337	0.026	0.891
	Han *vs.* Zhuang	0.508	0.922	0.422	0.315	0.811	0.501	0.222	0.665
	Kazakh *vs.* Mongol	0.382	0.639	0.342	0.327	0.589	0.694	0.453	0.526
	Kazakh *vs.* Tibetan	0.747	0.780	0.636	0.510	0.909	0.360	0.340	0.865
	Kazakh *vs.* Uyghur	0.590	0.427	0.516	0.625	0.384	0.651	0.678	0.631
	Kazakh *vs.* Zhuang	0.575	0.979	0.491	0.369	0.990	0.132	0.402	0.804
	Mongol *vs.* Tibetan	0.554	0.433	0.598	0.642	0.539	0.569	0.688	0.612
	Mongol *vs.* Uyghur	0.643	0.907	0.683	0.601	0.853	0.555	0.264	0.738
	Mongol *vs.* Zhuang	0.613	0.538	0.605	0.668	0.560	0.171	0.902	0.596
	Tibetan *vs.* Uyghur	0.912	0.371	0.922	0.819	0.414	0.306	0.201	0.817
	Tibetan *vs.* Zhuang	0.823	0.753	0.905	0.907	0.867	0.227	0.780	0.961
	Uyghur *vs.* Zhuang	0.966	0.394	0.988	0.697	0.336	0.113	0.216	0.830
(%) With significant differences		0	0	0	0	0	0	4.8% (1/21)	0
	Bai *vs.* Han	0.514	0.642	0.604	0.473	0.749	0.844	0.924	0.742
	Bai *vs.* Kazakh	0.539	0.903	0.553	0.452	0.895	0.522	0.185	0.734
	Bai *vs.* Mongol	0.684	0.612	0.701	0.769	0.611	0.977	0.782	0.680
	Bai *vs.* Tibetan	0.938	0.691	0.976	0.967	0.757	0.667	0.415	0.985
	Bai *vs.* Uyghur	0.985	0.482	0.904	0.604	0.492	0.437	0.201	0.862
	Bai *vs.* Zhuang	0.854	0.837	0.924	0.915	0.889	0.298	0.893	0.928
	Han *vs.* Kazakh	0.306	0.789	0.347	0.319	0.885	0.641	0.123	0.525
	Han *vs.* Mongol	0.899	0.880	0.991	0.701	0.781	0.749	0.736	0.864
	Han *vs.* Tibetan	0.520	0.355	0.669	0.505	0.560	0.642	0.121	0.803
	Han *vs.* Uyghur	0.652	0.729	0.603	0.441	0.627	0.586	0.136	0.919
Genus	Han *vs.* Zhuang	0.630	0.784	0.676	0.470	0.874	0.515	0.914	0.795
	Kazakh *vs.* Mongol	0.373	0.732	0.421	0.386	0.710	0.536	0.334	0.573
	Kazakh *vs.* Tibetan	0.720	0.635	0.696	0.609	0.720	0.749	0.095	0.788
	Kazakh *vs.* Uyghur	0.532	0.521	0.632	0.828	0.518	0.746	0.918	0.560
	Kazakh *vs.* Zhuang	0.430	0.971	0.492	0.437	0.983	0.186	0.206	0.656
	Mongol *vs.* Tibetan	0.676	0.435	0.726	0.761	0.523	0.664	0.222	0.737
	Mongol *vs.* Uyghur	0.718	0.847	0.679	0.531	0.882	0.472	0.365	0.782
	Mongol *vs.* Zhuang	0.793	0.730	0.756	0.797	0.693	0.272	0.857	0.746
	Tibetan *vs.* Uyghur	0.968	0.419	0.967	0.761	0.469	0.624	0.118	0.920
	Tibetan *vs.* Zhuang	0.814	0.542	0.919	0.918	0.654	0.263	0.275	0.954
	Uyghur *vs.* Zhuang	0.845	0.527	0.850	0.585	0.507	0.164	0.271	0.926
(%) With significant differences		0	0	0	0	0	0	0	0
Species	Bai *vs.* Han	0.992	0.598	0.932	0.601	0.750	0.621	0.029	0.858
	Bai *vs.* Kazakh	0.286	0.856	0.338	0.311	0.872	0.405	0.108	0.561
	Bai *vs.* Mongol	0.803	0.525	0.914	0.951	0.619	0.507	0.017	0.854
	Bai *vs.* Tibetan	0.566	0.870	0.691	0.861	0.842	0.730	0.289	0.675
	Bai *vs.* Uyghur	0.580	0.579	0.613	0.549	0.666	0.429	0.044	0.698
	Bai *vs.* Zhuang	0.507	0.798	0.677	0.906	0.898	0.852	0.033	0.488
	Han *vs.* Kazakh	0.308	0.552	0.295	0.272	0.650	0.384	0.749	0.657
	Han *vs.* Mongol	0.772	0.865	0.837	0.669	0.844	0.413	0.273	0.981
	Han *vs.* Tibetan	0.523	0.520	0.589	0.493	0.623	0.487	0.356	0.728
	Han *vs.* Uyghur	0.564	0.904	0.552	0.470	0.901	0.451	0.315	0.785
	Han *vs.* Zhuang	0.543	0.707	0.610	0.502	0.782	0.575	0.976	0.680
	Kazakh *vs.* Mongol	0.427	0.485	0.444	0.353	0.540	0.742	0.697	0.718
	Kazakh *vs.* Tibetan	0.611	0.986	0.610	0.476	0.983	0.542	0.485	0.891
	Kazakh *vs.* Uyghur	0.653	0.499	0.712	0.752	0.592	0.647	0.472	0.858
	Kazakh *vs.* Zhuang	0.401	0.662	0.396	0.271	0.752	0.465	0.723	0.856
	Mongol *vs.* Tibetan	0.802	0.496	0.799	0.799	0.568	0.703	0.169	0.830

		Mongol *vs.* Uyghur	0.736	0.943	0.712	0.567	0.935	0.881	0.874	0.782
		Mongol *vs.* Zhuang	0.811	0.573	0.826	0.840	0.617	0.607	0.306	0.704
		Tibetan *vs.* Uyghur	0.928	0.535	0.860	0.711	0.613	0.643	0.191	0.996
		Tibetan *vs.* Zhuang	0.902	0.692	0.930	0.897	0.770	0.807	0.394	0.973
		Uyghur *vs.* Zhuang	0.852	0.639	0.754	0.554	0.664	0.536	0.243	0.963
	(%) With significant differences		0	0	0	0	0	0	19.0% (4/21)	0
$q = 2$		Bai *vs.* Han	0.893	0.703	0.888	0.858	0.721	0.232	0.193	0.933
		Bai *vs.* Kazakh	0.885	0.665	0.812	0.822	0.784	0.504	0.217	0.842
		Bai *vs.* Mongol	0.901	0.641	0.866	0.802	0.721	0.391	0.568	0.969
		Bai *vs.* Tibetan	0.826	0.629	0.902	0.959	0.678	0.702	0.117	0.803
		Bai *vs.* Uyghur	0.956	0.541	0.898	0.911	0.546	0.412	0.172	0.864
		Bai *vs.* Zhuang	0.978	0.883	0.980	0.979	0.907	0.675	0.793	0.988
		Han *vs.* Kazakh	0.768	0.389	0.686	0.706	0.504	0.176	0.023	0.729
		Han *vs.* Mongol	0.999	0.364	0.956	0.907	0.479	0.358	0.046	0.904
		Han *vs.* Tibetan	0.719	0.400	0.769	0.783	0.469	0.229	0.006	0.743
		Han *vs.* Uyghur	0.977	0.291	0.960	0.960	0.333	0.172	0.028	0.883
	Phylum	Han *vs.* Zhuang	0.913	0.808	0.900	0.856	0.848	0.213	0.384	0.918
		Kazakh *vs.* Mongol	0.756	0.845	0.662	0.647	0.959	0.235	0.285	0.856
		Kazakh *vs.* Tibetan	0.983	0.979	0.890	0.833	0.913	0.749	0.889	0.996
		Kazakh *vs.* Uyghur	0.869	0.912	0.743	0.771	0.805	0.815	0.950	0.777
		Kazakh *vs.* Zhuang	0.857	0.543	0.805	0.814	0.662	0.780	0.190	0.848
		Mongol *vs.* Tibetan	0.701	0.885	0.751	0.756	0.860	0.305	0.213	0.812
		Mongol *vs.* Uyghur	0.978	0.686	0.983	0.979	0.654	0.170	0.296	0.809
		Mongol *vs.* Zhuang	0.921	0.566	0.889	0.829	0.636	0.279	0.431	0.988
		Tibetan *vs.* Uyghur	0.828	0.866	0.816	0.884	0.856	0.658	0.856	0.722
		Tibetan *vs.* Zhuang	0.822	0.581	0.860	0.935	0.591	0.973	0.143	0.815
		Uyghur *vs.* Zhuang	0.966	0.430	0.925	0.928	0.446	0.657	0.194	0.845
	(%) With significant differences		0	0	0	0	0	0	19.0% (4/21)	0
		Bai *vs.* Han	0.571	0.823	0.520	0.440	0.837	0.486	0.547	0.733
		Bai *vs.* Kazakh	0.482	0.865	0.574	0.542	0.952	0.904	0.496	0.812
		Bai *vs.* Mongol	0.639	0.562	0.704	0.742	0.622	0.978	0.814	0.658
		Bai *vs.* Tibetan	0.857	0.852	0.904	0.894	0.898	0.960	0.979	0.983
		Bai *vs.* Uyghur	0.861	0.444	0.834	0.659	0.515	0.634	0.129	0.967
		Bai *vs.* Zhuang	0.989	0.900	0.944	0.989	0.988	0.185	0.620	0.910
		Han *vs.* Kazakh	0.249	0.700	0.305	0.337	0.808	0.546	0.159	0.636
		Han *vs.* Mongol	0.939	0.666	0.930	0.718	0.711	0.474	0.406	0.856
		Han *vs.* Tibetan	0.396	0.603	0.451	0.368	0.689	0.500	0.514	0.706
		Han *vs.* Uyghur	0.515	0.552	0.509	0.481	0.649	0.377	0.047	0.715
	Family	Han *vs.* Zhuang	0.540	0.915	0.460	0.396	0.790	0.590	0.216	0.624
		Kazakh *vs.* Mongol	0.276	0.523	0.409	0.444	0.621	0.950	0.553	0.547
		Kazakh *vs.* Tibetan	0.584	0.998	0.696	0.624	0.966	0.957	0.438	0.832
		Kazakh *vs.* Uyghur	0.545	0.379	0.731	0.822	0.493	0.357	0.565	0.842
		Kazakh *vs.* Zhuang	0.426	0.773	0.575	0.493	0.959	0.193	0.639	0.856
		Mongol *vs.* Tibetan	0.518	0.458	0.623	0.647	0.590	0.999	0.740	0.665
		Mongol *vs.* Uyghur	0.530	0.922	0.585	0.562	0.913	0.621	0.212	0.616
		Mongol *vs.* Zhuang	0.598	0.613	0.619	0.731	0.609	0.202	0.850	0.595
		Tibetan *vs.* Uyghur	0.983	0.425	0.925	0.764	0.513	0.641	0.164	0.971
		Tibetan *vs.* Zhuang	0.856	0.765	0.938	0.865	0.910	0.188	0.594	0.923
		Uyghur *vs.* Zhuang	0.862	0.483	0.849	0.625	0.446	0.140	0.205	0.951
	(%) With significant differences		0	0	0	0	0	0	4.8% (1/21)	0
	Genus	Bai *vs.* Han	0.499	0.720	0.549	0.446	0.765	0.868	0.570	0.664
		Bai *vs.* Kazakh	0.487	0.907	0.477	0.374	0.939	0.627	0.076	0.715
		Bai *vs.* Mongol	0.578	0.654	0.624	0.741	0.605	0.572	0.852	0.603
		Bai *vs.* Tibetan	0.992	0.539	0.881	0.978	0.775	0.036	0.170	0.741
		Bai *vs.* Uyghur	0.953	0.521	0.798	0.487	0.549	0.443	0.197	0.873

		Bai *vs.* Zhuang	0.805	0.911	0.867	0.950	0.854	0.716	0.849	0.787
		Han *vs.* Kazakh	0.268	0.855	0.279	0.269	0.867	0.777	0.028	0.438
		Han *vs.* Mongol	0.958	0.850	0.989	0.666	0.779	0.499	0.768	0.846
		Han *vs.* Tibetan	0.579	0.276	0.825	0.563	0.546	0.049	0.020	0.929
		Han *vs.* Uyghur	0.579	0.754	0.470	0.331	0.709	0.613	0.076	0.838
		Han *vs.* Zhuang	0.687	0.808	0.661	0.455	0.933	0.656	0.847	0.902
		Kazakh *vs.* Mongol	0.271	0.760	0.315	0.316	0.684	0.990	0.075	0.425
		Kazakh *vs.* Tibetan	0.680	0.543	0.568	0.579	0.743	0.021	0.046	0.584
		Kazakh *vs.* Uyghur	0.523	0.613	0.648	0.879	0.581	0.523	0.347	0.562
		Kazakh *vs.* Zhuang	0.386	0.981	0.414	0.422	0.914	0.836	0.135	0.529
		Mongol *vs.* Tibetan	0.701	0.324	0.846	0.839	0.518	0.027	0.102	0.961
		Mongol *vs.* Uyghur	0.625	0.891	0.540	0.419	0.933	0.811	0.203	0.710
		Mongol *vs.* Zhuang	0.757	0.724	0.742	0.777	0.757	0.757	0.986	0.789
		Tibetan *vs.* Uyghur	0.963	0.377	0.790	0.672	0.535	0.012	0.090	0.839
		Tibetan *vs.* Zhuang	0.873	0.437	0.946	0.997	0.645	0.030	0.169	0.851
		Uyghur *vs.* Zhuang	0.800	0.582	0.718	0.525	0.619	0.585	0.282	0.909
	(%) With significant differences		0	0	0	0	0	23.8% (5/21)	14.3% (3/21)	0
		Bai *vs.* Han	0.929	0.824	0.970	0.695	0.885	0.237	0.164	0.879
		Bai *vs.* Kazakh	0.234	0.818	0.315	0.330	0.820	0.421	0.102	0.541
		Bai *vs.* Mongol	0.816	0.603	0.942	0.918	0.697	0.777	0.039	0.822
		Bai *vs.* Tibetan	0.683	0.721	0.864	0.947	0.853	0.582	0.877	0.897
		Bai *vs.* Uyghur	0.473	0.735	0.547	0.523	0.819	0.474	0.034	0.587
		Bai *vs.* Zhuang	0.589	0.847	0.622	0.831	0.939	0.968	0.046	0.491
		Han *vs.* Kazakh	0.326	0.700	0.343	0.347	0.746	0.151	0.399	0.650
		Han *vs.* Mongol	0.886	0.784	0.898	0.731	0.811	0.219	0.347	0.961
		Han *vs.* Tibetan	0.716	0.598	0.828	0.648	0.756	0.167	0.216	0.999
		Han *vs.* Uyghur	0.523	0.900	0.534	0.521	0.943	0.180	0.284	0.735
	Species	Han *vs.* Zhuang	0.713	0.930	0.602	0.495	0.816	0.253	0.677	0.736
		Kazakh *vs.* Mongol	0.351	0.497	0.379	0.367	0.550	0.529	0.841	0.643
		Kazakh *vs.* Tibetan	0.536	0.910	0.534	0.525	0.996	0.671	0.263	0.688
		Kazakh *vs.* Uyghur	0.706	0.628	0.762	0.809	0.708	0.676	0.844	0.886
		Kazakh *vs.* Zhuang	0.346	0.670	0.446	0.383	0.847	0.458	0.484	0.836
		Mongol *vs.* Tibetan	0.853	0.484	0.921	0.889	0.610	0.739	0.153	0.972
		Mongol *vs.* Uyghur	0.593	0.870	0.606	0.515	0.845	0.652	0.683	0.732
		Mongol *vs.* Zhuang	0.834	0.669	0.721	0.733	0.606	0.783	0.583	0.734
		Tibetan *vs.* Uyghur	0.788	0.571	0.706	0.685	0.734	0.836	0.182	0.735
		Tibetan *vs.* Zhuang	0.965	0.610	0.828	0.901	0.875	0.596	0.260	0.736
		Uyghur *vs.* Zhuang	0.650	0.836	0.758	0.586	0.783	0.495	0.360	0.959
	(%) With significant differences		0	0	0	0	0	0	14.3% (3/21)	0
q = 3	Phylum	Bai *vs.* Han	0.963	0.640	0.940	0.933	0.681	0.307	0.163	0.988
		Bai *vs.* Kazakh	0.946	0.677	0.880	0.877	0.771	0.704	0.269	0.964
		Bai *vs.* Mongol	0.952	0.703	0.911	0.862	0.764	0.562	0.584	0.989
		Bai *vs.* Tibetan	0.810	0.607	0.897	0.948	0.664	0.738	0.104	0.810
		Bai *vs.* Uyghur	0.904	0.570	0.842	0.849	0.565	0.615	0.209	0.811
		Bai *vs.* Zhuang	0.982	0.868	1.000	0.997	0.883	0.491	0.739	0.998
		Han *vs.* Kazakh	0.899	0.360	0.804	0.794	0.461	0.253	0.028	0.930
		Han *vs.* Mongol	0.985	0.343	0.936	0.883	0.475	0.420	0.035	0.980
		Han *vs.* Tibetan	0.756	0.347	0.798	0.854	0.433	0.381	0.001	0.789
		Han *vs.* Uyghur	0.913	0.271	0.848	0.839	0.301	0.228	0.036	0.740
		Han *vs.* Zhuang	0.984	0.755	0.939	0.905	0.835	0.450	0.408	0.984
		Kazakh *vs.* Mongol	0.879	0.817	0.767	0.736	0.906	0.367	0.354	0.981
		Kazakh *vs.* Tibetan	0.884	0.935	0.976	0.897	0.867	0.446	0.645	0.836
		Kazakh *vs.* Uyghur	0.881	0.918	0.773	0.780	0.806	0.828	1.000	0.816
		Kazakh *vs.* Zhuang	0.918	0.544	0.881	0.875	0.615	0.296	0.191	0.976
		Mongol *vs.* Tibetan	0.738	0.785	0.788	0.817	0.793	0.768	0.157	0.811

	Mongol *vs.* Uyghur	0.928	0.675	0.860	0.853	0.620	0.296	0.326	0.743
	Mongol *vs.* Zhuang	0.970	0.614	0.894	0.859	0.645	0.911	0.393	0.984
	Tibetan *vs.* Uyghur	0.781	0.945	0.765	0.828	0.919	0.414	0.637	0.673
	Tibetan *vs.* Zhuang	0.814	0.561	0.880	0.955	0.553	0.691	0.078	0.804
	Uyghur *vs.* Zhuang	0.896	0.445	0.842	0.864	0.420	0.263	0.195	0.765
(%) With significant differences		0	0	0	0	0	0	19.0% (4/21)	0
	Bai *vs.* Han	0.612	0.804	0.597	0.494	0.882	0.440	0.658	0.799
	Bai *vs.* Kazakh	0.452	0.717	0.612	0.612	0.915	0.291	0.682	0.837
	Bai *vs.* Mongol	0.666	0.621	0.737	0.776	0.702	0.547	0.997	0.695
	Bai *vs.* Tibetan	0.878	0.829	0.912	0.937	0.877	0.391	0.844	0.971
	Bai *vs.* Uyghur	0.813	0.500	0.757	0.653	0.611	0.312	0.219	0.851
	Bai *vs.* Zhuang	0.934	0.935	0.889	0.988	0.928	0.312	0.658	0.812
	Han *vs.* Kazakh	0.228	0.537	0.352	0.366	0.784	0.218	0.310	0.690
	Han *vs.* Mongol	0.933	0.755	0.953	0.758	0.749	0.285	0.673	0.811
	Han *vs.* Tibetan	0.444	0.579	0.512	0.431	0.703	0.890	0.753	0.762
	Han *vs.* Uyghur	0.488	0.611	0.482	0.487	0.731	0.228	0.071	0.696
Family	Han *vs.* Zhuang	0.525	0.874	0.462	0.430	0.759	0.998	0.290	0.617
	Kazakh *vs.* Mongol	0.261	0.465	0.446	0.500	0.651	0.610	0.601	0.589
	Kazakh *vs.* Tibetan	0.514	0.862	0.692	0.629	0.973	0.193	0.510	0.885
	Kazakh *vs.* Uyghur	0.558	0.341	0.836	0.891	0.554	0.920	0.569	0.954
	Kazakh *vs.* Zhuang	0.411	0.653	0.639	0.555	0.970	0.189	0.784	0.974
	Mongol *vs.* Tibetan	0.562	0.484	0.658	0.704	0.616	0.283	0.812	0.668
	Mongol *vs.* Uyghur	0.498	0.899	0.565	0.563	0.948	0.664	0.226	0.541
	Mongol *vs.* Zhuang	0.594	0.669	0.595	0.745	0.652	0.223	0.714	0.565
	Tibetan *vs.* Uyghur	0.926	0.437	0.840	0.724	0.552	0.191	0.174	0.880
	Tibetan *vs.* Zhuang	0.946	0.766	0.988	0.935	0.926	0.680	0.452	0.837
	Uyghur *vs.* Zhuang	0.835	0.507	0.777	0.592	0.495	0.170	0.226	0.965
(%) With significant differences		0	0	0	0	0	0	0	0
	Bai *vs.* Han	0.444	0.690	0.559	0.470	0.783	0.899	0.471	0.656
	Bai *vs.* Kazakh	0.487	0.884	0.435	0.353	0.955	0.239	0.082	0.719
	Bai *vs.* Mongol	0.533	0.637	0.562	0.710	0.570	0.310	0.680	0.512
	Bai *vs.* Tibetan	0.741	0.634	0.767	0.965	0.870	0.222	0.133	0.520
	Bai *vs.* Uyghur	0.998	0.496	0.700	0.404	0.592	0.205	0.261	0.882
	Bai *vs.* Zhuang	0.765	0.914	0.825	0.947	0.856	0.295	0.708	0.693
	Han *vs.* Kazakh	0.243	0.871	0.257	0.268	0.853	0.531	0.026	0.433
	Han *vs.* Mongol	0.962	0.847	0.951	0.717	0.735	0.453	0.833	0.770
	Han *vs.* Tibetan	0.878	0.333	0.996	0.610	0.640	0.429	0.023	0.601
	Han *vs.* Uyghur	0.566	0.762	0.409	0.284	0.737	0.457	0.092	0.805
Genus	Han *vs.* Zhuang	0.693	0.802	0.727	0.526	0.967	0.492	0.985	0.981
	Kazakh *vs.* Mongol	0.248	0.760	0.275	0.305	0.627	0.806	0.072	0.384
	Kazakh *vs.* Tibetan	0.517	0.608	0.473	0.563	0.838	0.988	0.041	0.398
	Kazakh *vs.* Uyghur	0.500	0.621	0.684	0.946	0.614	0.623	0.321	0.581
	Kazakh *vs.* Zhuang	0.377	0.967	0.384	0.438	0.869	0.765	0.146	0.504
	Mongol *vs.* Tibetan	0.945	0.399	0.969	0.850	0.561	0.748	0.106	0.861
	Mongol *vs.* Uyghur	0.619	0.877	0.451	0.354	0.941	0.652	0.222	0.633
	Mongol *vs.* Zhuang	0.746	0.712	0.753	0.789	0.749	0.998	0.922	0.808
	Tibetan *vs.* Uyghur	0.806	0.444	0.649	0.634	0.622	0.792	0.094	0.636
	Tibetan *vs.* Zhuang	0.885	0.544	0.857	0.980	0.709	0.711	0.140	0.684
	Uyghur *vs.* Zhuang	0.807	0.599	0.618	0.486	0.723	0.584	0.272	0.823
(%) With significant differences		0	0	0	0	0	0	14.3% (3/21)	0
Species	Bai *vs.* Han	0.870	0.996	0.972	0.774	0.991	0.971	0.367	0.878
	Bai *vs.* Kazakh	0.220	0.775	0.326	0.402	0.773	0.270	0.100	0.517
	Bai *vs.* Mongol	0.807	0.779	0.921	0.965	0.786	0.448	0.069	0.854
	Bai *vs.* Tibetan	0.810	0.689	0.956	0.929	0.826	0.261	0.866	0.933
	Bai *vs.* Uyghur	0.421	0.884	0.479	0.457	0.963	0.208	0.070	0.567

		Bai *vs*. Zhuang	0.603	0.984	0.655	0.804	0.884	0.679	0.159	0.569
		Han *vs*. Kazakh	0.348	0.817	0.410	0.445	0.823	0.443	0.290	0.640
		Han *vs*. Mongol	0.964	0.756	0.950	0.743	0.786	0.656	0.445	0.984
		Han *vs*. Tibetan	0.933	0.690	0.969	0.658	0.875	0.468	0.210	0.761
		Han *vs*. Uyghur	0.543	0.894	0.542	0.505	0.948	0.397	0.334	0.768
		Han *vs*. Zhuang	0.803	0.996	0.687	0.546	0.919	0.822	0.661	0.829
		Kazakh *vs*. Mongol	0.340	0.603	0.401	0.445	0.608	0.518	0.522	0.572
		Kazakh *vs*. Tibetan	0.476	0.906	0.530	0.632	0.965	0.885	0.212	0.521
		Kazakh *vs*. Uyghur	0.711	0.705	0.837	0.945	0.759	0.712	0.747	0.831
		Kazakh *vs*. Zhuang	0.356	0.772	0.497	0.520	0.871	0.421	0.389	0.719
		Mongol *vs*. Tibetan	0.971	0.551	0.995	0.914	0.683	0.596	0.205	0.825
		Mongol *vs*. Uyghur	0.549	0.875	0.557	0.492	0.812	0.364	0.673	0.718
		Mongol *vs*. Zhuang	0.833	0.733	0.745	0.769	0.670	0.720	0.741	0.789
		Tibetan *vs*. Uyghur	0.672	0.632	0.635	0.670	0.822	0.703	0.233	0.609
		Tibetan *vs*. Zhuang	0.895	0.686	0.772	0.915	0.913	0.480	0.281	0.613
		Uyghur *vs*. Zhuang	0.630	0.870	0.693	0.563	0.854	0.308	0.483	0.890
	(%) With significant differences		0	0	0	0	0	0	0	0

Scheme-2B (Rural)

**Diversity order**	**Taxon**	**Cohort**	**PL**		**PLEC**					
			z	**ln(*c*)**	z	d	**ln(*c*)**	A_{max}	D_{max}	***LGD***
$q = 0$		Bai-Rural *vs*. Han-Rural	0.649	0.506	0.846	0.704	0.799	0.818	0.613	0.940
		Bai-Rural *vs*. Kazakh-Rural	0.839	0.690	0.857	0.532	0.583	0.714	0.818	0.643
		Bai-Rural *vs*. Mongol-Rural	0.940	0.435	0.875	0.808	0.482	0.414	0.318	0.792
		Bai-Rural *vs*. Tibetan-Rural	0.716	0.978	0.829	0.920	0.880	0.762	0.685	0.903
		Bai-Rural *vs*. Uyghur-Rural	0.865	0.539	0.894	0.815	0.596	1.000	0.989	0.894
		Bai-Rural *vs*. Zhuang-Rural	0.989	0.893	0.990	0.957	0.985	0.661	0.669	0.821
		Han-Rural *vs*. Kazakh-Rural	0.882	0.781	0.733	0.413	0.763	0.683	0.554	0.466
		Han-Rural *vs*. Mongol-Rural	0.672	0.819	0.994	0.979	0.569	0.576	0.421	0.799
		Han-Rural *vs*. Tibetan-Rural	0.997	0.521	0.919	0.864	0.722	0.941	0.323	0.785
		Han-Rural *vs*. Uyghur-Rural	0.591	0.989	0.780	0.695	0.720	0.941	0.952	0.762
	Phylum	Han-Rural *vs*. Zhuang-Rural	0.640	0.578	0.863	0.795	0.804	0.869	0.929	0.804
		Kazakh-Rural *vs*. Mongol-Rural	0.874	0.604	0.687	0.337	0.734	0.222	0.314	0.475
		Kazakh-Rural *vs*. Tibetan-Rural	0.897	0.696	0.699	0.472	0.499	0.475	0.881	0.656
		Kazakh-Rural *vs*. Uyghur-Rural	0.702	0.756	0.970	0.717	0.928	0.447	0.414	0.613
		Kazakh-Rural *vs*. Zhuang-Rural	0.808	0.756	0.823	0.435	0.570	0.387	0.490	0.463
		Mongol-Rural *vs*. Tibetan-Rural	0.725	0.409	0.945	0.888	0.403	0.495	0.176	0.648
		Mongol-Rural *vs*. Uyghur-Rural	0.794	0.798	0.717	0.666	0.806	0.284	0.727	0.765
		Mongol-Rural *vs*. Zhuang-Rural	0.940	0.411	0.882	0.859	0.413	0.587	0.406	0.979
		Tibetan-Rural *vs*. Uyghur-Rural	0.616	0.554	0.766	0.766	0.504	0.693	0.907	0.932
		Tibetan-Rural *vs*. Zhuang-Rural	0.680	0.900	0.828	0.951	0.879	0.918	0.390	0.701
		Uyghur-Rural *vs*. Zhuang-Rural	0.836	0.573	0.870	0.740	0.562	0.593	0.914	0.788
	(%) With significant differences		0	0	0	0	0	0	0	0
	Family	Bai-Rural *vs*. Han-Rural	0.762	0.731	0.542	0.383	0.720	0.378	0.387	0.728
		Bai-Rural *vs*. Kazakh-Rural	0.739	0.795	0.955	0.562	0.624	0.467	0.474	0.439
		Bai-Rural *vs*. Mongol-Rural	0.728	0.272	0.661	0.682	0.313	0.644	0.210	0.936
		Bai-Rural *vs*. Tibetan-Rural	0.842	0.876	0.742	0.720	0.921	0.806	0.688	0.995
		Bai-Rural *vs*. Uyghur-Rural	0.810	0.711	0.891	0.788	0.560	0.498	0.622	0.567
		Bai-Rural *vs*. Zhuang-Rural	0.760	0.955	0.709	0.863	0.884	0.671	0.664	0.787
		Han-Rural *vs*. Kazakh-Rural	0.854	0.977	0.639	0.331	0.842	0.232	0.220	0.204
		Han-Rural *vs*. Mongol-Rural	0.915	0.349	0.995	0.830	0.439	0.510	0.840	0.633
		Han-Rural *vs*. Tibetan-Rural	0.927	0.553	0.855	0.723	0.627	0.452	0.282	0.702
		Han-Rural *vs*. Uyghur-Rural	0.980	0.960	0.835	0.564	0.768	0.227	0.274	0.309
		Han-Rural *vs*. Zhuang-Rural	0.987	0.756	0.890	0.593	0.824	0.470	0.206	0.444
		Kazakh-Rural *vs*. Mongol-Rural	0.928	0.369	0.685	0.373	0.519	0.306	0.155	0.378
		Kazakh-Rural *vs*. Tibetan-Rural	0.798	0.643	0.739	0.363	0.510	0.400	0.457	0.310
		Kazakh-Rural *vs*. Uyghur-Rural	0.873	0.947	0.829	0.717	0.927	0.944	0.591	0.763

		Kazakh-Rural *vs.* Zhuang-Rural	0.856	0.807	0.707	0.376	0.697	0.283	0.421	0.369
		Mongol-Rural *vs.* Tibetan-Rural	0.874	0.205	0.902	0.963	0.308	0.814	0.179	0.940
		Mongol-Rural *vs.* Uyghur-Rural	0.962	0.344	0.856	0.632	0.525	0.308	0.186	0.495
		Mongol-Rural *vs.* Zhuang-Rural	0.941	0.266	0.932	0.835	0.383	0.972	0.171	0.813
		Tibetan-Rural *vs.* Uyghur-Rural	0.908	0.586	0.917	0.632	0.461	0.475	0.765	0.521
		Tibetan-Rural *vs.* Zhuang-Rural	0.918	0.782	0.971	0.857	0.777	0.790	0.898	0.694
		Uyghur-Rural *vs.* Zhuang-Rural	0.980	0.711	0.888	0.684	0.593	0.312	0.695	0.555
	(%) With significant differences		0	0	0	0	0	0	0	0
		Bai-Rural *vs.* Han-Rural	0.981	0.803	0.793	0.540	0.786	0.317	0.298	0.550
		Bai-Rural *vs.* Kazakh-Rural	0.933	0.861	0.878	0.568	0.703	0.442	0.506	0.432
		Bai-Rural *vs.* Mongol-Rural	0.829	0.271	0.754	0.780	0.306	0.382	0.218	0.945
		Bai-Rural *vs.* Tibetan-Rural	0.955	0.709	1.000	0.957	0.708	0.897	0.566	0.880
		Bai-Rural *vs.* Uyghur-Rural	0.911	0.726	0.909	0.849	0.586	0.927	0.863	0.702
		Bai-Rural *vs.* Zhuang-Rural	0.955	0.974	0.870	0.995	0.947	0.806	0.620	0.891
		Han-Rural *vs.* Kazakh-Rural	0.931	0.985	0.739	0.436	0.834	0.191	0.236	0.204
		Han-Rural *vs.* Mongol-Rural	0.788	0.269	0.886	0.882	0.307	0.492	0.848	0.597
		Han-Rural *vs.* Tibetan-Rural	0.926	0.477	0.785	0.606	0.531	0.316	0.225	0.651
		Han-Rural *vs.* Uyghur-Rural	0.912	0.874	0.969	0.717	0.693	0.243	0.433	0.475
	Genus	Han-Rural *vs.* Zhuang-Rural	0.956	0.746	0.917	0.594	0.841	0.344	0.263	0.480
		Kazakh-Rural *vs.* Mongol-Rural	0.907	0.305	0.634	0.445	0.390	0.203	0.156	0.384
		Kazakh-Rural *vs.* Tibetan-Rural	0.891	0.556	0.881	0.540	0.429	0.560	0.669	0.323
		Kazakh-Rural *vs.* Uyghur-Rural	0.986	0.849	0.783	0.695	0.887	0.275	0.309	0.582
		Kazakh-Rural *vs.* Zhuang-Rural	0.955	0.799	0.790	0.510	0.688	0.323	0.448	0.391
		Mongol-Rural *vs.* Tibetan-Rural	0.789	0.152	0.764	0.859	0.194	0.323	0.125	0.935
		Mongol-Rural *vs.* Uyghur-Rural	0.934	0.351	0.884	0.753	0.437	0.272	0.293	0.698
		Mongol-Rural *vs.* Zhuang-Rural	0.852	0.226	0.836	0.798	0.282	0.425	0.121	0.831
		Tibetan-Rural *vs.* Uyghur-Rural	0.864	0.433	0.872	0.815	0.351	0.991	0.968	0.606
		Tibetan-Rural *vs.* Zhuang-Rural	0.889	0.670	0.865	0.962	0.642	0.684	0.828	0.710
		Uyghur-Rural *vs.* Zhuang-Rural	0.932	0.616	0.969	0.803	0.538	0.713	0.891	0.638
	(%) With significant differences		0	0	0	0	0	0	0	0
		Bai-Rural *vs.* Han-Rural	0.047	0.987	0.521	0.401	0.938	0.036	0.045	0.003
		Bai-Rural *vs.* Kazakh-Rural	0.183	0.919	0.277	0.411	0.973	0.669	0.664	0.474
		Bai-Rural *vs.* Mongol-Rural	0.282	0.312	0.728	0.737	0.387	0.653	0.054	0.077
		Bai-Rural *vs.* Tibetan-Rural	0.053	0.584	0.415	0.837	0.614	0.611	0.101	0.029
		Bai-Rural *vs.* Uyghur-Rural	0.192	0.936	0.579	0.835	0.720	0.919	0.201	0.338
		Bai-Rural *vs.* Zhuang-Rural	0.048	0.899	0.450	0.846	0.915	0.406	0.109	0.036
		Han-Rural *vs.* Kazakh-Rural	0.912	0.936	0.488	0.293	0.964	0.018	0.169	0.448
		Han-Rural *vs.* Mongol-Rural	0.682	0.238	0.929	0.796	0.372	0.035	0.488	0.414
		Han-Rural *vs.* Tibetan-Rural	0.997	0.588	0.787	0.567	0.605	0.039	0.130	0.617
		Han-Rural *vs.* Uyghur-Rural	0.971	0.964	0.879	0.668	0.812	0.030	0.212	0.565
	Species	Han-Rural *vs.* Zhuang-Rural	0.932	0.903	0.881	0.611	0.874	0.039	0.177	0.564
		Kazakh-Rural *vs.* Mongol-Rural	0.660	0.204	0.459	0.340	0.310	0.444	0.207	0.637
		Kazakh-Rural *vs.* Tibetan-Rural	0.908	0.723	0.573	0.371	0.662	0.546	0.692	0.503
		Kazakh-Rural *vs.* Uyghur-Rural	0.932	0.885	0.557	0.532	0.736	0.409	0.418	0.796
		Kazakh-Rural *vs.* Zhuang-Rural	0.865	0.981	0.509	0.325	0.908	0.275	0.426	0.516
		Mongol-Rural *vs.* Tibetan-Rural	0.728	0.155	0.816	0.889	0.239	0.897	0.216	0.813
		Mongol-Rural *vs.* Uyghur-Rural	0.705	0.234	0.819	0.735	0.398	0.845	0.382	0.792
		Mongol-Rural *vs.* Zhuang-Rural	0.748	0.198	0.841	0.899	0.293	0.616	0.279	0.800
		Tibetan-Rural *vs.* Uyghur-Rural	0.974	0.600	0.984	0.791	0.453	0.919	0.946	0.591
		Tibetan-Rural *vs.* Zhuang-Rural	0.923	0.690	0.922	0.997	0.689	0.589	0.621	0.937
		Uyghur-Rural *vs.* Zhuang-Rural	0.918	0.865	0.968	0.743	0.655	0.473	0.792	0.681
	(%) With significant differences		9.5% (2/21)	0	0	0	0	28.6% (6/21)	4.8% (1/21)	14.3% (3/21)
$q = 1$	Phylum	Bai-Rural *vs.* Han-Rural	0.745	0.859	0.712	0.681	0.805	0.385	0.860	0.776
		Bai-Rural *vs.* Kazakh-Rural	0.963	0.580	0.984	0.938	0.613	0.431	0.733	0.885
		Bai-Rural *vs.* Mongol-Rural	0.662	0.320	0.723	0.790	0.402	0.028	0.264	0.766
		Bai-Rural *vs.* Tibetan-Rural	0.996	0.518	0.956	0.936	0.558	0.758	0.271	0.998

	Bai-Rural *vs.* Uyghur-Rural	0.534	0.565	0.577	0.554	0.440	0.450	0.043	0.760
	Bai-Rural *vs.* Zhuang-Rural	0.644	0.688	0.660	0.744	0.694	0.564	0.895	0.685
	Han-Rural *vs.* Kazakh-Rural	0.872	0.714	0.830	0.878	0.786	0.203	0.629	0.889
	Han-Rural *vs.* Mongol-Rural	0.897	0.410	0.990	0.939	0.556	0.030	0.227	0.970
	Han-Rural *vs.* Tibetan-Rural	0.757	0.625	0.776	0.774	0.729	0.353	0.194	0.780
	Han-Rural *vs.* Uyghur-Rural	0.374	0.607	0.416	0.462	0.510	0.207	0.019	0.596
	Han-Rural *vs.* Zhuang-Rural	0.853	0.812	0.948	0.976	0.897	0.509	0.970	0.942
	Kazakh-Rural *vs.* Mongol-Rural	0.745	0.605	0.771	0.834	0.604	0.015	0.626	0.799
	Kazakh-Rural *vs.* Tibetan-Rural	0.934	0.938	0.989	0.912	0.914	0.654	0.638	0.879
	Kazakh-Rural *vs.* Uyghur-Rural	0.572	0.867	0.641	0.665	0.700	0.972	0.180	0.706
	Kazakh-Rural *vs.* Zhuang-Rural	0.747	0.850	0.766	0.840	0.865	0.186	0.577	0.751
	Mongol-Rural *vs.* Tibetan-Rural	0.612	0.708	0.710	0.822	0.786	0.019	0.742	0.695
	Mongol-Rural *vs.* Uyghur-Rural	0.188	0.899	0.284	0.340	0.792	0.008	0.045	0.409
	Mongol-Rural *vs.* Zhuang-Rural	0.954	0.490	0.946	0.975	0.546	0.022	0.266	0.964
	Tibetan-Rural *vs.* Uyghur-Rural	0.469	0.875	0.482	0.460	0.712	0.639	0.152	0.728
	Tibetan-Rural *vs.* Zhuang-Rural	0.620	0.776	0.694	0.828	0.761	0.421	0.171	0.693
	Uyghur-Rural *vs.* Zhuang-Rural	0.306	0.699	0.378	0.456	0.518	0.195	0.010	0.511
(%) With significant differences		0	0	0	0	0	28.6% (6/21)	19.0% (4/21)	0
	Bai-Rural *vs.* Han-Rural	0.958	0.931	0.979	0.836	0.991	0.596	0.645	0.926
	Bai-Rural *vs.* Kazakh-Rural	0.622	0.977	0.509	0.425	0.912	0.242	0.879	0.961
	Bai-Rural *vs.* Mongol-Rural	0.789	0.415	0.724	0.811	0.412	0.289	0.209	0.667
	Bai-Rural *vs.* Tibetan-Rural	0.684	0.672	0.761	0.799	0.726	0.871	0.896	0.772
	Bai-Rural *vs.* Uyghur-Rural	0.568	0.618	0.576	0.550	0.560	0.299	0.204	0.811
	Bai-Rural *vs.* Zhuang-Rural	0.963	0.938	0.847	0.757	0.993	0.463	0.864	0.972
	Han-Rural *vs.* Kazakh-Rural	0.589	0.902	0.471	0.347	0.905	0.231	0.956	0.902
	Han-Rural *vs.* Mongol-Rural	0.789	0.429	0.714	0.896	0.389	0.252	0.222	0.623
	Han-Rural *vs.* Tibetan-Rural	0.607	0.587	0.689	0.609	0.705	0.522	0.774	0.787
	Han-Rural *vs.* Uyghur-Rural	0.524	0.631	0.552	0.510	0.573	0.278	0.222	0.879
Family	Han-Rural *vs.* Zhuang-Rural	0.892	0.988	0.800	0.585	0.992	0.349	0.754	0.971
	Kazakh-Rural *vs.* Mongol-Rural	0.523	0.426	0.420	0.379	0.439	0.765	0.450	0.734
	Kazakh-Rural *vs.* Tibetan-Rural	0.902	0.765	0.711	0.505	0.700	0.229	0.940	0.737
	Kazakh-Rural *vs.* Uyghur-Rural	0.924	0.568	0.919	0.851	0.588	0.463	0.341	0.764
	Kazakh-Rural *vs.* Zhuang-Rural	0.610	0.918	0.497	0.373	0.927	0.495	0.899	0.937
	Mongol-Rural *vs.* Tibetan-Rural	0.530	0.271	0.573	0.703	0.326	0.295	0.241	0.506
	Mongol-Rural *vs.* Uyghur-Rural	0.378	0.684	0.401	0.463	0.633	0.985	0.659	0.449
	Mongol-Rural *vs.* Zhuang-Rural	0.733	0.392	0.603	0.631	0.335	0.606	0.184	0.638
	Tibetan-Rural *vs.* Uyghur-Rural	0.853	0.467	0.816	0.658	0.463	0.299	0.263	0.951
	Tibetan-Rural *vs.* Zhuang-Rural	0.684	0.601	0.890	0.962	0.706	0.504	0.980	0.757
	Uyghur-Rural *vs.* Zhuang-Rural	0.544	0.633	0.620	0.537	0.546	0.647	0.134	0.827
(%) With significant differences		0	0	0	0	0	0	0	0
Genus	Bai-Rural *vs.* Han-Rural	0.828	0.910	0.856	0.662	0.977	0.408	0.895	0.965
	Bai-Rural *vs.* Kazakh-Rural	0.550	0.890	0.735	0.667	0.927	0.323	0.870	0.962
	Bai-Rural *vs.* Mongol-Rural	0.816	0.641	0.776	0.879	0.604	0.429	0.690	0.719
	Bai-Rural *vs.* Tibetan-Rural	0.719	0.378	0.754	0.756	0.468	0.578	0.191	0.831
	Bai-Rural *vs.* Uyghur-Rural	0.543	0.898	0.670	0.553	0.724	0.325	0.674	0.982
	Bai-Rural *vs.* Zhuang-Rural	0.998	0.745	0.950	0.821	0.786	0.517	0.295	0.920
	Han-Rural *vs.* Kazakh-Rural	0.488	0.833	0.602	0.563	0.889	0.206	0.760	0.909
	Han-Rural *vs.* Mongol-Rural	0.904	0.666	0.853	0.856	0.575	0.250	0.694	0.638
	Han-Rural *vs.* Tibetan-Rural	0.565	0.284	0.587	0.455	0.417	0.308	0.059	0.865
	Han-Rural *vs.* Uyghur-Rural	0.472	0.951	0.598	0.505	0.672	0.190	0.732	0.996
	Han-Rural *vs.* Zhuang-Rural	0.848	0.608	0.824	0.550	0.785	0.294	0.171	0.879
	Kazakh-Rural *vs.* Mongol-Rural	0.465	0.542	0.596	0.628	0.615	0.714	0.606	0.718
	Kazakh-Rural *vs.* Tibetan-Rural	0.854	0.536	0.958	0.820	0.466	0.620	0.469	0.829
	Kazakh-Rural *vs.* Uyghur-Rural	0.993	0.812	0.940	0.890	0.791	0.936	0.456	0.920
	Kazakh-Rural *vs.* Zhuang-Rural	0.566	0.873	0.751	0.695	0.720	0.667	0.601	0.982

		Mongol-Rural *vs.* Tibetan-Rural	0.644	0.274	0.661	0.729	0.322	0.730	0.106	0.648
		Mongol-Rural *vs.* Uyghur-Rural	0.439	0.735	0.577	0.552	0.832	0.677	0.895	0.682
		Mongol-Rural *vs.* Zhuang-Rural	0.817	0.463	0.783	0.763	0.504	0.874	0.170	0.786
		Tibetan-Rural *vs.* Uyghur-Rural	0.825	0.467	0.929	0.758	0.378	0.580	0.208	0.893
		Tibetan-Rural *vs.* Zhuang-Rural	0.736	0.516	0.780	0.913	0.566	0.873	0.570	0.785
		Uyghur-Rural *vs.* Zhuang-Rural	0.561	0.656	0.679	0.612	0.516	0.608	0.226	0.906
	(%) With significant differences		0	0	0	0	0	0	0	0
		Bai-Rural *vs.* Han-Rural	0.677	0.820	0.756	0.902	0.965	0.525	0.074	0.538
		Bai-Rural *vs.* Kazakh-Rural	0.426	0.853	0.410	0.413	0.926	0.409	0.849	0.778
		Bai-Rural *vs.* Mongol-Rural	0.952	0.329	0.941	0.927	0.383	0.597	0.035	0.931
		Bai-Rural *vs.* Tibetan-Rural	0.442	0.637	0.567	0.741	0.616	0.341	0.416	0.480
		Bai-Rural *vs.* Uyghur-Rural	0.407	0.750	0.562	0.625	0.745	0.484	0.221	0.681
		Bai-Rural *vs.* Zhuang-Rural	0.626	0.988	0.700	0.797	0.900	0.955	0.158	0.570
		Han-Rural *vs.* Kazakh-Rural	0.667	0.746	0.634	0.497	0.964	0.303	0.385	0.850
		Han-Rural *vs.* Mongol-Rural	0.774	0.429	0.718	0.984	0.384	0.387	0.387	0.620
		Han-Rural *vs.* Tibetan-Rural	0.663	0.523	0.770	0.657	0.671	0.770	0.265	0.861
		Han-Rural *vs.* Uyghur-Rural	0.588	0.920	0.699	0.664	0.735	0.313	0.989	0.946
	Species	Han-Rural *vs.* Zhuang-Rural	0.944	0.860	0.970	0.731	0.976	0.617	0.235	0.938
		Kazakh-Rural *vs.* Mongol-Rural	0.526	0.283	0.475	0.466	0.309	0.646	0.171	0.774
		Kazakh-Rural *vs.* Tibetan-Rural	0.922	0.832	0.824	0.633	0.728	0.160	0.782	0.743
		Kazakh-Rural *vs.* Uyghur-Rural	0.932	0.650	0.917	0.844	0.687	0.813	0.304	0.860
		Kazakh-Rural *vs.* Zhuang-Rural	0.631	0.825	0.570	0.418	0.999	0.413	0.662	0.866
		Mongol-Rural *vs.* Tibetan-Rural	0.590	0.261	0.661	0.787	0.297	0.181	0.144	0.560
		Mongol-Rural *vs.* Uyghur-Rural	0.407	0.438	0.512	0.598	0.440	0.742	0.573	0.611
		Mongol-Rural *vs.* Zhuang-Rural	0.736	0.303	0.715	0.785	0.334	0.551	0.088	0.658
		Tibetan-Rural *vs.* Uyghur-Rural	0.884	0.557	0.918	0.807	0.496	0.157	0.565	0.840
		Tibetan-Rural *vs.* Zhuang-Rural	0.708	0.618	0.783	0.929	0.654	0.344	0.992	0.791
		Uyghur-Rural *vs.* Zhuang-Rural	0.564	0.774	0.728	0.642	0.676	0.473	0.373	0.986
	(%) With significant differences		0	0	0	0	0	0	4.8% (1/21)	0
$q = 2$		Bai-Rural *vs.* Han-Rural	0.755	0.902	0.757	0.728	0.883	0.324	0.747	0.836
		Bai-Rural *vs.* Kazakh-Rural	0.887	0.724	0.915	0.949	0.816	0.304	0.934	0.903
		Bai-Rural *vs.* Mongol-Rural	0.818	0.420	0.832	0.862	0.482	0.770	0.228	0.896
		Bai-Rural *vs.* Tibetan-Rural	0.890	0.460	0.953	0.985	0.512	0.498	0.065	0.923
		Bai-Rural *vs.* Uyghur-Rural	0.831	0.401	0.724	0.656	0.391	0.256	0.026	0.922
		Bai-Rural *vs.* Zhuang-Rural	0.709	0.749	0.741	0.843	0.757	0.379	0.917	0.721
		Han-Rural *vs.* Kazakh-Rural	0.961	0.835	0.891	0.997	0.946	0.163	0.749	0.925
		Han-Rural *vs.* Mongol-Rural	0.937	0.548	0.911	0.904	0.631	0.257	0.210	0.923
		Han-Rural *vs.* Tibetan-Rural	0.685	0.521	0.749	0.802	0.619	0.239	0.067	0.759
		Han-Rural *vs.* Uyghur-Rural	0.653	0.413	0.591	0.581	0.407	0.175	0.020	0.770
	Phylum	Han-Rural *vs.* Zhuang-Rural	0.910	0.855	0.997	0.943	0.869	0.222	0.857	0.920
		Kazakh-Rural *vs.* Mongol-Rural	1.000	0.648	0.957	0.980	0.557	0.398	0.426	0.995
		Kazakh-Rural *vs.* Tibetan-Rural	0.792	0.669	0.901	0.943	0.612	0.794	0.222	0.760
		Kazakh-Rural *vs.* Uyghur-Rural	0.810	0.633	0.761	0.731	0.543	0.404	0.137	0.852
		Kazakh-Rural *vs.* Zhuang-Rural	0.887	0.961	0.876	0.976	0.928	0.945	0.847	0.791
		Mongol-Rural *vs.* Tibetan-Rural	0.658	0.883	0.739	0.866	0.878	0.579	0.324	0.739
		Mongol-Rural *vs.* Uyghur-Rural	0.613	0.624	0.533	0.528	0.568	0.259	0.041	0.740
		Mongol-Rural *vs.* Zhuang-Rural	0.793	0.612	0.903	0.979	0.641	0.462	0.340	0.778
		Tibetan-Rural *vs.* Uyghur-Rural	0.879	0.752	0.740	0.630	0.722	0.535	0.279	0.993
		Tibetan-Rural *vs.* Zhuang-Rural	0.623	0.640	0.740	0.882	0.644	0.784	0.096	0.671
		Uyghur-Rural *vs.* Zhuang-Rural	0.634	0.466	0.578	0.614	0.423	0.588	0.018	0.662
	(%) With significant differences		0	0	0	0	0	0	19.0% (4/21)	0
	Family	Bai-Rural *vs.* Han-Rural	0.984	0.966	0.991	0.875	0.995	0.518	0.751	0.899
		Bai-Rural *vs.* Kazakh-Rural	0.755	0.900	0.587	0.476	0.947	0.866	0.924	0.855
		Bai-Rural *vs.* Mongol-Rural	0.780	0.524	0.798	0.885	0.548	0.538	0.541	0.716
		Bai-Rural *vs.* Tibetan-Rural	0.726	0.653	0.733	0.693	0.705	0.886	0.981	0.797

	Bai-Rural *vs.* Uyghur-Rural	0.473	0.710	0.489	0.532	0.691	0.377	0.247	0.645
	Bai-Rural *vs.* Zhuang-Rural	0.819	0.996	0.776	0.765	0.947	0.909	0.714	0.805
	Han-Rural *vs.* Kazakh-Rural	0.724	0.813	0.493	0.388	0.921	0.506	0.928	0.755
	Han-Rural *vs.* Mongol-Rural	0.710	0.505	0.777	0.948	0.506	0.355	0.568	0.625
	Han-Rural *vs.* Tibetan-Rural	0.685	0.587	0.664	0.523	0.696	0.597	0.671	0.875
	Han-Rural *vs.* Uyghur-Rural	0.392	0.699	0.446	0.471	0.671	0.293	0.201	0.739
	Han-Rural *vs.* Zhuang-Rural	0.746	0.944	0.715	0.596	0.922	0.492	0.939	0.866
	Kazakh-Rural *vs.* Mongol-Rural	0.634	0.496	0.505	0.456	0.522	0.601	0.734	0.855
	Kazakh-Rural *vs.* Tibetan-Rural	0.948	0.831	0.744	0.565	0.802	0.831	0.921	0.700
	Kazakh-Rural *vs.* Uyghur-Rural	0.749	0.638	0.952	0.924	0.662	0.197	0.454	0.583
	Kazakh-Rural *vs.* Zhuang-Rural	0.850	0.876	0.581	0.416	0.993	0.989	0.891	0.707
	Mongol-Rural *vs.* Tibetan-Rural	0.547	0.316	0.586	0.675	0.406	0.519	0.422	0.554
	Mongol-Rural *vs.* Uyghur-Rural	0.316	0.727	0.388	0.494	0.718	0.722	0.512	0.425
	Mongol-Rural *vs.* Zhuang-Rural	0.566	0.432	0.594	0.677	0.422	0.636	0.564	0.542
	Tibetan-Rural *vs.* Uyghur-Rural	0.709	0.509	0.720	0.653	0.520	0.379	0.243	0.869
	Tibetan-Rural *vs.* Zhuang-Rural	0.870	0.634	0.901	0.914	0.752	0.798	0.663	0.978
	Uyghur-Rural *vs.* Zhuang-Rural	0.487	0.696	0.542	0.502	0.648	0.478	0.164	0.824
(%) With significant differences		0	0	0	0	0	0	0	0
	Bai-Rural *vs.* Han-Rural	0.889	0.940	0.878	0.670	0.853	0.979	0.784	0.963
	Bai-Rural *vs.* Kazakh-Rural	0.470	0.861	0.687	0.681	0.970	0.170	0.950	0.986
	Bai-Rural *vs.* Mongol-Rural	0.886	0.826	0.803	0.896	0.741	0.229	0.989	0.741
	Bai-Rural *vs.* Tibetan-Rural	0.819	0.288	0.856	0.812	0.409	0.301	0.086	0.951
	Bai-Rural *vs.* Uyghur-Rural	0.434	0.992	0.518	0.502	0.836	0.268	0.522	0.830
	Bai-Rural *vs.* Zhuang-Rural	0.897	0.638	0.980	0.894	0.792	0.264	0.341	0.886
	Han-Rural *vs.* Kazakh-Rural	0.414	0.914	0.611	0.635	0.818	0.234	0.960	0.959
	Han-Rural *vs.* Mongol-Rural	0.964	0.770	0.875	0.839	0.623	0.326	0.809	0.657
	Han-Rural *vs.* Tibetan-Rural	0.711	0.262	0.753	0.533	0.423	0.367	0.039	0.898
	Han-Rural *vs.* Uyghur-Rural	0.384	0.929	0.475	0.460	0.716	0.463	0.377	0.880
Genus	Han-Rural *vs.* Zhuang-Rural	0.775	0.640	0.876	0.625	0.892	0.341	0.333	0.807
	Kazakh-Rural *vs.* Mongol-Rural	0.443	0.698	0.600	0.668	0.738	0.562	0.939	0.719
	Kazakh-Rural *vs.* Tibetan-Rural	0.763	0.466	0.897	0.876	0.470	0.515	0.261	0.975
	Kazakh-Rural *vs.* Uyghur-Rural	0.984	0.868	0.827	0.806	0.873	0.206	0.494	0.845
	Kazakh-Rural *vs.* Zhuang-Rural	0.550	0.792	0.742	0.776	0.755	0.464	0.533	0.929
	Mongol-Rural *vs.* Tibetan-Rural	0.779	0.284	0.778	0.766	0.356	0.866	0.080	0.829
	Mongol-Rural *vs.* Uyghur-Rural	0.417	0.854	0.498	0.500	0.901	0.673	0.476	0.623
	Mongol-Rural *vs.* Zhuang-Rural	0.802	0.540	0.826	0.835	0.607	0.867	0.301	0.852
	Tibetan-Rural *vs.* Uyghur-Rural	0.723	0.430	0.779	0.722	0.420	0.812	0.082	0.836
	Tibetan-Rural *vs.* Zhuang-Rural	0.898	0.474	0.880	0.914	0.512	0.967	0.285	0.973
	Uyghur-Rural *vs.* Zhuang-Rural	0.527	0.662	0.582	0.565	0.638	0.826	0.190	0.760
(%) With significant differences		0	0	0	0	0	0	4.8% (1/21)	0
Species	Bai-Rural *vs.* Han-Rural	0.675	0.939	0.729	0.977	0.780	0.910	0.283	0.632
	Bai-Rural *vs.* Kazakh-Rural	0.338	0.692	0.426	0.514	0.782	0.244	0.842	0.751
	Bai-Rural *vs.* Mongol-Rural	0.877	0.496	0.981	0.994	0.469	0.271	0.047	0.943
	Bai-Rural *vs.* Tibetan-Rural	0.545	0.455	0.652	0.722	0.544	0.428	0.844	0.761
	Bai-Rural *vs.* Uyghur-Rural	0.148	0.724	0.381	0.545	0.925	0.247	0.191	0.529
	Bai-Rural *vs.* Zhuang-Rural	0.667	0.888	0.637	0.710	0.727	0.512	0.387	0.590
	Han-Rural *vs.* Kazakh-Rural	0.623	0.853	0.712	0.638	0.977	0.337	0.636	0.914
	Han-Rural *vs.* Mongol-Rural	0.838	0.526	0.780	0.967	0.433	0.374	0.338	0.679
	Han-Rural *vs.* Tibetan-Rural	0.811	0.543	0.878	0.669	0.749	0.549	0.272	0.885
	Han-Rural *vs.* Uyghur-Rural	0.341	0.843	0.587	0.637	0.901	0.367	0.683	0.863
	Han-Rural *vs.* Zhuang-Rural	0.985	0.967	0.944	0.757	0.974	0.713	0.462	0.996
	Kazakh-Rural *vs.* Mongol-Rural	0.494	0.365	0.543	0.599	0.367	0.085	0.264	0.748
	Kazakh-Rural *vs.* Tibetan-Rural	0.815	0.747	0.818	0.776	0.734	0.570	0.803	0.996
	Kazakh-Rural *vs.* Uyghur-Rural	0.636	0.993	0.908	0.987	0.895	0.974	0.412	0.806
	Kazakh-Rural *vs.* Zhuang-Rural	0.489	0.782	0.664	0.581	0.946	0.190	0.926	0.919
	Mongol-Rural *vs.* Tibetan-Rural	0.743	0.292	0.740	0.794	0.337	0.149	0.115	0.809

		Mongol-Rural *vs.* Uyghur-Rural	0.217	0.345	0.421	0.572	0.395	0.073	0.710	0.538
		Mongol-Rural *vs.* Zhuang-Rural	0.857	0.436	0.695	0.798	0.350	0.430	0.078	0.674
		Tibetan-Rural *vs.* Uyghur-Rural	0.484	0.733	0.718	0.751	0.671	0.549	0.289	0.792
		Tibetan-Rural *vs.* Zhuang-Rural	0.807	0.518	0.931	0.928	0.752	0.274	0.461	0.892
		Uyghur-Rural *vs.* Zhuang-Rural	0.251	0.852	0.553	0.569	0.872	0.158	0.190	0.845
	(%) With significant differences		0	0	0	0	0	0	4.8% (1/21)	0
q = 3		Bai-Rural *vs.* Han-Rural	0.783	0.927	0.749	0.756	0.871	0.591	0.704	0.763
		Bai-Rural *vs.* Kazakh-Rural	0.859	0.772	0.849	0.874	0.803	0.245	0.975	0.820
		Bai-Rural *vs.* Mongol-Rural	0.864	0.459	0.882	0.888	0.517	0.781	0.199	0.945
		Bai-Rural *vs.* Tibetan-Rural	0.768	0.474	0.909	0.947	0.492	0.739	0.029	0.881
		Bai-Rural *vs.* Uyghur-Rural	0.988	0.336	0.923	0.817	0.340	0.318	0.017	0.836
		Bai-Rural *vs.* Zhuang-Rural	0.755	0.770	0.763	0.875	0.745	0.450	0.912	0.683
		Han-Rural *vs.* Kazakh-Rural	1.000	0.850	0.978	0.929	0.954	0.211	0.740	0.967
		Han-Rural *vs.* Mongol-Rural	0.918	0.565	0.860	0.886	0.681	0.472	0.171	0.843
		Han-Rural *vs.* Tibetan-Rural	0.614	0.522	0.705	0.771	0.575	0.486	0.030	0.703
		Han-Rural *vs.* Uyghur-Rural	0.886	0.326	0.775	0.737	0.362	0.279	0.019	0.999
	Phylum	Han-Rural *vs.* Zhuang-Rural	0.937	0.873	0.988	0.925	0.882	0.344	0.787	0.885
		Kazakh-Rural *vs.* Mongol-Rural	0.939	0.662	0.909	0.882	0.636	0.310	0.366	0.817
		Kazakh-Rural *vs.* Tibetan-Rural	0.711	0.637	0.807	0.884	0.581	0.294	0.155	0.697
		Kazakh-Rural *vs.* Uyghur-Rural	0.922	0.555	0.848	0.819	0.516	0.577	0.129	0.980
		Kazakh-Rural *vs.* Zhuang-Rural	0.952	0.982	0.988	0.879	0.932	0.505	0.916	0.822
		Mongol-Rural *vs.* Tibetan-Rural	0.610	0.828	0.745	0.870	0.760	0.951	0.197	0.758
		Mongol-Rural *vs.* Uyghur-Rural	0.897	0.477	0.785	0.747	0.462	0.335	0.054	0.824
		Mongol-Rural *vs.* Zhuang-Rural	0.819	0.630	0.866	0.979	0.712	0.558	0.345	0.671
		Tibetan-Rural *vs.* Uyghur-Rural	0.853	0.677	0.967	0.816	0.731	0.427	0.569	0.765
		Tibetan-Rural *vs.* Zhuang-Rural	0.596	0.645	0.736	0.889	0.641	0.597	0.080	0.604
		Uyghur-Rural *vs.* Zhuang-Rural	0.864	0.375	0.770	0.780	0.384	0.667	0.028	0.895
	(%) With significant differences		0	0	0	0	0	0	23.8% (5/21)	0
		Bai-Rural *vs.* Han-Rural	0.835	1.000	0.947	0.880	0.995	0.564	0.648	0.759
		Bai-Rural *vs.* Kazakh-Rural	0.889	0.899	0.677	0.556	0.875	0.549	0.799	0.993
		Bai-Rural *vs.* Mongol-Rural	0.830	0.593	0.851	0.900	0.629	0.897	0.635	0.802
		Bai-Rural *vs.* Tibetan-Rural	0.680	0.608	0.694	0.664	0.681	0.512	0.905	0.753
		Bai-Rural *vs.* Uyghur-Rural	0.349	0.866	0.374	0.480	0.858	0.489	0.220	0.446
		Bai-Rural *vs.* Zhuang-Rural	0.663	0.956	0.700	0.808	0.890	0.855	0.558	0.602
		Han-Rural *vs.* Kazakh-Rural	0.972	0.862	0.585	0.453	0.855	0.901	0.957	0.771
		Han-Rural *vs.* Mongol-Rural	0.662	0.547	0.793	0.970	0.588	0.530	0.805	0.608
		Han-Rural *vs.* Tibetan-Rural	0.758	0.546	0.674	0.514	0.669	0.996	0.638	0.928
		Han-Rural *vs.* Uyghur-Rural	0.328	0.827	0.332	0.406	0.830	0.389	0.218	0.624
	Family	Han-Rural *vs.* Zhuang-Rural	0.711	0.921	0.682	0.641	0.861	0.709	0.854	0.757
		Kazakh-Rural *vs.* Mongol-Rural	0.777	0.577	0.597	0.538	0.581	0.453	0.882	0.815
		Kazakh-Rural *vs.* Tibetan-Rural	0.882	0.787	0.809	0.619	0.831	0.834	0.843	0.756
		Kazakh-Rural *vs.* Uyghur-Rural	0.564	0.805	0.780	0.965	0.770	0.130	0.514	0.532
		Kazakh-Rural *vs.* Zhuang-Rural	0.865	0.911	0.720	0.487	0.974	0.785	0.870	0.657
		Mongol-Rural *vs.* Tibetan-Rural	0.546	0.332	0.613	0.672	0.449	0.467	0.586	0.577
		Mongol-Rural *vs.* Uyghur-Rural	0.247	0.664	0.338	0.461	0.689	0.489	0.455	0.357
		Mongol-Rural *vs.* Zhuang-Rural	0.481	0.462	0.594	0.727	0.480	0.775	0.901	0.464
		Tibetan-Rural *vs.* Uyghur-Rural	0.565	0.567	0.564	0.566	0.611	0.247	0.219	0.721
		Tibetan-Rural *vs.* Zhuang-Rural	1.000	0.615	0.944	0.881	0.782	0.629	0.533	0.875
		Uyghur-Rural *vs.* Zhuang-Rural	0.426	0.798	0.410	0.384	0.751	0.452	0.180	0.788
	(%) With significant differences		0	0	0	0	0	0	0	0
	Genus	Bai-Rural *vs.* Han-Rural	0.907	0.909	0.858	0.668	0.856	0.353	0.839	0.954
		Bai-Rural *vs.* Kazakh-Rural	0.449	0.875	0.764	0.801	0.902	0.596	0.908	0.996
		Bai-Rural *vs.* Mongol-Rural	0.974	0.954	0.790	0.873	0.801	0.829	0.916	0.768
		Bai-Rural *vs.* Tibetan-Rural	0.953	0.320	0.998	0.830	0.478	0.499	0.077	0.712
		Bai-Rural *vs.* Uyghur-Rural	0.381	0.967	0.550	0.556	0.827	0.405	0.573	0.859

		Bai-Rural *vs.* Zhuang-Rural	0.831	0.600	0.990	0.909	0.812	0.329	0.472	0.914
		Han-Rural *vs.* Kazakh-Rural	0.404	0.933	0.678	0.739	0.799	0.211	0.821	0.963
		Han-Rural *vs.* Mongol-Rural	0.934	0.865	0.896	0.874	0.698	0.312	0.953	0.693
		Han-Rural *vs.* Tibetan-Rural	0.999	0.318	0.907	0.580	0.498	0.260	0.049	0.631
		Han-Rural *vs.* Uyghur-Rural	0.340	0.942	0.489	0.501	0.706	0.180	0.438	0.898
		Han-Rural *vs.* Zhuang-Rural	0.742	0.618	0.870	0.671	0.897	0.734	0.474	0.848
		Kazakh-Rural *vs.* Mongol-Rural	0.494	0.819	0.696	0.772	0.844	0.557	0.848	0.752
		Kazakh-Rural *vs.* Tibetan-Rural	0.640	0.492	0.864	0.925	0.508	0.937	0.185	0.774
		Kazakh-Rural *vs.* Uyghur-Rural	0.949	0.906	0.805	0.801	0.908	0.436	0.686	0.872
		Kazakh-Rural *vs.* Zhuang-Rural	0.577	0.746	0.817	0.864	0.724	0.129	0.525	0.943
		Mongol-Rural *vs.* Tibetan-Rural	0.965	0.365	0.896	0.787	0.439	0.566	0.107	0.951
		Mongol-Rural *vs.* Uyghur-Rural	0.434	0.919	0.522	0.547	0.959	0.284	0.470	0.657
		Mongol-Rural *vs.* Zhuang-Rural	0.838	0.594	0.840	0.835	0.643	0.217	0.512	0.856
		Tibetan-Rural *vs.* Uyghur-Rural	0.574	0.479	0.725	0.763	0.458	0.718	0.069	0.691
		Tibetan-Rural *vs.* Zhuang-Rural	0.851	0.543	0.992	0.944	0.592	0.191	0.203	0.805
		Uyghur-Rural *vs.* Zhuang-Rural	0.523	0.651	0.612	0.628	0.634	0.107	0.302	0.793
	(%) With significant differences		0	0	0	0	0	0	4.8% (1/21)	0
		Bai-Rural *vs.* Han-Rural	0.632	0.766	0.734	0.973	0.691	0.733	0.449	0.621
		Bai-Rural *vs.* Kazakh-Rural	0.257	0.581	0.401	0.570	0.630	0.567	0.796	0.655
		Bai-Rural *vs.* Mongol-Rural	0.980	0.556	0.909	0.998	0.464	0.844	0.134	0.785
		Bai-Rural *vs.* Tibetan-Rural	0.680	0.423	0.736	0.770	0.502	0.935	0.619	0.911
		Bai-Rural *vs.* Uyghur-Rural	0.089	0.524	0.311	0.529	0.688	0.884	0.037	0.416
		Bai-Rural *vs.* Zhuang-Rural	0.612	0.708	0.634	0.705	0.611	0.910	0.773	0.605
		Han-Rural *vs.* Kazakh-Rural	0.591	0.902	0.714	0.701	0.984	0.486	0.798	0.980
		Han-Rural *vs.* Mongol-Rural	0.712	0.495	0.723	0.984	0.404	0.636	0.461	0.560
		Han-Rural *vs.* Tibetan-Rural	0.996	0.597	0.955	0.682	0.781	0.672	0.243	0.727
		Han-Rural *vs.* Uyghur-Rural	0.299	0.805	0.549	0.646	0.973	0.670	0.273	0.789
	Species	Han-Rural *vs.* Zhuang-Rural	0.950	0.990	0.925	0.769	0.989	0.789	0.475	0.979
		Kazakh-Rural *vs.* Mongol-Rural	0.407	0.352	0.518	0.672	0.318	0.510	0.469	0.596
		Kazakh-Rural *vs.* Tibetan-Rural	0.663	0.771	0.784	0.822	0.781	0.516	0.622	0.804
		Kazakh-Rural *vs.* Uyghur-Rural	0.606	0.919	0.865	0.929	0.988	0.429	0.274	0.768
		Kazakh-Rural *vs.* Zhuang-Rural	0.456	0.846	0.672	0.665	0.969	0.526	0.881	0.993
		Mongol-Rural *vs.* Tibetan-Rural	0.783	0.309	0.753	0.806	0.324	0.925	0.161	0.832
		Mongol-Rural *vs.* Uyghur-Rural	0.157	0.287	0.365	0.601	0.314	0.931	0.647	0.403
		Mongol-Rural *vs.* Zhuang-Rural	0.730	0.418	0.642	0.787	0.317	0.786	0.148	0.558
		Tibetan-Rural *vs.* Uyghur-Rural	0.351	0.837	0.629	0.740	0.779	0.860	0.058	0.614
		Tibetan-Rural *vs.* Zhuang-Rural	0.964	0.574	0.985	0.969	0.787	0.856	0.445	0.768
		Uyghur-Rural *vs.* Zhuang-Rural	0.179	0.773	0.486	0.591	0.969	0.813	0.044	0.759
	(%) With significant differences		0	0	0	0	0	0	9.5% (2/21)	0

Scheme-2B (Urban)

Diversity order	Taxon	Cohort	PL		PLEC					
			z	ln(*c*)	*z*	*d*	ln(*c*)	A_{max}	D_{max}	*LGD*
q = 0	Phylum	Bai-Urban *vs.* Han-Urban	0.798	0.929	0.913	0.754	0.778	0.244	0.777	0.679
		Bai-Urban *vs.* Kazakh-Urban	0.987	0.982	0.942	0.907	0.978	0.419	0.867	0.926
		Bai-Urban *vs.* Mongol-Urban	0.974	0.724	0.922	0.831	0.675	0.383	0.737	0.577
		Bai-Urban *vs.* Tibetan-Urban	0.850	0.761	0.963	0.915	0.653	0.302	0.568	0.975
		Bai-Urban *vs.* Uyghur-Urban	0.680	0.391	0.949	0.807	0.410	0.397	0.983	0.729
		Bai-Urban *vs.* Zhuang-Urban	0.888	0.909	0.923	0.933	0.847	0.850	0.668	0.611
		Han-Urban *vs.* Kazakh-Urban	0.783	0.918	0.872	0.639	0.751	0.307	0.748	0.543
		Han-Urban *vs.* Mongol-Urban	0.800	0.675	0.968	0.844	0.920	0.393	0.779	0.916
		Han-Urban *vs.* Tibetan-Urban	0.979	0.676	0.845	0.586	0.883	0.181	0.369	0.527
		Han-Urban *vs.* Uyghur-Urban	0.834	0.420	0.967	0.570	0.295	0.177	0.732	0.329
		Han-Urban *vs.* Zhuang-Urban	0.670	0.824	0.815	0.684	0.937	0.254	0.720	0.776
		Kazakh-Urban *vs.* Mongol-Urban	0.951	0.776	0.849	0.675	0.675	0.709	0.623	0.527

	Kazakh-Urban *vs.* Tibetan-Urban	0.835	0.779	0.987	0.977	0.642	0.190	0.608	0.935
	Kazakh-Urban *vs.* Uyghur-Urban	0.693	0.394	0.915	0.916	0.451	0.213	0.826	0.672
	Kazakh-Urban *vs.* Zhuang-Urban	0.910	0.926	0.990	0.799	0.837	0.764	0.574	0.590
	Mongol-Urban *vs.* Tibetan-Urban	0.840	0.986	0.891	0.677	0.936	0.232	0.354	0.557
	Mongol-Urban *vs.* Uyghur-Urban	0.685	0.254	0.987	0.662	0.250	0.230	0.793	0.363
	Mongol-Urban *vs.* Zhuang-Urban	0.844	0.821	0.827	0.844	0.843	0.581	0.974	0.911
	Tibetan-Urban *vs.* Uyghur-Urban	0.824	0.322	0.916	0.897	0.300	0.588	0.441	0.662
	Tibetan-Urban *vs.* Zhuang-Urban	0.674	0.822	0.976	0.773	0.778	0.390	0.287	0.522
	Uyghur-Urban *vs.* Zhuang-Urban	0.603	0.332	0.892	0.750	0.343	0.437	0.628	0.322
(%) With significant differences		0	0	0	0	0	0	0	0
Family	Bai-Urban *vs.* Han-Urban	0.981	0.625	0.935	0.585	0.942	0.475	0.296	0.335
	Bai-Urban *vs.* Kazakh-Urban	0.493	0.764	0.589	0.905	0.977	0.677	0.290	0.541
	Bai-Urban *vs.* Mongol-Urban	0.868	0.488	0.971	0.669	0.364	0.407	0.741	0.480
	Bai-Urban *vs.* Tibetan-Urban	0.989	0.622	0.999	0.912	0.797	0.183	0.270	0.704
	Bai-Urban *vs.* Uyghur-Urban	0.916	0.312	0.990	0.972	0.435	0.958	0.468	0.997
	Bai-Urban *vs.* Zhuang-Urban	0.857	0.953	0.953	0.689	0.710	0.181	0.258	0.407
	Han-Urban *vs.* Kazakh-Urban	0.490	0.822	0.526	0.504	0.909	0.553	0.805	0.729
	Han-Urban *vs.* Mongol-Urban	0.929	0.313	0.957	0.720	0.496	0.890	0.303	0.651
	Han-Urban *vs.* Tibetan-Urban	0.966	0.972	0.946	0.642	0.860	0.974	0.699	0.514
	Han-Urban *vs.* Uyghur-Urban	0.894	0.589	0.938	0.656	0.503	0.426	0.615	0.406
	Han-Urban *vs.* Zhuang-Urban	0.879	0.568	0.884	0.713	0.670	0.544	0.673	0.817
	Kazakh-Urban *vs.* Mongol-Urban	0.517	0.468	0.534	0.665	0.526	0.388	0.461	0.996
	Kazakh-Urban *vs.* Tibetan-Urban	0.523	0.841	0.634	0.828	0.803	0.211	0.761	0.733
	Kazakh-Urban *vs.* Uyghur-Urban	0.465	0.436	0.654	0.933	0.402	0.585	0.419	0.490
	Kazakh-Urban *vs.* Zhuang-Urban	0.562	0.767	0.625	0.653	0.773	0.178	0.958	0.812
	Mongol-Urban *vs.* Tibetan-Urban	0.901	0.324	0.973	0.817	0.331	0.712	0.499	0.690
	Mongol-Urban *vs.* Uyghur-Urban	0.850	0.225	0.972	0.800	0.276	0.241	0.922	0.551
	Mongol-Urban *vs.* Zhuang-Urban	0.955	0.582	0.892	0.988	0.674	0.410	0.373	0.849
	Tibetan-Urban *vs.* Uyghur-Urban	0.898	0.526	0.981	0.895	0.564	0.150	0.580	0.732
	Tibetan-Urban *vs.* Zhuang-Urban	0.868	0.604	0.930	0.797	0.547	0.378	0.676	0.576
	Uyghur-Urban *vs.* Zhuang-Urban	0.814	0.332	0.973	0.747	0.327	0.136	0.612	0.452
(%) With significant differences		0	0	0	0	0	0	0	0
Genus	Bai-Urban *vs.* Han-Urban	0.828	0.619	0.851	0.613	0.892	0.075	0.235	0.548
	Bai-Urban *vs.* Kazakh-Urban	0.903	0.623	0.731	0.781	0.836	0.473	0.711	1.000
	Bai-Urban *vs.* Mongol-Urban	0.830	0.542	0.768	0.700	0.533	0.752	0.969	0.790
	Bai-Urban *vs.* Tibetan-Urban	0.871	0.781	0.827	0.861	0.821	0.764	0.788	0.950
	Bai-Urban *vs.* Uyghur-Urban	0.880	0.316	0.965	0.862	0.367	0.396	0.760	0.685
	Bai-Urban *vs.* Zhuang-Urban	0.690	0.935	0.872	0.819	0.904	0.045	0.538	0.939
	Han-Urban *vs.* Kazakh-Urban	0.782	0.982	0.599	0.456	0.954	0.091	0.443	0.575
	Han-Urban *vs.* Mongol-Urban	0.959	0.385	0.973	0.807	0.586	0.108	0.235	0.609
	Han-Urban *vs.* Tibetan-Urban	0.722	0.864	0.670	0.466	0.932	0.065	0.242	0.461
	Han-Urban *vs.* Uyghur-Urban	0.972	0.558	0.832	0.566	0.457	0.070	0.385	0.383
	Han-Urban *vs.* Zhuang-Urban	0.855	0.665	0.951	0.634	0.804	0.527	0.320	0.444
	Kazakh-Urban *vs.* Mongol-Urban	0.770	0.344	0.578	0.565	0.461	0.363	0.829	0.794
	Kazakh-Urban *vs.* Tibetan-Urban	0.978	0.858	0.901	0.930	0.997	0.305	0.898	0.950
	Kazakh-Urban *vs.* Uyghur-Urban	0.786	0.560	0.777	0.954	0.446	0.893	0.940	0.634
	Kazakh-Urban *vs.* Zhuang-Urban	0.649	0.641	0.626	0.636	0.747	0.031	0.768	0.931
	Mongol-Urban *vs.* Tibetan-Urban	0.732	0.424	0.641	0.592	0.453	0.811	0.803	0.761
	Mongol-Urban *vs.* Uyghur-Urban	1.000	0.225	0.854	0.709	0.285	0.283	0.830	0.546
	Mongol-Urban *vs.* Zhuang-Urban	0.821	0.464	0.923	0.819	0.627	0.065	0.372	0.786
	Tibetan-Urban *vs.* Uyghur-Urban	0.782	0.445	0.876	0.969	0.500	0.274	0.926	0.696
	Tibetan-Urban *vs.* Zhuang-Urban	0.623	0.849	0.701	0.663	0.759	0.030	0.604	0.891
	Uyghur-Urban *vs.* Zhuang-Urban	0.906	0.362	0.892	0.755	0.351	0.028	0.776	0.664
(%) With significant differences		0	0	0	0	0	0	19.0% (4/21)	0
Species	Bai-Urban *vs.* Han-Urban	0.714	0.585	0.708	0.205	0.800	0.066	0.140	0.087

		Bai-Urban *vs.* Kazakh-Urban	0.509	0.668	0.888	0.885	0.693	0.352	0.288	0.352
		Bai-Urban *vs.* Mongol-Urban	0.599	0.633	0.658	0.252	0.658	0.095	0.137	0.101
		Bai-Urban *vs.* Tibetan-Urban	0.805	0.531	0.475	0.475	0.395	0.270	0.925	0.731
		Bai-Urban *vs.* Uyghur-Urban	0.644	0.366	0.938	0.748	0.394	0.318	0.193	0.440
		Bai-Urban *vs.* Zhuang-Urban	0.551	0.947	0.771	0.292	0.955	0.058	0.170	0.134
		Han-Urban *vs.* Kazakh-Urban	0.701	0.901	0.582	0.365	0.903	0.066	0.312	0.447
		Han-Urban *vs.* Mongol-Urban	0.925	0.316	0.954	0.822	0.518	0.098	0.293	0.705
		Han-Urban *vs.* Tibetan-Urban	0.499	0.981	0.538	0.975	0.622	0.058	0.108	0.018
		Han-Urban *vs.* Uyghur-Urban	0.849	0.650	0.788	0.560	0.508	0.056	0.548	0.465
		Han-Urban *vs.* Zhuang-Urban	0.856	0.530	0.923	0.692	0.759	0.150	0.348	0.654
		Kazakh-Urban *vs.* Mongol-Urban	0.719	0.374	0.562	0.456	0.396	0.097	0.660	0.603
		Kazakh-Urban *vs.* Tibetan-Urban	0.331	0.875	0.344	0.538	0.669	0.852	0.265	0.221
		Kazakh-Urban *vs.* Uyghur-Urban	0.905	0.549	0.831	0.862	0.568	0.960	0.597	0.921
		Kazakh-Urban *vs.* Zhuang-Urban	0.772	0.637	0.629	0.498	0.660	0.059	0.622	0.644
		Mongol-Urban *vs.* Tibetan-Urban	0.487	0.312	0.607	0.879	0.254	0.071	0.154	0.068
		Mongol-Urban *vs.* Uyghur-Urban	0.909	0.219	0.785	0.703	0.260	0.087	0.936	0.567
		Mongol-Urban *vs.* Zhuang-Urban	0.910	0.598	0.851	0.870	0.651	0.206	0.809	0.929
		Tibetan-Urban *vs.* Uyghur-Urban	0.356	0.602	0.420	0.642	0.774	0.876	0.217	0.234
		Tibetan-Urban *vs.* Zhuang-Urban	0.372	0.452	0.504	0.836	0.355	0.050	0.161	0.048
		Uyghur-Urban *vs.* Zhuang-Urban	0.956	0.338	0.843	0.704	0.378	0.046	0.841	0.524
	(%) With significant differences		0	0	0	0	0	9.5% (2/21)	0	9.5% (2/21)
q = 1		Bai-Urban *vs.* Han-Urban	0.526	0.809	0.641	0.688	0.732	0.324	0.129	0.712
		Bai-Urban *vs.* Kazakh-Urban	0.761	0.537	0.886	0.924	0.491	0.688	0.128	0.907
		Bai-Urban *vs.* Mongol-Urban	0.585	0.703	0.690	0.736	0.814	0.659	0.845	0.701
		Bai-Urban *vs.* Tibetan-Urban	0.907	0.819	0.915	0.862	0.870	0.988	0.800	0.870
		Bai-Urban *vs.* Uyghur-Urban	0.745	0.372	0.853	0.904	0.463	0.897	0.431	0.825
		Bai-Urban *vs.* Zhuang-Urban	0.915	0.903	0.878	0.966	0.924	0.477	0.814	0.866
		Han-Urban *vs.* Kazakh-Urban	0.384	0.361	0.579	0.639	0.287	0.361	0.004	0.660
		Han-Urban *vs.* Mongol-Urban	0.897	0.497	0.953	0.942	0.535	0.372	0.090	0.985
		Han-Urban *vs.* Tibetan-Urban	0.648	0.634	0.582	0.498	0.619	0.349	0.080	0.833
		Han-Urban *vs.* Uyghur-Urban	0.810	0.323	0.856	0.869	0.362	0.327	0.023	0.875
	Phylum	Han-Urban *vs.* Zhuang-Urban	0.444	0.924	0.465	0.440	0.828	0.489	0.102	0.618
		Kazakh-Urban *vs.* Mongol-Urban	0.428	0.700	0.641	0.723	0.588	0.874	0.043	0.678
		Kazakh-Urban *vs.* Tibetan-Urban	0.710	0.684	0.976	0.972	0.623	0.702	0.312	0.826
		Kazakh-Urban *vs.* Uyghur-Urban	0.501	0.980	0.733	0.827	0.817	0.710	0.243	0.741
		Kazakh-Urban *vs.* Zhuang-Urban	0.853	0.524	0.994	0.953	0.492	0.558	0.342	0.974
		Mongol-Urban *vs.* Tibetan-Urban	0.760	0.943	0.621	0.591	0.984	0.654	0.619	0.863
		Mongol-Urban *vs.* Uyghur-Urban	0.873	0.618	0.866	0.864	0.635	0.669	0.225	0.867
		Mongol-Urban *vs.* Zhuang-Urban	0.609	0.680	0.580	0.670	0.752	0.733	0.667	0.647
		Tibetan-Urban *vs.* Uyghur-Urban	0.887	0.623	0.803	0.815	0.663	0.951	0.790	0.969
		Tibetan-Urban *vs.* Zhuang-Urban	0.839	0.750	0.970	0.899	0.792	0.456	0.970	0.776
		Uyghur-Urban *vs.* Zhuang-Urban	0.761	0.455	0.754	0.879	0.537	0.458	0.851	0.751
	(%) With significant differences		0	0	0	0	0	0	14.3% (3/21)	0
	Family	Bai-Urban *vs.* Han-Urban	0.549	0.844	0.624	0.573	0.961	0.219	0.875	0.772
		Bai-Urban *vs.* Kazakh-Urban	0.601	0.743	0.606	0.692	0.880	0.994	0.152	0.756
		Bai-Urban *vs.* Mongol-Urban	0.835	0.869	0.906	0.833	0.857	0.477	0.902	0.939
		Bai-Urban *vs.* Tibetan-Urban	0.660	0.766	0.875	0.990	0.885	0.604	0.778	0.886
		Bai-Urban *vs.* Uyghur-Urban	0.944	0.282	0.973	0.885	0.342	0.942	0.054	0.950
		Bai-Urban *vs.* Zhuang-Urban	0.871	0.971	0.969	0.853	0.971	0.604	0.696	0.815
		Han-Urban *vs.* Kazakh-Urban	0.273	0.843	0.252	0.315	0.913	0.241	0.061	0.550
		Han-Urban *vs.* Mongol-Urban	0.612	0.723	0.655	0.553	0.837	0.360	0.985	0.832
		Han-Urban *vs.* Tibetan-Urban	0.306	0.645	0.515	0.544	0.865	0.347	0.558	0.690
		Han-Urban *vs.* Uyghur-Urban	0.647	0.424	0.655	0.596	0.442	0.210	0.055	0.851
		Han-Urban *vs.* Zhuang-Urban	0.417	0.901	0.521	0.572	0.994	0.383	0.341	0.643
		Kazakh-Urban *vs.* Mongol-Urban	0.488	0.658	0.481	0.521	0.784	0.439	0.120	0.690

		Kazakh-Urban *vs.* Tibetan-Urban	0.917	0.620	0.750	0.748	0.802	0.596	0.334	0.863
		Kazakh-Urban *vs.* Uyghur-Urban	0.465	0.570	0.540	0.744	0.491	0.919	0.633	0.669
		Kazakh-Urban *vs.* Zhuang-Urban	0.669	0.775	0.582	0.527	0.890	0.526	0.469	0.863
		Mongol-Urban *vs.* Tibetan-Urban	0.520	0.849	0.803	0.845	0.989	0.687	0.611	0.820
		Mongol-Urban *vs.* Uyghur-Urban	0.953	0.322	0.884	0.722	0.356	0.390	0.110	0.995
		Mongol-Urban *vs.* Zhuang-Urban	0.740	0.855	0.864	0.986	0.830	0.903	0.573	0.789
		Tibetan-Urban *vs.* Uyghur-Urban	0.629	0.314	0.906	0.926	0.417	0.524	0.431	0.834
		Tibetan-Urban *vs.* Zhuang-Urban	0.731	0.757	0.893	0.834	0.848	0.767	0.798	0.978
		Uyghur-Urban *vs.* Zhuang-Urban	0.811	0.372	0.999	0.767	0.409	0.490	0.683	0.768
	(%) With significant differences		0	0	0	0	0	0	0	0
		Bai-Urban *vs.* Han-Urban	0.604	0.602	0.884	0.760	0.911	0.161	0.215	0.976
		Bai-Urban *vs.* Kazakh-Urban	0.562	0.741	0.506	0.595	0.934	0.082	0.093	0.528
		Bai-Urban *vs.* Mongol-Urban	0.902	0.965	0.908	0.993	0.842	0.098	0.797	0.842
		Bai-Urban *vs.* Tibetan-Urban	0.599	0.608	0.660	0.730	0.653	0.044	0.772	0.752
		Bai-Urban *vs.* Uyghur-Urban	0.998	0.395	0.803	0.685	0.477	0.056	0.077	0.947
		Bai-Urban *vs.* Zhuang-Urban	0.896	0.548	0.764	0.972	0.844	0.147	0.113	0.561
		Han-Urban *vs.* Kazakh-Urban	0.325	0.917	0.365	0.376	0.994	0.288	0.122	0.521
		Han-Urban *vs.* Mongol-Urban	0.647	0.602	0.776	0.587	0.785	0.364	0.340	0.859
		Han-Urban *vs.* Tibetan-Urban	0.230	0.277	0.398	0.389	0.510	0.371	0.716	0.624
		Han-Urban *vs.* Uyghur-Urban	0.659	0.688	0.684	0.524	0.549	0.240	0.213	0.962
	Genus	Han-Urban *vs.* Zhuang-Urban	0.433	0.914	0.588	0.545	0.969	0.556	0.172	0.568
		Kazakh-Urban *vs.* Mongol-Urban	0.477	0.733	0.499	0.532	0.809	0.630	0.113	0.642
		Kazakh-Urban *vs.* Tibetan-Urban	0.924	0.480	0.948	0.951	0.610	0.646	0.237	0.907
		Kazakh-Urban *vs.* Uyghur-Urban	0.529	0.592	0.646	0.872	0.506	0.771	0.521	0.533
		Kazakh-Urban *vs.* Zhuang-Urban	0.557	0.975	0.575	0.498	0.975	0.393	0.638	0.839
		Mongol-Urban *vs.* Tibetan-Urban	0.459	0.612	0.609	0.606	0.747	0.829	0.896	0.793
		Mongol-Urban *vs.* Uyghur-Urban	0.938	0.425	0.872	0.694	0.416	0.481	0.131	0.921
		Mongol-Urban *vs.* Zhuang-Urban	0.751	0.567	0.809	0.951	0.651	0.702	0.118	0.696
		Tibetan-Urban *vs.* Uyghur-Urban	0.582	0.312	0.787	0.967	0.383	0.497	0.353	0.766
		Tibetan-Urban *vs.* Zhuang-Urban	0.515	0.342	0.667	0.610	0.477	0.544	0.312	0.987
		Uyghur-Urban *vs.* Zhuang-Urban	0.898	0.561	0.987	0.676	0.473	0.379	0.977	0.598
	(%) With significant differences		0	0	0	0	0	4.8% (1/21)	0	0
		Bai-Urban *vs.* Han-Urban	0.512	0.422	0.578	0.479	0.551	0.428	0.202	0.856
		Bai-Urban *vs.* Kazakh-Urban	0.567	0.834	0.618	0.746	0.921	0.785	0.138	0.604
		Bai-Urban *vs.* Mongol-Urban	0.878	0.711	0.809	0.624	0.822	0.578	0.281	0.825
		Bai-Urban *vs.* Tibetan-Urban	0.915	0.590	0.805	0.806	0.535	0.407	0.941	0.777
		Bai-Urban *vs.* Uyghur-Urban	0.971	0.350	0.934	0.875	0.415	0.729	0.095	0.975
		Bai-Urban *vs.* Zhuang-Urban	0.876	0.537	0.979	0.727	0.673	0.255	0.102	0.582
		Han-Urban *vs.* Kazakh-Urban	0.236	0.615	0.266	0.293	0.688	0.492	0.156	0.463
		Han-Urban *vs.* Mongol-Urban	0.533	0.595	0.661	0.655	0.702	0.560	0.639	0.659
		Han-Urban *vs.* Tibetan-Urban	0.551	0.622	0.749	0.699	0.796	0.292	0.292	0.882
		Han-Urban *vs.* Uyghur-Urban	0.551	0.874	0.554	0.454	0.779	0.331	0.336	0.832
	Species	Han-Urban *vs.* Zhuang-Urban	0.362	0.698	0.471	0.496	0.759	0.812	0.251	0.482
		Kazakh-Urban *vs.* Mongol-Urban	0.435	0.953	0.410	0.409	0.921	0.733	0.150	0.663
		Kazakh-Urban *vs.* Tibetan-Urban	0.463	0.903	0.469	0.587	0.767	0.397	0.235	0.474
		Kazakh-Urban *vs.* Uyghur-Urban	0.576	0.551	0.661	0.827	0.536	0.585	0.550	0.658
		Kazakh-Urban *vs.* Zhuang-Urban	0.525	0.834	0.489	0.439	0.820	0.260	0.573	0.834
		Mongol-Urban *vs.* Tibetan-Urban	0.990	0.946	0.954	0.920	0.823	0.395	0.538	0.552
		Mongol-Urban *vs.* Uyghur-Urban	0.923	0.507	0.787	0.584	0.533	0.474	0.187	0.851
		Mongol-Urban *vs.* Zhuang-Urban	0.687	0.859	0.704	0.801	0.897	0.360	0.144	0.747
		Tibetan-Urban *vs.* Uyghur-Urban	0.911	0.440	0.742	0.699	0.510	0.575	0.187	0.709
		Tibetan-Urban *vs.* Zhuang-Urban	0.710	0.924	0.707	0.952	0.888	0.180	0.100	0.324
		Uyghur-Urban *vs.* Zhuang-Urban	0.875	0.526	0.951	0.643	0.509	0.185	0.982	0.599
	(%) With significant differences		0	0	0	0	0	0	0	0
$q = 2$	Phylum	Bai-Urban *vs.* Han-Urban	0.656	0.571	0.708	0.795	0.579	0.496	0.043	0.751

	Bai-Urban *vs.* Kazakh-Urban	0.946	0.594	0.980	0.967	0.584	0.585	0.313	0.967
	Bai-Urban *vs.* Mongol-Urban	0.661	0.872	0.752	0.821	0.902	0.790	0.635	0.739
	Bai-Urban *vs.* Tibetan-Urban	0.938	0.854	0.961	0.912	0.825	0.167	0.772	0.762
	Bai-Urban *vs.* Uyghur-Urban	0.751	0.684	0.765	0.836	0.689	0.532	0.958	0.736
	Bai-Urban *vs.* Zhuang-Urban	0.905	0.830	0.979	0.978	0.879	0.918	0.810	0.887
	Han-Urban *vs.* Kazakh-Urban	0.632	0.222	0.698	0.789	0.253	0.404	0.008	0.820
	Han-Urban *vs.* Mongol-Urban	0.973	0.393	0.974	0.965	0.469	0.551	0.023	0.938
	Han-Urban *vs.* Tibetan-Urban	0.750	0.447	0.682	0.596	0.450	0.852	0.029	0.953
	Han-Urban *vs.* Uyghur-Urban	0.976	0.296	0.945	0.926	0.346	0.407	0.012	0.945
	Han-Urban *vs.* Zhuang-Urban	0.749	0.763	0.708	0.606	0.720	0.480	0.129	0.894
	Kazakh-Urban *vs.* Mongol-Urban	0.670	0.599	0.765	0.797	0.582	0.548	0.079	0.789
	Kazakh-Urban *vs.* Tibetan-Urban	0.896	0.725	0.982	0.955	0.748	0.122	0.587	0.842
	Kazakh-Urban *vs.* Uyghur-Urban	0.734	0.777	0.770	0.817	0.770	0.954	0.244	0.811
	Kazakh-Urban *vs.* Zhuang-Urban	0.875	0.472	0.970	0.983	0.525	0.740	0.218	0.947
	Mongol-Urban *vs.* Tibetan-Urban	0.782	0.932	0.681	0.649	0.885	0.237	0.447	0.980
	Mongol-Urban *vs.* Uyghur-Urban	0.953	0.725	0.972	0.933	0.722	0.450	0.442	0.974
	Mongol-Urban *vs.* Zhuang-Urban	0.795	0.688	0.742	0.725	0.746	0.790	0.978	0.877
	Tibetan-Urban *vs.* Uyghur-Urban	0.843	0.889	0.743	0.749	0.905	0.138	0.771	0.999
	Tibetan-Urban *vs.* Zhuang-Urban	0.981	0.665	0.930	0.898	0.683	0.220	0.593	0.859
	Uyghur-Urban *vs.* Zhuang-Urban	0.863	0.526	0.789	0.805	0.578	0.701	0.673	0.874
(%) With significant differences		0	0	0	0	0	0	23.8% (5/21)	0
	Bai-Urban *vs.* Han-Urban	0.447	0.983	0.465	0.496	0.937	0.567	0.337	0.557
	Bai-Urban *vs.* Kazakh-Urban	0.666	0.882	0.752	0.851	0.941	0.921	0.296	0.898
	Bai-Urban *vs.* Mongol-Urban	0.601	0.976	0.610	0.673	0.829	0.126	0.590	0.615
	Bai-Urban *vs.* Tibetan-Urban	0.743	0.764	0.895	0.955	0.889	0.788	0.935	0.993
	Bai-Urban *vs.* Uyghur-Urban	0.867	0.419	0.891	0.969	0.411	0.070	0.149	0.793
	Bai-Urban *vs.* Zhuang-Urban	0.903	0.901	0.771	0.751	0.869	0.135	0.878	0.850
	Han-Urban *vs.* Kazakh-Urban	0.255	0.885	0.281	0.403	0.990	0.532	0.061	0.528
	Han-Urban *vs.* Mongol-Urban	0.757	0.999	0.785	0.641	0.915	0.322	0.695	0.959
	Han-Urban *vs.* Tibetan-Urban	0.298	0.731	0.379	0.396	0.841	0.561	0.313	0.622
	Han-Urban *vs.* Uyghur-Urban	0.610	0.481	0.609	0.582	0.501	0.269	0.069	0.760
Family	Han-Urban *vs.* Zhuang-Urban	0.499	0.895	0.639	0.634	0.961	0.268	0.279	0.745
	Kazakh-Urban *vs.* Mongol-Urban	0.380	0.870	0.469	0.576	0.921	0.120	0.150	0.535
	Kazakh-Urban *vs.* Tibetan-Urban	0.854	0.719	0.844	0.903	0.842	0.873	0.358	0.906
	Kazakh-Urban *vs.* Uyghur-Urban	0.507	0.627	0.642	0.870	0.523	0.094	0.695	0.722
	Kazakh-Urban *vs.* Zhuang-Urban	0.546	0.814	0.564	0.610	0.978	0.092	0.510	0.745
	Mongol-Urban *vs.* Tibetan-Urban	0.435	0.729	0.582	0.631	0.739	0.150	0.588	0.664
	Mongol-Urban *vs.* Uyghur-Urban	0.773	0.434	0.745	0.651	0.494	0.742	0.131	0.787
	Mongol-Urban *vs.* Zhuang-Urban	0.706	0.910	0.858	0.983	0.954	0.526	0.638	0.731
	Tibetan-Urban *vs.* Uyghur-Urban	0.643	0.368	0.806	0.979	0.418	0.086	0.418	0.818
	Tibetan-Urban *vs.* Zhuang-Urban	0.657	0.854	0.670	0.678	0.775	0.121	0.897	0.835
	Uyghur-Urban *vs.* Zhuang-Urban	0.953	0.395	0.862	0.724	0.492	0.710	0.583	0.939
(%) With significant differences		0	0	0	0	0	0	0	0
Genus	Bai-Urban *vs.* Han-Urban	0.478	0.542	0.814	0.756	0.879	0.415	0.257	0.988
	Bai-Urban *vs.* Kazakh-Urban	0.657	0.694	0.564	0.607	0.899	0.631	0.093	0.574
	Bai-Urban *vs.* Mongol-Urban	0.701	0.787	0.977	0.974	0.927	0.514	0.606	0.966
	Bai-Urban *vs.* Tibetan-Urban	0.674	0.581	0.749	0.765	0.687	0.941	0.946	0.841
	Bai-Urban *vs.* Uyghur-Urban	0.891	0.409	0.847	0.678	0.486	0.671	0.143	0.997
	Bai-Urban *vs.* Zhuang-Urban	0.821	0.512	0.904	0.991	0.797	0.333	0.223	0.712
	Han-Urban *vs.* Kazakh-Urban	0.277	0.889	0.320	0.352	0.967	0.590	0.094	0.498
	Han-Urban *vs.* Mongol-Urban	0.655	0.709	0.831	0.612	0.943	0.685	0.570	0.938
	Han-Urban *vs.* Tibetan-Urban	0.184	0.184	0.448	0.400	0.492	0.468	0.483	0.777
	Han-Urban *vs.* Uyghur-Urban	0.595	0.768	0.628	0.473	0.565	0.560	0.246	0.984
	Han-Urban *vs.* Zhuang-Urban	0.490	0.900	0.661	0.563	0.966	0.947	0.356	0.707
	Kazakh-Urban *vs.* Mongol-Urban	0.396	0.878	0.415	0.478	0.971	0.701	0.089	0.539
	Kazakh-Urban *vs.* Tibetan-Urban	0.912	0.438	0.888	0.900	0.624	0.631	0.184	0.777

		Kazakh-Urban *vs.* Uyghur-Urban	0.502	0.664	0.616	0.859	0.553	0.800	0.355	0.520
		Kazakh-Urban *vs.* Zhuang-Urban	0.453	0.969	0.486	0.462	0.944	0.415	0.288	0.684
		Mongol-Urban *vs.* Tibetan-Urban	0.390	0.385	0.627	0.611	0.598	0.484	0.763	0.853
		Mongol-Urban *vs.* Uyghur-Urban	0.845	0.551	0.754	0.606	0.502	0.578	0.203	0.965
		Mongol-Urban *vs.* Zhuang-Urban	0.813	0.777	0.806	0.932	0.882	0.611	0.328	0.764
		Tibetan-Urban *vs.* Uyghur-Urban	0.552	0.289	0.831	0.999	0.393	0.698	0.335	0.836
		Tibetan-Urban *vs.* Zhuang-Urban	0.430	0.279	0.686	0.634	0.498	0.323	0.356	0.990
		Uyghur-Urban *vs.* Zhuang-Urban	0.977	0.634	0.900	0.611	0.504	0.365	0.869	0.715
	(%) With significant differences		0	0	0	0	0	0	0	0
	Species	Bai-Urban *vs.* Han-Urban	0.445	0.412	0.620	0.583	0.578	0.525	0.379	0.775
		Bai-Urban *vs.* Kazakh-Urban	0.731	0.614	0.749	0.853	0.721	0.995	0.206	0.807
		Bai-Urban *vs.* Mongol-Urban	0.622	0.439	0.753	0.669	0.606	0.159	0.209	0.996
		Bai-Urban *vs.* Tibetan-Urban	0.871	0.556	0.790	0.761	0.526	0.956	0.803	0.784
		Bai-Urban *vs.* Uyghur-Urban	0.827	0.289	0.973	0.904	0.414	0.857	0.147	0.932
		Bai-Urban *vs.* Zhuang-Urban	0.891	0.396	0.936	0.847	0.556	0.544	0.143	0.782
		Han-Urban *vs.* Kazakh-Urban	0.266	0.784	0.399	0.486	0.848	0.490	0.271	0.595
		Han-Urban *vs.* Mongol-Urban	0.642	0.775	0.743	0.725	0.852	0.326	0.934	0.724
		Han-Urban *vs.* Tibetan-Urban	0.492	0.620	0.855	0.913	0.798	0.487	0.635	0.963
		Han-Urban *vs.* Uyghur-Urban	0.564	0.903	0.622	0.560	0.838	0.424	0.464	0.833
		Han-Urban *vs.* Zhuang-Urban	0.452	0.843	0.493	0.483	0.852	0.770	0.260	0.558
		Kazakh-Urban *vs.* Mongol-Urban	0.425	0.955	0.497	0.554	0.961	0.158	0.264	0.745
		Kazakh-Urban *vs.* Tibetan-Urban	0.615	0.915	0.569	0.653	0.997	0.968	0.379	0.622
		Kazakh-Urban *vs.* Uyghur-Urban	0.624	0.712	0.767	0.924	0.706	0.842	0.518	0.761
		Kazakh-Urban *vs.* Zhuang-Urban	0.594	0.892	0.721	0.698	0.974	0.539	0.858	0.954
		Mongol-Urban *vs.* Tibetan-Urban	0.761	0.799	0.942	0.895	0.954	0.167	0.562	0.717
		Mongol-Urban *vs.* Uyghur-Urban	0.827	0.641	0.764	0.624	0.678	0.133	0.350	0.922
		Mongol-Urban *vs.* Zhuang-Urban	0.661	0.924	0.634	0.738	0.996	0.248	0.274	0.764
		Tibetan-Urban *vs.* Uyghur-Urban	0.958	0.465	0.746	0.669	0.570	0.899	0.344	0.844
		Tibetan-Urban *vs.* Zhuang-Urban	0.937	0.700	0.616	0.729	0.942	0.497	0.224	0.508
		Uyghur-Urban *vs.* Zhuang-Urban	0.889	0.682	0.969	0.767	0.621	0.437	0.679	0.668
	(%) With significant differences		0	0	0	0	0	0	0	0
$q = 3$	Phylum	Bai-Urban *vs.* Han-Urban	0.759	0.439	0.811	0.848	0.444	0.559	0.029	0.920
		Bai-Urban *vs.* Kazakh-Urban	0.945	0.592	0.997	0.988	0.715	0.379	0.466	0.993
		Bai-Urban *vs.* Mongol-Urban	0.736	0.970	0.823	0.843	0.960	0.811	0.541	0.875
		Bai-Urban *vs.* Tibetan-Urban	0.924	0.825	0.931	0.882	0.835	0.424	0.749	0.952
		Bai-Urban *vs.* Uyghur-Urban	0.775	0.796	0.806	0.819	0.858	0.287	0.862	0.842
		Bai-Urban *vs.* Zhuang-Urban	0.931	0.751	0.988	0.997	0.714	0.602	0.617	0.993
		Han-Urban *vs.* Kazakh-Urban	0.880	0.147	0.822	0.891	0.250	0.356	0.010	0.916
		Han-Urban *vs.* Mongol-Urban	0.923	0.364	0.941	0.909	0.457	0.493	0.019	0.897
		Han-Urban *vs.* Tibetan-Urban	0.889	0.340	0.724	0.591	0.361	0.437	0.023	0.997
		Han-Urban *vs.* Uyghur-Urban	0.920	0.266	0.872	0.851	0.337	0.337	0.018	0.902
		Han-Urban *vs.* Zhuang-Urban	0.823	0.698	0.756	0.719	0.756	0.433	0.170	0.902
		Kazakh-Urban *vs.* Mongol-Urban	0.867	0.516	0.857	0.885	0.609	0.579	0.100	0.858
		Kazakh-Urban *vs.* Tibetan-Urban	0.987	0.770	0.928	0.879	0.851	0.778	0.812	0.960
		Kazakh-Urban *vs.* Uyghur-Urban	0.879	0.657	0.814	0.859	0.771	0.815	0.268	0.869
		Kazakh-Urban *vs.* Zhuang-Urban	0.998	0.404	0.994	0.977	0.508	0.751	0.211	0.994
		Mongol-Urban *vs.* Tibetan-Urban	0.871	0.802	0.715	0.627	0.780	0.670	0.348	0.944
		Mongol-Urban *vs.* Uyghur-Urban	0.972	0.755	0.925	0.890	0.757	0.417	0.477	0.956
		Mongol-Urban *vs.* Zhuang-Urban	0.811	0.682	0.777	0.785	0.702	0.798	0.873	0.845
		Tibetan-Urban *vs.* Uyghur-Urban	0.868	0.960	0.758	0.711	0.948	0.615	0.649	0.927
		Tibetan-Urban *vs.* Zhuang-Urban	0.976	0.586	0.939	0.850	0.561	0.908	0.418	0.937
		Uyghur-Urban *vs.* Zhuang-Urban	0.838	0.523	0.780	0.787	0.549	0.622	0.600	0.864
	(%) With significant differences		0	0	0	0	0	0	23.8% (5/21)	0
	Family	Bai-Urban *vs.* Han-Urban	0.406	0.990	0.449	0.513	0.949	0.041	0.271	0.529
		Bai-Urban *vs.* Kazakh-Urban	0.670	0.978	0.857	0.961	0.965	0.479	0.064	0.983

	Bai-Urban *vs.* Mongol-Urban	0.617	0.955	0.586	0.669	0.833	0.431	0.407	0.545
	Bai-Urban *vs.* Tibetan-Urban	0.817	0.786	0.917	0.947	0.872	0.309	0.806	0.926
	Bai-Urban *vs.* Uyghur-Urban	0.872	0.528	0.933	0.891	0.532	0.300	0.382	0.779
	Bai-Urban *vs.* Zhuang-Urban	0.832	0.900	0.715	0.736	0.859	0.524	0.752	0.697
	Han-Urban *vs.* Kazakh-Urban	0.215	0.984	0.349	0.529	0.984	0.046	0.004	0.588
	Han-Urban *vs.* Mongol-Urban	0.691	0.947	0.818	0.734	0.895	0.045	0.873	1.000
	Han-Urban *vs.* Tibetan-Urban	0.304	0.734	0.384	0.421	0.810	0.060	0.383	0.651
	Han-Urban *vs.* Uyghur-Urban	0.565	0.574	0.550	0.530	0.590	0.045	0.101	0.738
	Han-Urban *vs.* Zhuang-Urban	0.551	0.868	0.712	0.696	0.924	0.052	0.635	0.838
	Kazakh-Urban *vs.* Mongol-Urban	0.366	0.933	0.524	0.695	0.886	0.235	0.008	0.569
	Kazakh-Urban *vs.* Tibetan-Urban	0.789	0.817	0.924	0.993	0.862	0.215	0.057	0.926
	Kazakh-Urban *vs.* Uyghur-Urban	0.526	0.633	0.796	0.971	0.607	0.171	0.100	0.822
	Kazakh-Urban *vs.* Zhuang-Urban	0.486	0.891	0.602	0.746	0.938	0.265	0.062	0.744
	Mongol-Urban *vs.* Tibetan-Urban	0.496	0.791	0.555	0.615	0.741	0.788	0.594	0.654
	Mongol-Urban *vs.* Uyghur-Urban	0.777	0.490	0.642	0.569	0.631	0.862	0.177	0.734
	Mongol-Urban *vs.* Zhuang-Urban	0.801	0.939	0.897	0.979	0.969	0.930	0.850	0.824
	Tibetan-Urban *vs.* Uyghur-Urban	0.723	0.425	0.858	0.978	0.482	0.844	0.430	0.877
	Tibetan-Urban *vs.* Zhuang-Urban	0.654	0.881	0.623	0.650	0.759	0.722	0.912	0.772
	Uyghur-Urban *vs.* Zhuang-Urban	0.952	0.434	0.753	0.639	0.605	0.817	0.410	0.904
(%) With significant differences		0	0	0	0	0	19.0% (4/21)	9.5% (2/21)	0
	Bai-Urban *vs.* Han-Urban	0.425	0.500	0.819	0.825	0.831	0.434	0.356	0.976
	Bai-Urban *vs.* Kazakh-Urban	0.695	0.705	0.568	0.620	0.887	0.627	0.127	0.586
	Bai-Urban *vs.* Mongol-Urban	0.668	0.725	1.000	0.944	0.862	0.612	0.643	0.997
	Bai-Urban *vs.* Tibetan-Urban	0.791	0.636	0.777	0.764	0.729	0.868	0.917	0.929
	Bai-Urban *vs.* Uyghur-Urban	0.843	0.418	0.820	0.638	0.490	0.322	0.196	1.000
	Bai-Urban *vs.* Zhuang-Urban	0.751	0.546	0.916	0.916	0.781	0.251	0.324	0.784
	Han-Urban *vs.* Kazakh-Urban	0.266	0.841	0.325	0.373	0.938	0.624	0.100	0.494
	Han-Urban *vs.* Mongol-Urban	0.644	0.730	0.809	0.569	0.960	0.613	0.664	0.945
	Han-Urban *vs.* Tibetan-Urban	0.258	0.210	0.483	0.413	0.496	0.445	0.367	0.878
	Han-Urban *vs.* Uyghur-Urban	0.564	0.812	0.584	0.430	0.611	0.871	0.276	0.974
Genus	Han-Urban *vs.* Zhuang-Urban	0.497	0.864	0.665	0.569	0.994	0.861	0.428	0.747
	Kazakh-Urban *vs.* Mongol-Urban	0.382	0.928	0.436	0.529	0.979	0.866	0.099	0.517
	Kazakh-Urban *vs.* Tibetan-Urban	0.940	0.467	0.852	0.891	0.649	0.491	0.174	0.718
	Kazakh-Urban *vs.* Uyghur-Urban	0.510	0.691	0.663	0.911	0.571	0.537	0.355	0.529
	Kazakh-Urban *vs.* Zhuang-Urban	0.428	0.972	0.503	0.530	0.949	0.304	0.299	0.654
	Mongol-Urban *vs.* Tibetan-Urban	0.503	0.415	0.682	0.667	0.598	0.522	0.593	0.925
	Mongol-Urban *vs.* Uyghur-Urban	0.839	0.620	0.749	0.583	0.560	0.814	0.254	1.000
	Mongol-Urban *vs.* Zhuang-Urban	0.852	0.880	0.849	0.974	0.946	0.387	0.423	0.770
	Tibetan-Urban *vs.* Uyghur-Urban	0.664	0.316	0.883	0.963	0.419	0.266	0.311	0.925
	Tibetan-Urban *vs.* Zhuang-Urban	0.518	0.332	0.743	0.664	0.540	0.195	0.347	0.895
	Uyghur-Urban *vs.* Zhuang-Urban	0.928	0.659	0.858	0.617	0.539	0.343	0.854	0.774
(%) With significant differences		0	0	0	0	0	0	0	0
Species	Bai-Urban *vs.* Han-Urban	0.533	0.502	0.706	0.630	0.711	0.166	0.489	0.859
	Bai-Urban *vs.* Kazakh-Urban	0.707	0.608	0.792	0.958	0.746	0.713	0.169	0.730
	Bai-Urban *vs.* Mongol-Urban	0.735	0.513	0.869	0.711	0.778	0.426	0.313	0.907
	Bai-Urban *vs.* Tibetan-Urban	0.946	0.602	0.813	0.734	0.654	0.764	0.775	0.870
	Bai-Urban *vs.* Uyghur-Urban	0.950	0.398	0.884	0.840	0.571	0.717	0.218	0.945
	Bai-Urban *vs.* Zhuang-Urban	0.969	0.472	0.909	0.855	0.701	0.208	0.209	0.726
	Han-Urban *vs.* Kazakh-Urban	0.304	0.897	0.505	0.639	0.933	0.165	0.207	0.611
	Han-Urban *vs.* Mongol-Urban	0.672	0.838	0.737	0.714	0.868	0.256	0.941	0.752
	Han-Urban *vs.* Tibetan-Urban	0.525	0.729	0.909	0.983	0.856	0.160	0.770	0.962
	Han-Urban *vs.* Uyghur-Urban	0.574	0.926	0.609	0.554	0.902	0.132	0.559	0.824
	Han-Urban *vs.* Zhuang-Urban	0.489	0.913	0.538	0.524	0.909	0.666	0.235	0.610
	Kazakh-Urban *vs.* Mongol-Urban	0.453	0.947	0.627	0.732	0.946	0.501	0.181	0.749
	Kazakh-Urban *vs.* Tibetan-Urban	0.655	0.867	0.624	0.698	0.936	0.606	0.345	0.628
	Kazakh-Urban *vs.* Uyghur-Urban	0.674	0.837	0.888	0.904	0.831	0.479	0.342	0.775

Kazakh-Urban *vs.* Zhuang-Urban	0.630	0.988	0.832	0.839	0.974	0.177	0.798	0.959
Mongol-Urban *vs.* Tibetan-Urban	0.747	0.840	0.875	0.811	1.000	0.382	0.651	0.760
Mongol-Urban *vs.* Uyghur-Urban	0.787	0.732	0.733	0.581	0.724	0.276	0.464	0.982
Mongol-Urban *vs.* Zhuang-Urban	0.685	0.917	0.726	0.796	0.955	0.316	0.261	0.777
Tibetan-Urban *vs.* Uyghur-Urban	0.994	0.601	0.657	0.559	0.700	0.887	0.430	0.800
Tibetan-Urban *vs.* Zhuang-Urban	0.973	0.758	0.639	0.686	0.932	0.193	0.295	0.571
Uyghur-Urban *vs.* Zhuang-Urban	0.968	0.805	0.951	0.708	0.755	0.132	0.538	0.763
(%) With significant differences	0	0	0	0	0	0	0	0
