# Supplementary material for: Diversity Scaling Analysis of Chinese Gut Microbiomes Across Ethnicities and Lifestyles
Source: Front Microbiol. 2021 Dec 8;12:736393. doi: 10.3389/fmicb.2021.736393 (PMC8692740; doi:10.3389/fmicb.2021.736393)
Supplement: Supplementary file 1 [file Data_Sheet_1.ZIP › TableS1-S2/CGP-Tables S1-S2.doc]

**Tables S1-S2**

Table S1. Fitting the DAR (diversity-area relationship) models for all cohorts of the Chinese gut microbiome datasets (with 100 times of random permutations of microbiome samples) for the phylum-, family- and genus-level counterpart results

| **Taxon** | **Cohort** | **Diversity Order** | **Power Law (PL)** | | | | | | **PL with Exponential Cutoff (PLEC)** | | | | | | | | |
| --- | --- | --- | --- | --- | --- | --- | --- | --- | --- | --- | --- | --- | --- | --- | --- | --- | --- |
|  |  |  | ***z*** | **ln(*c*)** | ***R*** | ***p*-value** | ***g*** | ***N*** | ***z*** | ***d*** | **ln(*c*)** | ***R*** | ***p*-value** | ***N*** | ***A_max_*** | ***D_max_*** | ***LGD*** |
| Phylum | Total | *q* = 0 | 0.169 | 3.002 | 0.966 | 0.000 | 0.876 | 100 | 0.228 | -0.001 | 2.833 | 0.986 | 0.000 | 97 | 558 | 51.8 | 39.3 |
|  |  | *q* = 1 | 0.009 | 1.092 | 0.497 | 0.001 | 0.994 | 86 | 0.024 | 0.000 | 1.049 | 0.618 | 0.001 | 83 | 200 | 3.1 | 95.8 |
|  |  | *q* = 2 | 0.000 | 0.669 | 0.488 | 0.002 | 1.000 | 87 | 0.006 | 0.000 | 0.653 | 0.612 | 0.000 | 83 | 844 | 2.0 | 100.6 |
|  |  | *q* = 3 | -0.001 | 0.542 | 0.502 | 0.002 | 1.001 | 84 | 0.002 | 0.000 | 0.529 | 0.610 | 0.001 | 80 | 200 | 1.7 | 100.6 |
|  | Rural | *q* = 0 | 0.192 | 2.914 | 0.977 | 0.000 | 0.858 | 100 | 0.238 | -0.001 | 2.810 | 0.986 | 0.000 | 89 | 407 | 50.1 | 37.4 |
|  |  | *q* = 1 | 0.020 | 1.068 | 0.595 | 0.001 | 0.985 | 90 | 0.037 | 0.000 | 1.031 | 0.685 | 0.001 | 84 | 364 | 3.2 | 91.7 |
|  |  | *q* = 2 | 0.007 | 0.670 | 0.572 | 0.001 | 0.995 | 91 | 0.010 | 0.000 | 0.663 | 0.655 | 0.001 | 85 | 203 | 2.0 | 97.9 |
|  |  | *q* = 3 | 0.003 | 0.550 | 0.579 | 0.000 | 0.998 | 89 | 0.000 | 0.000 | 0.559 | 0.671 | 0.001 | 83 | 341 | 1.8 | 100.8 |
|  | Urban | *q* = 0 | 0.218 | 2.833 | 0.981 | 0.000 | 0.837 | 100 | 0.268 | -0.001 | 2.730 | 0.989 | 0.000 | 77 | 344 | 51.8 | 33.5 |
|  |  | *q* = 1 | 0.018 | 1.009 | 0.581 | 0.003 | 0.987 | 90 | 0.033 | 0.000 | 0.986 | 0.668 | 0.001 | 81 | 90 | 3.0 | 93.9 |
|  |  | *q* = 2 | 0.005 | 0.605 | 0.584 | 0.002 | 0.996 | 84 | 0.006 | 0.000 | 0.601 | 0.668 | 0.001 | 82 | 247 | 1.9 | 100.0 |
|  |  | *q* = 3 | 0.002 | 0.489 | 0.596 | 0.001 | 0.999 | 82 | 0.001 | 0.000 | 0.490 | 0.667 | 0.001 | 83 | 123 | 1.6 | 100.4 |
|  | Bai | *q* = 0 | 0.242 | 2.763 | 0.964 | 0.000 | 0.817 | 100 | 0.288 | -0.004 | 2.761 | 0.974 | 0.000 | 42 | 123 | 43.4 | 40.2 |
|  |  | *q* = 1 | 0.038 | 0.942 | 0.662 | 0.005 | 0.972 | 78 | 0.064 | -0.003 | 0.945 | 0.721 | 0.002 | 71 | 32 | 3.0 | 93.2 |
|  |  | *q* = 2 | 0.009 | 0.606 | 0.673 | 0.003 | 0.992 | 76 | 0.017 | -0.001 | 0.619 | 0.715 | 0.003 | 76 | 36 | 1.9 | 101.1 |
|  |  | *q* = 3 | 0.001 | 0.510 | 0.699 | 0.003 | 0.998 | 71 | 0.001 | 0.000 | 0.523 | 0.735 | 0.004 | 72 | 29 | 1.7 | 102.4 |
|  | Han | *q* = 0 | 0.186 | 2.905 | 0.968 | 0.000 | 0.862 | 100 | 0.243 | -0.002 | 2.808 | 0.981 | 0.000 | 88 | 237 | 42.9 | 43.7 |
|  |  | *q* = 1 | 0.019 | 0.894 | 0.622 | 0.002 | 0.987 | 83 | 0.036 | -0.001 | 0.866 | 0.695 | 0.001 | 74 | 73 | 2.6 | 94.2 |
|  |  | *q* = 2 | 0.001 | 0.513 | 0.601 | 0.002 | 0.999 | 83 | 0.000 | 0.000 | 0.519 | 0.682 | 0.001 | 80 | 113 | 1.7 | 102.8 |
|  |  | *q* = 3 | -0.002 | 0.410 | 0.595 | 0.002 | 1.001 | 84 | -0.006 | 0.000 | 0.421 | 0.686 | 0.001 | 79 | 87 | 1.5 | 102.7 |
|  | Kazakh | *q* = 0 | 0.219 | 2.814 | 0.974 | 0.000 | 0.835 | 100 | 0.292 | -0.009 | 2.768 | 0.983 | 0.000 | 74 | 55 | 34.3 | 49.7 |
|  |  | *q* = 1 | 0.076 | 1.130 | 0.765 | 0.002 | 0.943 | 73 | 0.151 | -0.011 | 1.095 | 0.817 | 0.004 | 75 | 17 | 3.9 | 84.0 |
|  |  | *q* = 2 | 0.026 | 0.764 | 0.769 | 0.003 | 0.979 | 79 | 0.069 | -0.006 | 0.733 | 0.830 | 0.003 | 74 | 19 | 2.3 | 95.7 |
|  |  | *q* = 3 | 0.008 | 0.644 | 0.774 | 0.003 | 0.992 | 80 | 0.031 | -0.003 | 0.627 | 0.844 | 0.002 | 75 | 20 | 2.0 | 101.5 |
|  | Mongol | *q* = 0 | 0.219 | 2.887 | 0.978 | 0.000 | 0.836 | 100 | 0.272 | -0.003 | 2.821 | 0.985 | 0.000 | 73 | 259 | 47.1 | 39.6 |
|  |  | *q* = 1 | 0.018 | 1.101 | 0.636 | 0.005 | 0.987 | 75 | 0.025 | -0.001 | 1.097 | 0.722 | 0.003 | 83 | 35 | 3.2 | 95.3 |
|  |  | *q* = 2 | 0.001 | 0.714 | 0.662 | 0.002 | 0.999 | 71 | -0.006 | 0.001 | 0.715 | 0.714 | 0.002 | 83 | 64 | 2.0 | 100.3 |
|  |  | *q* = 3 | -0.003 | 0.590 | 0.635 | 0.005 | 1.002 | 78 | -0.013 | 0.001 | 0.598 | 0.717 | 0.002 | 84 | 46 | 1.8 | 102.1 |
|  | Tibetan | *q* = 0 | 0.238 | 2.681 | 0.976 | 0.000 | 0.821 | 100 | 0.299 | -0.004 | 2.599 | 0.984 | 0.000 | 50 | 348 | 42.9 | 34.9 |
|  |  | *q* = 1 | 0.053 | 1.044 | 0.705 | 0.002 | 0.961 | 82 | 0.090 | -0.003 | 1.024 | 0.777 | 0.001 | 78 | 62 | 3.5 | 86.3 |
|  |  | *q* = 2 | 0.028 | 0.757 | 0.629 | 0.006 | 0.979 | 74 | 0.040 | -0.002 | 0.771 | 0.723 | 0.002 | 80 | 26 | 2.4 | 95.5 |
|  |  | *q* = 3 | 0.021 | 0.669 | 0.632 | 0.004 | 0.984 | 69 | 0.026 | -0.001 | 0.683 | 0.708 | 0.003 | 76 | 38 | 2.1 | 96.8 |
|  | Uyghur | *q* = 0 | 0.205 | 2.955 | 0.971 | 0.000 | 0.847 | 100 | 0.286 | -0.011 | 2.906 | 0.985 | 0.000 | 75 | 97 | 37.3 | 52.5 |
|  |  | *q* = 1 | 0.042 | 1.210 | 0.748 | 0.003 | 0.969 | 73 | 0.063 | -0.004 | 1.214 | 0.823 | 0.003 | 74 | 19 | 3.8 | 92.5 |
|  |  | *q* = 2 | 0.003 | 0.819 | 0.730 | 0.004 | 0.995 | 79 | -0.009 | 0.001 | 0.846 | 0.822 | 0.004 | 73 | 16 | 2.3 | 105.5 |
|  |  | *q* = 3 | -0.010 | 0.690 | 0.731 | 0.005 | 1.005 | 78 | -0.038 | 0.003 | 0.727 | 0.822 | 0.004 | 74 | 18 | 2.0 | 108.9 |
|  | Zhuang | *q* = 0 | 0.240 | 2.762 | 0.980 | 0.000 | 0.819 | 100 | 0.302 | -0.004 | 2.680 | 0.986 | 0.000 | 68 | 944 | 45.6 | 36.6 |
|  |  | *q* = 1 | 0.039 | 0.936 | 0.635 | 0.004 | 0.972 | 76 | 0.066 | -0.003 | 0.941 | 0.731 | 0.001 | 74 | 242 | 3.0 | 93.0 |
|  |  | *q* = 2 | 0.008 | 0.568 | 0.631 | 0.003 | 0.994 | 73 | 0.014 | -0.001 | 0.576 | 0.733 | 0.002 | 70 | 25 | 1.8 | 100.7 |
|  |  | *q* = 3 | 0.000 | 0.471 | 0.629 | 0.003 | 0.999 | 74 | 0.002 | 0.000 | 0.474 | 0.734 | 0.002 | 68 | 49 | 1.6 | 102.4 |
|  | Bai-Rural | *q* = 0 | 0.227 | 2.756 | 0.955 | 0.000 | 0.829 | 100 | 0.304 | -0.010 | 2.744 | 0.965 | 0.000 | 49 | 47 | 34.2 | 49.8 |
|  |  | *q* = 1 | 0.059 | 0.873 | 0.704 | 0.004 | 0.957 | 77 | 0.117 | -0.008 | 0.859 | 0.780 | 0.002 | 78 | 20 | 2.9 | 88.1 |
|  |  | *q* = 2 | 0.021 | 0.548 | 0.710 | 0.002 | 0.984 | 73 | 0.058 | -0.004 | 0.524 | 0.789 | 0.001 | 72 | 31 | 1.9 | 95.7 |
|  |  | *q* = 3 | 0.010 | 0.457 | 0.702 | 0.003 | 0.992 | 77 | 0.038 | -0.003 | 0.431 | 0.794 | 0.002 | 73 | 33 | 1.7 | 97.4 |
|  | Bai-Urban | *q* = 0 | 0.240 | 2.785 | 0.960 | 0.000 | 0.818 | 100 | 0.310 | -0.013 | 2.768 | 0.972 | 0.000 | 47 | 81 | 33.6 | 52.4 |
|  |  | *q* = 1 | 0.078 | 0.890 | 0.780 | 0.006 | 0.943 | 52 | 0.119 | -0.012 | 0.930 | 0.837 | 0.007 | 62 | 9 | 3.0 | 88.5 |
|  |  | *q* = 2 | 0.041 | 0.599 | 0.771 | 0.008 | 0.969 | 55 | 0.069 | -0.006 | 0.606 | 0.848 | 0.005 | 62 | 13 | 2.0 | 94.2 |
|  |  | *q* = 3 | 0.023 | 0.526 | 0.789 | 0.006 | 0.981 | 53 | 0.035 | -0.004 | 0.545 | 0.844 | 0.006 | 68 | 18 | 1.8 | 99.2 |
|  | Han-Rural | *q* = 0 | 0.197 | 2.899 | 0.967 | 0.000 | 0.854 | 100 | 0.279 | -0.006 | 2.800 | 0.983 | 0.000 | 84 | 68 | 37.5 | 48.5 |
|  |  | *q* = 1 | 0.028 | 0.935 | 0.662 | 0.004 | 0.979 | 68 | 0.043 | -0.002 | 0.950 | 0.694 | 0.004 | 71 | 45 | 2.9 | 95.6 |
|  |  | *q* = 2 | -0.006 | 0.593 | 0.628 | 0.003 | 1.003 | 74 | -0.002 | 0.000 | 0.578 | 0.693 | 0.002 | 74 | 59 | 1.8 | 101.2 |
|  |  | *q* = 3 | -0.012 | 0.489 | 0.618 | 0.004 | 1.007 | 77 | -0.016 | 0.001 | 0.484 | 0.696 | 0.001 | 72 | 45 | 1.6 | 103.8 |
|  | Han-Urban | *q* = 0 | 0.221 | 2.801 | 0.972 | 0.000 | 0.835 | 100 | 0.292 | -0.005 | 2.709 | 0.981 | 0.000 | 63 | 271 | 40.9 | 41.0 |
|  |  | *q* = 1 | 0.017 | 0.824 | 0.674 | 0.003 | 0.988 | 75 | 0.026 | -0.001 | 0.822 | 0.762 | 0.001 | 79 | 39 | 2.4 | 96.5 |
|  |  | *q* = 2 | 0.001 | 0.455 | 0.655 | 0.002 | 0.999 | 74 | 0.001 | 0.000 | 0.456 | 0.748 | 0.001 | 80 | 32 | 1.6 | 100.7 |
|  |  | *q* = 3 | -0.001 | 0.359 | 0.657 | 0.001 | 1.000 | 73 | -0.003 | 0.000 | 0.362 | 0.754 | 0.001 | 78 | 34 | 1.4 | 101.0 |
|  | Kazakh-Rural | *q* = 0 | 0.208 | 2.846 | 0.949 | 0.000 | 0.844 | 100 | 0.348 | -0.038 | 2.860 | 0.974 | 0.000 | 60 | 23 | 28.3 | 63.2 |
|  |  | *q* = 1 | 0.051 | 1.069 | 0.839 | 0.011 | 0.959 | 49 | 0.108 | -0.013 | 1.046 | 0.899 | 0.012 | 50 | 6 | 3.2 | 91.7 |
|  |  | *q* = 2 | 0.002 | 0.663 | 0.852 | 0.011 | 0.994 | 46 | 0.032 | 0.000 | 0.601 | 0.899 | 0.012 | 41 | 13 | 1.9 | 99.0 |
|  |  | *q* = 3 | -0.012 | 0.544 | 0.855 | 0.011 | 1.004 | 45 | -0.010 | 0.005 | 0.501 | 0.905 | 0.011 | 41 | 11 | 1.7 | 103.1 |
|  | Kazakh-Urban | *q* = 0 | 0.243 | 2.781 | 0.967 | 0.000 | 0.816 | 100 | 0.324 | -0.017 | 2.773 | 0.982 | 0.000 | 59 | 116 | 32.3 | 53.9 |
|  |  | *q* = 1 | 0.124 | 1.125 | 0.815 | 0.004 | 0.907 | 54 | 0.164 | -0.016 | 1.199 | 0.862 | 0.006 | 65 | 13 | 4.1 | 85.1 |
|  |  | *q* = 2 | 0.050 | 0.816 | 0.789 | 0.007 | 0.959 | 54 | 0.077 | -0.009 | 0.835 | 0.859 | 0.006 | 64 | 9 | 2.6 | 95.5 |
|  |  | *q* = 3 | 0.012 | 0.729 | 0.781 | 0.009 | 0.988 | 60 | 0.034 | -0.003 | 0.694 | 0.868 | 0.005 | 62 | 8 | 2.1 | 98.8 |
|  | Mongol-Rural | *q* = 0 | 0.223 | 2.938 | 0.976 | 0.000 | 0.833 | 100 | 0.278 | -0.006 | 2.914 | 0.984 | 0.000 | 46 | 135 | 43.4 | 45.6 |
|  |  | *q* = 1 | 0.015 | 1.189 | 0.675 | 0.006 | 0.989 | 71 | 0.041 | -0.003 | 1.170 | 0.772 | 0.002 | 78 | 326 | 3.5 | 96.5 |
|  |  | *q* = 2 | 0.002 | 0.771 | 0.679 | 0.005 | 0.998 | 72 | 0.018 | -0.001 | 0.752 | 0.768 | 0.002 | 75 | 26 | 2.2 | 98.7 |
|  |  | *q* = 3 | -0.003 | 0.642 | 0.694 | 0.004 | 1.002 | 68 | 0.013 | -0.001 | 0.616 | 0.754 | 0.003 | 72 | 28 | 1.9 | 99.2 |
|  | Mongol-Urban | *q* = 0 | 0.238 | 2.726 | 0.977 | 0.000 | 0.820 | 100 | 0.296 | -0.007 | 2.692 | 0.985 | 0.000 | 46 | 144 | 37.1 | 43.0 |
|  |  | *q* = 1 | 0.026 | 0.987 | 0.700 | 0.006 | 0.981 | 74 | 0.036 | -0.002 | 0.993 | 0.775 | 0.006 | 72 | 15 | 2.9 | 96.0 |
|  |  | *q* = 2 | -0.001 | 0.645 | 0.728 | 0.004 | 1.000 | 62 | -0.004 | 0.000 | 0.644 | 0.790 | 0.003 | 74 | 16 | 1.9 | 101.7 |
|  |  | *q* = 3 | -0.007 | 0.537 | 0.727 | 0.005 | 1.004 | 65 | -0.014 | 0.001 | 0.531 | 0.802 | 0.003 | 73 | 16 | 1.7 | 102.6 |
|  | Tibetan-Rural | *q* = 0 | 0.196 | 2.763 | 0.961 | 0.000 | 0.854 | 100 | 0.268 | -0.008 | 2.713 | 0.975 | 0.000 | 75 | 75 | 31.5 | 51.9 |
|  |  | *q* = 1 | 0.059 | 1.093 | 0.763 | 0.002 | 0.957 | 75 | 0.106 | -0.006 | 1.087 | 0.807 | 0.002 | 66 | 14 | 3.6 | 88.1 |
|  |  | *q* = 2 | 0.039 | 0.809 | 0.697 | 0.005 | 0.972 | 72 | 0.071 | -0.004 | 0.788 | 0.794 | 0.003 | 76 | 18 | 2.5 | 92.4 |
|  |  | *q* = 3 | 0.041 | 0.701 | 0.698 | 0.003 | 0.970 | 61 | 0.064 | -0.004 | 0.704 | 0.778 | 0.003 | 75 | 27 | 2.3 | 93.2 |
|  | Tibeitan-Urban | *q* = 0 | 0.223 | 2.723 | 0.964 | 0.000 | 0.832 | 100 | 0.321 | -0.016 | 2.677 | 0.979 | 0.000 | 50 | 30 | 28.8 | 53.1 |
|  |  | *q* = 1 | 0.061 | 0.963 | 0.790 | 0.005 | 0.953 | 60 | 0.150 | -0.019 | 0.986 | 0.833 | 0.005 | 58 | 9 | 3.2 | 91.7 |
|  |  | *q* = 2 | 0.029 | 0.673 | 0.780 | 0.006 | 0.975 | 51 | 0.085 | -0.013 | 0.690 | 0.822 | 0.007 | 58 | 38 | 2.2 | 102.2 |
|  |  | *q* = 3 | 0.009 | 0.615 | 0.761 | 0.007 | 0.990 | 57 | 0.062 | -0.012 | 0.622 | 0.825 | 0.006 | 63 | 11 | 2.0 | 101.2 |
|  | Uyghur-Rural | *q* = 0 | 0.244 | 2.901 | 0.979 | 0.000 | 0.815 | 100 | 0.337 | -0.021 | 2.877 | 0.991 | 0.000 | 52 | 47 | 34.7 | 54.4 |
|  |  | *q* = 1 | 0.179 | 1.157 | 0.862 | 0.006 | 0.861 | 49 | 0.343 | -0.057 | 1.245 | 0.928 | 0.006 | 48 | 6 | 4.8 | 77.8 |
|  |  | *q* = 2 | 0.065 | 0.931 | 0.832 | 0.010 | 0.942 | 67 | 0.196 | -0.038 | 0.944 | 0.914 | 0.009 | 57 | 8 | 3.1 | 91.9 |
|  |  | *q* = 3 | 0.012 | 0.856 | 0.834 | 0.010 | 0.981 | 71 | 0.081 | -0.020 | 0.853 | 0.913 | 0.009 | 62 | 15 | 2.5 | 103.8 |
|  | Uyghur-Urban | *q* = 0 | 0.201 | 2.981 | 0.967 | 0.000 | 0.850 | 100 | 0.299 | -0.021 | 2.963 | 0.979 | 0.000 | 59 | 48 | 33.5 | 60.3 |
|  |  | *q* = 1 | 0.044 | 1.117 | 0.787 | 0.007 | 0.968 | 70 | 0.071 | -0.007 | 1.124 | 0.860 | 0.006 | 64 | 10 | 3.4 | 92.7 |
|  |  | *q* = 2 | 0.003 | 0.720 | 0.764 | 0.012 | 0.996 | 56 | -0.013 | 0.003 | 0.730 | 0.863 | 0.008 | 62 | 8 | 2.1 | 102.2 |
|  |  | *q* = 3 | -0.009 | 0.598 | 0.762 | 0.014 | 1.005 | 52 | -0.030 | 0.005 | 0.598 | 0.857 | 0.009 | 63 | 7 | 1.8 | 103.3 |
|  | Zhuang-Rural | *q* = 0 | 0.228 | 2.788 | 0.963 | 0.000 | 0.828 | 100 | 0.303 | -0.009 | 2.750 | 0.977 | 0.000 | 46 | 82 | 37.0 | 46.0 |
|  |  | *q* = 1 | 0.009 | 1.005 | 0.664 | 0.004 | 0.992 | 58 | 0.030 | -0.002 | 0.992 | 0.769 | 0.002 | 70 | 29 | 2.9 | 97.5 |
|  |  | *q* = 2 | -0.017 | 0.649 | 0.650 | 0.007 | 1.010 | 60 | -0.003 | -0.001 | 0.630 | 0.751 | 0.004 | 71 | 14 | 1.9 | 103.9 |
|  |  | *q* = 3 | -0.019 | 0.536 | 0.643 | 0.009 | 1.012 | 62 | -0.013 | -0.001 | 0.529 | 0.751 | 0.005 | 72 | 20 | 1.6 | 107.0 |
|  | Zhuang-Urban | *q* = 0 | 0.252 | 2.761 | 0.974 | 0.000 | 0.809 | 100 | 0.326 | -0.010 | 2.727 | 0.981 | 0.000 | 68 | 95 | 37.4 | 44.5 |
|  |  | *q* = 1 | 0.091 | 0.851 | 0.703 | 0.006 | 0.933 | 64 | 0.160 | -0.014 | 0.895 | 0.732 | 0.007 | 62 | 20 | 3.2 | 83.9 |
|  |  | *q* = 2 | 0.025 | 0.525 | 0.689 | 0.007 | 0.980 | 57 | 0.062 | -0.007 | 0.549 | 0.735 | 0.006 | 54 | 12 | 1.9 | 97.9 |
|  |  | *q* = 3 | 0.012 | 0.429 | 0.711 | 0.004 | 0.989 | 53 | 0.038 | -0.004 | 0.429 | 0.741 | 0.006 | 53 | 12 | 1.6 | 99.0 |
| Family | Total | *q* = 0 | 0.255 | 4.524 | 0.992 | 0.000 | 0.806 | 100 | 0.291 | 0.000 | 4.420 | 0.996 | 0.000 | 89 | 7279 | 467.8 | 20.7 |
|  |  | *q* = 1 | 0.024 | 2.813 | 0.574 | 0.000 | 0.983 | 95 | 0.052 | 0.000 | 2.736 | 0.687 | 0.000 | 92 | 164 | 19.1 | 88.2 |
|  |  | *q* = 2 | 0.026 | 2.398 | 0.504 | 0.001 | 0.982 | 93 | 0.057 | 0.000 | 2.311 | 0.636 | 0.000 | 93 | 174 | 12.7 | 88.0 |
|  |  | *q* = 3 | 0.028 | 2.216 | 0.480 | 0.001 | 0.980 | 86 | 0.061 | 0.000 | 2.132 | 0.613 | 0.000 | 91 | 162 | 10.8 | 88.1 |
|  | Rural | *q* = 0 | 0.268 | 4.494 | 0.989 | 0.000 | 0.795 | 100 | 0.316 | -0.001 | 4.381 | 0.993 | 0.000 | 88 | 499 | 379.9 | 24.0 |
|  |  | *q* = 1 | 0.042 | 2.750 | 0.651 | 0.001 | 0.970 | 95 | 0.084 | -0.001 | 2.657 | 0.747 | 0.000 | 89 | 147 | 19.2 | 82.5 |
|  |  | *q* = 2 | 0.053 | 2.288 | 0.611 | 0.001 | 0.962 | 90 | 0.107 | -0.001 | 2.172 | 0.720 | 0.001 | 83 | 123 | 12.8 | 79.3 |
|  |  | *q* = 3 | 0.062 | 2.072 | 0.595 | 0.001 | 0.956 | 85 | 0.118 | -0.001 | 1.959 | 0.705 | 0.001 | 80 | 256 | 10.8 | 77.8 |
|  | Urban | *q* = 0 | 0.280 | 4.394 | 0.993 | 0.000 | 0.786 | 100 | 0.326 | -0.001 | 4.290 | 0.996 | 0.000 | 91 | 764 | 361.8 | 22.9 |
|  |  | *q* = 1 | 0.050 | 2.699 | 0.721 | 0.000 | 0.965 | 96 | 0.097 | -0.001 | 2.598 | 0.813 | 0.000 | 89 | 105 | 18.8 | 79.9 |
|  |  | *q* = 2 | 0.057 | 2.264 | 0.661 | 0.000 | 0.959 | 93 | 0.114 | -0.001 | 2.141 | 0.734 | 0.001 | 87 | 88 | 12.6 | 78.1 |
|  |  | *q* = 3 | 0.062 | 2.080 | 0.631 | 0.001 | 0.955 | 92 | 0.123 | -0.002 | 1.953 | 0.718 | 0.000 | 88 | 92 | 10.8 | 77.0 |
|  | Bai | *q* = 0 | 0.325 | 4.271 | 0.981 | 0.000 | 0.747 | 100 | 0.397 | -0.006 | 4.216 | 0.985 | 0.000 | 49 | 748 | 310.0 | 27.0 |
|  |  | *q* = 1 | 0.079 | 2.565 | 0.763 | 0.002 | 0.943 | 92 | 0.153 | -0.006 | 2.497 | 0.846 | 0.000 | 82 | 81 | 17.4 | 77.0 |
|  |  | *q* = 2 | 0.101 | 2.095 | 0.694 | 0.001 | 0.927 | 85 | 0.196 | -0.008 | 2.024 | 0.789 | 0.001 | 78 | 25 | 11.7 | 75.1 |
|  |  | *q* = 3 | 0.106 | 1.894 | 0.653 | 0.002 | 0.922 | 79 | 0.195 | -0.008 | 1.854 | 0.753 | 0.001 | 81 | 39 | 10.0 | 76.3 |
|  | Han | *q* = 0 | 0.276 | 4.439 | 0.991 | 0.000 | 0.789 | 100 | 0.328 | -0.002 | 4.335 | 0.995 | 0.000 | 80 | 435 | 335.7 | 25.5 |
|  |  | *q* = 1 | 0.048 | 2.623 | 0.725 | 0.001 | 0.966 | 93 | 0.088 | -0.002 | 2.561 | 0.808 | 0.001 | 85 | 69 | 17.0 | 82.8 |
|  |  | *q* = 2 | 0.053 | 2.166 | 0.649 | 0.000 | 0.962 | 90 | 0.094 | -0.002 | 2.110 | 0.748 | 0.000 | 83 | 60 | 11.0 | 83.0 |
|  |  | *q* = 3 | 0.057 | 1.981 | 0.602 | 0.001 | 0.959 | 89 | 0.098 | -0.002 | 1.929 | 0.716 | 0.001 | 81 | 76 | 9.3 | 82.7 |
|  | Kazakh | *q* = 0 | 0.313 | 4.364 | 0.983 | 0.000 | 0.757 | 100 | 0.415 | -0.012 | 4.285 | 0.987 | 0.000 | 52 | 113 | 238.6 | 34.8 |
|  |  | *q* = 1 | 0.135 | 2.615 | 0.755 | 0.004 | 0.899 | 96 | 0.307 | -0.023 | 2.507 | 0.880 | 0.001 | 75 | 21 | 20.6 | 69.6 |
|  |  | *q* = 2 | 0.205 | 2.024 | 0.701 | 0.005 | 0.841 | 80 | 0.364 | -0.025 | 1.997 | 0.831 | 0.002 | 77 | 28 | 13.6 | 67.3 |
|  |  | *q* = 3 | 0.239 | 1.709 | 0.690 | 0.008 | 0.811 | 65 | 0.374 | -0.027 | 1.790 | 0.811 | 0.004 | 75 | 13 | 11.3 | 68.7 |
|  | Mongol | *q* = 0 | 0.285 | 4.504 | 0.982 | 0.000 | 0.780 | 100 | 0.384 | -0.005 | 4.348 | 0.989 | 0.000 | 66 | 131 | 287.8 | 30.6 |
|  |  | *q* = 1 | 0.045 | 2.776 | 0.654 | 0.002 | 0.967 | 81 | 0.099 | -0.004 | 2.710 | 0.760 | 0.001 | 79 | 32 | 18.9 | 87.2 |
|  |  | *q* = 2 | 0.047 | 2.318 | 0.607 | 0.004 | 0.965 | 78 | 0.110 | -0.005 | 2.256 | 0.731 | 0.001 | 79 | 26 | 12.2 | 88.3 |
|  |  | *q* = 3 | 0.049 | 2.107 | 0.602 | 0.005 | 0.963 | 70 | 0.109 | -0.005 | 2.059 | 0.716 | 0.002 | 81 | 24 | 10.0 | 89.7 |
|  | Tibetan | *q* = 0 | 0.297 | 4.310 | 0.990 | 0.000 | 0.771 | 100 | 0.359 | -0.004 | 4.227 | 0.993 | 0.000 | 73 | 200 | 264.3 | 28.7 |
|  |  | *q* = 1 | 0.101 | 2.526 | 0.730 | 0.001 | 0.926 | 83 | 0.195 | -0.008 | 2.451 | 0.818 | 0.001 | 85 | 46 | 18.1 | 74.1 |
|  |  | *q* = 2 | 0.121 | 2.021 | 0.671 | 0.001 | 0.911 | 79 | 0.225 | -0.010 | 1.970 | 0.765 | 0.001 | 84 | 26 | 11.7 | 74.7 |
|  |  | *q* = 3 | 0.126 | 1.800 | 0.627 | 0.004 | 0.906 | 78 | 0.224 | -0.010 | 1.769 | 0.750 | 0.001 | 83 | 66 | 9.7 | 75.2 |
|  | Uyghur | *q* = 0 | 0.276 | 4.503 | 0.989 | 0.000 | 0.789 | 100 | 0.351 | -0.009 | 4.441 | 0.993 | 0.000 | 59 | 1488 | 254.2 | 38.0 |
|  |  | *q* = 1 | 0.088 | 2.812 | 0.818 | 0.002 | 0.936 | 85 | 0.178 | -0.013 | 2.769 | 0.901 | 0.001 | 78 | 16 | 21.3 | 80.0 |
|  |  | *q* = 2 | 0.124 | 2.364 | 0.780 | 0.002 | 0.909 | 85 | 0.252 | -0.019 | 2.297 | 0.880 | 0.001 | 81 | 14 | 15.0 | 73.7 |
|  |  | *q* = 3 | 0.141 | 2.168 | 0.753 | 0.003 | 0.895 | 85 | 0.290 | -0.021 | 2.087 | 0.866 | 0.001 | 80 | 14 | 12.9 | 70.9 |
|  | Zhuang | *q* = 0 | 0.287 | 4.324 | 0.988 | 0.000 | 0.780 | 100 | 0.353 | -0.004 | 4.237 | 0.993 | 0.000 | 82 | 445 | 264.0 | 30.6 |
|  |  | *q* = 1 | 0.084 | 2.605 | 0.760 | 0.001 | 0.939 | 94 | 0.179 | -0.007 | 2.504 | 0.854 | 0.000 | 82 | 103 | 18.7 | 75.1 |
|  |  | *q* = 2 | 0.102 | 2.134 | 0.732 | 0.001 | 0.926 | 92 | 0.210 | -0.008 | 2.019 | 0.819 | 0.000 | 85 | 85 | 12.5 | 71.9 |
|  |  | *q* = 3 | 0.115 | 1.925 | 0.735 | 0.001 | 0.915 | 89 | 0.227 | -0.009 | 1.813 | 0.808 | 0.001 | 81 | 76 | 10.7 | 69.5 |
|  | Bai-Rural | *q* = 0 | 0.321 | 4.315 | 0.972 | 0.000 | 0.750 | 100 | 0.447 | -0.013 | 4.226 | 0.980 | 0.000 | 45 | 74 | 235.8 | 33.4 |
|  |  | *q* = 1 | 0.085 | 2.560 | 0.731 | 0.002 | 0.938 | 86 | 0.150 | -0.008 | 2.526 | 0.809 | 0.001 | 76 | 33 | 16.9 | 80.2 |
|  |  | *q* = 2 | 0.093 | 2.092 | 0.665 | 0.004 | 0.931 | 67 | 0.160 | -0.008 | 2.066 | 0.748 | 0.003 | 67 | 21 | 11.0 | 81.1 |
|  |  | *q* = 3 | 0.077 | 1.920 | 0.644 | 0.004 | 0.941 | 61 | 0.138 | -0.008 | 1.897 | 0.715 | 0.004 | 64 | 17 | 8.7 | 86.3 |
|  | Bai-Urban | *q* = 0 | 0.297 | 4.267 | 0.985 | 0.000 | 0.771 | 100 | 0.377 | -0.016 | 4.267 | 0.992 | 0.000 | 53 | 40 | 172.7 | 43.6 |
|  |  | *q* = 1 | 0.112 | 2.534 | 0.889 | 0.001 | 0.919 | 92 | 0.208 | -0.019 | 2.507 | 0.934 | 0.001 | 78 | 13 | 16.5 | 77.3 |
|  |  | *q* = 2 | 0.165 | 2.074 | 0.880 | 0.000 | 0.877 | 92 | 0.336 | -0.031 | 1.998 | 0.934 | 0.001 | 73 | 13 | 11.9 | 66.7 |
|  |  | *q* = 3 | 0.199 | 1.877 | 0.874 | 0.000 | 0.850 | 92 | 0.383 | -0.034 | 1.805 | 0.928 | 0.001 | 75 | 26 | 11.0 | 61.7 |
|  | Han-Rural | *q* = 0 | 0.298 | 4.398 | 0.989 | 0.000 | 0.770 | 100 | 0.359 | -0.004 | 4.321 | 0.993 | 0.000 | 83 | 227 | 291.7 | 29.1 |
|  |  | *q* = 1 | 0.081 | 2.584 | 0.755 | 0.001 | 0.942 | 85 | 0.146 | -0.005 | 2.523 | 0.822 | 0.001 | 78 | 46 | 17.7 | 78.2 |
|  |  | *q* = 2 | 0.095 | 2.111 | 0.679 | 0.004 | 0.930 | 81 | 0.157 | -0.005 | 2.070 | 0.758 | 0.002 | 76 | 36 | 11.5 | 77.6 |
|  |  | *q* = 3 | 0.104 | 1.920 | 0.639 | 0.005 | 0.923 | 81 | 0.155 | -0.005 | 1.891 | 0.736 | 0.002 | 76 | 29 | 9.7 | 77.3 |
|  | Han-Urban | *q* = 0 | 0.299 | 4.371 | 0.988 | 0.000 | 0.769 | 100 | 0.364 | -0.004 | 4.285 | 0.992 | 0.000 | 71 | 167 | 282.8 | 28.6 |
|  |  | *q* = 1 | 0.058 | 2.572 | 0.738 | 0.002 | 0.959 | 87 | 0.110 | -0.004 | 2.520 | 0.793 | 0.001 | 79 | 51 | 16.3 | 82.9 |
|  |  | *q* = 2 | 0.072 | 2.080 | 0.676 | 0.002 | 0.947 | 79 | 0.129 | -0.005 | 2.031 | 0.732 | 0.001 | 76 | 29 | 10.4 | 81.6 |
|  |  | *q* = 3 | 0.080 | 1.883 | 0.652 | 0.002 | 0.941 | 78 | 0.136 | -0.005 | 1.839 | 0.728 | 0.002 | 75 | 188 | 9.0 | 79.8 |
|  | Kazakh-Rural | *q* = 0 | 0.278 | 4.391 | 0.969 | 0.000 | 0.786 | 100 | 0.458 | -0.045 | 4.368 | 0.983 | 0.000 | 59 | 19 | 154.3 | 52.9 |
|  |  | *q* = 1 | 0.152 | 2.550 | 0.830 | 0.012 | 0.870 | 43 | 0.378 | -0.064 | 2.559 | 0.904 | 0.010 | 56 | 6 | 17.5 | 81.5 |
|  |  | *q* = 2 | 0.158 | 2.024 | 0.852 | 0.007 | 0.841 | 32 | 0.449 | -0.083 | 2.032 | 0.899 | 0.012 | 49 | 18 | 11.3 | 86.6 |
|  |  | *q* = 3 | 0.111 | 1.845 | 0.859 | 0.007 | 0.872 | 29 | 0.414 | -0.082 | 1.806 | 0.895 | 0.013 | 47 | 25 | 9.6 | 86.0 |
|  | Kazakh-Urban | *q* = 0 | 0.358 | 4.322 | 0.986 | 0.000 | 0.717 | 100 | 0.478 | -0.020 | 4.260 | 0.990 | 0.000 | 46 | 68 | 239.3 | 34.2 |
|  |  | *q* = 1 | 0.180 | 2.633 | 0.864 | 0.001 | 0.863 | 96 | 0.374 | -0.038 | 2.560 | 0.926 | 0.002 | 74 | 13 | 21.0 | 68.3 |
|  |  | *q* = 2 | 0.253 | 2.145 | 0.808 | 0.006 | 0.797 | 87 | 0.490 | -0.045 | 2.039 | 0.900 | 0.004 | 70 | 13 | 15.0 | 61.5 |
|  |  | *q* = 3 | 0.304 | 1.891 | 0.803 | 0.005 | 0.753 | 75 | 0.486 | -0.038 | 1.830 | 0.892 | 0.004 | 69 | 33 | 17.4 | 60.9 |
|  | Mongol-Rural | *q* = 0 | 0.289 | 4.623 | 0.989 | 0.000 | 0.777 | 100 | 0.361 | -0.007 | 4.549 | 0.993 | 0.000 | 65 | 120 | 309.6 | 34.1 |
|  |  | *q* = 1 | 0.052 | 2.851 | 0.670 | 0.005 | 0.961 | 59 | 0.063 | -0.003 | 2.882 | 0.765 | 0.004 | 77 | 11 | 20.2 | 92.7 |
|  |  | *q* = 2 | 0.044 | 2.408 | 0.659 | 0.005 | 0.965 | 65 | 0.070 | -0.004 | 2.411 | 0.767 | 0.002 | 76 | 12 | 12.8 | 94.8 |
|  |  | *q* = 3 | 0.031 | 2.218 | 0.662 | 0.004 | 0.973 | 66 | 0.065 | -0.004 | 2.195 | 0.772 | 0.002 | 75 | 15 | 10.3 | 97.1 |
|  | Mongol-Urban | *q* = 0 | 0.306 | 4.163 | 0.992 | 0.000 | 0.763 | 100 | 0.371 | -0.008 | 4.124 | 0.995 | 0.000 | 56 | 144 | 195.7 | 34.1 |
|  |  | *q* = 1 | 0.095 | 2.499 | 0.784 | 0.002 | 0.930 | 81 | 0.180 | -0.013 | 2.468 | 0.850 | 0.003 | 72 | 26 | 16.2 | 78.8 |
|  |  | *q* = 2 | 0.104 | 2.081 | 0.709 | 0.006 | 0.923 | 73 | 0.191 | -0.014 | 2.069 | 0.810 | 0.004 | 71 | 56 | 10.9 | 80.6 |
|  |  | *q* = 3 | 0.129 | 1.857 | 0.711 | 0.005 | 0.903 | 61 | 0.196 | -0.013 | 1.885 | 0.791 | 0.004 | 68 | 14 | 9.3 | 79.8 |
|  | Tibetan-Rural | *q* = 0 | 0.304 | 4.276 | 0.989 | 0.000 | 0.765 | 100 | 0.384 | -0.008 | 4.199 | 0.993 | 0.000 | 67 | 101 | 219.9 | 33.1 |
|  |  | *q* = 1 | 0.130 | 2.426 | 0.770 | 0.002 | 0.904 | 80 | 0.224 | -0.012 | 2.389 | 0.843 | 0.002 | 80 | 30 | 17.2 | 71.9 |
|  |  | *q* = 2 | 0.144 | 1.900 | 0.704 | 0.004 | 0.891 | 74 | 0.273 | -0.018 | 1.880 | 0.807 | 0.002 | 75 | 23 | 10.9 | 72.9 |
|  |  | *q* = 3 | 0.144 | 1.664 | 0.683 | 0.005 | 0.891 | 70 | 0.278 | -0.019 | 1.668 | 0.792 | 0.002 | 74 | 29 | 9.0 | 74.6 |
|  | Tibeitan-Urban | *q* = 0 | 0.296 | 4.363 | 0.985 | 0.000 | 0.771 | 100 | 0.376 | -0.013 | 4.324 | 0.990 | 0.000 | 55 | 177 | 226.9 | 38.9 |
|  |  | *q* = 1 | 0.167 | 2.447 | 0.824 | 0.003 | 0.874 | 77 | 0.252 | -0.019 | 2.463 | 0.878 | 0.003 | 75 | 19 | 17.6 | 73.5 |
|  |  | *q* = 2 | 0.218 | 1.958 | 0.800 | 0.004 | 0.830 | 74 | 0.391 | -0.035 | 1.937 | 0.879 | 0.002 | 69 | 11 | 12.1 | 66.4 |
|  |  | *q* = 3 | 0.242 | 1.754 | 0.792 | 0.003 | 0.810 | 72 | 0.431 | -0.038 | 1.728 | 0.866 | 0.003 | 69 | 10 | 10.4 | 64.4 |
|  | Uyghur-Rural | *q* = 0 | 0.295 | 4.409 | 0.987 | 0.000 | 0.772 | 100 | 0.407 | -0.026 | 4.390 | 0.994 | 0.000 | 50 | 21 | 171.2 | 48.1 |
|  |  | *q* = 1 | 0.168 | 2.735 | 0.869 | 0.006 | 0.875 | 76 | 0.336 | -0.046 | 2.735 | 0.945 | 0.004 | 65 | 11 | 21.6 | 73.8 |
|  |  | *q* = 2 | 0.242 | 2.261 | 0.849 | 0.008 | 0.813 | 76 | 0.487 | -0.068 | 2.256 | 0.934 | 0.006 | 68 | 7 | 15.2 | 66.7 |
|  |  | *q* = 3 | 0.299 | 2.006 | 0.845 | 0.009 | 0.764 | 69 | 0.625 | -0.089 | 1.991 | 0.940 | 0.004 | 62 | 7 | 13.3 | 59.8 |
|  | Uyghur-Urban | *q* = 0 | 0.287 | 4.500 | 0.988 | 0.000 | 0.779 | 100 | 0.379 | -0.017 | 4.459 | 0.993 | 0.000 | 59 | 38 | 206.1 | 43.6 |
|  |  | *q* = 1 | 0.102 | 2.774 | 0.894 | 0.001 | 0.926 | 96 | 0.215 | -0.024 | 2.742 | 0.951 | 0.000 | 85 | 12 | 20.2 | 79.0 |
|  |  | *q* = 2 | 0.139 | 2.327 | 0.852 | 0.003 | 0.898 | 93 | 0.289 | -0.033 | 2.294 | 0.924 | 0.002 | 87 | 60 | 14.2 | 73.8 |
|  |  | *q* = 3 | 0.171 | 2.111 | 0.851 | 0.003 | 0.872 | 84 | 0.356 | -0.041 | 2.076 | 0.926 | 0.002 | 76 | 12 | 12.3 | 70.1 |
|  | Zhuang-Rural | *q* = 0 | 0.297 | 4.331 | 0.986 | 0.000 | 0.771 | 100 | 0.379 | -0.010 | 4.269 | 0.992 | 0.000 | 84 | 123 | 214.8 | 36.8 |
|  |  | *q* = 1 | 0.090 | 2.581 | 0.766 | 0.003 | 0.935 | 88 | 0.186 | -0.013 | 2.528 | 0.866 | 0.001 | 83 | 18 | 17.2 | 79.3 |
|  |  | *q* = 2 | 0.125 | 2.090 | 0.797 | 0.001 | 0.908 | 84 | 0.235 | -0.015 | 2.037 | 0.868 | 0.001 | 79 | 19 | 11.6 | 73.8 |
|  |  | *q* = 3 | 0.143 | 1.889 | 0.802 | 0.002 | 0.894 | 87 | 0.253 | -0.014 | 1.823 | 0.867 | 0.001 | 77 | 20 | 10.0 | 69.7 |
|  | Zhuang-Urban | *q* = 0 | 0.310 | 4.258 | 0.989 | 0.000 | 0.760 | 100 | 0.386 | -0.009 | 4.192 | 0.992 | 0.000 | 57 | 266 | 244.9 | 31.5 |
|  |  | *q* = 1 | 0.126 | 2.543 | 0.805 | 0.001 | 0.907 | 92 | 0.215 | -0.013 | 2.516 | 0.875 | 0.001 | 77 | 24 | 18.6 | 72.7 |
|  |  | *q* = 2 | 0.149 | 2.037 | 0.749 | 0.003 | 0.889 | 82 | 0.236 | -0.015 | 2.053 | 0.824 | 0.002 | 80 | 65 | 12.5 | 72.4 |
|  |  | *q* = 3 | 0.162 | 1.822 | 0.720 | 0.005 | 0.878 | 78 | 0.234 | -0.014 | 1.874 | 0.809 | 0.003 | 80 | 15 | 10.0 | 73.7 |
| Genus | Total | *q* = 0 | 0.288 | 5.137 | 0.994 | 0.000 | 0.779 | 100 | 0.328 | 0.000 | 5.020 | 0.997 | 0.000 | 94 | 1093 | 984.5 | 17.6 |
|  |  | *q* = 1 | 0.040 | 3.357 | 0.657 | 0.000 | 0.972 | 100 | 0.087 | -0.001 | 3.223 | 0.791 | 0.000 | 96 | 183 | 35.8 | 80.9 |
|  |  | *q* = 2 | 0.045 | 2.831 | 0.631 | 0.000 | 0.968 | 99 | 0.101 | -0.001 | 2.672 | 0.771 | 0.000 | 97 | 201 | 21.7 | 78.9 |
|  |  | *q* = 3 | 0.050 | 2.619 | 0.632 | 0.000 | 0.965 | 99 | 0.111 | -0.001 | 2.446 | 0.772 | 0.000 | 98 | 215 | 18.1 | 76.9 |
|  | Rural | *q* = 0 | 0.296 | 5.125 | 0.992 | 0.000 | 0.772 | 100 | 0.341 | -0.001 | 5.017 | 0.995 | 0.000 | 86 | 13123 | 947.7 | 19.4 |
|  |  | *q* = 1 | 0.051 | 3.324 | 0.712 | 0.000 | 0.964 | 97 | 0.101 | -0.001 | 3.210 | 0.809 | 0.000 | 91 | 150 | 35.6 | 78.8 |
|  |  | *q* = 2 | 0.058 | 2.788 | 0.677 | 0.000 | 0.959 | 98 | 0.120 | -0.001 | 2.648 | 0.779 | 0.000 | 89 | 1121 | 21.6 | 76.8 |
|  |  | *q* = 3 | 0.068 | 2.561 | 0.687 | 0.000 | 0.951 | 98 | 0.136 | -0.002 | 2.410 | 0.783 | 0.000 | 90 | 566 | 18.1 | 73.2 |
|  | Urban | *q* = 0 | 0.309 | 5.023 | 0.994 | 0.000 | 0.761 | 100 | 0.353 | -0.001 | 4.923 | 0.996 | 0.000 | 84 | 659 | 829.8 | 18.7 |
|  |  | *q* = 1 | 0.060 | 3.274 | 0.745 | 0.000 | 0.957 | 98 | 0.120 | -0.002 | 3.148 | 0.851 | 0.000 | 96 | 117 | 35.1 | 76.3 |
|  |  | *q* = 2 | 0.067 | 2.743 | 0.712 | 0.001 | 0.952 | 95 | 0.142 | -0.002 | 2.588 | 0.824 | 0.000 | 95 | 87 | 21.4 | 74.4 |
|  |  | *q* = 3 | 0.071 | 2.530 | 0.690 | 0.001 | 0.949 | 95 | 0.151 | -0.002 | 2.365 | 0.810 | 0.000 | 96 | 89 | 17.6 | 73.4 |
|  | Bai | *q* = 0 | 0.340 | 4.927 | 0.986 | 0.000 | 0.733 | 100 | 0.420 | -0.006 | 4.841 | 0.989 | 0.000 | 59 | 140 | 553.1 | 26.4 |
|  |  | *q* = 1 | 0.110 | 3.106 | 0.809 | 0.000 | 0.920 | 95 | 0.206 | -0.008 | 3.007 | 0.872 | 0.000 | 88 | 48 | 32.9 | 70.2 |
|  |  | *q* = 2 | 0.134 | 2.545 | 0.808 | 0.000 | 0.901 | 95 | 0.260 | -0.009 | 2.399 | 0.877 | 0.000 | 86 | 45 | 20.4 | 64.1 |
|  |  | *q* = 3 | 0.154 | 2.296 | 0.824 | 0.000 | 0.886 | 94 | 0.283 | -0.010 | 2.147 | 0.885 | 0.000 | 85 | 73 | 17.0 | 60.5 |
|  | Han | *q* = 0 | 0.312 | 5.048 | 0.992 | 0.000 | 0.758 | 100 | 0.364 | -0.002 | 4.950 | 0.995 | 0.000 | 86 | 721 | 735.7 | 21.9 |
|  |  | *q* = 1 | 0.068 | 3.213 | 0.788 | 0.000 | 0.951 | 98 | 0.138 | -0.003 | 3.092 | 0.883 | 0.000 | 94 | 62 | 33.2 | 75.9 |
|  |  | *q* = 2 | 0.079 | 2.643 | 0.768 | 0.001 | 0.943 | 97 | 0.164 | -0.003 | 2.497 | 0.858 | 0.001 | 91 | 57 | 19.7 | 72.9 |
|  |  | *q* = 3 | 0.087 | 2.409 | 0.763 | 0.000 | 0.937 | 96 | 0.179 | -0.004 | 2.249 | 0.856 | 0.000 | 90 | 63 | 16.1 | 70.5 |
|  | Kazakh | *q* = 0 | 0.351 | 4.966 | 0.986 | 0.000 | 0.724 | 100 | 0.433 | -0.010 | 4.917 | 0.989 | 0.000 | 60 | 366 | 548.6 | 29.9 |
|  |  | *q* = 1 | 0.168 | 3.141 | 0.889 | 0.000 | 0.874 | 100 | 0.322 | -0.020 | 3.043 | 0.936 | 0.000 | 86 | 28 | 37.8 | 63.0 |
|  |  | *q* = 2 | 0.219 | 2.587 | 0.873 | 0.000 | 0.832 | 100 | 0.441 | -0.029 | 2.429 | 0.929 | 0.000 | 81 | 30 | 25.6 | 54.1 |
|  |  | *q* = 3 | 0.249 | 2.351 | 0.871 | 0.000 | 0.807 | 100 | 0.500 | -0.032 | 2.171 | 0.928 | 0.000 | 75 | 24 | 21.9 | 50.2 |
|  | Mongol | *q* = 0 | 0.337 | 5.073 | 0.984 | 0.000 | 0.736 | 100 | 0.426 | -0.005 | 4.939 | 0.988 | 0.000 | 68 | 524 | 732.4 | 24.1 |
|  |  | *q* = 1 | 0.076 | 3.249 | 0.710 | 0.002 | 0.945 | 84 | 0.139 | -0.005 | 3.187 | 0.801 | 0.000 | 84 | 47 | 33.9 | 79.3 |
|  |  | *q* = 2 | 0.083 | 2.695 | 0.705 | 0.001 | 0.939 | 84 | 0.166 | -0.006 | 2.607 | 0.801 | 0.000 | 76 | 31 | 20.1 | 77.2 |
|  |  | *q* = 3 | 0.091 | 2.465 | 0.693 | 0.001 | 0.934 | 85 | 0.166 | -0.006 | 2.397 | 0.773 | 0.002 | 82 | 33 | 16.3 | 77.1 |
|  | Tibetan | *q* = 0 | 0.316 | 4.959 | 0.988 | 0.000 | 0.755 | 100 | 0.389 | -0.005 | 4.873 | 0.994 | 0.000 | 80 | 485 | 553.8 | 28.1 |
|  |  | *q* = 1 | 0.118 | 2.955 | 0.712 | 0.002 | 0.913 | 88 | 0.213 | -0.008 | 2.871 | 0.808 | 0.000 | 82 | 37 | 29.2 | 70.7 |
|  |  | *q* = 2 | 0.133 | 2.232 | 0.632 | 0.004 | 0.899 | 66 | 0.213 | -0.009 | 2.223 | 0.728 | 0.002 | 74 | 371 | 15.5 | 75.1 |
|  |  | *q* = 3 | 0.101 | 2.022 | 0.595 | 0.006 | 0.921 | 67 | 0.180 | -0.009 | 2.023 | 0.710 | 0.004 | 70 | 25 | 11.4 | 83.8 |
|  | Uyghur | *q* = 0 | 0.317 | 5.124 | 0.993 | 0.000 | 0.754 | 100 | 0.386 | -0.008 | 5.062 | 0.995 | 0.000 | 61 | 136 | 563.6 | 30.8 |
|  |  | *q* = 1 | 0.112 | 3.308 | 0.842 | 0.001 | 0.918 | 96 | 0.229 | -0.016 | 3.238 | 0.911 | 0.001 | 85 | 25 | 37.6 | 73.7 |
|  |  | *q* = 2 | 0.140 | 2.749 | 0.825 | 0.001 | 0.897 | 95 | 0.320 | -0.024 | 2.635 | 0.905 | 0.001 | 80 | 22 | 23.5 | 67.4 |
|  |  | *q* = 3 | 0.155 | 2.523 | 0.817 | 0.001 | 0.885 | 96 | 0.378 | -0.030 | 2.371 | 0.912 | 0.000 | 77 | 19 | 19.5 | 63.7 |
|  | Zhuang | *q* = 0 | 0.300 | 4.977 | 0.990 | 0.000 | 0.768 | 100 | 0.362 | -0.004 | 4.898 | 0.993 | 0.000 | 88 | 203 | 507.3 | 29.2 |
|  |  | *q* = 1 | 0.097 | 3.153 | 0.768 | 0.000 | 0.930 | 99 | 0.191 | -0.007 | 3.049 | 0.870 | 0.000 | 86 | 91 | 33.4 | 71.8 |
|  |  | *q* = 2 | 0.112 | 2.578 | 0.698 | 0.002 | 0.918 | 93 | 0.229 | -0.009 | 2.465 | 0.829 | 0.001 | 80 | 37 | 20.0 | 70.1 |
|  |  | *q* = 3 | 0.122 | 2.334 | 0.676 | 0.002 | 0.910 | 88 | 0.235 | -0.009 | 2.236 | 0.808 | 0.000 | 82 | 33 | 16.1 | 70.1 |
|  | Bai-Rural | *q* = 0 | 0.333 | 4.962 | 0.978 | 0.000 | 0.739 | 100 | 0.446 | -0.011 | 4.860 | 0.986 | 0.000 | 69 | 89 | 484.5 | 31.2 |
|  |  | *q* = 1 | 0.121 | 3.137 | 0.799 | 0.001 | 0.911 | 93 | 0.234 | -0.013 | 3.057 | 0.878 | 0.000 | 84 | 31 | 34.1 | 70.4 |
|  |  | *q* = 2 | 0.138 | 2.599 | 0.806 | 0.001 | 0.898 | 93 | 0.273 | -0.015 | 2.504 | 0.877 | 0.000 | 84 | 47 | 21.1 | 67.0 |
|  |  | *q* = 3 | 0.150 | 2.373 | 0.809 | 0.001 | 0.889 | 94 | 0.298 | -0.016 | 2.256 | 0.884 | 0.000 | 80 | 29 | 17.3 | 64.2 |
|  | Bai-Urban | *q* = 0 | 0.343 | 4.891 | 0.991 | 0.000 | 0.731 | 100 | 0.426 | -0.015 | 4.862 | 0.994 | 0.000 | 61 | 105 | 418.9 | 34.3 |
|  |  | *q* = 1 | 0.139 | 3.004 | 0.863 | 0.002 | 0.898 | 86 | 0.203 | -0.014 | 2.998 | 0.909 | 0.003 | 87 | 102 | 28.0 | 75.2 |
|  |  | *q* = 2 | 0.196 | 2.368 | 0.826 | 0.004 | 0.851 | 79 | 0.277 | -0.019 | 2.367 | 0.881 | 0.004 | 76 | 12 | 16.5 | 70.6 |
|  |  | *q* = 3 | 0.225 | 2.099 | 0.815 | 0.004 | 0.826 | 78 | 0.297 | -0.017 | 2.099 | 0.878 | 0.004 | 74 | 11 | 13.6 | 68.3 |
|  | Han-Rural | *q* = 0 | 0.331 | 5.021 | 0.990 | 0.000 | 0.741 | 100 | 0.404 | -0.005 | 4.926 | 0.993 | 0.000 | 74 | 304 | 638.7 | 25.6 |
|  |  | *q* = 1 | 0.105 | 3.163 | 0.830 | 0.000 | 0.924 | 99 | 0.203 | -0.007 | 3.049 | 0.897 | 0.000 | 87 | 58 | 34.5 | 69.4 |
|  |  | *q* = 2 | 0.123 | 2.575 | 0.809 | 0.000 | 0.910 | 100 | 0.242 | -0.009 | 2.438 | 0.884 | 0.000 | 88 | 48 | 20.5 | 65.6 |
|  |  | *q* = 3 | 0.136 | 2.330 | 0.802 | 0.000 | 0.900 | 100 | 0.259 | -0.009 | 2.189 | 0.876 | 0.000 | 88 | 63 | 16.9 | 62.9 |
|  | Han-Urban | *q* = 0 | 0.325 | 4.994 | 0.989 | 0.000 | 0.747 | 100 | 0.392 | -0.004 | 4.896 | 0.992 | 0.000 | 78 | 909 | 668.6 | 24.6 |
|  |  | *q* = 1 | 0.084 | 3.141 | 0.776 | 0.001 | 0.939 | 97 | 0.174 | -0.006 | 3.032 | 0.864 | 0.001 | 93 | 51 | 31.3 | 74.6 |
|  |  | *q* = 2 | 0.096 | 2.581 | 0.732 | 0.001 | 0.930 | 97 | 0.216 | -0.008 | 2.432 | 0.833 | 0.001 | 89 | 37 | 18.8 | 71.1 |
|  |  | *q* = 3 | 0.104 | 2.353 | 0.728 | 0.001 | 0.924 | 96 | 0.231 | -0.009 | 2.196 | 0.829 | 0.001 | 88 | 35 | 15.4 | 69.4 |
|  | Kazakh-Rural | *q* = 0 | 0.322 | 5.016 | 0.973 | 0.000 | 0.747 | 100 | 0.488 | -0.040 | 4.987 | 0.985 | 0.000 | 60 | 23 | 325.6 | 48.1 |
|  |  | *q* = 1 | 0.207 | 3.091 | 0.870 | 0.006 | 0.839 | 87 | 0.353 | -0.040 | 3.094 | 0.935 | 0.005 | 71 | 9 | 32.8 | 72.1 |
|  |  | *q* = 2 | 0.269 | 2.531 | 0.852 | 0.008 | 0.782 | 80 | 0.432 | -0.045 | 2.526 | 0.925 | 0.007 | 67 | 7 | 20.8 | 67.6 |
|  |  | *q* = 3 | 0.300 | 2.300 | 0.849 | 0.008 | 0.754 | 77 | 0.426 | -0.037 | 2.308 | 0.916 | 0.009 | 69 | 16 | 17.9 | 64.8 |
|  | Kazakh-Urban | *q* = 0 | 0.354 | 5.002 | 0.984 | 0.000 | 0.721 | 100 | 0.498 | -0.023 | 4.913 | 0.988 | 0.000 | 52 | 58 | 450.9 | 34.2 |
|  |  | *q* = 1 | 0.215 | 3.110 | 0.935 | 0.000 | 0.836 | 100 | 0.415 | -0.038 | 3.029 | 0.974 | 0.000 | 78 | 15 | 37.6 | 59.7 |
|  |  | *q* = 2 | 0.282 | 2.527 | 0.921 | 0.000 | 0.778 | 100 | 0.564 | -0.054 | 2.419 | 0.966 | 0.000 | 77 | 18 | 24.4 | 52.2 |
|  |  | *q* = 3 | 0.317 | 2.278 | 0.913 | 0.000 | 0.747 | 100 | 0.613 | -0.057 | 2.165 | 0.960 | 0.000 | 77 | 17 | 20.7 | 48.9 |
|  | Mongol-Rural | *q* = 0 | 0.309 | 5.297 | 0.990 | 0.000 | 0.760 | 100 | 0.375 | -0.007 | 5.238 | 0.992 | 0.000 | 60 | 193 | 677.3 | 30.6 |
|  |  | *q* = 1 | 0.092 | 3.303 | 0.697 | 0.005 | 0.932 | 68 | 0.165 | -0.010 | 3.274 | 0.804 | 0.004 | 66 | 15 | 35.9 | 81.5 |
|  |  | *q* = 2 | 0.118 | 2.690 | 0.700 | 0.005 | 0.911 | 71 | 0.205 | -0.012 | 2.665 | 0.787 | 0.005 | 74 | 16 | 21.1 | 78.6 |
|  |  | *q* = 3 | 0.146 | 2.401 | 0.720 | 0.003 | 0.889 | 69 | 0.225 | -0.012 | 2.393 | 0.790 | 0.004 | 73 | 25 | 17.0 | 75.1 |
|  | Mongol-Urban | *q* = 0 | 0.328 | 4.781 | 0.993 | 0.000 | 0.744 | 100 | 0.387 | -0.007 | 4.744 | 0.996 | 0.000 | 63 | 143 | 411.9 | 30.8 |
|  |  | *q* = 1 | 0.127 | 2.994 | 0.852 | 0.001 | 0.907 | 95 | 0.231 | -0.015 | 2.940 | 0.899 | 0.001 | 79 | 24 | 29.1 | 71.0 |
|  |  | *q* = 2 | 0.143 | 2.465 | 0.809 | 0.001 | 0.895 | 92 | 0.265 | -0.017 | 2.403 | 0.880 | 0.001 | 87 | 25 | 17.9 | 69.3 |
|  |  | *q* = 3 | 0.156 | 2.241 | 0.785 | 0.002 | 0.884 | 90 | 0.297 | -0.020 | 2.177 | 0.859 | 0.003 | 85 | 20 | 14.8 | 68.3 |
|  | Tibetan-Rural | *q* = 0 | 0.338 | 4.855 | 0.987 | 0.000 | 0.735 | 100 | 0.446 | -0.010 | 4.743 | 0.992 | 0.000 | 71 | 77 | 429.2 | 29.6 |
|  |  | *q* = 1 | 0.167 | 2.780 | 0.772 | 0.003 | 0.874 | 89 | 0.330 | -0.019 | 2.683 | 0.849 | 0.001 | 81 | 20 | 27.5 | 64.8 |
|  |  | *q* = 2 | 0.176 | 2.020 | 0.716 | 0.003 | 0.864 | 77 | 0.340 | -0.022 | 1.972 | 0.816 | 0.001 | 77 | 18 | 13.7 | 69.3 |
|  |  | *q* = 3 | 0.136 | 1.785 | 0.690 | 0.004 | 0.893 | 76 | 0.297 | -0.022 | 1.763 | 0.792 | 0.002 | 76 | 15 | 10.1 | 77.7 |
|  | Tibeitan-Urban | *q* = 0 | 0.357 | 4.952 | 0.984 | 0.000 | 0.718 | 100 | 0.469 | -0.020 | 4.915 | 0.990 | 0.000 | 67 | 125 | 440.1 | 35.0 |
|  |  | *q* = 1 | 0.231 | 2.808 | 0.812 | 0.004 | 0.823 | 77 | 0.387 | -0.034 | 2.807 | 0.887 | 0.003 | 68 | 20 | 29.6 | 63.7 |
|  |  | *q* = 2 | 0.305 | 2.045 | 0.792 | 0.005 | 0.756 | 64 | 0.469 | -0.042 | 2.126 | 0.858 | 0.006 | 66 | 13 | 16.8 | 62.7 |
|  |  | *q* = 3 | 0.298 | 1.797 | 0.781 | 0.005 | 0.757 | 64 | 0.480 | -0.044 | 1.868 | 0.862 | 0.004 | 59 | 10 | 13.1 | 64.6 |
|  | Uyghur-Rural | *q* = 0 | 0.320 | 5.060 | 0.994 | 0.000 | 0.751 | 100 | 0.413 | -0.020 | 5.022 | 0.997 | 0.000 | 53 | 72 | 406.8 | 40.1 |
|  |  | *q* = 1 | 0.210 | 3.181 | 0.894 | 0.003 | 0.842 | 77 | 0.381 | -0.050 | 3.197 | 0.945 | 0.004 | 70 | 8 | 36.6 | 69.7 |
|  |  | *q* = 2 | 0.275 | 2.607 | 0.883 | 0.004 | 0.785 | 85 | 0.534 | -0.071 | 2.599 | 0.948 | 0.004 | 72 | 22 | 24.0 | 60.8 |
|  |  | *q* = 3 | 0.318 | 2.353 | 0.889 | 0.003 | 0.747 | 89 | 0.567 | -0.071 | 2.364 | 0.947 | 0.004 | 77 | 9 | 19.8 | 58.6 |
|  | Uyghur-Urban | *q* = 0 | 0.329 | 5.146 | 0.989 | 0.000 | 0.743 | 100 | 0.435 | -0.021 | 5.109 | 0.995 | 0.000 | 68 | 53 | 446.6 | 39.8 |
|  |  | *q* = 1 | 0.139 | 3.255 | 0.899 | 0.001 | 0.898 | 91 | 0.275 | -0.030 | 3.229 | 0.944 | 0.001 | 85 | 13 | 35.8 | 73.8 |
|  |  | *q* = 2 | 0.172 | 2.675 | 0.860 | 0.003 | 0.872 | 89 | 0.356 | -0.042 | 2.653 | 0.924 | 0.002 | 86 | 16 | 21.6 | 70.5 |
|  |  | *q* = 3 | 0.190 | 2.432 | 0.837 | 0.004 | 0.857 | 89 | 0.402 | -0.049 | 2.408 | 0.910 | 0.003 | 88 | 24 | 17.7 | 68.2 |
|  | Zhuang-Rural | *q* = 0 | 0.328 | 4.952 | 0.987 | 0.000 | 0.744 | 100 | 0.421 | -0.011 | 4.876 | 0.993 | 0.000 | 86 | 107 | 443.7 | 32.8 |
|  |  | *q* = 1 | 0.121 | 3.035 | 0.802 | 0.001 | 0.912 | 88 | 0.248 | -0.017 | 2.963 | 0.875 | 0.001 | 75 | 17 | 29.9 | 72.9 |
|  |  | *q* = 2 | 0.154 | 2.424 | 0.750 | 0.002 | 0.885 | 80 | 0.280 | -0.018 | 2.390 | 0.821 | 0.003 | 78 | 18 | 17.9 | 71.1 |
|  |  | *q* = 3 | 0.179 | 2.154 | 0.746 | 0.002 | 0.865 | 75 | 0.302 | -0.019 | 2.140 | 0.814 | 0.002 | 72 | 54 | 15.0 | 67.9 |
|  | Zhuang-Urban | *q* = 0 | 0.313 | 4.906 | 0.989 | 0.000 | 0.757 | 100 | 0.400 | -0.010 | 4.837 | 0.992 | 0.000 | 61 | 1018 | 492.2 | 33.4 |
|  |  | *q* = 1 | 0.151 | 3.117 | 0.905 | 0.000 | 0.889 | 100 | 0.271 | -0.016 | 3.040 | 0.947 | 0.000 | 76 | 33 | 35.9 | 64.0 |
|  |  | *q* = 2 | 0.167 | 2.541 | 0.842 | 0.001 | 0.875 | 100 | 0.316 | -0.019 | 2.446 | 0.905 | 0.000 | 81 | 34 | 21.0 | 62.3 |
|  |  | *q* = 3 | 0.179 | 2.295 | 0.810 | 0.001 | 0.866 | 98 | 0.337 | -0.021 | 2.200 | 0.884 | 0.001 | 85 | 41 | 17.1 | 61.1 |

**Table S2**. The *p*-value of the permutation tests for the differences in the parameters of the DAR models for all pair-wise comparisons of Chinese gut microbiome datasets of four Schemes (1A-2B)

| **Scheme-1A** | | | | | | | | | | |
| --- | --- | --- | --- | --- | --- | --- | --- | --- | --- | --- |
| **Diversity order** | **Taxon** | **Cohort** | **PL** | | **PLEC** | | | | | |
|  |  |  | ***z*** | **ln(*c*)** | ***z*** | ***d*** | **ln(*c*)** | ***A_max_*** | ***D_max_*** | ***LGD*** |
| *q* = 0 | Phylum | Rural *vs.* Urban | 0.480 | 0.620 | 0.657 | 0.846 | 0.685 | 0.727 | 0.726 | 0.673 |
|  | Family |  | 0.749 | 0.540 | 0.897 | 0.927 | 0.691 | 0.622 | 0.787 | 0.849 |
|  | Genus |  | 0.727 | 0.567 | 0.872 | 0.940 | 0.683 | 0.034 | 0.488 | 0.900 |
|  | Species |  | 0.964 | 0.600 | 0.961 | 0.861 | 0.698 | 0.390 | 0.371 | 0.860 |
|  | (%) With significant differences | | 0 | 0 | 0 | 0 | 0 | 25% (1/4) | 0 | 0 |
| *q* = 1 | Phylum | Rural *vs.* Urban | 0.970 | 0.805 | 0.964 | 0.972 | 0.871 | 0.160 | 0.342 | 0.919 |
|  | Family |  | 0.844 | 0.773 | 0.853 | 0.838 | 0.813 | 0.532 | 0.699 | 0.856 |
|  | Genus |  | 0.814 | 0.778 | 0.815 | 0.720 | 0.816 | 0.517 | 0.774 | 0.864 |
|  | Species |  | 0.953 | 0.893 | 0.950 | 0.942 | 0.977 | 0.891 | 0.568 | 0.990 |
|  | (%) With significant differences | | 0 | 0 | 0 | 0 | 0 | 0 | 0 | 0 |
| *q* = 2 | Phylum | Rural *vs.* Urban | 0.967 | 0.774 | 0.975 | 0.979 | 0.814 | 0.651 | 0.270 | 0.920 |
|  | Family |  | 0.939 | 0.916 | 0.954 | 0.901 | 0.935 | 0.610 | 0.796 | 0.942 |
|  | Genus |  | 0.860 | 0.840 | 0.843 | 0.733 | 0.841 | 0.024 | 0.850 | 0.896 |
|  | Species |  | 0.993 | 0.918 | 0.985 | 0.897 | 0.932 | 0.417 | 0.622 | 0.959 |
|  | (%) With significant differences | | 0 | 0 | 0 | 0 | 0 | 25% (1/4) | 0 | 0 |
| *q* = 3 | Phylum | Rural *vs.* Urban | 0.976 | 0.763 | 0.996 | 0.991 | 0.789 | 0.198 | 0.298 | 0.981 |
|  | Family |  | 0.999 | 0.978 | 0.969 | 0.935 | 0.992 | 0.218 | 0.969 | 0.971 |
|  | Genus |  | 0.960 | 0.893 | 0.886 | 0.757 | 0.893 | 0.055 | 0.599 | 0.990 |
|  | Species |  | 0.984 | 0.893 | 0.983 | 0.909 | 0.868 | 0.856 | 0.647 | 0.969 |
|  | (%) With significant differences | | 0 | 0 | 0 | 0 | 0 | 0 | 0 | 0 |
| **Scheme-1B** | | | | | | | | | | |
| **Diversity order** | **Taxon** | **Cohort** | **PL** | | **PLEC** | | | | | |
|  |  |  | ***z*** | **ln(*c*)** | ***z*** | ***d*** | **ln(*c*)** | ***A_max_*** | ***D_max_*** | ***LGD*** |
| *q*  = 0 | Phylum | Bai-Rural *vs.* Bai-Urban | 0.868 | 0.909 | 0.975 | 0.894 | 0.913 | 0.597 | 0.983 | 0.913 |
|  |  | Han-Rural *vs.* Han-Urban | 0.712 | 0.618 | 0.902 | 0.830 | 0.664 | 0.262 | 0.558 | 0.509 |
|  |  | Kazakh-Rural *vs.* Kazakh-Urban | 0.667 | 0.689 | 0.893 | 0.598 | 0.620 | 0.134 | 0.546 | 0.567 |
|  |  | Mongol-Rural *vs.* Mongol-Urban | 0.846 | 0.303 | 0.907 | 0.948 | 0.356 | 0.901 | 0.454 | 0.871 |
|  |  | Tibetan-Rural *vs.* Tibetan-Urban | 0.739 | 0.839 | 0.738 | 0.739 | 0.868 | 0.581 | 0.786 | 0.954 |
|  |  | Uyghur-Rural *vs.* Uyghur-Urban | 0.657 | 0.672 | 0.852 | 1.000 | 0.657 | 0.970 | 0.826 | 0.715 |
|  |  | Zhuang-Rural *vs.* Zhuang-Urban | 0.715 | 0.880 | 0.886 | 0.919 | 0.909 | 0.861 | 0.961 | 0.920 |
|  | (%) With significant differences | | 0 | 0 | 0 | 0 | 0 | 0 | 0 | 0 |
|  | Family | Bai-Rural *vs.* Bai-Urban | 0.818 | 0.854 | 0.755 | 0.915 | 0.859 | 0.735 | 0.440 | 0.581 |
|  |  | Han-Rural *vs.* Han-Urban | 0.979 | 0.910 | 0.973 | 0.974 | 0.898 | 0.677 | 0.829 | 0.935 |
|  |  | Kazakh-Rural *vs.* Kazakh-Urban | 0.470 | 0.800 | 0.940 | 0.604 | 0.696 | 0.324 | 0.198 | 0.267 |
|  |  | Mongol-Rural *vs.* Mongol-Urban | 0.870 | 0.121 | 0.958 | 0.945 | 0.223 | 0.713 | 0.114 | 0.993 |
|  |  | Tibetan-Rural *vs.* Tibetan-Urban | 0.902 | 0.689 | 0.973 | 0.794 | 0.621 | 0.485 | 0.933 | 0.625 |
|  |  | Uyghur-Rural *vs.* Uyghur-Urban | 0.929 | 0.636 | 0.873 | 0.817 | 0.735 | 0.724 | 0.492 | 0.781 |
|  |  | Zhuang-Rural *vs.* Zhuang-Urban | 0.852 | 0.712 | 0.966 | 0.911 | 0.717 | 0.285 | 0.455 | 0.623 |
|  | (%) With significant differences | | 0 | 0 | 0 | 0 | 0 | 0 | 0 | 0 |
|  | Genus | Bai-Rural *vs.* Bai-Urban | 0.908 | 0.789 | 0.931 | 0.905 | 0.996 | 0.873 | 0.759 | 0.847 |
|  |  | Han-Rural *vs.* Han-Urban | 0.915 | 0.910 | 0.920 | 0.891 | 0.916 | 0.139 | 0.750 | 0.916 |
|  |  | Kazakh-Rural *vs.* Kazakh-Urban | 0.807 | 0.962 | 0.961 | 0.745 | 0.806 | 0.426 | 0.297 | 0.404 |
|  |  | Mongol-Rural *vs.* Mongol-Urban | 0.858 | 0.119 | 0.951 | 0.968 | 0.206 | 0.533 | 0.134 | 0.990 |
|  |  | Tibetan-Rural *vs.* Tibetan-Urban | 0.844 | 0.699 | 0.907 | 0.719 | 0.543 | 0.622 | 0.962 | 0.689 |
|  |  | Uyghur-Rural *vs.* Uyghur-Urban | 0.909 | 0.644 | 0.901 | 0.982 | 0.654 | 0.721 | 0.707 | 0.980 |
|  |  | Zhuang-Rural *vs.* Zhuang-Urban | 0.832 | 0.818 | 0.904 | 0.952 | 0.864 | 0.041 | 0.539 | 0.931 |
|  | (%) With significant differences | | 0 | 0 | 0 | 0 | 0 | 14.3% (1/7) | 0 | 0 |
|  | Species | Bai-Rural *vs.* Bai-Urban | 0.360 | 0.567 | 0.464 | 0.460 | 0.750 | 0.384 | 0.584 | 0.798 |
|  |  | Han-Rural *vs.* Han-Urban | 0.749 | 0.984 | 0.912 | 0.930 | 0.933 | 0.118 | 0.418 | 0.940 |
|  |  | Kazakh-Rural *vs.* Kazakh-Urban | 0.942 | 0.998 | 0.796 | 0.660 | 0.945 | 0.716 | 0.515 | 0.750 |
|  |  | Mongol-Rural *vs.* Mongol-Urban | 0.826 | 0.102 | 0.989 | 0.966 | 0.226 | 0.095 | 0.245 | 0.785 |
|  |  | Tibetan-Rural *vs.* Tibetan-Urban | 0.349 | 0.580 | 0.404 | 0.758 | 0.366 | 0.670 | 0.192 | 0.040 |
|  |  | Uyghur-Rural *vs.* Uyghur-Urban | 0.968 | 0.553 | 0.958 | 0.998 | 0.601 | 0.689 | 0.688 | 0.978 |
|  |  | Zhuang-Rural *vs.* Zhuang-Urban | 0.982 | 0.602 | 0.874 | 0.941 | 0.806 | 0.100 | 0.885 | 0.994 |
|  | (%) With significant differences | | 0 | 0 | 0 | 0 | 0 | 0 | 0 | 14.3% (1/7) |
| *q*  = 1 | Phylum | Bai-Rural *vs.* Bai-Urban | 0.865 | 0.955 | 0.995 | 0.904 | 0.836 | 0.561 | 0.951 | 0.996 |
|  |  | Han-Rural *vs.* Han-Urban | 0.878 | 0.704 | 0.921 | 0.891 | 0.700 | 0.825 | 0.274 | 0.971 |
|  |  | Kazakh-Rural *vs.* Kazakh-Urban | 0.654 | 0.886 | 0.887 | 0.975 | 0.723 | 0.408 | 0.258 | 0.805 |
|  |  | Mongol-Rural *vs.* Mongol-Urban | 0.921 | 0.458 | 0.982 | 0.949 | 0.571 | 0.027 | 0.129 | 0.980 |
|  |  | Tibetan-Rural *vs.* Tibetan-Urban | 0.989 | 0.713 | 0.854 | 0.711 | 0.771 | 0.747 | 0.608 | 0.886 |
|  |  | Uyghur-Rural *vs.* Uyghur-Urban | 0.404 | 0.904 | 0.456 | 0.497 | 0.749 | 0.583 | 0.109 | 0.609 |
|  |  | Zhuang-Rural *vs.* Zhuang-Urban | 0.453 | 0.596 | 0.588 | 0.661 | 0.743 | 0.506 | 0.613 | 0.582 |
|  | (%) With significant differences | | 0 | 0 | 0 | 0 | 0 | 14.3% (1/7) | 0 | 0 |
|  | Family | Bai-Rural *vs.* Bai-Urban | 0.781 | 0.939 | 0.788 | 0.715 | 0.943 | 0.388 | 0.873 | 0.904 |
|  |  | Han-Rural *vs.* Han-Urban | 0.730 | 0.955 | 0.794 | 0.843 | 0.989 | 0.790 | 0.285 | 0.826 |
|  |  | Kazakh-Rural *vs.* Kazakh-Urban | 0.870 | 0.781 | 0.995 | 0.729 | 0.999 | 0.458 | 0.353 | 0.664 |
|  |  | Mongol-Rural *vs.* Mongol-Urban | 0.731 | 0.339 | 0.646 | 0.712 | 0.324 | 0.365 | 0.123 | 0.640 |
|  |  | Tibetan-Rural *vs.* Tibetan-Urban | 0.781 | 0.957 | 0.909 | 0.841 | 0.859 | 0.514 | 0.889 | 0.955 |
|  |  | Uyghur-Rural *vs.* Uyghur-Urban | 0.554 | 0.854 | 0.669 | 0.661 | 0.979 | 0.822 | 0.503 | 0.811 |
|  |  | Zhuang-Rural *vs.* Zhuang-Urban | 0.674 | 0.872 | 0.877 | 0.999 | 0.967 | 0.722 | 0.658 | 0.780 |
|  | (%) With significant differences | | 0 | 0 | 0 | 0 | 0 | 0 | 0 | 0 |
|  | Genus | Bai-Rural *vs.* Bai-Urban | 0.893 | 0.666 | 0.922 | 0.978 | 0.868 | 0.113 | 0.221 | 0.856 |
|  |  | Han-Rural *vs.* Han-Urban | 0.742 | 0.918 | 0.836 | 0.885 | 0.958 | 0.690 | 0.130 | 0.753 |
|  |  | Kazakh-Rural *vs.* Kazakh-Urban | 0.973 | 0.955 | 0.816 | 0.962 | 0.854 | 0.605 | 0.307 | 0.642 |
|  |  | Mongol-Rural *vs.* Mongol-Urban | 0.781 | 0.412 | 0.794 | 0.848 | 0.467 | 0.546 | 0.129 | 0.730 |
|  |  | Tibetan-Rural *vs.* Tibetan-Urban | 0.734 | 0.967 | 0.895 | 0.769 | 0.825 | 0.972 | 0.730 | 0.978 |
|  |  | Uyghur-Rural *vs.* Uyghur-Urban | 0.605 | 0.815 | 0.738 | 0.721 | 0.925 | 0.611 | 0.820 | 0.903 |
|  |  | Zhuang-Rural *vs.* Zhuang-Urban | 0.741 | 0.741 | 0.914 | 0.959 | 0.766 | 0.453 | 0.183 | 0.683 |
|  | (%) With significant differences | | 0 | 0 | 0 | 0 | 0 | 0 | 0 | 0 |
|  | Species | Bai-Rural *vs.* Bai-Urban | 0.512 | 0.712 | 0.655 | 0.593 | 0.769 | 0.771 | 0.896 | 0.761 |
|  |  | Han-Rural *vs.* Han-Urban | 0.575 | 0.777 | 0.606 | 0.703 | 0.720 | 0.925 | 0.315 | 0.638 |
|  |  | Kazakh-Rural *vs.* Kazakh-Urban | 0.802 | 0.953 | 0.901 | 0.877 | 0.972 | 0.332 | 0.233 | 0.662 |
|  |  | Mongol-Rural *vs.* Mongol-Urban | 0.698 | 0.335 | 0.761 | 0.850 | 0.375 | 0.450 | 0.135 | 0.606 |
|  |  | Tibetan-Rural *vs.* Tibetan-Urban | 0.848 | 0.649 | 0.786 | 0.988 | 0.577 | 0.164 | 0.759 | 0.593 |
|  |  | Uyghur-Rural *vs.* Uyghur-Urban | 0.711 | 0.752 | 0.862 | 0.936 | 0.801 | 0.579 | 0.663 | 0.895 |
|  |  | Zhuang-Rural *vs.* Zhuang-Urban | 0.618 | 0.915 | 0.814 | 0.899 | 0.875 | 0.356 | 0.086 | 0.725 |
|  | (%) With significant differences | | 0 | 0 | 0 | 0 | 0 | 0 | 0 | 0 |
| *q*  = 2 | Phylum | Bai-Rural *vs.* Bai-Urban | 0.874 | 0.886 | 0.951 | 0.946 | 0.822 | 0.389 | 0.639 | 0.955 |
|  |  | Han-Rural *vs.* Han-Urban | 0.933 | 0.592 | 0.981 | 0.987 | 0.661 | 0.425 | 0.296 | 0.972 |
|  |  | Kazakh-Rural *vs.* Kazakh-Urban | 0.786 | 0.720 | 0.924 | 0.903 | 0.591 | 0.517 | 0.257 | 0.917 |
|  |  | Mongol-Rural *vs.* Mongol-Urban | 0.964 | 0.615 | 0.874 | 0.893 | 0.680 | 0.531 | 0.212 | 0.893 |
|  |  | Tibetan-Rural *vs.* Tibetan-Urban | 0.955 | 0.676 | 0.950 | 0.785 | 0.788 | 0.264 | 0.514 | 0.707 |
|  |  | Uyghur-Rural *vs.* Uyghur-Urban | 0.749 | 0.620 | 0.650 | 0.630 | 0.618 | 0.938 | 0.073 | 0.761 |
|  |  | Zhuang-Rural *vs.* Zhuang-Urban | 0.664 | 0.654 | 0.764 | 0.782 | 0.809 | 0.886 | 0.868 | 0.810 |
|  | (%) With significant differences | | 0 | 0 | 0 | 0 | 0 | 0 | 0 | 0 |
|  | Family | Bai-Rural *vs.* Bai-Urban | 0.632 | 0.969 | 0.595 | 0.623 | 0.879 | 0.680 | 0.722 | 0.632 |
|  |  | Han-Rural *vs.* Han-Urban | 0.793 | 0.931 | 0.876 | 0.925 | 0.914 | 0.768 | 0.407 | 0.886 |
|  |  | Kazakh-Rural *vs.* Kazakh-Urban | 0.741 | 0.833 | 0.954 | 0.786 | 0.986 | 0.460 | 0.449 | 0.568 |
|  |  | Mongol-Rural *vs.* Mongol-Urban | 0.682 | 0.480 | 0.703 | 0.763 | 0.494 | 0.133 | 0.441 | 0.657 |
|  |  | Tibetan-Rural *vs.* Tibetan-Urban | 0.698 | 0.909 | 0.770 | 0.721 | 0.920 | 0.454 | 0.704 | 0.846 |
|  |  | Uyghur-Rural *vs.* Uyghur-Urban | 0.517 | 0.847 | 0.652 | 0.643 | 0.924 | 0.040 | 0.650 | 0.795 |
|  |  | Zhuang-Rural *vs.* Zhuang-Urban | 0.842 | 0.887 | 0.994 | 0.991 | 0.970 | 0.142 | 0.714 | 0.965 |
|  | (%) With significant differences | | 0 | 0 | 0 | 0 | 0 | 14.3% (1/7) | 0 | 0 |
|  | Genus | Bai-Rural *vs.* Bai-Urban | 0.696 | 0.559 | 0.986 | 0.925 | 0.754 | 0.235 | 0.201 | 0.902 |
|  |  | Han-Rural *vs.* Han-Urban | 0.735 | 0.985 | 0.898 | 0.959 | 0.990 | 0.589 | 0.243 | 0.817 |
|  |  | Kazakh-Rural *vs.* Kazakh-Urban | 0.948 | 0.991 | 0.778 | 0.920 | 0.815 | 0.420 | 0.337 | 0.652 |
|  |  | Mongol-Rural *vs.* Mongol-Urban | 0.865 | 0.594 | 0.863 | 0.861 | 0.601 | 0.566 | 0.287 | 0.784 |
|  |  | Tibetan-Rural *vs.* Tibetan-Urban | 0.623 | 0.977 | 0.791 | 0.788 | 0.840 | 0.723 | 0.551 | 0.866 |
|  |  | Uyghur-Rural *vs.* Uyghur-Urban | 0.556 | 0.832 | 0.706 | 0.718 | 0.884 | 0.522 | 0.455 | 0.751 |
|  |  | Zhuang-Rural *vs.* Zhuang-Urban | 0.927 | 0.729 | 0.911 | 0.978 | 0.885 | 0.370 | 0.444 | 0.765 |
|  | (%) With significant differences | | 0 | 0 | 0 | 0 | 0 | 0 | 0 | 0 |
|  | Species | Bai-Rural *vs.* Bai-Urban | 0.360 | 0.439 | 0.576 | 0.561 | 0.579 | 0.275 | 0.844 | 0.657 |
|  |  | Han-Rural *vs.* Han-Urban | 0.609 | 0.717 | 0.707 | 0.807 | 0.705 | 0.898 | 0.748 | 0.698 |
|  |  | Kazakh-Rural *vs.* Kazakh-Urban | 0.901 | 0.836 | 0.940 | 0.910 | 0.916 | 0.662 | 0.360 | 0.766 |
|  |  | Mongol-Rural *vs.* Mongol-Urban | 0.798 | 0.533 | 0.805 | 0.881 | 0.474 | 0.947 | 0.242 | 0.685 |
|  |  | Tibetan-Rural *vs.* Tibetan-Urban | 0.966 | 0.627 | 0.857 | 0.866 | 0.632 | 0.677 | 0.775 | 0.899 |
|  |  | Uyghur-Rural *vs.* Uyghur-Urban | 0.400 | 0.495 | 0.724 | 0.821 | 0.672 | 0.725 | 0.917 | 0.746 |
|  |  | Zhuang-Rural *vs.* Zhuang-Urban | 0.622 | 0.811 | 0.762 | 0.810 | 0.761 | 0.371 | 0.066 | 0.822 |
|  | (%) With significant differences | | 0 | 0 | 0 | 0 | 0 | 0 | 0 | 0 |
| *q*  = 3 | Phylum | Bai-Rural *vs.* Bai-Urban | 0.910 | 0.827 | 0.985 | 0.956 | 0.724 | 0.444 | 0.509 | 0.942 |
|  |  | Han-Rural *vs.* Han-Urban | 0.857 | 0.542 | 0.918 | 0.933 | 0.609 | 0.675 | 0.319 | 0.862 |
|  |  | Kazakh-Rural *vs.* Kazakh-Urban | 0.887 | 0.647 | 0.927 | 0.897 | 0.639 | 0.664 | 0.267 | 0.887 |
|  |  | Mongol-Rural *vs.* Mongol-Urban | 0.956 | 0.650 | 0.840 | 0.848 | 0.728 | 0.454 | 0.215 | 0.848 |
|  |  | Tibetan-Rural *vs.* Tibetan-Urban | 0.795 | 0.780 | 0.991 | 0.801 | 0.812 | 0.315 | 0.484 | 0.750 |
|  |  | Uyghur-Rural *vs.* Uyghur-Urban | 0.915 | 0.521 | 0.812 | 0.763 | 0.531 | 0.340 | 0.067 | 0.981 |
|  |  | Zhuang-Rural *vs.* Zhuang-Urban | 0.726 | 0.666 | 0.777 | 0.865 | 0.727 | 0.577 | 0.999 | 0.704 |
|  | (%) With significant differences | | 0 | 0 | 0 | 0 | 0 | 0 | 0 | 0 |
|  | Family | Bai-Rural *vs.* Bai-Urban | 0.493 | 0.934 | 0.541 | 0.641 | 0.850 | 0.561 | 0.415 | 0.461 |
|  |  | Han-Rural *vs.* Han-Urban | 0.828 | 0.924 | 0.927 | 0.981 | 0.889 | 0.067 | 0.638 | 0.934 |
|  |  | Kazakh-Rural *vs.* Kazakh-Urban | 0.572 | 0.940 | 0.931 | 0.806 | 0.974 | 0.294 | 0.147 | 0.609 |
|  |  | Mongol-Rural *vs.* Mongol-Urban | 0.561 | 0.493 | 0.725 | 0.810 | 0.558 | 0.943 | 0.679 | 0.625 |
|  |  | Tibetan-Rural *vs.* Tibetan-Urban | 0.659 | 0.869 | 0.734 | 0.737 | 0.923 | 0.283 | 0.660 | 0.809 |
|  |  | Uyghur-Rural *vs.* Uyghur-Urban | 0.503 | 0.800 | 0.596 | 0.603 | 0.871 | 0.483 | 0.698 | 0.750 |
|  |  | Zhuang-Rural *vs.* Zhuang-Urban | 0.892 | 0.888 | 0.937 | 0.997 | 0.913 | 0.784 | 0.997 | 0.886 |
|  | (%) With significant differences | | 0 | 0 | 0 | 0 | 0 | 0 | 0 | 0 |
|  | Genus | Bai-Rural *vs.* Bai-Urban | 0.644 | 0.524 | 0.992 | 0.973 | 0.742 | 0.485 | 0.227 | 0.887 |
|  |  | Han-Rural *vs.* Han-Urban | 0.723 | 0.933 | 0.882 | 0.969 | 0.993 | 0.392 | 0.270 | 0.809 |
|  |  | Kazakh-Rural *vs.* Kazakh-Urban | 0.949 | 0.963 | 0.721 | 0.851 | 0.772 | 0.933 | 0.469 | 0.681 |
|  |  | Mongol-Rural *vs.* Mongol-Urban | 0.954 | 0.720 | 0.829 | 0.796 | 0.677 | 0.745 | 0.410 | 0.844 |
|  |  | Tibetan-Rural *vs.* Tibetan-Urban | 0.575 | 0.990 | 0.743 | 0.795 | 0.892 | 0.705 | 0.575 | 0.778 |
|  |  | Uyghur-Rural *vs.* Uyghur-Urban | 0.504 | 0.832 | 0.757 | 0.812 | 0.924 | 0.265 | 0.527 | 0.757 |
|  |  | Zhuang-Rural *vs.* Zhuang-Urban | 1.000 | 0.719 | 0.918 | 0.946 | 0.892 | 0.451 | 0.590 | 0.839 |
|  | (%) With significant differences | | 0 | 0 | 0 | 0 | 0 | 0 | 0 | 0 |
|  | Species | Bai-Rural *vs.* Bai-Urban | 0.385 | 0.403 | 0.638 | 0.577 | 0.618 | 0.580 | 0.735 | 0.749 |
|  |  | Han-Rural *vs.* Han-Urban | 0.634 | 0.731 | 0.712 | 0.833 | 0.690 | 0.324 | 0.833 | 0.707 |
|  |  | Kazakh-Rural *vs.* Kazakh-Urban | 0.977 | 0.784 | 0.971 | 0.889 | 0.794 | 0.537 | 0.358 | 0.834 |
|  |  | Mongol-Rural *vs.* Mongol-Urban | 0.666 | 0.504 | 0.722 | 0.851 | 0.425 | 0.477 | 0.407 | 0.601 |
|  |  | Tibetan-Rural *vs.* Tibetan-Urban | 0.835 | 0.649 | 0.889 | 0.828 | 0.615 | 0.452 | 0.630 | 0.999 |
|  |  | Uyghur-Rural *vs.* Uyghur-Urban | 0.363 | 0.484 | 0.725 | 0.855 | 0.638 | 0.340 | 0.261 | 0.702 |
|  |  | Zhuang-Rural *vs.* Zhuang-Urban | 0.637 | 0.778 | 0.818 | 0.860 | 0.701 | 0.256 | 0.089 | 0.871 |
|  | (%) With significant differences | | 0 | 0 | 0 | 0 | 0 | 0 | 0 | 0 |
| **Scheme-2A** | | | | | | | | | | |
| **Diversity order** | **Taxon** | **Cohort** | **PL** | | **PLEC** | | | | | |
|  |  |  | ***z*** | **ln(*c*)** | ***z*** | ***d*** | **ln(*c*)** | ***A_max_*** | ***D_max_*** | ***LGD*** |
| *q*  = 0 | Phylum | Bai *vs.* Han | 0.306 | 0.469 | 0.641 | 0.723 | 0.830 | 0.637 | 0.975 | 0.768 |
|  |  | Bai *vs.* Kazakh | 0.747 | 0.799 | 0.977 | 0.692 | 0.969 | 0.570 | 0.554 | 0.592 |
|  |  | Bai *vs.* Mongol | 0.705 | 0.538 | 0.899 | 0.908 | 0.796 | 0.465 | 0.615 | 0.951 |
|  |  | Bai *vs.* Tibetan | 0.946 | 0.697 | 0.942 | 0.935 | 0.433 | 0.292 | 0.958 | 0.665 |
|  |  | Bai *vs.* Uyghur | 0.609 | 0.392 | 0.985 | 0.677 | 0.542 | 0.844 | 0.721 | 0.513 |
|  |  | Bai *vs.* Zhuang | 0.974 | 0.994 | 0.895 | 1.000 | 0.692 | 0.110 | 0.806 | 0.783 |
|  |  | Han *vs.* Kazakh | 0.619 | 0.661 | 0.677 | 0.547 | 0.852 | 0.416 | 0.642 | 0.667 |
|  |  | Han *vs.* Mongol | 0.546 | 0.926 | 0.761 | 0.766 | 0.952 | 0.896 | 0.563 | 0.677 |
|  |  | Han *vs.* Tibetan | 0.352 | 0.246 | 0.612 | 0.806 | 0.351 | 0.609 | 0.994 | 0.452 |
|  |  | Han *vs.* Uyghur | 0.788 | 0.799 | 0.768 | 0.520 | 0.651 | 0.479 | 0.794 | 0.534 |
|  |  | Han *vs.* Zhuang | 0.349 | 0.438 | 0.515 | 0.674 | 0.503 | 0.102 | 0.694 | 0.502 |
|  |  | Kazakh *vs.* Mongol | 0.998 | 0.694 | 0.896 | 0.653 | 0.839 | 0.279 | 0.305 | 0.509 |
|  |  | Kazakh *vs.* Tibetan | 0.771 | 0.480 | 0.961 | 0.631 | 0.422 | 0.168 | 0.419 | 0.352 |
|  |  | Kazakh *vs.* Uyghur | 0.834 | 0.458 | 0.967 | 0.936 | 0.522 | 0.592 | 0.646 | 0.861 |
|  |  | Kazakh *vs.* Zhuang | 0.729 | 0.764 | 0.946 | 0.673 | 0.636 | 0.067 | 0.271 | 0.369 |
|  |  | Mongol *vs.* Tibetan | 0.758 | 0.282 | 0.844 | 0.982 | 0.332 | 0.653 | 0.585 | 0.687 |
|  |  | Mongol *vs.* Uyghur | 0.865 | 0.726 | 0.929 | 0.564 | 0.711 | 0.339 | 0.504 | 0.438 |
|  |  | Mongol *vs.* Zhuang | 0.737 | 0.517 | 0.791 | 0.905 | 0.520 | 0.100 | 0.837 | 0.755 |
|  |  | Tibetan *vs.* Uyghur | 0.674 | 0.190 | 0.933 | 0.606 | 0.188 | 0.212 | 0.742 | 0.317 |
|  |  | Tibetan *vs.* Zhuang | 0.967 | 0.671 | 0.972 | 0.942 | 0.711 | 0.118 | 0.689 | 0.871 |
|  |  | Uyghur *vs.* Zhuang | 0.624 | 0.320 | 0.902 | 0.609 | 0.301 | 0.051 | 0.361 | 0.216 |
|  | (%) With significant differences | | 0 | 0 | 0 | 0 | 0 | 0 | 0 | 0 |
|  | Family | Bai *vs.* Han | 0.361 | 0.384 | 0.516 | 0.474 | 0.580 | 0.410 | 0.841 | 0.884 |
|  |  | Bai *vs.* Kazakh | 0.877 | 0.678 | 0.914 | 0.648 | 0.779 | 0.109 | 0.419 | 0.539 |
|  |  | Bai *vs.* Mongol | 0.606 | 0.380 | 0.937 | 0.994 | 0.684 | 0.122 | 0.646 | 0.697 |
|  |  | Bai *vs.* Tibetan | 0.650 | 0.851 | 0.729 | 0.846 | 0.963 | 0.164 | 0.428 | 0.892 |
|  |  | Bai *vs.* Uyghur | 0.513 | 0.307 | 0.772 | 0.828 | 0.382 | 0.094 | 0.509 | 0.421 |
|  |  | Bai *vs.* Zhuang | 0.556 | 0.801 | 0.695 | 0.874 | 0.929 | 0.315 | 0.418 | 0.733 |
|  |  | Han *vs.* Kazakh | 0.621 | 0.737 | 0.549 | 0.410 | 0.856 | 0.345 | 0.573 | 0.541 |
|  |  | Han *vs.* Mongol | 0.875 | 0.778 | 0.635 | 0.407 | 0.957 | 0.370 | 0.553 | 0.563 |
|  |  | Han *vs.* Tibetan | 0.696 | 0.476 | 0.764 | 0.615 | 0.643 | 0.530 | 0.477 | 0.729 |
|  |  | Han *vs.* Uyghur | 0.998 | 0.760 | 0.853 | 0.590 | 0.654 | 0.098 | 0.707 | 0.383 |
|  |  | Han *vs.* Zhuang | 0.852 | 0.506 | 0.798 | 0.556 | 0.654 | 0.969 | 0.354 | 0.558 |
|  |  | Kazakh *vs.* Mongol | 0.737 | 0.598 | 0.890 | 0.685 | 0.821 | 0.940 | 0.658 | 0.774 |
|  |  | Kazakh *vs.* Tibetan | 0.789 | 0.775 | 0.707 | 0.513 | 0.821 | 0.668 | 0.829 | 0.666 |
|  |  | Kazakh *vs.* Uyghur | 0.631 | 0.509 | 0.669 | 0.836 | 0.514 | 0.026 | 0.721 | 0.746 |
|  |  | Kazakh *vs.* Zhuang | 0.683 | 0.849 | 0.620 | 0.486 | 0.846 | 0.244 | 0.802 | 0.720 |
|  |  | Mongol *vs.* Tibetan | 0.862 | 0.448 | 0.873 | 0.856 | 0.681 | 0.717 | 0.650 | 0.821 |
|  |  | Mongol *vs.* Uyghur | 0.909 | 0.999 | 0.836 | 0.811 | 0.744 | 0.056 | 0.814 | 0.588 |
|  |  | Mongol *vs.* Zhuang | 0.984 | 0.476 | 0.811 | 0.870 | 0.687 | 0.269 | 0.575 | 0.998 |
|  |  | Tibetan *vs.* Uyghur | 0.739 | 0.310 | 0.960 | 0.723 | 0.351 | 0.043 | 0.922 | 0.455 |
|  |  | Tibetan *vs.* Zhuang | 0.841 | 0.943 | 0.946 | 0.972 | 0.958 | 0.396 | 1.000 | 0.807 |
|  |  | Uyghur *vs.* Zhuang | 0.897 | 0.357 | 0.994 | 0.718 | 0.374 | 0.062 | 0.925 | 0.569 |
|  | (%) With significant differences | | 0 | 0 | 0 | 0 | 0 | 9.5% (2/21) | 0 | 0 |
|  | Genus | Bai *vs.* Han | 0.622 | 0.573 | 0.592 | 0.432 | 0.662 | 0.245 | 0.407 | 0.656 |
|  |  | Bai *vs.* Kazakh | 0.912 | 0.889 | 0.928 | 0.770 | 0.757 | 0.319 | 0.989 | 0.804 |
|  |  | Bai *vs.* Mongol | 0.970 | 0.627 | 0.978 | 0.915 | 0.775 | 0.201 | 0.276 | 0.813 |
|  |  | Bai *vs.* Tibetan | 0.734 | 0.891 | 0.803 | 0.944 | 0.911 | 0.253 | 0.994 | 0.842 |
|  |  | Bai *vs.* Uyghur | 0.793 | 0.428 | 0.826 | 0.879 | 0.448 | 0.986 | 0.975 | 0.701 |
|  |  | Bai *vs.* Zhuang | 0.503 | 0.816 | 0.629 | 0.800 | 0.813 | 0.733 | 0.691 | 0.749 |
|  |  | Han *vs.* Kazakh | 0.610 | 0.717 | 0.648 | 0.488 | 0.892 | 0.411 | 0.765 | 0.684 |
|  |  | Han *vs.* Mongol | 0.693 | 0.911 | 0.606 | 0.532 | 0.978 | 0.536 | 0.995 | 0.780 |
|  |  | Han *vs.* Tibetan | 0.950 | 0.692 | 0.811 | 0.496 | 0.769 | 0.476 | 0.356 | 0.452 |
|  |  | Han *vs.* Uyghur | 0.964 | 0.755 | 0.888 | 0.622 | 0.664 | 0.211 | 0.791 | 0.555 |
|  |  | Han *vs.* Zhuang | 0.829 | 0.698 | 0.982 | 0.611 | 0.834 | 0.303 | 0.287 | 0.376 |
|  |  | Kazakh *vs.* Mongol | 0.882 | 0.736 | 0.975 | 0.760 | 0.944 | 0.386 | 0.428 | 0.700 |
|  |  | Kazakh *vs.* Tibetan | 0.664 | 0.982 | 0.779 | 0.682 | 0.861 | 0.487 | 0.975 | 0.883 |
|  |  | Kazakh *vs.* Uyghur | 0.659 | 0.455 | 0.756 | 0.893 | 0.543 | 0.204 | 0.873 | 0.946 |
|  |  | Kazakh *vs.* Zhuang | 0.497 | 0.966 | 0.624 | 0.627 | 0.947 | 0.442 | 0.887 | 0.962 |
|  |  | Mongol *vs.* Tibetan | 0.807 | 0.680 | 0.802 | 0.975 | 0.863 | 0.792 | 0.241 | 0.643 |
|  |  | Mongol *vs.* Uyghur | 0.811 | 0.847 | 0.829 | 0.856 | 0.711 | 0.168 | 0.436 | 0.572 |
|  |  | Mongol *vs.* Zhuang | 0.625 | 0.721 | 0.659 | 0.899 | 0.887 | 0.275 | 0.206 | 0.572 |
|  |  | Tibetan *vs.* Uyghur | 0.995 | 0.458 | 0.980 | 0.847 | 0.492 | 0.192 | 0.968 | 0.826 |
|  |  | Tibetan *vs.* Zhuang | 0.786 | 0.925 | 0.819 | 0.859 | 0.924 | 0.298 | 0.597 | 0.890 |
|  |  | Uyghur *vs.* Zhuang | 0.820 | 0.482 | 0.872 | 0.768 | 0.503 | 0.735 | 0.822 | 0.904 |
|  | (%) With significant differences | | 0 | 0 | 0 | 0 | 0 | 0 | 0 | 0 |
|  | Species | Bai *vs.* Han | 0.041 | 0.873 | 0.449 | 0.391 | 0.954 | 0.311 | 0.108 | 0.002 |
|  |  | Bai *vs.* Kazakh | 0.090 | 0.985 | 0.305 | 0.655 | 0.947 | 0.478 | 0.169 | 0.228 |
|  |  | Bai *vs.* Mongol | 0.138 | 0.786 | 0.396 | 0.882 | 0.904 | 0.537 | 0.094 | 0.035 |
|  |  | Bai *vs.* Tibetan | 0.130 | 0.879 | 0.508 | 0.920 | 0.916 | 0.327 | 0.135 | 0.103 |
|  |  | Bai *vs.* Uyghur | 0.105 | 0.695 | 0.461 | 0.863 | 0.662 | 0.799 | 0.121 | 0.200 |
|  |  | Bai *vs.* Zhuang | 0.040 | 0.745 | 0.377 | 0.676 | 0.786 | 0.533 | 0.115 | 0.020 |
|  |  | Han *vs.* Kazakh | 0.749 | 0.855 | 0.565 | 0.364 | 0.880 | 0.412 | 0.642 | 0.539 |
|  |  | Han *vs.* Mongol | 0.856 | 0.836 | 0.687 | 0.494 | 0.965 | 0.359 | 0.661 | 0.587 |
|  |  | Han *vs.* Tibetan | 0.682 | 0.744 | 0.994 | 0.446 | 0.851 | 0.530 | 0.226 | 0.257 |
|  |  | Han *vs.* Uyghur | 0.868 | 0.787 | 0.831 | 0.598 | 0.723 | 0.316 | 0.792 | 0.558 |
|  |  | Han *vs.* Zhuang | 0.924 | 0.615 | 0.888 | 0.610 | 0.748 | 0.423 | 0.344 | 0.508 |
|  |  | Kazakh *vs.* Mongol | 0.915 | 0.774 | 0.860 | 0.650 | 0.897 | 0.981 | 0.622 | 0.653 |
|  |  | Kazakh *vs.* Tibetan | 0.531 | 0.921 | 0.600 | 0.576 | 0.996 | 0.476 | 0.901 | 0.993 |
|  |  | Kazakh *vs.* Uyghur | 0.896 | 0.648 | 0.775 | 0.769 | 0.610 | 0.564 | 0.719 | 0.962 |
|  |  | Kazakh *vs.* Zhuang | 0.796 | 0.792 | 0.645 | 0.466 | 0.894 | 0.948 | 0.904 | 0.664 |
|  |  | Mongol *vs.* Tibetan | 0.653 | 0.680 | 0.722 | 0.976 | 0.847 | 0.541 | 0.243 | 0.506 |
|  |  | Mongol *vs.* Uyghur | 0.996 | 0.983 | 0.940 | 0.842 | 0.814 | 0.712 | 0.792 | 0.684 |
|  |  | Mongol *vs.* Zhuang | 0.906 | 0.566 | 0.818 | 0.860 | 0.797 | 0.953 | 0.469 | 0.934 |
|  |  | Tibetan *vs.* Uyghur | 0.606 | 0.559 | 0.826 | 0.820 | 0.588 | 0.346 | 0.792 | 0.967 |
|  |  | Tibetan *vs.* Zhuang | 0.649 | 0.859 | 0.901 | 0.771 | 0.896 | 0.564 | 0.515 | 0.524 |
|  |  | Uyghur *vs.* Zhuang | 0.926 | 0.480 | 0.880 | 0.706 | 0.527 | 0.692 | 0.988 | 0.687 |
|  | (%) With significant differences | | 9.5% (2/21) | 0 | 0 | 0 | 0 | 0 | 0 | 14.3% (3/21) |
| *q*  = 1 | Phylum | Bai *vs.* Han | 0.799 | 0.862 | 0.836 | 0.701 | 0.805 | 0.451 | 0.336 | 0.972 |
|  |  | Bai *vs.* Kazakh | 0.742 | 0.608 | 0.713 | 0.674 | 0.724 | 0.580 | 0.108 | 0.734 |
|  |  | Bai *vs.* Mongol | 0.780 | 0.541 | 0.787 | 0.762 | 0.606 | 0.874 | 0.476 | 0.913 |
|  |  | Bai *vs.* Tibetan | 0.885 | 0.757 | 0.875 | 0.967 | 0.832 | 0.383 | 0.297 | 0.769 |
|  |  | Bai *vs.* Uyghur | 0.966 | 0.454 | 0.998 | 0.975 | 0.479 | 0.635 | 0.093 | 0.979 |
|  |  | Bai *vs.* Zhuang | 0.997 | 0.985 | 0.991 | 0.998 | 0.993 | 0.061 | 0.958 | 0.988 |
|  |  | Han *vs.* Kazakh | 0.585 | 0.477 | 0.517 | 0.533 | 0.529 | 0.339 | 0.012 | 0.697 |
|  |  | Han *vs.* Mongol | 0.992 | 0.410 | 0.947 | 0.992 | 0.452 | 0.447 | 0.064 | 0.972 |
|  |  | Han *vs.* Tibetan | 0.666 | 0.623 | 0.699 | 0.706 | 0.633 | 0.834 | 0.035 | 0.729 |
|  |  | Han *vs.* Uyghur | 0.824 | 0.312 | 0.912 | 0.870 | 0.346 | 0.333 | 0.010 | 0.952 |
|  |  | Han *vs.* Zhuang | 0.792 | 0.857 | 0.837 | 0.718 | 0.812 | 0.120 | 0.370 | 0.946 |
|  |  | Kazakh *vs.* Mongol | 0.504 | 0.931 | 0.478 | 0.451 | 0.995 | 0.468 | 0.138 | 0.619 |
|  |  | Kazakh *vs.* Tibetan | 0.822 | 0.800 | 0.750 | 0.652 | 0.837 | 0.215 | 0.501 | 0.939 |
|  |  | Kazakh *vs.* Uyghur | 0.779 | 0.841 | 0.681 | 0.728 | 0.763 | 0.830 | 0.885 | 0.777 |
|  |  | Kazakh *vs.* Zhuang | 0.740 | 0.561 | 0.716 | 0.687 | 0.685 | 0.038 | 0.169 | 0.717 |
|  |  | Mongol *vs.* Tibetan | 0.632 | 0.834 | 0.665 | 0.745 | 0.822 | 0.437 | 0.539 | 0.694 |
|  |  | Mongol *vs.* Uyghur | 0.782 | 0.686 | 0.825 | 0.808 | 0.691 | 0.514 | 0.158 | 0.896 |
|  |  | Mongol *vs.* Zhuang | 0.781 | 0.538 | 0.793 | 0.770 | 0.608 | 0.066 | 0.600 | 0.926 |
|  |  | Tibetan *vs.* Uyghur | 0.926 | 0.621 | 0.887 | 0.983 | 0.601 | 0.236 | 0.541 | 0.792 |
|  |  | Tibetan *vs.* Zhuang | 0.869 | 0.733 | 0.858 | 0.967 | 0.803 | 0.069 | 0.376 | 0.763 |
|  |  | Uyghur *vs.* Zhuang | 0.967 | 0.392 | 0.992 | 0.974 | 0.443 | 0.034 | 0.199 | 0.974 |
|  | (%) With significant differences | | 0 | 0 | 0 | 0 | 0 | 9.5% (2/21) | 14.3% (3/21) | 0 |
|  | Family | Bai *vs.* Han | 0.579 | 0.808 | 0.552 | 0.454 | 0.804 | 0.856 | 0.720 | 0.738 |
|  |  | Bai *vs.* Kazakh | 0.573 | 0.873 | 0.431 | 0.354 | 0.973 | 0.169 | 0.174 | 0.773 |
|  |  | Bai *vs.* Mongol | 0.689 | 0.468 | 0.753 | 0.788 | 0.581 | 0.214 | 0.394 | 0.686 |
|  |  | Bai *vs.* Tibetan | 0.758 | 0.900 | 0.801 | 0.842 | 0.886 | 0.316 | 0.736 | 0.901 |
|  |  | Bai *vs.* Uyghur | 0.928 | 0.345 | 0.883 | 0.653 | 0.379 | 0.149 | 0.066 | 0.893 |
|  |  | Bai *vs.* Zhuang | 0.945 | 0.868 | 0.830 | 0.877 | 0.978 | 0.466 | 0.517 | 0.934 |
|  |  | Han *vs.* Kazakh | 0.368 | 0.985 | 0.273 | 0.239 | 0.872 | 0.395 | 0.052 | 0.590 |
|  |  | Han *vs.* Mongol | 0.962 | 0.553 | 0.930 | 0.681 | 0.636 | 0.454 | 0.154 | 0.839 |
|  |  | Han *vs.* Tibetan | 0.393 | 0.654 | 0.408 | 0.349 | 0.679 | 0.669 | 0.418 | 0.621 |
|  |  | Han *vs.* Uyghur | 0.636 | 0.466 | 0.608 | 0.496 | 0.490 | 0.337 | 0.026 | 0.891 |
|  |  | Han *vs.* Zhuang | 0.508 | 0.922 | 0.422 | 0.315 | 0.811 | 0.501 | 0.222 | 0.665 |
|  |  | Kazakh *vs.* Mongol | 0.382 | 0.639 | 0.342 | 0.327 | 0.589 | 0.694 | 0.453 | 0.526 |
|  |  | Kazakh *vs.* Tibetan | 0.747 | 0.780 | 0.636 | 0.510 | 0.909 | 0.360 | 0.340 | 0.865 |
|  |  | Kazakh *vs.* Uyghur | 0.590 | 0.427 | 0.516 | 0.625 | 0.384 | 0.651 | 0.678 | 0.631 |
|  |  | Kazakh *vs.* Zhuang | 0.575 | 0.979 | 0.491 | 0.369 | 0.990 | 0.132 | 0.402 | 0.804 |
|  |  | Mongol *vs.* Tibetan | 0.554 | 0.433 | 0.598 | 0.642 | 0.539 | 0.569 | 0.688 | 0.612 |
|  |  | Mongol *vs.* Uyghur | 0.643 | 0.907 | 0.683 | 0.601 | 0.853 | 0.555 | 0.264 | 0.738 |
|  |  | Mongol *vs.* Zhuang | 0.613 | 0.538 | 0.605 | 0.668 | 0.560 | 0.171 | 0.902 | 0.596 |
|  |  | Tibetan *vs.* Uyghur | 0.912 | 0.371 | 0.922 | 0.819 | 0.414 | 0.306 | 0.201 | 0.817 |
|  |  | Tibetan *vs.* Zhuang | 0.823 | 0.753 | 0.905 | 0.907 | 0.867 | 0.227 | 0.780 | 0.961 |
|  |  | Uyghur *vs.* Zhuang | 0.966 | 0.394 | 0.988 | 0.697 | 0.336 | 0.113 | 0.216 | 0.830 |
|  | (%) With significant differences | | 0 | 0 | 0 | 0 | 0 | 0 | 4.8% (1/21) | 0 |
|  | Genus | Bai *vs.* Han | 0.514 | 0.642 | 0.604 | 0.473 | 0.749 | 0.844 | 0.924 | 0.742 |
|  |  | Bai *vs.* Kazakh | 0.539 | 0.903 | 0.553 | 0.452 | 0.895 | 0.522 | 0.185 | 0.734 |
|  |  | Bai *vs.* Mongol | 0.684 | 0.612 | 0.701 | 0.769 | 0.611 | 0.977 | 0.782 | 0.680 |
|  |  | Bai *vs.* Tibetan | 0.938 | 0.691 | 0.976 | 0.967 | 0.757 | 0.667 | 0.415 | 0.985 |
|  |  | Bai *vs.* Uyghur | 0.985 | 0.482 | 0.904 | 0.604 | 0.492 | 0.437 | 0.201 | 0.862 |
|  |  | Bai *vs.* Zhuang | 0.854 | 0.837 | 0.924 | 0.915 | 0.889 | 0.298 | 0.893 | 0.928 |
|  |  | Han *vs.* Kazakh | 0.306 | 0.789 | 0.347 | 0.319 | 0.885 | 0.641 | 0.123 | 0.525 |
|  |  | Han *vs.* Mongol | 0.899 | 0.880 | 0.991 | 0.701 | 0.781 | 0.749 | 0.736 | 0.864 |
|  |  | Han *vs.* Tibetan | 0.520 | 0.355 | 0.669 | 0.505 | 0.560 | 0.642 | 0.121 | 0.803 |
|  |  | Han *vs.* Uyghur | 0.652 | 0.729 | 0.603 | 0.441 | 0.627 | 0.586 | 0.136 | 0.919 |
|  |  | Han *vs.* Zhuang | 0.630 | 0.784 | 0.676 | 0.470 | 0.874 | 0.515 | 0.914 | 0.795 |
|  |  | Kazakh *vs.* Mongol | 0.373 | 0.732 | 0.421 | 0.386 | 0.710 | 0.536 | 0.334 | 0.573 |
|  |  | Kazakh *vs.* Tibetan | 0.720 | 0.635 | 0.696 | 0.609 | 0.720 | 0.749 | 0.095 | 0.788 |
|  |  | Kazakh *vs.* Uyghur | 0.532 | 0.521 | 0.632 | 0.828 | 0.518 | 0.746 | 0.918 | 0.560 |
|  |  | Kazakh *vs.* Zhuang | 0.430 | 0.971 | 0.492 | 0.437 | 0.983 | 0.186 | 0.206 | 0.656 |
|  |  | Mongol *vs.* Tibetan | 0.676 | 0.435 | 0.726 | 0.761 | 0.523 | 0.664 | 0.222 | 0.737 |
|  |  | Mongol *vs.* Uyghur | 0.718 | 0.847 | 0.679 | 0.531 | 0.882 | 0.472 | 0.365 | 0.782 |
|  |  | Mongol *vs.* Zhuang | 0.793 | 0.730 | 0.756 | 0.797 | 0.693 | 0.272 | 0.857 | 0.746 |
|  |  | Tibetan *vs.* Uyghur | 0.968 | 0.419 | 0.967 | 0.761 | 0.469 | 0.624 | 0.118 | 0.920 |
|  |  | Tibetan *vs.* Zhuang | 0.814 | 0.542 | 0.919 | 0.918 | 0.654 | 0.263 | 0.275 | 0.954 |
|  |  | Uyghur *vs.* Zhuang | 0.845 | 0.527 | 0.850 | 0.585 | 0.507 | 0.164 | 0.271 | 0.926 |
|  | (%) With significant differences | | 0 | 0 | 0 | 0 | 0 | 0 | 0 | 0 |
|  | Species | Bai *vs.* Han | 0.992 | 0.598 | 0.932 | 0.601 | 0.750 | 0.621 | 0.029 | 0.858 |
|  |  | Bai *vs.* Kazakh | 0.286 | 0.856 | 0.338 | 0.311 | 0.872 | 0.405 | 0.108 | 0.561 |
|  |  | Bai *vs.* Mongol | 0.803 | 0.525 | 0.914 | 0.951 | 0.619 | 0.507 | 0.017 | 0.854 |
|  |  | Bai *vs.* Tibetan | 0.566 | 0.870 | 0.691 | 0.861 | 0.842 | 0.730 | 0.289 | 0.675 |
|  |  | Bai *vs.* Uyghur | 0.580 | 0.579 | 0.613 | 0.549 | 0.666 | 0.429 | 0.044 | 0.698 |
|  |  | Bai *vs.* Zhuang | 0.507 | 0.798 | 0.677 | 0.906 | 0.898 | 0.852 | 0.033 | 0.488 |
|  |  | Han *vs.* Kazakh | 0.308 | 0.552 | 0.295 | 0.272 | 0.650 | 0.384 | 0.749 | 0.657 |
|  |  | Han *vs.* Mongol | 0.772 | 0.865 | 0.837 | 0.669 | 0.844 | 0.413 | 0.273 | 0.981 |
|  |  | Han *vs.* Tibetan | 0.523 | 0.520 | 0.589 | 0.493 | 0.623 | 0.487 | 0.356 | 0.728 |
|  |  | Han *vs.* Uyghur | 0.564 | 0.904 | 0.552 | 0.470 | 0.901 | 0.451 | 0.315 | 0.785 |
|  |  | Han *vs.* Zhuang | 0.543 | 0.707 | 0.610 | 0.502 | 0.782 | 0.575 | 0.976 | 0.680 |
|  |  | Kazakh *vs.* Mongol | 0.427 | 0.485 | 0.444 | 0.353 | 0.540 | 0.742 | 0.697 | 0.718 |
|  |  | Kazakh *vs.* Tibetan | 0.611 | 0.986 | 0.610 | 0.476 | 0.983 | 0.542 | 0.485 | 0.891 |
|  |  | Kazakh *vs.* Uyghur | 0.653 | 0.499 | 0.712 | 0.752 | 0.592 | 0.647 | 0.472 | 0.858 |
|  |  | Kazakh *vs.* Zhuang | 0.401 | 0.662 | 0.396 | 0.271 | 0.752 | 0.465 | 0.723 | 0.856 |
|  |  | Mongol *vs.* Tibetan | 0.802 | 0.496 | 0.799 | 0.799 | 0.568 | 0.703 | 0.169 | 0.830 |
|  |  | Mongol *vs.* Uyghur | 0.736 | 0.943 | 0.712 | 0.567 | 0.935 | 0.881 | 0.874 | 0.782 |
|  |  | Mongol *vs.* Zhuang | 0.811 | 0.573 | 0.826 | 0.840 | 0.617 | 0.607 | 0.306 | 0.704 |
|  |  | Tibetan *vs.* Uyghur | 0.928 | 0.535 | 0.860 | 0.711 | 0.613 | 0.643 | 0.191 | 0.996 |
|  |  | Tibetan *vs.* Zhuang | 0.902 | 0.692 | 0.930 | 0.897 | 0.770 | 0.807 | 0.394 | 0.973 |
|  |  | Uyghur *vs.* Zhuang | 0.852 | 0.639 | 0.754 | 0.554 | 0.664 | 0.536 | 0.243 | 0.963 |
|  | (%) With significant differences | | 0 | 0 | 0 | 0 | 0 | 0 | 19.0% (4/21) | 0 |
| *q* = 2 | Phylum | Bai *vs.* Han | 0.893 | 0.703 | 0.888 | 0.858 | 0.721 | 0.232 | 0.193 | 0.933 |
|  |  | Bai *vs.* Kazakh | 0.885 | 0.665 | 0.812 | 0.822 | 0.784 | 0.504 | 0.217 | 0.842 |
|  |  | Bai *vs.* Mongol | 0.901 | 0.641 | 0.866 | 0.802 | 0.721 | 0.391 | 0.568 | 0.969 |
|  |  | Bai *vs.* Tibetan | 0.826 | 0.629 | 0.902 | 0.959 | 0.678 | 0.702 | 0.117 | 0.803 |
|  |  | Bai *vs.* Uyghur | 0.956 | 0.541 | 0.898 | 0.911 | 0.546 | 0.412 | 0.172 | 0.864 |
|  |  | Bai *vs.* Zhuang | 0.978 | 0.883 | 0.980 | 0.979 | 0.907 | 0.675 | 0.793 | 0.988 |
|  |  | Han *vs.* Kazakh | 0.768 | 0.389 | 0.686 | 0.706 | 0.504 | 0.176 | 0.023 | 0.729 |
|  |  | Han *vs.* Mongol | 0.999 | 0.364 | 0.956 | 0.907 | 0.479 | 0.358 | 0.046 | 0.904 |
|  |  | Han *vs.* Tibetan | 0.719 | 0.400 | 0.769 | 0.783 | 0.469 | 0.229 | 0.006 | 0.743 |
|  |  | Han *vs.* Uyghur | 0.977 | 0.291 | 0.960 | 0.960 | 0.333 | 0.172 | 0.028 | 0.883 |
|  |  | Han *vs.* Zhuang | 0.913 | 0.808 | 0.900 | 0.856 | 0.848 | 0.213 | 0.384 | 0.918 |
|  |  | Kazakh *vs.* Mongol | 0.756 | 0.845 | 0.662 | 0.647 | 0.959 | 0.235 | 0.285 | 0.856 |
|  |  | Kazakh *vs.* Tibetan | 0.983 | 0.979 | 0.890 | 0.833 | 0.913 | 0.749 | 0.889 | 0.996 |
|  |  | Kazakh *vs.* Uyghur | 0.869 | 0.912 | 0.743 | 0.771 | 0.805 | 0.815 | 0.950 | 0.777 |
|  |  | Kazakh *vs.* Zhuang | 0.857 | 0.543 | 0.805 | 0.814 | 0.662 | 0.780 | 0.190 | 0.848 |
|  |  | Mongol *vs.* Tibetan | 0.701 | 0.885 | 0.751 | 0.756 | 0.860 | 0.305 | 0.213 | 0.812 |
|  |  | Mongol *vs.* Uyghur | 0.978 | 0.686 | 0.983 | 0.979 | 0.654 | 0.170 | 0.296 | 0.809 |
|  |  | Mongol *vs.* Zhuang | 0.921 | 0.566 | 0.889 | 0.829 | 0.636 | 0.279 | 0.431 | 0.988 |
|  |  | Tibetan *vs.* Uyghur | 0.828 | 0.866 | 0.816 | 0.884 | 0.856 | 0.658 | 0.856 | 0.722 |
|  |  | Tibetan *vs.* Zhuang | 0.822 | 0.581 | 0.860 | 0.935 | 0.591 | 0.973 | 0.143 | 0.815 |
|  |  | Uyghur *vs.* Zhuang | 0.966 | 0.430 | 0.925 | 0.928 | 0.446 | 0.657 | 0.194 | 0.845 |
|  | (%) With significant differences | | 0 | 0 | 0 | 0 | 0 | 0 | 19.0% (4/21) | 0 |
|  | Family | Bai *vs.* Han | 0.571 | 0.823 | 0.520 | 0.440 | 0.837 | 0.486 | 0.547 | 0.733 |
|  |  | Bai *vs.* Kazakh | 0.482 | 0.865 | 0.574 | 0.542 | 0.952 | 0.904 | 0.496 | 0.812 |
|  |  | Bai *vs.* Mongol | 0.639 | 0.562 | 0.704 | 0.742 | 0.622 | 0.978 | 0.814 | 0.658 |
|  |  | Bai *vs.* Tibetan | 0.857 | 0.852 | 0.904 | 0.894 | 0.898 | 0.960 | 0.979 | 0.983 |
|  |  | Bai *vs.* Uyghur | 0.861 | 0.444 | 0.834 | 0.659 | 0.515 | 0.634 | 0.129 | 0.967 |
|  |  | Bai *vs.* Zhuang | 0.989 | 0.900 | 0.944 | 0.989 | 0.988 | 0.185 | 0.620 | 0.910 |
|  |  | Han *vs.* Kazakh | 0.249 | 0.700 | 0.305 | 0.337 | 0.808 | 0.546 | 0.159 | 0.636 |
|  |  | Han *vs.* Mongol | 0.939 | 0.666 | 0.930 | 0.718 | 0.711 | 0.474 | 0.406 | 0.856 |
|  |  | Han *vs.* Tibetan | 0.396 | 0.603 | 0.451 | 0.368 | 0.689 | 0.500 | 0.514 | 0.706 |
|  |  | Han *vs.* Uyghur | 0.515 | 0.552 | 0.509 | 0.481 | 0.649 | 0.377 | 0.047 | 0.715 |
|  |  | Han *vs.* Zhuang | 0.540 | 0.915 | 0.460 | 0.396 | 0.790 | 0.590 | 0.216 | 0.624 |
|  |  | Kazakh *vs.* Mongol | 0.276 | 0.523 | 0.409 | 0.444 | 0.621 | 0.950 | 0.553 | 0.547 |
|  |  | Kazakh *vs.* Tibetan | 0.584 | 0.998 | 0.696 | 0.624 | 0.966 | 0.957 | 0.438 | 0.832 |
|  |  | Kazakh *vs.* Uyghur | 0.545 | 0.379 | 0.731 | 0.822 | 0.493 | 0.357 | 0.565 | 0.842 |
|  |  | Kazakh *vs.* Zhuang | 0.426 | 0.773 | 0.575 | 0.493 | 0.959 | 0.193 | 0.639 | 0.856 |
|  |  | Mongol *vs.* Tibetan | 0.518 | 0.458 | 0.623 | 0.647 | 0.590 | 0.999 | 0.740 | 0.665 |
|  |  | Mongol *vs.* Uyghur | 0.530 | 0.922 | 0.585 | 0.562 | 0.913 | 0.621 | 0.212 | 0.616 |
|  |  | Mongol *vs.* Zhuang | 0.598 | 0.613 | 0.619 | 0.731 | 0.609 | 0.202 | 0.850 | 0.595 |
|  |  | Tibetan *vs.* Uyghur | 0.983 | 0.425 | 0.925 | 0.764 | 0.513 | 0.641 | 0.164 | 0.971 |
|  |  | Tibetan *vs.* Zhuang | 0.856 | 0.765 | 0.938 | 0.865 | 0.910 | 0.188 | 0.594 | 0.923 |
|  |  | Uyghur *vs.* Zhuang | 0.862 | 0.483 | 0.849 | 0.625 | 0.446 | 0.140 | 0.205 | 0.951 |
|  | (%) With significant differences | | 0 | 0 | 0 | 0 | 0 | 0 | 4.8% (1/21) | 0 |
|  | Genus | Bai *vs.* Han | 0.499 | 0.720 | 0.549 | 0.446 | 0.765 | 0.868 | 0.570 | 0.664 |
|  |  | Bai *vs.* Kazakh | 0.487 | 0.907 | 0.477 | 0.374 | 0.939 | 0.627 | 0.076 | 0.715 |
|  |  | Bai *vs.* Mongol | 0.578 | 0.654 | 0.624 | 0.741 | 0.605 | 0.572 | 0.852 | 0.603 |
|  |  | Bai *vs.* Tibetan | 0.992 | 0.539 | 0.881 | 0.978 | 0.775 | 0.036 | 0.170 | 0.741 |
|  |  | Bai *vs.* Uyghur | 0.953 | 0.521 | 0.798 | 0.487 | 0.549 | 0.443 | 0.197 | 0.873 |
|  |  | Bai *vs.* Zhuang | 0.805 | 0.911 | 0.867 | 0.950 | 0.854 | 0.716 | 0.849 | 0.787 |
|  |  | Han *vs.* Kazakh | 0.268 | 0.855 | 0.279 | 0.269 | 0.867 | 0.777 | 0.028 | 0.438 |
|  |  | Han *vs.* Mongol | 0.958 | 0.850 | 0.989 | 0.666 | 0.779 | 0.499 | 0.768 | 0.846 |
|  |  | Han *vs.* Tibetan | 0.579 | 0.276 | 0.825 | 0.563 | 0.546 | 0.049 | 0.020 | 0.929 |
|  |  | Han *vs.* Uyghur | 0.579 | 0.754 | 0.470 | 0.331 | 0.709 | 0.613 | 0.076 | 0.838 |
|  |  | Han *vs.* Zhuang | 0.687 | 0.808 | 0.661 | 0.455 | 0.933 | 0.656 | 0.847 | 0.902 |
|  |  | Kazakh *vs.* Mongol | 0.271 | 0.760 | 0.315 | 0.316 | 0.684 | 0.990 | 0.075 | 0.425 |
|  |  | Kazakh *vs.* Tibetan | 0.680 | 0.543 | 0.568 | 0.579 | 0.743 | 0.021 | 0.046 | 0.584 |
|  |  | Kazakh *vs.* Uyghur | 0.523 | 0.613 | 0.648 | 0.879 | 0.581 | 0.523 | 0.347 | 0.562 |
|  |  | Kazakh *vs.* Zhuang | 0.386 | 0.981 | 0.414 | 0.422 | 0.914 | 0.836 | 0.135 | 0.529 |
|  |  | Mongol *vs.* Tibetan | 0.701 | 0.324 | 0.846 | 0.839 | 0.518 | 0.027 | 0.102 | 0.961 |
|  |  | Mongol *vs.* Uyghur | 0.625 | 0.891 | 0.540 | 0.419 | 0.933 | 0.811 | 0.203 | 0.710 |
|  |  | Mongol *vs.* Zhuang | 0.757 | 0.724 | 0.742 | 0.777 | 0.757 | 0.757 | 0.986 | 0.789 |
|  |  | Tibetan *vs.* Uyghur | 0.963 | 0.377 | 0.790 | 0.672 | 0.535 | 0.012 | 0.090 | 0.839 |
|  |  | Tibetan *vs.* Zhuang | 0.873 | 0.437 | 0.946 | 0.997 | 0.645 | 0.030 | 0.169 | 0.851 |
|  |  | Uyghur *vs.* Zhuang | 0.800 | 0.582 | 0.718 | 0.525 | 0.619 | 0.585 | 0.282 | 0.909 |
|  | (%) With significant differences | | 0 | 0 | 0 | 0 | 0 | 23.8% (5/21) | 14.3% (3/21) | 0 |
|  | Species | Bai *vs.* Han | 0.929 | 0.824 | 0.970 | 0.695 | 0.885 | 0.237 | 0.164 | 0.879 |
|  |  | Bai *vs.* Kazakh | 0.234 | 0.818 | 0.315 | 0.330 | 0.820 | 0.421 | 0.102 | 0.541 |
|  |  | Bai *vs.* Mongol | 0.816 | 0.603 | 0.942 | 0.918 | 0.697 | 0.777 | 0.039 | 0.822 |
|  |  | Bai *vs.* Tibetan | 0.683 | 0.721 | 0.864 | 0.947 | 0.853 | 0.582 | 0.877 | 0.897 |
|  |  | Bai *vs.* Uyghur | 0.473 | 0.735 | 0.547 | 0.523 | 0.819 | 0.474 | 0.034 | 0.587 |
|  |  | Bai *vs.* Zhuang | 0.589 | 0.847 | 0.622 | 0.831 | 0.939 | 0.968 | 0.046 | 0.491 |
|  |  | Han *vs.* Kazakh | 0.326 | 0.700 | 0.343 | 0.347 | 0.746 | 0.151 | 0.399 | 0.650 |
|  |  | Han *vs.* Mongol | 0.886 | 0.784 | 0.898 | 0.731 | 0.811 | 0.219 | 0.347 | 0.961 |
|  |  | Han *vs.* Tibetan | 0.716 | 0.598 | 0.828 | 0.648 | 0.756 | 0.167 | 0.216 | 0.999 |
|  |  | Han *vs.* Uyghur | 0.523 | 0.900 | 0.534 | 0.521 | 0.943 | 0.180 | 0.284 | 0.735 |
|  |  | Han *vs.* Zhuang | 0.713 | 0.930 | 0.602 | 0.495 | 0.816 | 0.253 | 0.677 | 0.736 |
|  |  | Kazakh *vs.* Mongol | 0.351 | 0.497 | 0.379 | 0.367 | 0.550 | 0.529 | 0.841 | 0.643 |
|  |  | Kazakh *vs.* Tibetan | 0.536 | 0.910 | 0.534 | 0.525 | 0.996 | 0.671 | 0.263 | 0.688 |
|  |  | Kazakh *vs.* Uyghur | 0.706 | 0.628 | 0.762 | 0.809 | 0.708 | 0.676 | 0.844 | 0.886 |
|  |  | Kazakh *vs.* Zhuang | 0.346 | 0.670 | 0.446 | 0.383 | 0.847 | 0.458 | 0.484 | 0.836 |
|  |  | Mongol *vs.* Tibetan | 0.853 | 0.484 | 0.921 | 0.889 | 0.610 | 0.739 | 0.153 | 0.972 |
|  |  | Mongol *vs.* Uyghur | 0.593 | 0.870 | 0.606 | 0.515 | 0.845 | 0.652 | 0.683 | 0.732 |
|  |  | Mongol *vs.* Zhuang | 0.834 | 0.669 | 0.721 | 0.733 | 0.606 | 0.783 | 0.583 | 0.734 |
|  |  | Tibetan *vs.* Uyghur | 0.788 | 0.571 | 0.706 | 0.685 | 0.734 | 0.836 | 0.182 | 0.735 |
|  |  | Tibetan *vs.* Zhuang | 0.965 | 0.610 | 0.828 | 0.901 | 0.875 | 0.596 | 0.260 | 0.736 |
|  |  | Uyghur *vs.* Zhuang | 0.650 | 0.836 | 0.758 | 0.586 | 0.783 | 0.495 | 0.360 | 0.959 |
|  | (%) With significant differences | | 0 | 0 | 0 | 0 | 0 | 0 | 14.3% (3/21) | 0 |
| *q*  = 3 | Phylum | Bai *vs.* Han | 0.963 | 0.640 | 0.940 | 0.933 | 0.681 | 0.307 | 0.163 | 0.988 |
|  |  | Bai *vs.* Kazakh | 0.946 | 0.677 | 0.880 | 0.877 | 0.771 | 0.704 | 0.269 | 0.964 |
|  |  | Bai *vs.* Mongol | 0.952 | 0.703 | 0.911 | 0.862 | 0.764 | 0.562 | 0.584 | 0.989 |
|  |  | Bai *vs.* Tibetan | 0.810 | 0.607 | 0.897 | 0.948 | 0.664 | 0.738 | 0.104 | 0.810 |
|  |  | Bai *vs.* Uyghur | 0.904 | 0.570 | 0.842 | 0.849 | 0.565 | 0.615 | 0.209 | 0.811 |
|  |  | Bai *vs.* Zhuang | 0.982 | 0.868 | 1.000 | 0.997 | 0.883 | 0.491 | 0.739 | 0.998 |
|  |  | Han *vs.* Kazakh | 0.899 | 0.360 | 0.804 | 0.794 | 0.461 | 0.253 | 0.028 | 0.930 |
|  |  | Han *vs.* Mongol | 0.985 | 0.343 | 0.936 | 0.883 | 0.475 | 0.420 | 0.035 | 0.980 |
|  |  | Han *vs.* Tibetan | 0.756 | 0.347 | 0.798 | 0.854 | 0.433 | 0.381 | 0.001 | 0.789 |
|  |  | Han *vs.* Uyghur | 0.913 | 0.271 | 0.848 | 0.839 | 0.301 | 0.228 | 0.036 | 0.740 |
|  |  | Han *vs.* Zhuang | 0.984 | 0.755 | 0.939 | 0.905 | 0.835 | 0.450 | 0.408 | 0.984 |
|  |  | Kazakh *vs.* Mongol | 0.879 | 0.817 | 0.767 | 0.736 | 0.906 | 0.367 | 0.354 | 0.981 |
|  |  | Kazakh *vs.* Tibetan | 0.884 | 0.935 | 0.976 | 0.897 | 0.867 | 0.446 | 0.645 | 0.836 |
|  |  | Kazakh *vs.* Uyghur | 0.881 | 0.918 | 0.773 | 0.780 | 0.806 | 0.828 | 1.000 | 0.816 |
|  |  | Kazakh *vs.* Zhuang | 0.918 | 0.544 | 0.881 | 0.875 | 0.615 | 0.296 | 0.191 | 0.976 |
|  |  | Mongol *vs.* Tibetan | 0.738 | 0.785 | 0.788 | 0.817 | 0.793 | 0.768 | 0.157 | 0.811 |
|  |  | Mongol *vs.* Uyghur | 0.928 | 0.675 | 0.860 | 0.853 | 0.620 | 0.296 | 0.326 | 0.743 |
|  |  | Mongol *vs.* Zhuang | 0.970 | 0.614 | 0.894 | 0.859 | 0.645 | 0.911 | 0.393 | 0.984 |
|  |  | Tibetan *vs.* Uyghur | 0.781 | 0.945 | 0.765 | 0.828 | 0.919 | 0.414 | 0.637 | 0.673 |
|  |  | Tibetan *vs.* Zhuang | 0.814 | 0.561 | 0.880 | 0.955 | 0.553 | 0.691 | 0.078 | 0.804 |
|  |  | Uyghur *vs.* Zhuang | 0.896 | 0.445 | 0.842 | 0.864 | 0.420 | 0.263 | 0.195 | 0.765 |
|  | (%) With significant differences | | 0 | 0 | 0 | 0 | 0 | 0 | 19.0% (4/21) | 0 |
|  | Family | Bai *vs.* Han | 0.612 | 0.804 | 0.597 | 0.494 | 0.882 | 0.440 | 0.658 | 0.799 |
|  |  | Bai *vs.* Kazakh | 0.452 | 0.717 | 0.612 | 0.612 | 0.915 | 0.291 | 0.682 | 0.837 |
|  |  | Bai *vs.* Mongol | 0.666 | 0.621 | 0.737 | 0.776 | 0.702 | 0.547 | 0.997 | 0.695 |
|  |  | Bai *vs.* Tibetan | 0.878 | 0.829 | 0.912 | 0.937 | 0.877 | 0.391 | 0.844 | 0.971 |
|  |  | Bai *vs.* Uyghur | 0.813 | 0.500 | 0.757 | 0.653 | 0.611 | 0.312 | 0.219 | 0.851 |
|  |  | Bai *vs.* Zhuang | 0.934 | 0.935 | 0.889 | 0.988 | 0.928 | 0.312 | 0.658 | 0.812 |
|  |  | Han *vs.* Kazakh | 0.228 | 0.537 | 0.352 | 0.366 | 0.784 | 0.218 | 0.310 | 0.690 |
|  |  | Han *vs.* Mongol | 0.933 | 0.755 | 0.953 | 0.758 | 0.749 | 0.285 | 0.673 | 0.811 |
|  |  | Han *vs.* Tibetan | 0.444 | 0.579 | 0.512 | 0.431 | 0.703 | 0.890 | 0.753 | 0.762 |
|  |  | Han *vs.* Uyghur | 0.488 | 0.611 | 0.482 | 0.487 | 0.731 | 0.228 | 0.071 | 0.696 |
|  |  | Han *vs.* Zhuang | 0.525 | 0.874 | 0.462 | 0.430 | 0.759 | 0.998 | 0.290 | 0.617 |
|  |  | Kazakh *vs.* Mongol | 0.261 | 0.465 | 0.446 | 0.500 | 0.651 | 0.610 | 0.601 | 0.589 |
|  |  | Kazakh *vs.* Tibetan | 0.514 | 0.862 | 0.692 | 0.629 | 0.973 | 0.193 | 0.510 | 0.885 |
|  |  | Kazakh *vs.* Uyghur | 0.558 | 0.341 | 0.836 | 0.891 | 0.554 | 0.920 | 0.569 | 0.954 |
|  |  | Kazakh *vs.* Zhuang | 0.411 | 0.653 | 0.639 | 0.555 | 0.970 | 0.189 | 0.784 | 0.974 |
|  |  | Mongol *vs.* Tibetan | 0.562 | 0.484 | 0.658 | 0.704 | 0.616 | 0.283 | 0.812 | 0.668 |
|  |  | Mongol *vs.* Uyghur | 0.498 | 0.899 | 0.565 | 0.563 | 0.948 | 0.664 | 0.226 | 0.541 |
|  |  | Mongol *vs.* Zhuang | 0.594 | 0.669 | 0.595 | 0.745 | 0.652 | 0.223 | 0.714 | 0.565 |
|  |  | Tibetan *vs.* Uyghur | 0.926 | 0.437 | 0.840 | 0.724 | 0.552 | 0.191 | 0.174 | 0.880 |
|  |  | Tibetan *vs.* Zhuang | 0.946 | 0.766 | 0.988 | 0.935 | 0.926 | 0.680 | 0.452 | 0.837 |
|  |  | Uyghur *vs.* Zhuang | 0.835 | 0.507 | 0.777 | 0.592 | 0.495 | 0.170 | 0.226 | 0.965 |
|  | (%) With significant differences | | 0 | 0 | 0 | 0 | 0 | 0 | 0 | 0 |
|  | Genus | Bai *vs.* Han | 0.444 | 0.690 | 0.559 | 0.470 | 0.783 | 0.899 | 0.471 | 0.656 |
|  |  | Bai *vs.* Kazakh | 0.487 | 0.884 | 0.435 | 0.353 | 0.955 | 0.239 | 0.082 | 0.719 |
|  |  | Bai *vs.* Mongol | 0.533 | 0.637 | 0.562 | 0.710 | 0.570 | 0.310 | 0.680 | 0.512 |
|  |  | Bai *vs.* Tibetan | 0.741 | 0.634 | 0.767 | 0.965 | 0.870 | 0.222 | 0.133 | 0.520 |
|  |  | Bai *vs.* Uyghur | 0.998 | 0.496 | 0.700 | 0.404 | 0.592 | 0.205 | 0.261 | 0.882 |
|  |  | Bai *vs.* Zhuang | 0.765 | 0.914 | 0.825 | 0.947 | 0.856 | 0.295 | 0.708 | 0.693 |
|  |  | Han *vs.* Kazakh | 0.243 | 0.871 | 0.257 | 0.268 | 0.853 | 0.531 | 0.026 | 0.433 |
|  |  | Han *vs.* Mongol | 0.962 | 0.847 | 0.951 | 0.717 | 0.735 | 0.453 | 0.833 | 0.770 |
|  |  | Han *vs.* Tibetan | 0.878 | 0.333 | 0.996 | 0.610 | 0.640 | 0.429 | 0.023 | 0.601 |
|  |  | Han *vs.* Uyghur | 0.566 | 0.762 | 0.409 | 0.284 | 0.737 | 0.457 | 0.092 | 0.805 |
|  |  | Han *vs.* Zhuang | 0.693 | 0.802 | 0.727 | 0.526 | 0.967 | 0.492 | 0.985 | 0.981 |
|  |  | Kazakh *vs.* Mongol | 0.248 | 0.760 | 0.275 | 0.305 | 0.627 | 0.806 | 0.072 | 0.384 |
|  |  | Kazakh *vs.* Tibetan | 0.517 | 0.608 | 0.473 | 0.563 | 0.838 | 0.988 | 0.041 | 0.398 |
|  |  | Kazakh *vs.* Uyghur | 0.500 | 0.621 | 0.684 | 0.946 | 0.614 | 0.623 | 0.321 | 0.581 |
|  |  | Kazakh *vs.* Zhuang | 0.377 | 0.967 | 0.384 | 0.438 | 0.869 | 0.765 | 0.146 | 0.504 |
|  |  | Mongol *vs.* Tibetan | 0.945 | 0.399 | 0.969 | 0.850 | 0.561 | 0.748 | 0.106 | 0.861 |
|  |  | Mongol *vs.* Uyghur | 0.619 | 0.877 | 0.451 | 0.354 | 0.941 | 0.652 | 0.222 | 0.633 |
|  |  | Mongol *vs.* Zhuang | 0.746 | 0.712 | 0.753 | 0.789 | 0.749 | 0.998 | 0.922 | 0.808 |
|  |  | Tibetan *vs.* Uyghur | 0.806 | 0.444 | 0.649 | 0.634 | 0.622 | 0.792 | 0.094 | 0.636 |
|  |  | Tibetan *vs.* Zhuang | 0.885 | 0.544 | 0.857 | 0.980 | 0.709 | 0.711 | 0.140 | 0.684 |
|  |  | Uyghur *vs.* Zhuang | 0.807 | 0.599 | 0.618 | 0.486 | 0.723 | 0.584 | 0.272 | 0.823 |
|  | (%) With significant differences | | 0 | 0 | 0 | 0 | 0 | 0 | 14.3% (3/21) | 0 |
|  | Species | Bai *vs.* Han | 0.870 | 0.996 | 0.972 | 0.774 | 0.991 | 0.971 | 0.367 | 0.878 |
|  |  | Bai *vs.* Kazakh | 0.220 | 0.775 | 0.326 | 0.402 | 0.773 | 0.270 | 0.100 | 0.517 |
|  |  | Bai *vs.* Mongol | 0.807 | 0.779 | 0.921 | 0.965 | 0.786 | 0.448 | 0.069 | 0.854 |
|  |  | Bai *vs.* Tibetan | 0.810 | 0.689 | 0.956 | 0.929 | 0.826 | 0.261 | 0.866 | 0.933 |
|  |  | Bai *vs.* Uyghur | 0.421 | 0.884 | 0.479 | 0.457 | 0.963 | 0.208 | 0.070 | 0.567 |
|  |  | Bai *vs.* Zhuang | 0.603 | 0.984 | 0.655 | 0.804 | 0.884 | 0.679 | 0.159 | 0.569 |
|  |  | Han *vs.* Kazakh | 0.348 | 0.817 | 0.410 | 0.445 | 0.823 | 0.443 | 0.290 | 0.640 |
|  |  | Han *vs.* Mongol | 0.964 | 0.756 | 0.950 | 0.743 | 0.786 | 0.656 | 0.445 | 0.984 |
|  |  | Han *vs.* Tibetan | 0.933 | 0.690 | 0.969 | 0.658 | 0.875 | 0.468 | 0.210 | 0.761 |
|  |  | Han *vs.* Uyghur | 0.543 | 0.894 | 0.542 | 0.505 | 0.948 | 0.397 | 0.334 | 0.768 |
|  |  | Han *vs.* Zhuang | 0.803 | 0.996 | 0.687 | 0.546 | 0.919 | 0.822 | 0.661 | 0.829 |
|  |  | Kazakh *vs.* Mongol | 0.340 | 0.603 | 0.401 | 0.445 | 0.608 | 0.518 | 0.522 | 0.572 |
|  |  | Kazakh *vs.* Tibetan | 0.476 | 0.906 | 0.530 | 0.632 | 0.965 | 0.885 | 0.212 | 0.521 |
|  |  | Kazakh *vs.* Uyghur | 0.711 | 0.705 | 0.837 | 0.945 | 0.759 | 0.712 | 0.747 | 0.831 |
|  |  | Kazakh *vs.* Zhuang | 0.356 | 0.772 | 0.497 | 0.520 | 0.871 | 0.421 | 0.389 | 0.719 |
|  |  | Mongol *vs.* Tibetan | 0.971 | 0.551 | 0.995 | 0.914 | 0.683 | 0.596 | 0.205 | 0.825 |
|  |  | Mongol *vs.* Uyghur | 0.549 | 0.875 | 0.557 | 0.492 | 0.812 | 0.364 | 0.673 | 0.718 |
|  |  | Mongol *vs.* Zhuang | 0.833 | 0.733 | 0.745 | 0.769 | 0.670 | 0.720 | 0.741 | 0.789 |
|  |  | Tibetan *vs.* Uyghur | 0.672 | 0.632 | 0.635 | 0.670 | 0.822 | 0.703 | 0.233 | 0.609 |
|  |  | Tibetan *vs.* Zhuang | 0.895 | 0.686 | 0.772 | 0.915 | 0.913 | 0.480 | 0.281 | 0.613 |
|  |  | Uyghur *vs.* Zhuang | 0.630 | 0.870 | 0.693 | 0.563 | 0.854 | 0.308 | 0.483 | 0.890 |
|  | (%) With significant differences | | 0 | 0 | 0 | 0 | 0 | 0 | 0 | 0 |
| **Scheme-2B (Rural)** | | | | | | | | | | |
| **Diversity order** | **Taxon** | **Cohort** | **PL** | | **PLEC** | | | | | |
|  |  |  | ***z*** | **ln(*c*)** | ***z*** | ***d*** | **ln(*c*)** | ***A_max_*** | ***D_max_*** | ***LGD*** |
| *q*  = 0 | Phylum | Bai-Rural *vs.* Han-Rural | 0.649 | 0.506 | 0.846 | 0.704 | 0.799 | 0.818 | 0.613 | 0.940 |
|  |  | Bai-Rural *vs.* Kazakh-Rural | 0.839 | 0.690 | 0.857 | 0.532 | 0.583 | 0.714 | 0.818 | 0.643 |
|  |  | Bai-Rural *vs.* Mongol-Rural | 0.940 | 0.435 | 0.875 | 0.808 | 0.482 | 0.414 | 0.318 | 0.792 |
|  |  | Bai-Rural *vs.* Tibetan-Rural | 0.716 | 0.978 | 0.829 | 0.920 | 0.880 | 0.762 | 0.685 | 0.903 |
|  |  | Bai-Rural *vs.* Uyghur-Rural | 0.865 | 0.539 | 0.894 | 0.815 | 0.596 | 1.000 | 0.989 | 0.894 |
|  |  | Bai-Rural *vs.* Zhuang-Rural | 0.989 | 0.893 | 0.990 | 0.957 | 0.985 | 0.661 | 0.669 | 0.821 |
|  |  | Han-Rural *vs.* Kazakh-Rural | 0.882 | 0.781 | 0.733 | 0.413 | 0.763 | 0.683 | 0.554 | 0.466 |
|  |  | Han-Rural *vs.* Mongol-Rural | 0.672 | 0.819 | 0.994 | 0.979 | 0.569 | 0.576 | 0.421 | 0.799 |
|  |  | Han-Rural *vs.* Tibetan-Rural | 0.997 | 0.521 | 0.919 | 0.864 | 0.722 | 0.941 | 0.323 | 0.785 |
|  |  | Han-Rural *vs.* Uyghur-Rural | 0.591 | 0.989 | 0.780 | 0.695 | 0.720 | 0.941 | 0.952 | 0.762 |
|  |  | Han-Rural *vs.* Zhuang-Rural | 0.640 | 0.578 | 0.863 | 0.795 | 0.804 | 0.869 | 0.929 | 0.804 |
|  |  | Kazakh-Rural *vs.* Mongol-Rural | 0.874 | 0.604 | 0.687 | 0.337 | 0.734 | 0.222 | 0.314 | 0.475 |
|  |  | Kazakh-Rural *vs.* Tibetan-Rural | 0.897 | 0.696 | 0.699 | 0.472 | 0.499 | 0.475 | 0.881 | 0.656 |
|  |  | Kazakh-Rural *vs.* Uyghur-Rural | 0.702 | 0.756 | 0.970 | 0.717 | 0.928 | 0.447 | 0.414 | 0.613 |
|  |  | Kazakh-Rural *vs.* Zhuang-Rural | 0.808 | 0.756 | 0.823 | 0.435 | 0.570 | 0.387 | 0.490 | 0.463 |
|  |  | Mongol-Rural *vs.* Tibetan-Rural | 0.725 | 0.409 | 0.945 | 0.888 | 0.403 | 0.495 | 0.176 | 0.648 |
|  |  | Mongol-Rural *vs.* Uyghur-Rural | 0.794 | 0.798 | 0.717 | 0.666 | 0.806 | 0.284 | 0.727 | 0.765 |
|  |  | Mongol-Rural *vs.* Zhuang-Rural | 0.940 | 0.411 | 0.882 | 0.859 | 0.413 | 0.587 | 0.406 | 0.979 |
|  |  | Tibetan-Rural *vs.* Uyghur-Rural | 0.616 | 0.554 | 0.766 | 0.766 | 0.504 | 0.693 | 0.907 | 0.932 |
|  |  | Tibetan-Rural *vs.* Zhuang-Rural | 0.680 | 0.900 | 0.828 | 0.951 | 0.879 | 0.918 | 0.390 | 0.701 |
|  |  | Uyghur-Rural *vs.* Zhuang-Rural | 0.836 | 0.573 | 0.870 | 0.740 | 0.562 | 0.593 | 0.914 | 0.788 |
|  | (%) With significant differences | | 0 | 0 | 0 | 0 | 0 | 0 | 0 | 0 |
|  | Family | Bai-Rural *vs.* Han-Rural | 0.762 | 0.731 | 0.542 | 0.383 | 0.720 | 0.378 | 0.387 | 0.728 |
|  |  | Bai-Rural *vs.* Kazakh-Rural | 0.739 | 0.795 | 0.955 | 0.562 | 0.624 | 0.467 | 0.474 | 0.439 |
|  |  | Bai-Rural *vs.* Mongol-Rural | 0.728 | 0.272 | 0.661 | 0.682 | 0.313 | 0.644 | 0.210 | 0.936 |
|  |  | Bai-Rural *vs.* Tibetan-Rural | 0.842 | 0.876 | 0.742 | 0.720 | 0.921 | 0.806 | 0.688 | 0.995 |
|  |  | Bai-Rural *vs.* Uyghur-Rural | 0.810 | 0.711 | 0.891 | 0.788 | 0.560 | 0.498 | 0.622 | 0.567 |
|  |  | Bai-Rural *vs.* Zhuang-Rural | 0.760 | 0.955 | 0.709 | 0.863 | 0.884 | 0.671 | 0.664 | 0.787 |
|  |  | Han-Rural *vs.* Kazakh-Rural | 0.854 | 0.977 | 0.639 | 0.331 | 0.842 | 0.232 | 0.220 | 0.204 |
|  |  | Han-Rural *vs.* Mongol-Rural | 0.915 | 0.349 | 0.995 | 0.830 | 0.439 | 0.510 | 0.840 | 0.633 |
|  |  | Han-Rural *vs.* Tibetan-Rural | 0.927 | 0.553 | 0.855 | 0.723 | 0.627 | 0.452 | 0.282 | 0.702 |
|  |  | Han-Rural *vs.* Uyghur-Rural | 0.980 | 0.960 | 0.835 | 0.564 | 0.768 | 0.227 | 0.274 | 0.309 |
|  |  | Han-Rural *vs.* Zhuang-Rural | 0.987 | 0.756 | 0.890 | 0.593 | 0.824 | 0.470 | 0.206 | 0.444 |
|  |  | Kazakh-Rural *vs.* Mongol-Rural | 0.928 | 0.369 | 0.685 | 0.373 | 0.519 | 0.306 | 0.155 | 0.378 |
|  |  | Kazakh-Rural *vs.* Tibetan-Rural | 0.798 | 0.643 | 0.739 | 0.363 | 0.510 | 0.400 | 0.457 | 0.310 |
|  |  | Kazakh-Rural *vs.* Uyghur-Rural | 0.873 | 0.947 | 0.829 | 0.717 | 0.927 | 0.944 | 0.591 | 0.763 |
|  |  | Kazakh-Rural *vs.* Zhuang-Rural | 0.856 | 0.807 | 0.707 | 0.376 | 0.697 | 0.283 | 0.421 | 0.369 |
|  |  | Mongol-Rural *vs.* Tibetan-Rural | 0.874 | 0.205 | 0.902 | 0.963 | 0.308 | 0.814 | 0.179 | 0.940 |
|  |  | Mongol-Rural *vs.* Uyghur-Rural | 0.962 | 0.344 | 0.856 | 0.632 | 0.525 | 0.308 | 0.186 | 0.495 |
|  |  | Mongol-Rural *vs.* Zhuang-Rural | 0.941 | 0.266 | 0.932 | 0.835 | 0.383 | 0.972 | 0.171 | 0.813 |
|  |  | Tibetan-Rural *vs.* Uyghur-Rural | 0.908 | 0.586 | 0.917 | 0.632 | 0.461 | 0.475 | 0.765 | 0.521 |
|  |  | Tibetan-Rural *vs.* Zhuang-Rural | 0.918 | 0.782 | 0.971 | 0.857 | 0.777 | 0.790 | 0.898 | 0.694 |
|  |  | Uyghur-Rural *vs.* Zhuang-Rural | 0.980 | 0.711 | 0.888 | 0.684 | 0.593 | 0.312 | 0.695 | 0.555 |
|  | (%) With significant differences | | 0 | 0 | 0 | 0 | 0 | 0 | 0 | 0 |
|  | Genus | Bai-Rural *vs.* Han-Rural | 0.981 | 0.803 | 0.793 | 0.540 | 0.786 | 0.317 | 0.298 | 0.550 |
|  |  | Bai-Rural *vs.* Kazakh-Rural | 0.933 | 0.861 | 0.878 | 0.568 | 0.703 | 0.442 | 0.506 | 0.432 |
|  |  | Bai-Rural *vs.* Mongol-Rural | 0.829 | 0.271 | 0.754 | 0.780 | 0.306 | 0.382 | 0.218 | 0.945 |
|  |  | Bai-Rural *vs.* Tibetan-Rural | 0.955 | 0.709 | 1.000 | 0.957 | 0.708 | 0.897 | 0.566 | 0.880 |
|  |  | Bai-Rural *vs.* Uyghur-Rural | 0.911 | 0.726 | 0.909 | 0.849 | 0.586 | 0.927 | 0.863 | 0.702 |
|  |  | Bai-Rural *vs.* Zhuang-Rural | 0.955 | 0.974 | 0.870 | 0.995 | 0.947 | 0.806 | 0.620 | 0.891 |
|  |  | Han-Rural *vs.* Kazakh-Rural | 0.931 | 0.985 | 0.739 | 0.436 | 0.834 | 0.191 | 0.236 | 0.204 |
|  |  | Han-Rural *vs.* Mongol-Rural | 0.788 | 0.269 | 0.886 | 0.882 | 0.307 | 0.492 | 0.848 | 0.597 |
|  |  | Han-Rural *vs.* Tibetan-Rural | 0.926 | 0.477 | 0.785 | 0.606 | 0.531 | 0.316 | 0.225 | 0.651 |
|  |  | Han-Rural *vs.* Uyghur-Rural | 0.912 | 0.874 | 0.969 | 0.717 | 0.693 | 0.243 | 0.433 | 0.475 |
|  |  | Han-Rural *vs.* Zhuang-Rural | 0.956 | 0.746 | 0.917 | 0.594 | 0.841 | 0.344 | 0.263 | 0.480 |
|  |  | Kazakh-Rural *vs.* Mongol-Rural | 0.907 | 0.305 | 0.634 | 0.445 | 0.390 | 0.203 | 0.156 | 0.384 |
|  |  | Kazakh-Rural *vs.* Tibetan-Rural | 0.891 | 0.556 | 0.881 | 0.540 | 0.429 | 0.560 | 0.669 | 0.323 |
|  |  | Kazakh-Rural *vs.* Uyghur-Rural | 0.986 | 0.849 | 0.783 | 0.695 | 0.887 | 0.275 | 0.309 | 0.582 |
|  |  | Kazakh-Rural *vs.* Zhuang-Rural | 0.955 | 0.799 | 0.790 | 0.510 | 0.688 | 0.323 | 0.448 | 0.391 |
|  |  | Mongol-Rural *vs.* Tibetan-Rural | 0.789 | 0.152 | 0.764 | 0.859 | 0.194 | 0.323 | 0.125 | 0.935 |
|  |  | Mongol-Rural *vs.* Uyghur-Rural | 0.934 | 0.351 | 0.884 | 0.753 | 0.437 | 0.272 | 0.293 | 0.698 |
|  |  | Mongol-Rural *vs.* Zhuang-Rural | 0.852 | 0.226 | 0.836 | 0.798 | 0.282 | 0.425 | 0.121 | 0.831 |
|  |  | Tibetan-Rural *vs.* Uyghur-Rural | 0.864 | 0.433 | 0.872 | 0.815 | 0.351 | 0.991 | 0.968 | 0.606 |
|  |  | Tibetan-Rural *vs.* Zhuang-Rural | 0.889 | 0.670 | 0.865 | 0.962 | 0.642 | 0.684 | 0.828 | 0.710 |
|  |  | Uyghur-Rural *vs.* Zhuang-Rural | 0.932 | 0.616 | 0.969 | 0.803 | 0.538 | 0.713 | 0.891 | 0.638 |
|  | (%) With significant differences | | 0 | 0 | 0 | 0 | 0 | 0 | 0 | 0 |
|  | Species | Bai-Rural *vs.* Han-Rural | 0.047 | 0.987 | 0.521 | 0.401 | 0.938 | 0.036 | 0.045 | 0.003 |
|  |  | Bai-Rural *vs.* Kazakh-Rural | 0.183 | 0.919 | 0.277 | 0.411 | 0.973 | 0.669 | 0.664 | 0.474 |
|  |  | Bai-Rural *vs.* Mongol-Rural | 0.282 | 0.312 | 0.728 | 0.737 | 0.387 | 0.653 | 0.054 | 0.077 |
|  |  | Bai-Rural *vs.* Tibetan-Rural | 0.053 | 0.584 | 0.415 | 0.837 | 0.614 | 0.611 | 0.101 | 0.029 |
|  |  | Bai-Rural *vs.* Uyghur-Rural | 0.192 | 0.936 | 0.579 | 0.835 | 0.720 | 0.919 | 0.201 | 0.338 |
|  |  | Bai-Rural *vs.* Zhuang-Rural | 0.048 | 0.899 | 0.450 | 0.846 | 0.915 | 0.406 | 0.109 | 0.036 |
|  |  | Han-Rural *vs.* Kazakh-Rural | 0.912 | 0.936 | 0.488 | 0.293 | 0.964 | 0.018 | 0.169 | 0.448 |
|  |  | Han-Rural *vs.* Mongol-Rural | 0.682 | 0.238 | 0.929 | 0.796 | 0.372 | 0.035 | 0.488 | 0.414 |
|  |  | Han-Rural *vs.* Tibetan-Rural | 0.997 | 0.588 | 0.787 | 0.567 | 0.605 | 0.039 | 0.130 | 0.617 |
|  |  | Han-Rural *vs.* Uyghur-Rural | 0.971 | 0.964 | 0.879 | 0.668 | 0.812 | 0.030 | 0.212 | 0.565 |
|  |  | Han-Rural *vs.* Zhuang-Rural | 0.932 | 0.903 | 0.881 | 0.611 | 0.874 | 0.039 | 0.177 | 0.564 |
|  |  | Kazakh-Rural *vs.* Mongol-Rural | 0.660 | 0.204 | 0.459 | 0.340 | 0.310 | 0.444 | 0.207 | 0.637 |
|  |  | Kazakh-Rural *vs.* Tibetan-Rural | 0.908 | 0.723 | 0.573 | 0.371 | 0.662 | 0.546 | 0.692 | 0.503 |
|  |  | Kazakh-Rural *vs.* Uyghur-Rural | 0.932 | 0.885 | 0.557 | 0.532 | 0.736 | 0.409 | 0.418 | 0.796 |
|  |  | Kazakh-Rural *vs.* Zhuang-Rural | 0.865 | 0.981 | 0.509 | 0.325 | 0.908 | 0.275 | 0.426 | 0.516 |
|  |  | Mongol-Rural *vs.* Tibetan-Rural | 0.728 | 0.155 | 0.816 | 0.889 | 0.239 | 0.897 | 0.216 | 0.813 |
|  |  | Mongol-Rural *vs.* Uyghur-Rural | 0.705 | 0.234 | 0.819 | 0.735 | 0.398 | 0.845 | 0.382 | 0.792 |
|  |  | Mongol-Rural *vs.* Zhuang-Rural | 0.748 | 0.198 | 0.841 | 0.899 | 0.293 | 0.616 | 0.279 | 0.800 |
|  |  | Tibetan-Rural *vs.* Uyghur-Rural | 0.974 | 0.600 | 0.984 | 0.791 | 0.453 | 0.919 | 0.946 | 0.591 |
|  |  | Tibetan-Rural *vs.* Zhuang-Rural | 0.923 | 0.690 | 0.922 | 0.997 | 0.689 | 0.589 | 0.621 | 0.937 |
|  |  | Uyghur-Rural *vs.* Zhuang-Rural | 0.918 | 0.865 | 0.968 | 0.743 | 0.655 | 0.473 | 0.792 | 0.681 |
|  | (%) With significant differences | | 9.5% (2/21) | 0 | 0 | 0 | 0 | 28.6% (6/21) | 4.8% (1/21) | 14.3% (3/21) |
| *q*  = 1 | Phylum | Bai-Rural *vs.* Han-Rural | 0.745 | 0.859 | 0.712 | 0.681 | 0.805 | 0.385 | 0.860 | 0.776 |
|  |  | Bai-Rural *vs.* Kazakh-Rural | 0.963 | 0.580 | 0.984 | 0.938 | 0.613 | 0.431 | 0.733 | 0.885 |
|  |  | Bai-Rural *vs.* Mongol-Rural | 0.662 | 0.320 | 0.723 | 0.790 | 0.402 | 0.028 | 0.264 | 0.766 |
|  |  | Bai-Rural *vs.* Tibetan-Rural | 0.996 | 0.518 | 0.956 | 0.936 | 0.558 | 0.758 | 0.271 | 0.998 |
|  |  | Bai-Rural *vs.* Uyghur-Rural | 0.534 | 0.565 | 0.577 | 0.554 | 0.440 | 0.450 | 0.043 | 0.760 |
|  |  | Bai-Rural *vs.* Zhuang-Rural | 0.644 | 0.688 | 0.660 | 0.744 | 0.694 | 0.564 | 0.895 | 0.685 |
|  |  | Han-Rural *vs.* Kazakh-Rural | 0.872 | 0.714 | 0.830 | 0.878 | 0.786 | 0.203 | 0.629 | 0.889 |
|  |  | Han-Rural *vs.* Mongol-Rural | 0.897 | 0.410 | 0.990 | 0.939 | 0.556 | 0.030 | 0.227 | 0.970 |
|  |  | Han-Rural *vs.* Tibetan-Rural | 0.757 | 0.625 | 0.776 | 0.774 | 0.729 | 0.353 | 0.194 | 0.780 |
|  |  | Han-Rural *vs.* Uyghur-Rural | 0.374 | 0.607 | 0.416 | 0.462 | 0.510 | 0.207 | 0.019 | 0.596 |
|  |  | Han-Rural *vs.* Zhuang-Rural | 0.853 | 0.812 | 0.948 | 0.976 | 0.897 | 0.509 | 0.970 | 0.942 |
|  |  | Kazakh-Rural *vs.* Mongol-Rural | 0.745 | 0.605 | 0.771 | 0.834 | 0.604 | 0.015 | 0.626 | 0.799 |
|  |  | Kazakh-Rural *vs.* Tibetan-Rural | 0.934 | 0.938 | 0.989 | 0.912 | 0.914 | 0.654 | 0.638 | 0.879 |
|  |  | Kazakh-Rural *vs.* Uyghur-Rural | 0.572 | 0.867 | 0.641 | 0.665 | 0.700 | 0.972 | 0.180 | 0.706 |
|  |  | Kazakh-Rural *vs.* Zhuang-Rural | 0.747 | 0.850 | 0.766 | 0.840 | 0.865 | 0.186 | 0.577 | 0.751 |
|  |  | Mongol-Rural *vs.* Tibetan-Rural | 0.612 | 0.708 | 0.710 | 0.822 | 0.786 | 0.019 | 0.742 | 0.695 |
|  |  | Mongol-Rural *vs.* Uyghur-Rural | 0.188 | 0.899 | 0.284 | 0.340 | 0.792 | 0.008 | 0.045 | 0.409 |
|  |  | Mongol-Rural *vs.* Zhuang-Rural | 0.954 | 0.490 | 0.946 | 0.975 | 0.546 | 0.022 | 0.266 | 0.964 |
|  |  | Tibetan-Rural *vs.* Uyghur-Rural | 0.469 | 0.875 | 0.482 | 0.460 | 0.712 | 0.639 | 0.152 | 0.728 |
|  |  | Tibetan-Rural *vs.* Zhuang-Rural | 0.620 | 0.776 | 0.694 | 0.828 | 0.761 | 0.421 | 0.171 | 0.693 |
|  |  | Uyghur-Rural *vs.* Zhuang-Rural | 0.306 | 0.699 | 0.378 | 0.456 | 0.518 | 0.195 | 0.010 | 0.511 |
|  | (%) With significant differences | | 0 | 0 | 0 | 0 | 0 | 28.6% (6/21) | 19.0% (4/21) | 0 |
|  | Family | Bai-Rural *vs.* Han-Rural | 0.958 | 0.931 | 0.979 | 0.836 | 0.991 | 0.596 | 0.645 | 0.926 |
|  |  | Bai-Rural *vs.* Kazakh-Rural | 0.622 | 0.977 | 0.509 | 0.425 | 0.912 | 0.242 | 0.879 | 0.961 |
|  |  | Bai-Rural *vs.* Mongol-Rural | 0.789 | 0.415 | 0.724 | 0.811 | 0.412 | 0.289 | 0.209 | 0.667 |
|  |  | Bai-Rural *vs.* Tibetan-Rural | 0.684 | 0.672 | 0.761 | 0.799 | 0.726 | 0.871 | 0.896 | 0.772 |
|  |  | Bai-Rural *vs.* Uyghur-Rural | 0.568 | 0.618 | 0.576 | 0.550 | 0.560 | 0.299 | 0.204 | 0.811 |
|  |  | Bai-Rural *vs.* Zhuang-Rural | 0.963 | 0.938 | 0.847 | 0.757 | 0.993 | 0.463 | 0.864 | 0.972 |
|  |  | Han-Rural *vs.* Kazakh-Rural | 0.589 | 0.902 | 0.471 | 0.347 | 0.905 | 0.231 | 0.956 | 0.902 |
|  |  | Han-Rural *vs.* Mongol-Rural | 0.789 | 0.429 | 0.714 | 0.896 | 0.389 | 0.252 | 0.222 | 0.623 |
|  |  | Han-Rural *vs.* Tibetan-Rural | 0.607 | 0.587 | 0.689 | 0.609 | 0.705 | 0.522 | 0.774 | 0.787 |
|  |  | Han-Rural *vs.* Uyghur-Rural | 0.524 | 0.631 | 0.552 | 0.510 | 0.573 | 0.278 | 0.222 | 0.879 |
|  |  | Han-Rural *vs.* Zhuang-Rural | 0.892 | 0.988 | 0.800 | 0.585 | 0.992 | 0.349 | 0.754 | 0.971 |
|  |  | Kazakh-Rural *vs.* Mongol-Rural | 0.523 | 0.426 | 0.420 | 0.379 | 0.439 | 0.765 | 0.450 | 0.734 |
|  |  | Kazakh-Rural *vs.* Tibetan-Rural | 0.902 | 0.765 | 0.711 | 0.505 | 0.700 | 0.229 | 0.940 | 0.737 |
|  |  | Kazakh-Rural *vs.* Uyghur-Rural | 0.924 | 0.568 | 0.919 | 0.851 | 0.588 | 0.463 | 0.341 | 0.764 |
|  |  | Kazakh-Rural *vs.* Zhuang-Rural | 0.610 | 0.918 | 0.497 | 0.373 | 0.927 | 0.495 | 0.899 | 0.937 |
|  |  | Mongol-Rural *vs.* Tibetan-Rural | 0.530 | 0.271 | 0.573 | 0.703 | 0.326 | 0.295 | 0.241 | 0.506 |
|  |  | Mongol-Rural *vs.* Uyghur-Rural | 0.378 | 0.684 | 0.401 | 0.463 | 0.633 | 0.985 | 0.659 | 0.449 |
|  |  | Mongol-Rural *vs.* Zhuang-Rural | 0.733 | 0.392 | 0.603 | 0.631 | 0.335 | 0.606 | 0.184 | 0.638 |
|  |  | Tibetan-Rural *vs.* Uyghur-Rural | 0.853 | 0.467 | 0.816 | 0.658 | 0.463 | 0.299 | 0.263 | 0.951 |
|  |  | Tibetan-Rural *vs.* Zhuang-Rural | 0.684 | 0.601 | 0.890 | 0.962 | 0.706 | 0.504 | 0.980 | 0.757 |
|  |  | Uyghur-Rural *vs.* Zhuang-Rural | 0.544 | 0.633 | 0.620 | 0.537 | 0.546 | 0.647 | 0.134 | 0.827 |
|  | (%) With significant differences | | 0 | 0 | 0 | 0 | 0 | 0 | 0 | 0 |
|  | Genus | Bai-Rural *vs.* Han-Rural | 0.828 | 0.910 | 0.856 | 0.662 | 0.977 | 0.408 | 0.895 | 0.965 |
|  |  | Bai-Rural *vs.* Kazakh-Rural | 0.550 | 0.890 | 0.735 | 0.667 | 0.927 | 0.323 | 0.870 | 0.962 |
|  |  | Bai-Rural *vs.* Mongol-Rural | 0.816 | 0.641 | 0.776 | 0.879 | 0.604 | 0.429 | 0.690 | 0.719 |
|  |  | Bai-Rural *vs.* Tibetan-Rural | 0.719 | 0.378 | 0.754 | 0.756 | 0.468 | 0.578 | 0.191 | 0.831 |
|  |  | Bai-Rural *vs.* Uyghur-Rural | 0.543 | 0.898 | 0.670 | 0.553 | 0.724 | 0.325 | 0.674 | 0.982 |
|  |  | Bai-Rural *vs.* Zhuang-Rural | 0.998 | 0.745 | 0.950 | 0.821 | 0.786 | 0.517 | 0.295 | 0.920 |
|  |  | Han-Rural *vs.* Kazakh-Rural | 0.488 | 0.833 | 0.602 | 0.563 | 0.889 | 0.206 | 0.760 | 0.909 |
|  |  | Han-Rural *vs.* Mongol-Rural | 0.904 | 0.666 | 0.853 | 0.856 | 0.575 | 0.250 | 0.694 | 0.638 |
|  |  | Han-Rural *vs.* Tibetan-Rural | 0.565 | 0.284 | 0.587 | 0.455 | 0.417 | 0.308 | 0.059 | 0.865 |
|  |  | Han-Rural *vs.* Uyghur-Rural | 0.472 | 0.951 | 0.598 | 0.505 | 0.672 | 0.190 | 0.732 | 0.996 |
|  |  | Han-Rural *vs.* Zhuang-Rural | 0.848 | 0.608 | 0.824 | 0.550 | 0.785 | 0.294 | 0.171 | 0.879 |
|  |  | Kazakh-Rural *vs.* Mongol-Rural | 0.465 | 0.542 | 0.596 | 0.628 | 0.615 | 0.714 | 0.606 | 0.718 |
|  |  | Kazakh-Rural *vs.* Tibetan-Rural | 0.854 | 0.536 | 0.958 | 0.820 | 0.466 | 0.620 | 0.469 | 0.829 |
|  |  | Kazakh-Rural *vs.* Uyghur-Rural | 0.993 | 0.812 | 0.940 | 0.890 | 0.791 | 0.936 | 0.456 | 0.920 |
|  |  | Kazakh-Rural *vs.* Zhuang-Rural | 0.566 | 0.873 | 0.751 | 0.695 | 0.720 | 0.667 | 0.601 | 0.982 |
|  |  | Mongol-Rural *vs.* Tibetan-Rural | 0.644 | 0.274 | 0.661 | 0.729 | 0.322 | 0.730 | 0.106 | 0.648 |
|  |  | Mongol-Rural *vs.* Uyghur-Rural | 0.439 | 0.735 | 0.577 | 0.552 | 0.832 | 0.677 | 0.895 | 0.682 |
|  |  | Mongol-Rural *vs.* Zhuang-Rural | 0.817 | 0.463 | 0.783 | 0.763 | 0.504 | 0.874 | 0.170 | 0.786 |
|  |  | Tibetan-Rural *vs.* Uyghur-Rural | 0.825 | 0.467 | 0.929 | 0.758 | 0.378 | 0.580 | 0.208 | 0.893 |
|  |  | Tibetan-Rural *vs.* Zhuang-Rural | 0.736 | 0.516 | 0.780 | 0.913 | 0.566 | 0.873 | 0.570 | 0.785 |
|  |  | Uyghur-Rural *vs.* Zhuang-Rural | 0.561 | 0.656 | 0.679 | 0.612 | 0.516 | 0.608 | 0.226 | 0.906 |
|  | (%) With significant differences | | 0 | 0 | 0 | 0 | 0 | 0 | 0 | 0 |
|  | Species | Bai-Rural *vs.* Han-Rural | 0.677 | 0.820 | 0.756 | 0.902 | 0.965 | 0.525 | 0.074 | 0.538 |
|  |  | Bai-Rural *vs.* Kazakh-Rural | 0.426 | 0.853 | 0.410 | 0.413 | 0.926 | 0.409 | 0.849 | 0.778 |
|  |  | Bai-Rural *vs.* Mongol-Rural | 0.952 | 0.329 | 0.941 | 0.927 | 0.383 | 0.597 | 0.035 | 0.931 |
|  |  | Bai-Rural *vs.* Tibetan-Rural | 0.442 | 0.637 | 0.567 | 0.741 | 0.616 | 0.341 | 0.416 | 0.480 |
|  |  | Bai-Rural *vs.* Uyghur-Rural | 0.407 | 0.750 | 0.562 | 0.625 | 0.745 | 0.484 | 0.221 | 0.681 |
|  |  | Bai-Rural *vs.* Zhuang-Rural | 0.626 | 0.988 | 0.700 | 0.797 | 0.900 | 0.955 | 0.158 | 0.570 |
|  |  | Han-Rural *vs.* Kazakh-Rural | 0.667 | 0.746 | 0.634 | 0.497 | 0.964 | 0.303 | 0.385 | 0.850 |
|  |  | Han-Rural *vs.* Mongol-Rural | 0.774 | 0.429 | 0.718 | 0.984 | 0.384 | 0.387 | 0.387 | 0.620 |
|  |  | Han-Rural *vs.* Tibetan-Rural | 0.663 | 0.523 | 0.770 | 0.657 | 0.671 | 0.770 | 0.265 | 0.861 |
|  |  | Han-Rural *vs.* Uyghur-Rural | 0.588 | 0.920 | 0.699 | 0.664 | 0.735 | 0.313 | 0.989 | 0.946 |
|  |  | Han-Rural *vs.* Zhuang-Rural | 0.944 | 0.860 | 0.970 | 0.731 | 0.976 | 0.617 | 0.235 | 0.938 |
|  |  | Kazakh-Rural *vs.* Mongol-Rural | 0.526 | 0.283 | 0.475 | 0.466 | 0.309 | 0.646 | 0.171 | 0.774 |
|  |  | Kazakh-Rural *vs.* Tibetan-Rural | 0.922 | 0.832 | 0.824 | 0.633 | 0.728 | 0.160 | 0.782 | 0.743 |
|  |  | Kazakh-Rural *vs.* Uyghur-Rural | 0.932 | 0.650 | 0.917 | 0.844 | 0.687 | 0.813 | 0.304 | 0.860 |
|  |  | Kazakh-Rural *vs.* Zhuang-Rural | 0.631 | 0.825 | 0.570 | 0.418 | 0.999 | 0.413 | 0.662 | 0.866 |
|  |  | Mongol-Rural *vs.* Tibetan-Rural | 0.590 | 0.261 | 0.661 | 0.787 | 0.297 | 0.181 | 0.144 | 0.560 |
|  |  | Mongol-Rural *vs.* Uyghur-Rural | 0.407 | 0.438 | 0.512 | 0.598 | 0.440 | 0.742 | 0.573 | 0.611 |
|  |  | Mongol-Rural *vs.* Zhuang-Rural | 0.736 | 0.303 | 0.715 | 0.785 | 0.334 | 0.551 | 0.088 | 0.658 |
|  |  | Tibetan-Rural *vs.* Uyghur-Rural | 0.884 | 0.557 | 0.918 | 0.807 | 0.496 | 0.157 | 0.565 | 0.840 |
|  |  | Tibetan-Rural *vs.* Zhuang-Rural | 0.708 | 0.618 | 0.783 | 0.929 | 0.654 | 0.344 | 0.992 | 0.791 |
|  |  | Uyghur-Rural *vs.* Zhuang-Rural | 0.564 | 0.774 | 0.728 | 0.642 | 0.676 | 0.473 | 0.373 | 0.986 |
|  | (%) With significant differences | | 0 | 0 | 0 | 0 | 0 | 0 | 4.8% (1/21) | 0 |
| *q* = 2 | Phylum | Bai-Rural *vs.* Han-Rural | 0.755 | 0.902 | 0.757 | 0.728 | 0.883 | 0.324 | 0.747 | 0.836 |
|  |  | Bai-Rural *vs.* Kazakh-Rural | 0.887 | 0.724 | 0.915 | 0.949 | 0.816 | 0.304 | 0.934 | 0.903 |
|  |  | Bai-Rural *vs.* Mongol-Rural | 0.818 | 0.420 | 0.832 | 0.862 | 0.482 | 0.770 | 0.228 | 0.896 |
|  |  | Bai-Rural *vs.* Tibetan-Rural | 0.890 | 0.460 | 0.953 | 0.985 | 0.512 | 0.498 | 0.065 | 0.923 |
|  |  | Bai-Rural *vs.* Uyghur-Rural | 0.831 | 0.401 | 0.724 | 0.656 | 0.391 | 0.256 | 0.026 | 0.922 |
|  |  | Bai-Rural *vs.* Zhuang-Rural | 0.709 | 0.749 | 0.741 | 0.843 | 0.757 | 0.379 | 0.917 | 0.721 |
|  |  | Han-Rural *vs.* Kazakh-Rural | 0.961 | 0.835 | 0.891 | 0.997 | 0.946 | 0.163 | 0.749 | 0.925 |
|  |  | Han-Rural *vs.* Mongol-Rural | 0.937 | 0.548 | 0.911 | 0.904 | 0.631 | 0.257 | 0.210 | 0.923 |
|  |  | Han-Rural *vs.* Tibetan-Rural | 0.685 | 0.521 | 0.749 | 0.802 | 0.619 | 0.239 | 0.067 | 0.759 |
|  |  | Han-Rural *vs.* Uyghur-Rural | 0.653 | 0.413 | 0.591 | 0.581 | 0.407 | 0.175 | 0.020 | 0.770 |
|  |  | Han-Rural *vs.* Zhuang-Rural | 0.910 | 0.855 | 0.997 | 0.943 | 0.869 | 0.222 | 0.857 | 0.920 |
|  |  | Kazakh-Rural *vs.* Mongol-Rural | 1.000 | 0.648 | 0.957 | 0.980 | 0.557 | 0.398 | 0.426 | 0.995 |
|  |  | Kazakh-Rural *vs.* Tibetan-Rural | 0.792 | 0.669 | 0.901 | 0.943 | 0.612 | 0.794 | 0.222 | 0.760 |
|  |  | Kazakh-Rural *vs.* Uyghur-Rural | 0.810 | 0.633 | 0.761 | 0.731 | 0.543 | 0.404 | 0.137 | 0.852 |
|  |  | Kazakh-Rural *vs.* Zhuang-Rural | 0.887 | 0.961 | 0.876 | 0.976 | 0.928 | 0.945 | 0.847 | 0.791 |
|  |  | Mongol-Rural *vs.* Tibetan-Rural | 0.658 | 0.883 | 0.739 | 0.866 | 0.878 | 0.579 | 0.324 | 0.739 |
|  |  | Mongol-Rural *vs.* Uyghur-Rural | 0.613 | 0.624 | 0.533 | 0.528 | 0.568 | 0.259 | 0.041 | 0.740 |
|  |  | Mongol-Rural *vs.* Zhuang-Rural | 0.793 | 0.612 | 0.903 | 0.979 | 0.641 | 0.462 | 0.340 | 0.778 |
|  |  | Tibetan-Rural *vs.* Uyghur-Rural | 0.879 | 0.752 | 0.740 | 0.630 | 0.722 | 0.535 | 0.279 | 0.993 |
|  |  | Tibetan-Rural *vs.* Zhuang-Rural | 0.623 | 0.640 | 0.740 | 0.882 | 0.644 | 0.784 | 0.096 | 0.671 |
|  |  | Uyghur-Rural *vs.* Zhuang-Rural | 0.634 | 0.466 | 0.578 | 0.614 | 0.423 | 0.588 | 0.018 | 0.662 |
|  | (%) With significant differences | | 0 | 0 | 0 | 0 | 0 | 0 | 19.0% (4/21) | 0 |
|  | Family | Bai-Rural *vs.* Han-Rural | 0.984 | 0.966 | 0.991 | 0.875 | 0.995 | 0.518 | 0.751 | 0.899 |
|  |  | Bai-Rural *vs.* Kazakh-Rural | 0.755 | 0.900 | 0.587 | 0.476 | 0.947 | 0.866 | 0.924 | 0.855 |
|  |  | Bai-Rural *vs.* Mongol-Rural | 0.780 | 0.524 | 0.798 | 0.885 | 0.548 | 0.538 | 0.541 | 0.716 |
|  |  | Bai-Rural *vs.* Tibetan-Rural | 0.726 | 0.653 | 0.733 | 0.693 | 0.705 | 0.886 | 0.981 | 0.797 |
|  |  | Bai-Rural *vs.* Uyghur-Rural | 0.473 | 0.710 | 0.489 | 0.532 | 0.691 | 0.377 | 0.247 | 0.645 |
|  |  | Bai-Rural *vs.* Zhuang-Rural | 0.819 | 0.996 | 0.776 | 0.765 | 0.947 | 0.909 | 0.714 | 0.805 |
|  |  | Han-Rural *vs.* Kazakh-Rural | 0.724 | 0.813 | 0.493 | 0.388 | 0.921 | 0.506 | 0.928 | 0.755 |
|  |  | Han-Rural *vs.* Mongol-Rural | 0.710 | 0.505 | 0.777 | 0.948 | 0.506 | 0.355 | 0.568 | 0.625 |
|  |  | Han-Rural *vs.* Tibetan-Rural | 0.685 | 0.587 | 0.664 | 0.523 | 0.696 | 0.597 | 0.671 | 0.875 |
|  |  | Han-Rural *vs.* Uyghur-Rural | 0.392 | 0.699 | 0.446 | 0.471 | 0.671 | 0.293 | 0.201 | 0.739 |
|  |  | Han-Rural *vs.* Zhuang-Rural | 0.746 | 0.944 | 0.715 | 0.596 | 0.922 | 0.492 | 0.939 | 0.866 |
|  |  | Kazakh-Rural *vs.* Mongol-Rural | 0.634 | 0.496 | 0.505 | 0.456 | 0.522 | 0.601 | 0.734 | 0.855 |
|  |  | Kazakh-Rural *vs.* Tibetan-Rural | 0.948 | 0.831 | 0.744 | 0.565 | 0.802 | 0.831 | 0.921 | 0.700 |
|  |  | Kazakh-Rural *vs.* Uyghur-Rural | 0.749 | 0.638 | 0.952 | 0.924 | 0.662 | 0.197 | 0.454 | 0.583 |
|  |  | Kazakh-Rural *vs.* Zhuang-Rural | 0.850 | 0.876 | 0.581 | 0.416 | 0.993 | 0.989 | 0.891 | 0.707 |
|  |  | Mongol-Rural *vs.* Tibetan-Rural | 0.547 | 0.316 | 0.586 | 0.675 | 0.406 | 0.519 | 0.422 | 0.554 |
|  |  | Mongol-Rural *vs.* Uyghur-Rural | 0.316 | 0.727 | 0.388 | 0.494 | 0.718 | 0.722 | 0.512 | 0.425 |
|  |  | Mongol-Rural *vs.* Zhuang-Rural | 0.566 | 0.432 | 0.594 | 0.677 | 0.422 | 0.636 | 0.564 | 0.542 |
|  |  | Tibetan-Rural *vs.* Uyghur-Rural | 0.709 | 0.509 | 0.720 | 0.653 | 0.520 | 0.379 | 0.243 | 0.869 |
|  |  | Tibetan-Rural *vs.* Zhuang-Rural | 0.870 | 0.634 | 0.901 | 0.914 | 0.752 | 0.798 | 0.663 | 0.978 |
|  |  | Uyghur-Rural *vs.* Zhuang-Rural | 0.487 | 0.696 | 0.542 | 0.502 | 0.648 | 0.478 | 0.164 | 0.824 |
|  | (%) With significant differences | | 0 | 0 | 0 | 0 | 0 | 0 | 0 | 0 |
|  | Genus | Bai-Rural *vs.* Han-Rural | 0.889 | 0.940 | 0.878 | 0.670 | 0.853 | 0.979 | 0.784 | 0.963 |
|  |  | Bai-Rural *vs.* Kazakh-Rural | 0.470 | 0.861 | 0.687 | 0.681 | 0.970 | 0.170 | 0.950 | 0.986 |
|  |  | Bai-Rural *vs.* Mongol-Rural | 0.886 | 0.826 | 0.803 | 0.896 | 0.741 | 0.229 | 0.989 | 0.741 |
|  |  | Bai-Rural *vs.* Tibetan-Rural | 0.819 | 0.288 | 0.856 | 0.812 | 0.409 | 0.301 | 0.086 | 0.951 |
|  |  | Bai-Rural *vs.* Uyghur-Rural | 0.434 | 0.992 | 0.518 | 0.502 | 0.836 | 0.268 | 0.522 | 0.830 |
|  |  | Bai-Rural *vs.* Zhuang-Rural | 0.897 | 0.638 | 0.980 | 0.894 | 0.792 | 0.264 | 0.341 | 0.886 |
|  |  | Han-Rural *vs.* Kazakh-Rural | 0.414 | 0.914 | 0.611 | 0.635 | 0.818 | 0.234 | 0.960 | 0.959 |
|  |  | Han-Rural *vs.* Mongol-Rural | 0.964 | 0.770 | 0.875 | 0.839 | 0.623 | 0.326 | 0.809 | 0.657 |
|  |  | Han-Rural *vs.* Tibetan-Rural | 0.711 | 0.262 | 0.753 | 0.533 | 0.423 | 0.367 | 0.039 | 0.898 |
|  |  | Han-Rural *vs.* Uyghur-Rural | 0.384 | 0.929 | 0.475 | 0.460 | 0.716 | 0.463 | 0.377 | 0.880 |
|  |  | Han-Rural *vs.* Zhuang-Rural | 0.775 | 0.640 | 0.876 | 0.625 | 0.892 | 0.341 | 0.333 | 0.807 |
|  |  | Kazakh-Rural *vs.* Mongol-Rural | 0.443 | 0.698 | 0.600 | 0.668 | 0.738 | 0.562 | 0.939 | 0.719 |
|  |  | Kazakh-Rural *vs.* Tibetan-Rural | 0.763 | 0.466 | 0.897 | 0.876 | 0.470 | 0.515 | 0.261 | 0.975 |
|  |  | Kazakh-Rural *vs.* Uyghur-Rural | 0.984 | 0.868 | 0.827 | 0.806 | 0.873 | 0.206 | 0.494 | 0.845 |
|  |  | Kazakh-Rural *vs.* Zhuang-Rural | 0.550 | 0.792 | 0.742 | 0.776 | 0.755 | 0.464 | 0.533 | 0.929 |
|  |  | Mongol-Rural *vs.* Tibetan-Rural | 0.779 | 0.284 | 0.778 | 0.766 | 0.356 | 0.866 | 0.080 | 0.829 |
|  |  | Mongol-Rural *vs.* Uyghur-Rural | 0.417 | 0.854 | 0.498 | 0.500 | 0.901 | 0.673 | 0.476 | 0.623 |
|  |  | Mongol-Rural *vs.* Zhuang-Rural | 0.802 | 0.540 | 0.826 | 0.835 | 0.607 | 0.867 | 0.301 | 0.852 |
|  |  | Tibetan-Rural *vs.* Uyghur-Rural | 0.723 | 0.430 | 0.779 | 0.722 | 0.420 | 0.812 | 0.082 | 0.836 |
|  |  | Tibetan-Rural *vs.* Zhuang-Rural | 0.898 | 0.474 | 0.880 | 0.914 | 0.512 | 0.967 | 0.285 | 0.973 |
|  |  | Uyghur-Rural *vs.* Zhuang-Rural | 0.527 | 0.662 | 0.582 | 0.565 | 0.638 | 0.826 | 0.190 | 0.760 |
|  | (%) With significant differences | | 0 | 0 | 0 | 0 | 0 | 0 | 4.8% (1/21) | 0 |
|  | Species | Bai-Rural *vs.* Han-Rural | 0.675 | 0.939 | 0.729 | 0.977 | 0.780 | 0.910 | 0.283 | 0.632 |
|  |  | Bai-Rural *vs.* Kazakh-Rural | 0.338 | 0.692 | 0.426 | 0.514 | 0.782 | 0.244 | 0.842 | 0.751 |
|  |  | Bai-Rural *vs.* Mongol-Rural | 0.877 | 0.496 | 0.981 | 0.994 | 0.469 | 0.271 | 0.047 | 0.943 |
|  |  | Bai-Rural *vs.* Tibetan-Rural | 0.545 | 0.455 | 0.652 | 0.722 | 0.544 | 0.428 | 0.844 | 0.761 |
|  |  | Bai-Rural *vs.* Uyghur-Rural | 0.148 | 0.724 | 0.381 | 0.545 | 0.925 | 0.247 | 0.191 | 0.529 |
|  |  | Bai-Rural *vs.* Zhuang-Rural | 0.667 | 0.888 | 0.637 | 0.710 | 0.727 | 0.512 | 0.387 | 0.590 |
|  |  | Han-Rural *vs.* Kazakh-Rural | 0.623 | 0.853 | 0.712 | 0.638 | 0.977 | 0.337 | 0.636 | 0.914 |
|  |  | Han-Rural *vs.* Mongol-Rural | 0.838 | 0.526 | 0.780 | 0.967 | 0.433 | 0.374 | 0.338 | 0.679 |
|  |  | Han-Rural *vs.* Tibetan-Rural | 0.811 | 0.543 | 0.878 | 0.669 | 0.749 | 0.549 | 0.272 | 0.885 |
|  |  | Han-Rural *vs.* Uyghur-Rural | 0.341 | 0.843 | 0.587 | 0.637 | 0.901 | 0.367 | 0.683 | 0.863 |
|  |  | Han-Rural *vs.* Zhuang-Rural | 0.985 | 0.967 | 0.944 | 0.757 | 0.974 | 0.713 | 0.462 | 0.996 |
|  |  | Kazakh-Rural *vs.* Mongol-Rural | 0.494 | 0.365 | 0.543 | 0.599 | 0.367 | 0.085 | 0.264 | 0.748 |
|  |  | Kazakh-Rural *vs.* Tibetan-Rural | 0.815 | 0.747 | 0.818 | 0.776 | 0.734 | 0.570 | 0.803 | 0.996 |
|  |  | Kazakh-Rural *vs.* Uyghur-Rural | 0.636 | 0.993 | 0.908 | 0.987 | 0.895 | 0.974 | 0.412 | 0.806 |
|  |  | Kazakh-Rural *vs.* Zhuang-Rural | 0.489 | 0.782 | 0.664 | 0.581 | 0.946 | 0.190 | 0.926 | 0.919 |
|  |  | Mongol-Rural *vs.* Tibetan-Rural | 0.743 | 0.292 | 0.740 | 0.794 | 0.337 | 0.149 | 0.115 | 0.809 |
|  |  | Mongol-Rural *vs.* Uyghur-Rural | 0.217 | 0.345 | 0.421 | 0.572 | 0.395 | 0.073 | 0.710 | 0.538 |
|  |  | Mongol-Rural *vs.* Zhuang-Rural | 0.857 | 0.436 | 0.695 | 0.798 | 0.350 | 0.430 | 0.078 | 0.674 |
|  |  | Tibetan-Rural *vs.* Uyghur-Rural | 0.484 | 0.733 | 0.718 | 0.751 | 0.671 | 0.549 | 0.289 | 0.792 |
|  |  | Tibetan-Rural *vs.* Zhuang-Rural | 0.807 | 0.518 | 0.931 | 0.928 | 0.752 | 0.274 | 0.461 | 0.892 |
|  |  | Uyghur-Rural *vs.* Zhuang-Rural | 0.251 | 0.852 | 0.553 | 0.569 | 0.872 | 0.158 | 0.190 | 0.845 |
|  | (%) With significant differences | | 0 | 0 | 0 | 0 | 0 | 0 | 4.8% (1/21) | 0 |
| *q*  = 3 | Phylum | Bai-Rural *vs.* Han-Rural | 0.783 | 0.927 | 0.749 | 0.756 | 0.871 | 0.591 | 0.704 | 0.763 |
|  |  | Bai-Rural *vs.* Kazakh-Rural | 0.859 | 0.772 | 0.849 | 0.874 | 0.803 | 0.245 | 0.975 | 0.820 |
|  |  | Bai-Rural *vs.* Mongol-Rural | 0.864 | 0.459 | 0.882 | 0.888 | 0.517 | 0.781 | 0.199 | 0.945 |
|  |  | Bai-Rural *vs.* Tibetan-Rural | 0.768 | 0.474 | 0.909 | 0.947 | 0.492 | 0.739 | 0.029 | 0.881 |
|  |  | Bai-Rural *vs.* Uyghur-Rural | 0.988 | 0.336 | 0.923 | 0.817 | 0.340 | 0.318 | 0.017 | 0.836 |
|  |  | Bai-Rural *vs.* Zhuang-Rural | 0.755 | 0.770 | 0.763 | 0.875 | 0.745 | 0.450 | 0.912 | 0.683 |
|  |  | Han-Rural *vs.* Kazakh-Rural | 1.000 | 0.850 | 0.978 | 0.929 | 0.954 | 0.211 | 0.740 | 0.967 |
|  |  | Han-Rural *vs.* Mongol-Rural | 0.918 | 0.565 | 0.860 | 0.886 | 0.681 | 0.472 | 0.171 | 0.843 |
|  |  | Han-Rural *vs.* Tibetan-Rural | 0.614 | 0.522 | 0.705 | 0.771 | 0.575 | 0.486 | 0.030 | 0.703 |
|  |  | Han-Rural *vs.* Uyghur-Rural | 0.886 | 0.326 | 0.775 | 0.737 | 0.362 | 0.279 | 0.019 | 0.999 |
|  |  | Han-Rural *vs.* Zhuang-Rural | 0.937 | 0.873 | 0.988 | 0.925 | 0.882 | 0.344 | 0.787 | 0.885 |
|  |  | Kazakh-Rural *vs.* Mongol-Rural | 0.939 | 0.662 | 0.909 | 0.882 | 0.636 | 0.310 | 0.366 | 0.817 |
|  |  | Kazakh-Rural *vs.* Tibetan-Rural | 0.711 | 0.637 | 0.807 | 0.884 | 0.581 | 0.294 | 0.155 | 0.697 |
|  |  | Kazakh-Rural *vs.* Uyghur-Rural | 0.922 | 0.555 | 0.848 | 0.819 | 0.516 | 0.577 | 0.129 | 0.980 |
|  |  | Kazakh-Rural *vs.* Zhuang-Rural | 0.952 | 0.982 | 0.988 | 0.879 | 0.932 | 0.505 | 0.916 | 0.822 |
|  |  | Mongol-Rural *vs.* Tibetan-Rural | 0.610 | 0.828 | 0.745 | 0.870 | 0.760 | 0.951 | 0.197 | 0.758 |
|  |  | Mongol-Rural *vs.* Uyghur-Rural | 0.897 | 0.477 | 0.785 | 0.747 | 0.462 | 0.335 | 0.054 | 0.824 |
|  |  | Mongol-Rural *vs.* Zhuang-Rural | 0.819 | 0.630 | 0.866 | 0.979 | 0.712 | 0.558 | 0.345 | 0.671 |
|  |  | Tibetan-Rural *vs.* Uyghur-Rural | 0.853 | 0.677 | 0.967 | 0.816 | 0.731 | 0.427 | 0.569 | 0.765 |
|  |  | Tibetan-Rural *vs.* Zhuang-Rural | 0.596 | 0.645 | 0.736 | 0.889 | 0.641 | 0.597 | 0.080 | 0.604 |
|  |  | Uyghur-Rural *vs.* Zhuang-Rural | 0.864 | 0.375 | 0.770 | 0.780 | 0.384 | 0.667 | 0.028 | 0.895 |
|  | (%) With significant differences | | 0 | 0 | 0 | 0 | 0 | 0 | 23.8% (5/21) | 0 |
|  | Family | Bai-Rural *vs.* Han-Rural | 0.835 | 1.000 | 0.947 | 0.880 | 0.995 | 0.564 | 0.648 | 0.759 |
|  |  | Bai-Rural *vs.* Kazakh-Rural | 0.889 | 0.899 | 0.677 | 0.556 | 0.875 | 0.549 | 0.799 | 0.993 |
|  |  | Bai-Rural *vs.* Mongol-Rural | 0.830 | 0.593 | 0.851 | 0.900 | 0.629 | 0.897 | 0.635 | 0.802 |
|  |  | Bai-Rural *vs.* Tibetan-Rural | 0.680 | 0.608 | 0.694 | 0.664 | 0.681 | 0.512 | 0.905 | 0.753 |
|  |  | Bai-Rural *vs.* Uyghur-Rural | 0.349 | 0.866 | 0.374 | 0.480 | 0.858 | 0.489 | 0.220 | 0.446 |
|  |  | Bai-Rural *vs.* Zhuang-Rural | 0.663 | 0.956 | 0.700 | 0.808 | 0.890 | 0.855 | 0.558 | 0.602 |
|  |  | Han-Rural *vs.* Kazakh-Rural | 0.972 | 0.862 | 0.585 | 0.453 | 0.855 | 0.901 | 0.957 | 0.771 |
|  |  | Han-Rural *vs.* Mongol-Rural | 0.662 | 0.547 | 0.793 | 0.970 | 0.588 | 0.530 | 0.805 | 0.608 |
|  |  | Han-Rural *vs.* Tibetan-Rural | 0.758 | 0.546 | 0.674 | 0.514 | 0.669 | 0.996 | 0.638 | 0.928 |
|  |  | Han-Rural *vs.* Uyghur-Rural | 0.328 | 0.827 | 0.332 | 0.406 | 0.830 | 0.389 | 0.218 | 0.624 |
|  |  | Han-Rural *vs.* Zhuang-Rural | 0.711 | 0.921 | 0.682 | 0.641 | 0.861 | 0.709 | 0.854 | 0.757 |
|  |  | Kazakh-Rural *vs.* Mongol-Rural | 0.777 | 0.577 | 0.597 | 0.538 | 0.581 | 0.453 | 0.882 | 0.815 |
|  |  | Kazakh-Rural *vs.* Tibetan-Rural | 0.882 | 0.787 | 0.809 | 0.619 | 0.831 | 0.834 | 0.843 | 0.756 |
|  |  | Kazakh-Rural *vs.* Uyghur-Rural | 0.564 | 0.805 | 0.780 | 0.965 | 0.770 | 0.130 | 0.514 | 0.532 |
|  |  | Kazakh-Rural *vs.* Zhuang-Rural | 0.865 | 0.911 | 0.720 | 0.487 | 0.974 | 0.785 | 0.870 | 0.657 |
|  |  | Mongol-Rural *vs.* Tibetan-Rural | 0.546 | 0.332 | 0.613 | 0.672 | 0.449 | 0.467 | 0.586 | 0.577 |
|  |  | Mongol-Rural *vs.* Uyghur-Rural | 0.247 | 0.664 | 0.338 | 0.461 | 0.689 | 0.489 | 0.455 | 0.357 |
|  |  | Mongol-Rural *vs.* Zhuang-Rural | 0.481 | 0.462 | 0.594 | 0.727 | 0.480 | 0.775 | 0.901 | 0.464 |
|  |  | Tibetan-Rural *vs.* Uyghur-Rural | 0.565 | 0.567 | 0.564 | 0.566 | 0.611 | 0.247 | 0.219 | 0.721 |
|  |  | Tibetan-Rural *vs.* Zhuang-Rural | 1.000 | 0.615 | 0.944 | 0.881 | 0.782 | 0.629 | 0.533 | 0.875 |
|  |  | Uyghur-Rural *vs.* Zhuang-Rural | 0.426 | 0.798 | 0.410 | 0.384 | 0.751 | 0.452 | 0.180 | 0.788 |
|  | (%) With significant differences | | 0 | 0 | 0 | 0 | 0 | 0 | 0 | 0 |
|  | Genus | Bai-Rural *vs.* Han-Rural | 0.907 | 0.909 | 0.858 | 0.668 | 0.856 | 0.353 | 0.839 | 0.954 |
|  |  | Bai-Rural *vs.* Kazakh-Rural | 0.449 | 0.875 | 0.764 | 0.801 | 0.902 | 0.596 | 0.908 | 0.996 |
|  |  | Bai-Rural *vs.* Mongol-Rural | 0.974 | 0.954 | 0.790 | 0.873 | 0.801 | 0.829 | 0.916 | 0.768 |
|  |  | Bai-Rural *vs.* Tibetan-Rural | 0.953 | 0.320 | 0.998 | 0.830 | 0.478 | 0.499 | 0.077 | 0.712 |
|  |  | Bai-Rural *vs.* Uyghur-Rural | 0.381 | 0.967 | 0.550 | 0.556 | 0.827 | 0.405 | 0.573 | 0.859 |
|  |  | Bai-Rural *vs.* Zhuang-Rural | 0.831 | 0.600 | 0.990 | 0.909 | 0.812 | 0.329 | 0.472 | 0.914 |
|  |  | Han-Rural *vs.* Kazakh-Rural | 0.404 | 0.933 | 0.678 | 0.739 | 0.799 | 0.211 | 0.821 | 0.963 |
|  |  | Han-Rural *vs.* Mongol-Rural | 0.934 | 0.865 | 0.896 | 0.874 | 0.698 | 0.312 | 0.953 | 0.693 |
|  |  | Han-Rural *vs.* Tibetan-Rural | 0.999 | 0.318 | 0.907 | 0.580 | 0.498 | 0.260 | 0.049 | 0.631 |
|  |  | Han-Rural *vs.* Uyghur-Rural | 0.340 | 0.942 | 0.489 | 0.501 | 0.706 | 0.180 | 0.438 | 0.898 |
|  |  | Han-Rural *vs.* Zhuang-Rural | 0.742 | 0.618 | 0.870 | 0.671 | 0.897 | 0.734 | 0.474 | 0.848 |
|  |  | Kazakh-Rural *vs.* Mongol-Rural | 0.494 | 0.819 | 0.696 | 0.772 | 0.844 | 0.557 | 0.848 | 0.752 |
|  |  | Kazakh-Rural *vs.* Tibetan-Rural | 0.640 | 0.492 | 0.864 | 0.925 | 0.508 | 0.937 | 0.185 | 0.774 |
|  |  | Kazakh-Rural *vs.* Uyghur-Rural | 0.949 | 0.906 | 0.805 | 0.801 | 0.908 | 0.436 | 0.686 | 0.872 |
|  |  | Kazakh-Rural *vs.* Zhuang-Rural | 0.577 | 0.746 | 0.817 | 0.864 | 0.724 | 0.129 | 0.525 | 0.943 |
|  |  | Mongol-Rural *vs.* Tibetan-Rural | 0.965 | 0.365 | 0.896 | 0.787 | 0.439 | 0.566 | 0.107 | 0.951 |
|  |  | Mongol-Rural *vs.* Uyghur-Rural | 0.434 | 0.919 | 0.522 | 0.547 | 0.959 | 0.284 | 0.470 | 0.657 |
|  |  | Mongol-Rural *vs.* Zhuang-Rural | 0.838 | 0.594 | 0.840 | 0.835 | 0.643 | 0.217 | 0.512 | 0.856 |
|  |  | Tibetan-Rural *vs.* Uyghur-Rural | 0.574 | 0.479 | 0.725 | 0.763 | 0.458 | 0.718 | 0.069 | 0.691 |
|  |  | Tibetan-Rural *vs.* Zhuang-Rural | 0.851 | 0.543 | 0.992 | 0.944 | 0.592 | 0.191 | 0.203 | 0.805 |
|  |  | Uyghur-Rural *vs.* Zhuang-Rural | 0.523 | 0.651 | 0.612 | 0.628 | 0.634 | 0.107 | 0.302 | 0.793 |
|  | (%) With significant differences | | 0 | 0 | 0 | 0 | 0 | 0 | 4.8% (1/21) | 0 |
|  | Species | Bai-Rural *vs.* Han-Rural | 0.632 | 0.766 | 0.734 | 0.973 | 0.691 | 0.733 | 0.449 | 0.621 |
|  |  | Bai-Rural *vs.* Kazakh-Rural | 0.257 | 0.581 | 0.401 | 0.570 | 0.630 | 0.567 | 0.796 | 0.655 |
|  |  | Bai-Rural *vs.* Mongol-Rural | 0.980 | 0.556 | 0.909 | 0.998 | 0.464 | 0.844 | 0.134 | 0.785 |
|  |  | Bai-Rural *vs.* Tibetan-Rural | 0.680 | 0.423 | 0.736 | 0.770 | 0.502 | 0.935 | 0.619 | 0.911 |
|  |  | Bai-Rural *vs.* Uyghur-Rural | 0.089 | 0.524 | 0.311 | 0.529 | 0.688 | 0.884 | 0.037 | 0.416 |
|  |  | Bai-Rural *vs.* Zhuang-Rural | 0.612 | 0.708 | 0.634 | 0.705 | 0.611 | 0.910 | 0.773 | 0.605 |
|  |  | Han-Rural *vs.* Kazakh-Rural | 0.591 | 0.902 | 0.714 | 0.701 | 0.984 | 0.486 | 0.798 | 0.980 |
|  |  | Han-Rural *vs.* Mongol-Rural | 0.712 | 0.495 | 0.723 | 0.984 | 0.404 | 0.636 | 0.461 | 0.560 |
|  |  | Han-Rural *vs.* Tibetan-Rural | 0.996 | 0.597 | 0.955 | 0.682 | 0.781 | 0.672 | 0.243 | 0.727 |
|  |  | Han-Rural *vs.* Uyghur-Rural | 0.299 | 0.805 | 0.549 | 0.646 | 0.973 | 0.670 | 0.273 | 0.789 |
|  |  | Han-Rural *vs.* Zhuang-Rural | 0.950 | 0.990 | 0.925 | 0.769 | 0.989 | 0.789 | 0.475 | 0.979 |
|  |  | Kazakh-Rural *vs.* Mongol-Rural | 0.407 | 0.352 | 0.518 | 0.672 | 0.318 | 0.510 | 0.469 | 0.596 |
|  |  | Kazakh-Rural *vs.* Tibetan-Rural | 0.663 | 0.771 | 0.784 | 0.822 | 0.781 | 0.516 | 0.622 | 0.804 |
|  |  | Kazakh-Rural *vs.* Uyghur-Rural | 0.606 | 0.919 | 0.865 | 0.929 | 0.988 | 0.429 | 0.274 | 0.768 |
|  |  | Kazakh-Rural *vs.* Zhuang-Rural | 0.456 | 0.846 | 0.672 | 0.665 | 0.969 | 0.526 | 0.881 | 0.993 |
|  |  | Mongol-Rural *vs.* Tibetan-Rural | 0.783 | 0.309 | 0.753 | 0.806 | 0.324 | 0.925 | 0.161 | 0.832 |
|  |  | Mongol-Rural *vs.* Uyghur-Rural | 0.157 | 0.287 | 0.365 | 0.601 | 0.314 | 0.931 | 0.647 | 0.403 |
|  |  | Mongol-Rural *vs.* Zhuang-Rural | 0.730 | 0.418 | 0.642 | 0.787 | 0.317 | 0.786 | 0.148 | 0.558 |
|  |  | Tibetan-Rural *vs.* Uyghur-Rural | 0.351 | 0.837 | 0.629 | 0.740 | 0.779 | 0.860 | 0.058 | 0.614 |
|  |  | Tibetan-Rural *vs.* Zhuang-Rural | 0.964 | 0.574 | 0.985 | 0.969 | 0.787 | 0.856 | 0.445 | 0.768 |
|  |  | Uyghur-Rural *vs.* Zhuang-Rural | 0.179 | 0.773 | 0.486 | 0.591 | 0.969 | 0.813 | 0.044 | 0.759 |
|  | (%) With significant differences | | 0 | 0 | 0 | 0 | 0 | 0 | 9.5% (2/21) | 0 |
| **Scheme-2B (Urban)** | | | | | | | | | | |
| **Diversity order** | **Taxon** | **Cohort** | **PL** | | **PLEC** | | | | | |
|  |  |  | ***z*** | **ln(*c*)** | ***z*** | ***d*** | **ln(*c*)** | ***A_max_*** | ***D_max_*** | ***LGD*** |
| *q*  = 0 | Phylum | Bai-Urban *vs.* Han-Urban | 0.798 | 0.929 | 0.913 | 0.754 | 0.778 | 0.244 | 0.777 | 0.679 |
|  |  | Bai-Urban *vs.* Kazakh-Urban | 0.987 | 0.982 | 0.942 | 0.907 | 0.978 | 0.419 | 0.867 | 0.926 |
|  |  | Bai-Urban *vs.* Mongol-Urban | 0.974 | 0.724 | 0.922 | 0.831 | 0.675 | 0.383 | 0.737 | 0.577 |
|  |  | Bai-Urban *vs.* Tibetan-Urban | 0.850 | 0.761 | 0.963 | 0.915 | 0.653 | 0.302 | 0.568 | 0.975 |
|  |  | Bai-Urban *vs.* Uyghur-Urban | 0.680 | 0.391 | 0.949 | 0.807 | 0.410 | 0.397 | 0.983 | 0.729 |
|  |  | Bai-Urban *vs.* Zhuang-Urban | 0.888 | 0.909 | 0.923 | 0.933 | 0.847 | 0.850 | 0.668 | 0.611 |
|  |  | Han-Urban *vs.* Kazakh-Urban | 0.783 | 0.918 | 0.872 | 0.639 | 0.751 | 0.307 | 0.748 | 0.543 |
|  |  | Han-Urban *vs.* Mongol-Urban | 0.800 | 0.675 | 0.968 | 0.844 | 0.920 | 0.393 | 0.779 | 0.916 |
|  |  | Han-Urban *vs.* Tibetan-Urban | 0.979 | 0.676 | 0.845 | 0.586 | 0.883 | 0.181 | 0.369 | 0.527 |
|  |  | Han-Urban *vs.* Uyghur-Urban | 0.834 | 0.420 | 0.967 | 0.570 | 0.295 | 0.177 | 0.732 | 0.329 |
|  |  | Han-Urban *vs.* Zhuang-Urban | 0.670 | 0.824 | 0.815 | 0.684 | 0.937 | 0.254 | 0.720 | 0.776 |
|  |  | Kazakh-Urban *vs.* Mongol-Urban | 0.951 | 0.776 | 0.849 | 0.675 | 0.675 | 0.709 | 0.623 | 0.527 |
|  |  | Kazakh-Urban *vs.* Tibetan-Urban | 0.835 | 0.779 | 0.987 | 0.977 | 0.642 | 0.190 | 0.608 | 0.935 |
|  |  | Kazakh-Urban *vs.* Uyghur-Urban | 0.693 | 0.394 | 0.915 | 0.916 | 0.451 | 0.213 | 0.826 | 0.672 |
|  |  | Kazakh-Urban *vs.* Zhuang-Urban | 0.910 | 0.926 | 0.990 | 0.799 | 0.837 | 0.764 | 0.574 | 0.590 |
|  |  | Mongol-Urban *vs.* Tibetan-Urban | 0.840 | 0.986 | 0.891 | 0.677 | 0.936 | 0.232 | 0.354 | 0.557 |
|  |  | Mongol-Urban *vs.* Uyghur-Urban | 0.685 | 0.254 | 0.987 | 0.662 | 0.250 | 0.230 | 0.793 | 0.363 |
|  |  | Mongol-Urban *vs.* Zhuang-Urban | 0.844 | 0.821 | 0.827 | 0.844 | 0.843 | 0.581 | 0.974 | 0.911 |
|  |  | Tibetan-Urban *vs.* Uyghur-Urban | 0.824 | 0.322 | 0.916 | 0.897 | 0.300 | 0.588 | 0.441 | 0.662 |
|  |  | Tibetan-Urban *vs.* Zhuang-Urban | 0.674 | 0.822 | 0.976 | 0.773 | 0.778 | 0.390 | 0.287 | 0.522 |
|  |  | Uyghur-Urban *vs.* Zhuang-Urban | 0.603 | 0.332 | 0.892 | 0.750 | 0.343 | 0.437 | 0.628 | 0.322 |
|  | (%) With significant differences | | 0 | 0 | 0 | 0 | 0 | 0 | 0 | 0 |
|  | Family | Bai-Urban *vs.* Han-Urban | 0.981 | 0.625 | 0.935 | 0.585 | 0.942 | 0.475 | 0.296 | 0.335 |
|  |  | Bai-Urban *vs.* Kazakh-Urban | 0.493 | 0.764 | 0.589 | 0.905 | 0.977 | 0.677 | 0.290 | 0.541 |
|  |  | Bai-Urban *vs.* Mongol-Urban | 0.868 | 0.488 | 0.971 | 0.669 | 0.364 | 0.407 | 0.741 | 0.480 |
|  |  | Bai-Urban *vs.* Tibetan-Urban | 0.989 | 0.622 | 0.999 | 0.912 | 0.797 | 0.183 | 0.270 | 0.704 |
|  |  | Bai-Urban *vs.* Uyghur-Urban | 0.916 | 0.312 | 0.990 | 0.972 | 0.435 | 0.958 | 0.468 | 0.997 |
|  |  | Bai-Urban *vs.* Zhuang-Urban | 0.857 | 0.953 | 0.953 | 0.689 | 0.710 | 0.181 | 0.258 | 0.407 |
|  |  | Han-Urban *vs.* Kazakh-Urban | 0.490 | 0.822 | 0.526 | 0.504 | 0.909 | 0.553 | 0.805 | 0.729 |
|  |  | Han-Urban *vs.* Mongol-Urban | 0.929 | 0.313 | 0.957 | 0.720 | 0.496 | 0.890 | 0.303 | 0.651 |
|  |  | Han-Urban *vs.* Tibetan-Urban | 0.966 | 0.972 | 0.946 | 0.642 | 0.860 | 0.974 | 0.699 | 0.514 |
|  |  | Han-Urban *vs.* Uyghur-Urban | 0.894 | 0.589 | 0.938 | 0.656 | 0.503 | 0.426 | 0.615 | 0.406 |
|  |  | Han-Urban *vs.* Zhuang-Urban | 0.879 | 0.568 | 0.884 | 0.713 | 0.670 | 0.544 | 0.673 | 0.817 |
|  |  | Kazakh-Urban *vs.* Mongol-Urban | 0.517 | 0.468 | 0.534 | 0.665 | 0.526 | 0.388 | 0.461 | 0.996 |
|  |  | Kazakh-Urban *vs.* Tibetan-Urban | 0.523 | 0.841 | 0.634 | 0.828 | 0.803 | 0.211 | 0.761 | 0.733 |
|  |  | Kazakh-Urban *vs.* Uyghur-Urban | 0.465 | 0.436 | 0.654 | 0.933 | 0.402 | 0.585 | 0.419 | 0.490 |
|  |  | Kazakh-Urban *vs.* Zhuang-Urban | 0.562 | 0.767 | 0.625 | 0.653 | 0.773 | 0.178 | 0.958 | 0.812 |
|  |  | Mongol-Urban *vs.* Tibetan-Urban | 0.901 | 0.324 | 0.973 | 0.817 | 0.331 | 0.712 | 0.499 | 0.690 |
|  |  | Mongol-Urban *vs.* Uyghur-Urban | 0.850 | 0.225 | 0.972 | 0.800 | 0.276 | 0.241 | 0.922 | 0.551 |
|  |  | Mongol-Urban *vs.* Zhuang-Urban | 0.955 | 0.582 | 0.892 | 0.988 | 0.674 | 0.410 | 0.373 | 0.849 |
|  |  | Tibetan-Urban *vs.* Uyghur-Urban | 0.898 | 0.526 | 0.981 | 0.895 | 0.564 | 0.150 | 0.580 | 0.732 |
|  |  | Tibetan-Urban *vs.* Zhuang-Urban | 0.868 | 0.604 | 0.930 | 0.797 | 0.547 | 0.378 | 0.676 | 0.576 |
|  |  | Uyghur-Urban *vs.* Zhuang-Urban | 0.814 | 0.332 | 0.973 | 0.747 | 0.327 | 0.136 | 0.612 | 0.452 |
|  | (%) With significant differences | | 0 | 0 | 0 | 0 | 0 | 0 | 0 | 0 |
|  | Genus | Bai-Urban *vs.* Han-Urban | 0.828 | 0.619 | 0.851 | 0.613 | 0.892 | 0.075 | 0.235 | 0.548 |
|  |  | Bai-Urban *vs.* Kazakh-Urban | 0.903 | 0.623 | 0.731 | 0.781 | 0.836 | 0.473 | 0.711 | 1.000 |
|  |  | Bai-Urban *vs.* Mongol-Urban | 0.830 | 0.542 | 0.768 | 0.700 | 0.533 | 0.752 | 0.969 | 0.790 |
|  |  | Bai-Urban *vs.* Tibetan-Urban | 0.871 | 0.781 | 0.827 | 0.861 | 0.821 | 0.764 | 0.788 | 0.950 |
|  |  | Bai-Urban *vs.* Uyghur-Urban | 0.880 | 0.316 | 0.965 | 0.862 | 0.367 | 0.396 | 0.760 | 0.685 |
|  |  | Bai-Urban *vs.* Zhuang-Urban | 0.690 | 0.935 | 0.872 | 0.819 | 0.904 | 0.045 | 0.538 | 0.939 |
|  |  | Han-Urban *vs.* Kazakh-Urban | 0.782 | 0.982 | 0.599 | 0.456 | 0.954 | 0.091 | 0.443 | 0.575 |
|  |  | Han-Urban *vs.* Mongol-Urban | 0.959 | 0.385 | 0.973 | 0.807 | 0.586 | 0.108 | 0.235 | 0.609 |
|  |  | Han-Urban *vs.* Tibetan-Urban | 0.722 | 0.864 | 0.670 | 0.466 | 0.932 | 0.065 | 0.242 | 0.461 |
|  |  | Han-Urban *vs.* Uyghur-Urban | 0.972 | 0.558 | 0.832 | 0.566 | 0.457 | 0.070 | 0.385 | 0.383 |
|  |  | Han-Urban *vs.* Zhuang-Urban | 0.855 | 0.665 | 0.951 | 0.634 | 0.804 | 0.527 | 0.320 | 0.444 |
|  |  | Kazakh-Urban *vs.* Mongol-Urban | 0.770 | 0.344 | 0.578 | 0.565 | 0.461 | 0.363 | 0.829 | 0.794 |
|  |  | Kazakh-Urban *vs.* Tibetan-Urban | 0.978 | 0.858 | 0.901 | 0.930 | 0.997 | 0.305 | 0.898 | 0.950 |
|  |  | Kazakh-Urban *vs.* Uyghur-Urban | 0.786 | 0.560 | 0.777 | 0.954 | 0.446 | 0.893 | 0.940 | 0.634 |
|  |  | Kazakh-Urban *vs.* Zhuang-Urban | 0.649 | 0.641 | 0.626 | 0.636 | 0.747 | 0.031 | 0.768 | 0.931 |
|  |  | Mongol-Urban *vs.* Tibetan-Urban | 0.732 | 0.424 | 0.641 | 0.592 | 0.453 | 0.811 | 0.803 | 0.761 |
|  |  | Mongol-Urban *vs.* Uyghur-Urban | 1.000 | 0.225 | 0.854 | 0.709 | 0.285 | 0.283 | 0.830 | 0.546 |
|  |  | Mongol-Urban *vs.* Zhuang-Urban | 0.821 | 0.464 | 0.923 | 0.819 | 0.627 | 0.065 | 0.372 | 0.786 |
|  |  | Tibetan-Urban *vs.* Uyghur-Urban | 0.782 | 0.445 | 0.876 | 0.969 | 0.500 | 0.274 | 0.926 | 0.696 |
|  |  | Tibetan-Urban *vs.* Zhuang-Urban | 0.623 | 0.849 | 0.701 | 0.663 | 0.759 | 0.030 | 0.604 | 0.891 |
|  |  | Uyghur-Urban *vs.* Zhuang-Urban | 0.906 | 0.362 | 0.892 | 0.755 | 0.351 | 0.028 | 0.776 | 0.664 |
|  | (%) With significant differences | | 0 | 0 | 0 | 0 | 0 | 0 | 19.0% (4/21) | 0 |
|  | Species | Bai-Urban *vs.* Han-Urban | 0.714 | 0.585 | 0.708 | 0.205 | 0.800 | 0.066 | 0.140 | 0.087 |
|  |  | Bai-Urban *vs.* Kazakh-Urban | 0.509 | 0.668 | 0.888 | 0.885 | 0.693 | 0.352 | 0.288 | 0.352 |
|  |  | Bai-Urban *vs.* Mongol-Urban | 0.599 | 0.633 | 0.658 | 0.252 | 0.658 | 0.095 | 0.137 | 0.101 |
|  |  | Bai-Urban *vs.* Tibetan-Urban | 0.805 | 0.531 | 0.475 | 0.475 | 0.395 | 0.270 | 0.925 | 0.731 |
|  |  | Bai-Urban *vs.* Uyghur-Urban | 0.644 | 0.366 | 0.938 | 0.748 | 0.394 | 0.318 | 0.193 | 0.440 |
|  |  | Bai-Urban *vs.* Zhuang-Urban | 0.551 | 0.947 | 0.771 | 0.292 | 0.955 | 0.058 | 0.170 | 0.134 |
|  |  | Han-Urban *vs.* Kazakh-Urban | 0.701 | 0.901 | 0.582 | 0.365 | 0.903 | 0.066 | 0.312 | 0.447 |
|  |  | Han-Urban *vs.* Mongol-Urban | 0.925 | 0.316 | 0.954 | 0.822 | 0.518 | 0.098 | 0.293 | 0.705 |
|  |  | Han-Urban *vs.* Tibetan-Urban | 0.499 | 0.981 | 0.538 | 0.975 | 0.622 | 0.058 | 0.108 | 0.018 |
|  |  | Han-Urban *vs.* Uyghur-Urban | 0.849 | 0.650 | 0.788 | 0.560 | 0.508 | 0.056 | 0.548 | 0.465 |
|  |  | Han-Urban *vs.* Zhuang-Urban | 0.856 | 0.530 | 0.923 | 0.692 | 0.759 | 0.150 | 0.348 | 0.654 |
|  |  | Kazakh-Urban *vs.* Mongol-Urban | 0.719 | 0.374 | 0.562 | 0.456 | 0.396 | 0.097 | 0.660 | 0.603 |
|  |  | Kazakh-Urban *vs.* Tibetan-Urban | 0.331 | 0.875 | 0.344 | 0.538 | 0.669 | 0.852 | 0.265 | 0.221 |
|  |  | Kazakh-Urban *vs.* Uyghur-Urban | 0.905 | 0.549 | 0.831 | 0.862 | 0.568 | 0.960 | 0.597 | 0.921 |
|  |  | Kazakh-Urban *vs.* Zhuang-Urban | 0.772 | 0.637 | 0.629 | 0.498 | 0.660 | 0.059 | 0.622 | 0.644 |
|  |  | Mongol-Urban *vs.* Tibetan-Urban | 0.487 | 0.312 | 0.607 | 0.879 | 0.254 | 0.071 | 0.154 | 0.068 |
|  |  | Mongol-Urban *vs.* Uyghur-Urban | 0.909 | 0.219 | 0.785 | 0.703 | 0.260 | 0.087 | 0.936 | 0.567 |
|  |  | Mongol-Urban *vs.* Zhuang-Urban | 0.910 | 0.598 | 0.851 | 0.870 | 0.651 | 0.206 | 0.809 | 0.929 |
|  |  | Tibetan-Urban *vs.* Uyghur-Urban | 0.356 | 0.602 | 0.420 | 0.642 | 0.774 | 0.876 | 0.217 | 0.234 |
|  |  | Tibetan-Urban *vs.* Zhuang-Urban | 0.372 | 0.452 | 0.504 | 0.836 | 0.355 | 0.050 | 0.161 | 0.048 |
|  |  | Uyghur-Urban *vs.* Zhuang-Urban | 0.956 | 0.338 | 0.843 | 0.704 | 0.378 | 0.046 | 0.841 | 0.524 |
|  | (%) With significant differences | | 0 | 0 | 0 | 0 | 0 | 9.5% (2/21) | 0 | 9.5% (2/21) |
| *q*  = 1 | Phylum | Bai-Urban *vs.* Han-Urban | 0.526 | 0.809 | 0.641 | 0.688 | 0.732 | 0.324 | 0.129 | 0.712 |
|  |  | Bai-Urban *vs.* Kazakh-Urban | 0.761 | 0.537 | 0.886 | 0.924 | 0.491 | 0.688 | 0.128 | 0.907 |
|  |  | Bai-Urban *vs.* Mongol-Urban | 0.585 | 0.703 | 0.690 | 0.736 | 0.814 | 0.659 | 0.845 | 0.701 |
|  |  | Bai-Urban *vs.* Tibetan-Urban | 0.907 | 0.819 | 0.915 | 0.862 | 0.870 | 0.988 | 0.800 | 0.870 |
|  |  | Bai-Urban *vs.* Uyghur-Urban | 0.745 | 0.372 | 0.853 | 0.904 | 0.463 | 0.897 | 0.431 | 0.825 |
|  |  | Bai-Urban *vs.* Zhuang-Urban | 0.915 | 0.903 | 0.878 | 0.966 | 0.924 | 0.477 | 0.814 | 0.866 |
|  |  | Han-Urban *vs.* Kazakh-Urban | 0.384 | 0.361 | 0.579 | 0.639 | 0.287 | 0.361 | 0.004 | 0.660 |
|  |  | Han-Urban *vs.* Mongol-Urban | 0.897 | 0.497 | 0.953 | 0.942 | 0.535 | 0.372 | 0.090 | 0.985 |
|  |  | Han-Urban *vs.* Tibetan-Urban | 0.648 | 0.634 | 0.582 | 0.498 | 0.619 | 0.349 | 0.080 | 0.833 |
|  |  | Han-Urban *vs.* Uyghur-Urban | 0.810 | 0.323 | 0.856 | 0.869 | 0.362 | 0.327 | 0.023 | 0.875 |
|  |  | Han-Urban *vs.* Zhuang-Urban | 0.444 | 0.924 | 0.465 | 0.440 | 0.828 | 0.489 | 0.102 | 0.618 |
|  |  | Kazakh-Urban *vs.* Mongol-Urban | 0.428 | 0.700 | 0.641 | 0.723 | 0.588 | 0.874 | 0.043 | 0.678 |
|  |  | Kazakh-Urban *vs.* Tibetan-Urban | 0.710 | 0.684 | 0.976 | 0.972 | 0.623 | 0.702 | 0.312 | 0.826 |
|  |  | Kazakh-Urban *vs.* Uyghur-Urban | 0.501 | 0.980 | 0.733 | 0.827 | 0.817 | 0.710 | 0.243 | 0.741 |
|  |  | Kazakh-Urban *vs.* Zhuang-Urban | 0.853 | 0.524 | 0.994 | 0.953 | 0.492 | 0.558 | 0.342 | 0.974 |
|  |  | Mongol-Urban *vs.* Tibetan-Urban | 0.760 | 0.943 | 0.621 | 0.591 | 0.984 | 0.654 | 0.619 | 0.863 |
|  |  | Mongol-Urban *vs.* Uyghur-Urban | 0.873 | 0.618 | 0.866 | 0.864 | 0.635 | 0.669 | 0.225 | 0.867 |
|  |  | Mongol-Urban *vs.* Zhuang-Urban | 0.609 | 0.680 | 0.580 | 0.670 | 0.752 | 0.733 | 0.667 | 0.647 |
|  |  | Tibetan-Urban *vs.* Uyghur-Urban | 0.887 | 0.623 | 0.803 | 0.815 | 0.663 | 0.951 | 0.790 | 0.969 |
|  |  | Tibetan-Urban *vs.* Zhuang-Urban | 0.839 | 0.750 | 0.970 | 0.899 | 0.792 | 0.456 | 0.970 | 0.776 |
|  |  | Uyghur-Urban *vs.* Zhuang-Urban | 0.761 | 0.455 | 0.754 | 0.879 | 0.537 | 0.458 | 0.851 | 0.751 |
|  | (%) With significant differences | | 0 | 0 | 0 | 0 | 0 | 0 | 14.3% (3/21) | 0 |
|  | Family | Bai-Urban *vs.* Han-Urban | 0.549 | 0.844 | 0.624 | 0.573 | 0.961 | 0.219 | 0.875 | 0.772 |
|  |  | Bai-Urban *vs.* Kazakh-Urban | 0.601 | 0.743 | 0.606 | 0.692 | 0.880 | 0.994 | 0.152 | 0.756 |
|  |  | Bai-Urban *vs.* Mongol-Urban | 0.835 | 0.869 | 0.906 | 0.833 | 0.857 | 0.477 | 0.902 | 0.939 |
|  |  | Bai-Urban *vs.* Tibetan-Urban | 0.660 | 0.766 | 0.875 | 0.990 | 0.885 | 0.604 | 0.778 | 0.886 |
|  |  | Bai-Urban *vs.* Uyghur-Urban | 0.944 | 0.282 | 0.973 | 0.885 | 0.342 | 0.942 | 0.054 | 0.950 |
|  |  | Bai-Urban *vs.* Zhuang-Urban | 0.871 | 0.971 | 0.969 | 0.853 | 0.971 | 0.604 | 0.696 | 0.815 |
|  |  | Han-Urban *vs.* Kazakh-Urban | 0.273 | 0.843 | 0.252 | 0.315 | 0.913 | 0.241 | 0.061 | 0.550 |
|  |  | Han-Urban *vs.* Mongol-Urban | 0.612 | 0.723 | 0.655 | 0.553 | 0.837 | 0.360 | 0.985 | 0.832 |
|  |  | Han-Urban *vs.* Tibetan-Urban | 0.306 | 0.645 | 0.515 | 0.544 | 0.865 | 0.347 | 0.558 | 0.690 |
|  |  | Han-Urban *vs.* Uyghur-Urban | 0.647 | 0.424 | 0.655 | 0.596 | 0.442 | 0.210 | 0.055 | 0.851 |
|  |  | Han-Urban *vs.* Zhuang-Urban | 0.417 | 0.901 | 0.521 | 0.572 | 0.994 | 0.383 | 0.341 | 0.643 |
|  |  | Kazakh-Urban *vs.* Mongol-Urban | 0.488 | 0.658 | 0.481 | 0.521 | 0.784 | 0.439 | 0.120 | 0.690 |
|  |  | Kazakh-Urban *vs.* Tibetan-Urban | 0.917 | 0.620 | 0.750 | 0.748 | 0.802 | 0.596 | 0.334 | 0.863 |
|  |  | Kazakh-Urban *vs.* Uyghur-Urban | 0.465 | 0.570 | 0.540 | 0.744 | 0.491 | 0.919 | 0.633 | 0.669 |
|  |  | Kazakh-Urban *vs.* Zhuang-Urban | 0.669 | 0.775 | 0.582 | 0.527 | 0.890 | 0.526 | 0.469 | 0.863 |
|  |  | Mongol-Urban *vs.* Tibetan-Urban | 0.520 | 0.849 | 0.803 | 0.845 | 0.989 | 0.687 | 0.611 | 0.820 |
|  |  | Mongol-Urban *vs.* Uyghur-Urban | 0.953 | 0.322 | 0.884 | 0.722 | 0.356 | 0.390 | 0.110 | 0.995 |
|  |  | Mongol-Urban *vs.* Zhuang-Urban | 0.740 | 0.855 | 0.864 | 0.986 | 0.830 | 0.903 | 0.573 | 0.789 |
|  |  | Tibetan-Urban *vs.* Uyghur-Urban | 0.629 | 0.314 | 0.906 | 0.926 | 0.417 | 0.524 | 0.431 | 0.834 |
|  |  | Tibetan-Urban *vs.* Zhuang-Urban | 0.731 | 0.757 | 0.893 | 0.834 | 0.848 | 0.767 | 0.798 | 0.978 |
|  |  | Uyghur-Urban *vs.* Zhuang-Urban | 0.811 | 0.372 | 0.999 | 0.767 | 0.409 | 0.490 | 0.683 | 0.768 |
|  | (%) With significant differences | | 0 | 0 | 0 | 0 | 0 | 0 | 0 | 0 |
|  | Genus | Bai-Urban *vs.* Han-Urban | 0.604 | 0.602 | 0.884 | 0.760 | 0.911 | 0.161 | 0.215 | 0.976 |
|  |  | Bai-Urban *vs.* Kazakh-Urban | 0.562 | 0.741 | 0.506 | 0.595 | 0.934 | 0.082 | 0.093 | 0.528 |
|  |  | Bai-Urban *vs.* Mongol-Urban | 0.902 | 0.965 | 0.908 | 0.993 | 0.842 | 0.098 | 0.797 | 0.842 |
|  |  | Bai-Urban *vs.* Tibetan-Urban | 0.599 | 0.608 | 0.660 | 0.730 | 0.653 | 0.044 | 0.772 | 0.752 |
|  |  | Bai-Urban *vs.* Uyghur-Urban | 0.998 | 0.395 | 0.803 | 0.685 | 0.477 | 0.056 | 0.077 | 0.947 |
|  |  | Bai-Urban *vs.* Zhuang-Urban | 0.896 | 0.548 | 0.764 | 0.972 | 0.844 | 0.147 | 0.113 | 0.561 |
|  |  | Han-Urban *vs.* Kazakh-Urban | 0.325 | 0.917 | 0.365 | 0.376 | 0.994 | 0.288 | 0.122 | 0.521 |
|  |  | Han-Urban *vs.* Mongol-Urban | 0.647 | 0.602 | 0.776 | 0.587 | 0.785 | 0.364 | 0.340 | 0.859 |
|  |  | Han-Urban *vs.* Tibetan-Urban | 0.230 | 0.277 | 0.398 | 0.389 | 0.510 | 0.371 | 0.716 | 0.624 |
|  |  | Han-Urban *vs.* Uyghur-Urban | 0.659 | 0.688 | 0.684 | 0.524 | 0.549 | 0.240 | 0.213 | 0.962 |
|  |  | Han-Urban *vs.* Zhuang-Urban | 0.433 | 0.914 | 0.588 | 0.545 | 0.969 | 0.556 | 0.172 | 0.568 |
|  |  | Kazakh-Urban *vs.* Mongol-Urban | 0.477 | 0.733 | 0.499 | 0.532 | 0.809 | 0.630 | 0.113 | 0.642 |
|  |  | Kazakh-Urban *vs.* Tibetan-Urban | 0.924 | 0.480 | 0.948 | 0.951 | 0.610 | 0.646 | 0.237 | 0.907 |
|  |  | Kazakh-Urban *vs.* Uyghur-Urban | 0.529 | 0.592 | 0.646 | 0.872 | 0.506 | 0.771 | 0.521 | 0.533 |
|  |  | Kazakh-Urban *vs.* Zhuang-Urban | 0.557 | 0.975 | 0.575 | 0.498 | 0.975 | 0.393 | 0.638 | 0.839 |
|  |  | Mongol-Urban *vs.* Tibetan-Urban | 0.459 | 0.612 | 0.609 | 0.606 | 0.747 | 0.829 | 0.896 | 0.793 |
|  |  | Mongol-Urban *vs.* Uyghur-Urban | 0.938 | 0.425 | 0.872 | 0.694 | 0.416 | 0.481 | 0.131 | 0.921 |
|  |  | Mongol-Urban *vs.* Zhuang-Urban | 0.751 | 0.567 | 0.809 | 0.951 | 0.651 | 0.702 | 0.118 | 0.696 |
|  |  | Tibetan-Urban *vs.* Uyghur-Urban | 0.582 | 0.312 | 0.787 | 0.967 | 0.383 | 0.497 | 0.353 | 0.766 |
|  |  | Tibetan-Urban *vs.* Zhuang-Urban | 0.515 | 0.342 | 0.667 | 0.610 | 0.477 | 0.544 | 0.312 | 0.987 |
|  |  | Uyghur-Urban *vs.* Zhuang-Urban | 0.898 | 0.561 | 0.987 | 0.676 | 0.473 | 0.379 | 0.977 | 0.598 |
|  | (%) With significant differences | | 0 | 0 | 0 | 0 | 0 | 4.8% (1/21) | 0 | 0 |
|  | Species | Bai-Urban *vs.* Han-Urban | 0.512 | 0.422 | 0.578 | 0.479 | 0.551 | 0.428 | 0.202 | 0.856 |
|  |  | Bai-Urban *vs.* Kazakh-Urban | 0.567 | 0.834 | 0.618 | 0.746 | 0.921 | 0.785 | 0.138 | 0.604 |
|  |  | Bai-Urban *vs.* Mongol-Urban | 0.878 | 0.711 | 0.809 | 0.624 | 0.822 | 0.578 | 0.281 | 0.825 |
|  |  | Bai-Urban *vs.* Tibetan-Urban | 0.915 | 0.590 | 0.805 | 0.806 | 0.535 | 0.407 | 0.941 | 0.777 |
|  |  | Bai-Urban *vs.* Uyghur-Urban | 0.971 | 0.350 | 0.934 | 0.875 | 0.415 | 0.729 | 0.095 | 0.975 |
|  |  | Bai-Urban *vs.* Zhuang-Urban | 0.876 | 0.537 | 0.979 | 0.727 | 0.673 | 0.255 | 0.102 | 0.582 |
|  |  | Han-Urban *vs.* Kazakh-Urban | 0.236 | 0.615 | 0.266 | 0.293 | 0.688 | 0.492 | 0.156 | 0.463 |
|  |  | Han-Urban *vs.* Mongol-Urban | 0.533 | 0.595 | 0.661 | 0.655 | 0.702 | 0.560 | 0.639 | 0.659 |
|  |  | Han-Urban *vs.* Tibetan-Urban | 0.551 | 0.622 | 0.749 | 0.699 | 0.796 | 0.292 | 0.292 | 0.882 |
|  |  | Han-Urban *vs.* Uyghur-Urban | 0.551 | 0.874 | 0.554 | 0.454 | 0.779 | 0.331 | 0.336 | 0.832 |
|  |  | Han-Urban *vs.* Zhuang-Urban | 0.362 | 0.698 | 0.471 | 0.496 | 0.759 | 0.812 | 0.251 | 0.482 |
|  |  | Kazakh-Urban *vs.* Mongol-Urban | 0.435 | 0.953 | 0.410 | 0.409 | 0.921 | 0.733 | 0.150 | 0.663 |
|  |  | Kazakh-Urban *vs.* Tibetan-Urban | 0.463 | 0.903 | 0.469 | 0.587 | 0.767 | 0.397 | 0.235 | 0.474 |
|  |  | Kazakh-Urban *vs.* Uyghur-Urban | 0.576 | 0.551 | 0.661 | 0.827 | 0.536 | 0.585 | 0.550 | 0.658 |
|  |  | Kazakh-Urban *vs.* Zhuang-Urban | 0.525 | 0.834 | 0.489 | 0.439 | 0.820 | 0.260 | 0.573 | 0.834 |
|  |  | Mongol-Urban *vs.* Tibetan-Urban | 0.990 | 0.946 | 0.954 | 0.920 | 0.823 | 0.395 | 0.538 | 0.552 |
|  |  | Mongol-Urban *vs.* Uyghur-Urban | 0.923 | 0.507 | 0.787 | 0.584 | 0.533 | 0.474 | 0.187 | 0.851 |
|  |  | Mongol-Urban *vs.* Zhuang-Urban | 0.687 | 0.859 | 0.704 | 0.801 | 0.897 | 0.360 | 0.144 | 0.747 |
|  |  | Tibetan-Urban *vs.* Uyghur-Urban | 0.911 | 0.440 | 0.742 | 0.699 | 0.510 | 0.575 | 0.187 | 0.709 |
|  |  | Tibetan-Urban *vs.* Zhuang-Urban | 0.710 | 0.924 | 0.707 | 0.952 | 0.888 | 0.180 | 0.100 | 0.324 |
|  |  | Uyghur-Urban *vs.* Zhuang-Urban | 0.875 | 0.526 | 0.951 | 0.643 | 0.509 | 0.185 | 0.982 | 0.599 |
|  | (%) With significant differences | | 0 | 0 | 0 | 0 | 0 | 0 | 0 | 0 |
| *q* = 2 | Phylum | Bai-Urban *vs.* Han-Urban | 0.656 | 0.571 | 0.708 | 0.795 | 0.579 | 0.496 | 0.043 | 0.751 |
|  |  | Bai-Urban *vs.* Kazakh-Urban | 0.946 | 0.594 | 0.980 | 0.967 | 0.584 | 0.585 | 0.313 | 0.967 |
|  |  | Bai-Urban *vs.* Mongol-Urban | 0.661 | 0.872 | 0.752 | 0.821 | 0.902 | 0.790 | 0.635 | 0.739 |
|  |  | Bai-Urban *vs.* Tibetan-Urban | 0.938 | 0.854 | 0.961 | 0.912 | 0.825 | 0.167 | 0.772 | 0.762 |
|  |  | Bai-Urban *vs.* Uyghur-Urban | 0.751 | 0.684 | 0.765 | 0.836 | 0.689 | 0.532 | 0.958 | 0.736 |
|  |  | Bai-Urban *vs.* Zhuang-Urban | 0.905 | 0.830 | 0.979 | 0.978 | 0.879 | 0.918 | 0.810 | 0.887 |
|  |  | Han-Urban *vs.* Kazakh-Urban | 0.632 | 0.222 | 0.698 | 0.789 | 0.253 | 0.404 | 0.008 | 0.820 |
|  |  | Han-Urban *vs.* Mongol-Urban | 0.973 | 0.393 | 0.974 | 0.965 | 0.469 | 0.551 | 0.023 | 0.938 |
|  |  | Han-Urban *vs.* Tibetan-Urban | 0.750 | 0.447 | 0.682 | 0.596 | 0.450 | 0.852 | 0.029 | 0.953 |
|  |  | Han-Urban *vs.* Uyghur-Urban | 0.976 | 0.296 | 0.945 | 0.926 | 0.346 | 0.407 | 0.012 | 0.945 |
|  |  | Han-Urban *vs.* Zhuang-Urban | 0.749 | 0.763 | 0.708 | 0.606 | 0.720 | 0.480 | 0.129 | 0.894 |
|  |  | Kazakh-Urban *vs.* Mongol-Urban | 0.670 | 0.599 | 0.765 | 0.797 | 0.582 | 0.548 | 0.079 | 0.789 |
|  |  | Kazakh-Urban *vs.* Tibetan-Urban | 0.896 | 0.725 | 0.982 | 0.955 | 0.748 | 0.122 | 0.587 | 0.842 |
|  |  | Kazakh-Urban *vs.* Uyghur-Urban | 0.734 | 0.777 | 0.770 | 0.817 | 0.770 | 0.954 | 0.244 | 0.811 |
|  |  | Kazakh-Urban *vs.* Zhuang-Urban | 0.875 | 0.472 | 0.970 | 0.983 | 0.525 | 0.740 | 0.218 | 0.947 |
|  |  | Mongol-Urban *vs.* Tibetan-Urban | 0.782 | 0.932 | 0.681 | 0.649 | 0.885 | 0.237 | 0.447 | 0.980 |
|  |  | Mongol-Urban *vs.* Uyghur-Urban | 0.953 | 0.725 | 0.972 | 0.933 | 0.722 | 0.450 | 0.442 | 0.974 |
|  |  | Mongol-Urban *vs.* Zhuang-Urban | 0.795 | 0.688 | 0.742 | 0.725 | 0.746 | 0.790 | 0.978 | 0.877 |
|  |  | Tibetan-Urban *vs.* Uyghur-Urban | 0.843 | 0.889 | 0.743 | 0.749 | 0.905 | 0.138 | 0.771 | 0.999 |
|  |  | Tibetan-Urban *vs.* Zhuang-Urban | 0.981 | 0.665 | 0.930 | 0.898 | 0.683 | 0.220 | 0.593 | 0.859 |
|  |  | Uyghur-Urban *vs.* Zhuang-Urban | 0.863 | 0.526 | 0.789 | 0.805 | 0.578 | 0.701 | 0.673 | 0.874 |
|  | (%) With significant differences | | 0 | 0 | 0 | 0 | 0 | 0 | 23.8% (5/21) | 0 |
|  | Family | Bai-Urban *vs.* Han-Urban | 0.447 | 0.983 | 0.465 | 0.496 | 0.937 | 0.567 | 0.337 | 0.557 |
|  |  | Bai-Urban *vs.* Kazakh-Urban | 0.666 | 0.882 | 0.752 | 0.851 | 0.941 | 0.921 | 0.296 | 0.898 |
|  |  | Bai-Urban *vs.* Mongol-Urban | 0.601 | 0.976 | 0.610 | 0.673 | 0.829 | 0.126 | 0.590 | 0.615 |
|  |  | Bai-Urban *vs.* Tibetan-Urban | 0.743 | 0.764 | 0.895 | 0.955 | 0.889 | 0.788 | 0.935 | 0.993 |
|  |  | Bai-Urban *vs.* Uyghur-Urban | 0.867 | 0.419 | 0.891 | 0.969 | 0.411 | 0.070 | 0.149 | 0.793 |
|  |  | Bai-Urban *vs.* Zhuang-Urban | 0.903 | 0.901 | 0.771 | 0.751 | 0.869 | 0.135 | 0.878 | 0.850 |
|  |  | Han-Urban *vs.* Kazakh-Urban | 0.255 | 0.885 | 0.281 | 0.403 | 0.990 | 0.532 | 0.061 | 0.528 |
|  |  | Han-Urban *vs.* Mongol-Urban | 0.757 | 0.999 | 0.785 | 0.641 | 0.915 | 0.322 | 0.695 | 0.959 |
|  |  | Han-Urban *vs.* Tibetan-Urban | 0.298 | 0.731 | 0.379 | 0.396 | 0.841 | 0.561 | 0.313 | 0.622 |
|  |  | Han-Urban *vs.* Uyghur-Urban | 0.610 | 0.481 | 0.609 | 0.582 | 0.501 | 0.269 | 0.069 | 0.760 |
|  |  | Han-Urban *vs.* Zhuang-Urban | 0.499 | 0.895 | 0.639 | 0.634 | 0.961 | 0.268 | 0.279 | 0.745 |
|  |  | Kazakh-Urban *vs.* Mongol-Urban | 0.380 | 0.870 | 0.469 | 0.576 | 0.921 | 0.120 | 0.150 | 0.535 |
|  |  | Kazakh-Urban *vs.* Tibetan-Urban | 0.854 | 0.719 | 0.844 | 0.903 | 0.842 | 0.873 | 0.358 | 0.906 |
|  |  | Kazakh-Urban *vs.* Uyghur-Urban | 0.507 | 0.627 | 0.642 | 0.870 | 0.523 | 0.094 | 0.695 | 0.722 |
|  |  | Kazakh-Urban *vs.* Zhuang-Urban | 0.546 | 0.814 | 0.564 | 0.610 | 0.978 | 0.092 | 0.510 | 0.745 |
|  |  | Mongol-Urban *vs.* Tibetan-Urban | 0.435 | 0.729 | 0.582 | 0.631 | 0.739 | 0.150 | 0.588 | 0.664 |
|  |  | Mongol-Urban *vs.* Uyghur-Urban | 0.773 | 0.434 | 0.745 | 0.651 | 0.494 | 0.742 | 0.131 | 0.787 |
|  |  | Mongol-Urban *vs.* Zhuang-Urban | 0.706 | 0.910 | 0.858 | 0.983 | 0.954 | 0.526 | 0.638 | 0.731 |
|  |  | Tibetan-Urban *vs.* Uyghur-Urban | 0.643 | 0.368 | 0.806 | 0.979 | 0.418 | 0.086 | 0.418 | 0.818 |
|  |  | Tibetan-Urban *vs.* Zhuang-Urban | 0.657 | 0.854 | 0.670 | 0.678 | 0.775 | 0.121 | 0.897 | 0.835 |
|  |  | Uyghur-Urban *vs.* Zhuang-Urban | 0.953 | 0.395 | 0.862 | 0.724 | 0.492 | 0.710 | 0.583 | 0.939 |
|  | (%) With significant differences | | 0 | 0 | 0 | 0 | 0 | 0 | 0 | 0 |
|  | Genus | Bai-Urban *vs.* Han-Urban | 0.478 | 0.542 | 0.814 | 0.756 | 0.879 | 0.415 | 0.257 | 0.988 |
|  |  | Bai-Urban *vs.* Kazakh-Urban | 0.657 | 0.694 | 0.564 | 0.607 | 0.899 | 0.631 | 0.093 | 0.574 |
|  |  | Bai-Urban *vs.* Mongol-Urban | 0.701 | 0.787 | 0.977 | 0.974 | 0.927 | 0.514 | 0.606 | 0.966 |
|  |  | Bai-Urban *vs.* Tibetan-Urban | 0.674 | 0.581 | 0.749 | 0.765 | 0.687 | 0.941 | 0.946 | 0.841 |
|  |  | Bai-Urban *vs.* Uyghur-Urban | 0.891 | 0.409 | 0.847 | 0.678 | 0.486 | 0.671 | 0.143 | 0.997 |
|  |  | Bai-Urban *vs.* Zhuang-Urban | 0.821 | 0.512 | 0.904 | 0.991 | 0.797 | 0.333 | 0.223 | 0.712 |
|  |  | Han-Urban *vs.* Kazakh-Urban | 0.277 | 0.889 | 0.320 | 0.352 | 0.967 | 0.590 | 0.094 | 0.498 |
|  |  | Han-Urban *vs.* Mongol-Urban | 0.655 | 0.709 | 0.831 | 0.612 | 0.943 | 0.685 | 0.570 | 0.938 |
|  |  | Han-Urban *vs.* Tibetan-Urban | 0.184 | 0.184 | 0.448 | 0.400 | 0.492 | 0.468 | 0.483 | 0.777 |
|  |  | Han-Urban *vs.* Uyghur-Urban | 0.595 | 0.768 | 0.628 | 0.473 | 0.565 | 0.560 | 0.246 | 0.984 |
|  |  | Han-Urban *vs.* Zhuang-Urban | 0.490 | 0.900 | 0.661 | 0.563 | 0.966 | 0.947 | 0.356 | 0.707 |
|  |  | Kazakh-Urban *vs.* Mongol-Urban | 0.396 | 0.878 | 0.415 | 0.478 | 0.971 | 0.701 | 0.089 | 0.539 |
|  |  | Kazakh-Urban *vs.* Tibetan-Urban | 0.912 | 0.438 | 0.888 | 0.900 | 0.624 | 0.631 | 0.184 | 0.777 |
|  |  | Kazakh-Urban *vs.* Uyghur-Urban | 0.502 | 0.664 | 0.616 | 0.859 | 0.553 | 0.800 | 0.355 | 0.520 |
|  |  | Kazakh-Urban *vs.* Zhuang-Urban | 0.453 | 0.969 | 0.486 | 0.462 | 0.944 | 0.415 | 0.288 | 0.684 |
|  |  | Mongol-Urban *vs.* Tibetan-Urban | 0.390 | 0.385 | 0.627 | 0.611 | 0.598 | 0.484 | 0.763 | 0.853 |
|  |  | Mongol-Urban *vs.* Uyghur-Urban | 0.845 | 0.551 | 0.754 | 0.606 | 0.502 | 0.578 | 0.203 | 0.965 |
|  |  | Mongol-Urban *vs.* Zhuang-Urban | 0.813 | 0.777 | 0.806 | 0.932 | 0.882 | 0.611 | 0.328 | 0.764 |
|  |  | Tibetan-Urban *vs.* Uyghur-Urban | 0.552 | 0.289 | 0.831 | 0.999 | 0.393 | 0.698 | 0.335 | 0.836 |
|  |  | Tibetan-Urban *vs.* Zhuang-Urban | 0.430 | 0.279 | 0.686 | 0.634 | 0.498 | 0.323 | 0.356 | 0.990 |
|  |  | Uyghur-Urban *vs.* Zhuang-Urban | 0.977 | 0.634 | 0.900 | 0.611 | 0.504 | 0.365 | 0.869 | 0.715 |
|  | (%) With significant differences | | 0 | 0 | 0 | 0 | 0 | 0 | 0 | 0 |
|  | Species | Bai-Urban *vs.* Han-Urban | 0.445 | 0.412 | 0.620 | 0.583 | 0.578 | 0.525 | 0.379 | 0.775 |
|  |  | Bai-Urban *vs.* Kazakh-Urban | 0.731 | 0.614 | 0.749 | 0.853 | 0.721 | 0.995 | 0.206 | 0.807 |
|  |  | Bai-Urban *vs.* Mongol-Urban | 0.622 | 0.439 | 0.753 | 0.669 | 0.606 | 0.159 | 0.209 | 0.996 |
|  |  | Bai-Urban *vs.* Tibetan-Urban | 0.871 | 0.556 | 0.790 | 0.761 | 0.526 | 0.956 | 0.803 | 0.784 |
|  |  | Bai-Urban *vs.* Uyghur-Urban | 0.827 | 0.289 | 0.973 | 0.904 | 0.414 | 0.857 | 0.147 | 0.932 |
|  |  | Bai-Urban *vs.* Zhuang-Urban | 0.891 | 0.396 | 0.936 | 0.847 | 0.556 | 0.544 | 0.143 | 0.782 |
|  |  | Han-Urban *vs.* Kazakh-Urban | 0.266 | 0.784 | 0.399 | 0.486 | 0.848 | 0.490 | 0.271 | 0.595 |
|  |  | Han-Urban *vs.* Mongol-Urban | 0.642 | 0.775 | 0.743 | 0.725 | 0.852 | 0.326 | 0.934 | 0.724 |
|  |  | Han-Urban *vs.* Tibetan-Urban | 0.492 | 0.620 | 0.855 | 0.913 | 0.798 | 0.487 | 0.635 | 0.963 |
|  |  | Han-Urban *vs.* Uyghur-Urban | 0.564 | 0.903 | 0.622 | 0.560 | 0.838 | 0.424 | 0.464 | 0.833 |
|  |  | Han-Urban *vs.* Zhuang-Urban | 0.452 | 0.843 | 0.493 | 0.483 | 0.852 | 0.770 | 0.260 | 0.558 |
|  |  | Kazakh-Urban *vs.* Mongol-Urban | 0.425 | 0.955 | 0.497 | 0.554 | 0.961 | 0.158 | 0.264 | 0.745 |
|  |  | Kazakh-Urban *vs.* Tibetan-Urban | 0.615 | 0.915 | 0.569 | 0.653 | 0.997 | 0.968 | 0.379 | 0.622 |
|  |  | Kazakh-Urban *vs.* Uyghur-Urban | 0.624 | 0.712 | 0.767 | 0.924 | 0.706 | 0.842 | 0.518 | 0.761 |
|  |  | Kazakh-Urban *vs.* Zhuang-Urban | 0.594 | 0.892 | 0.721 | 0.698 | 0.974 | 0.539 | 0.858 | 0.954 |
|  |  | Mongol-Urban *vs.* Tibetan-Urban | 0.761 | 0.799 | 0.942 | 0.895 | 0.954 | 0.167 | 0.562 | 0.717 |
|  |  | Mongol-Urban *vs.* Uyghur-Urban | 0.827 | 0.641 | 0.764 | 0.624 | 0.678 | 0.133 | 0.350 | 0.922 |
|  |  | Mongol-Urban *vs.* Zhuang-Urban | 0.661 | 0.924 | 0.634 | 0.738 | 0.996 | 0.248 | 0.274 | 0.764 |
|  |  | Tibetan-Urban *vs.* Uyghur-Urban | 0.958 | 0.465 | 0.746 | 0.669 | 0.570 | 0.899 | 0.344 | 0.844 |
|  |  | Tibetan-Urban *vs.* Zhuang-Urban | 0.937 | 0.700 | 0.616 | 0.729 | 0.942 | 0.497 | 0.224 | 0.508 |
|  |  | Uyghur-Urban *vs.* Zhuang-Urban | 0.889 | 0.682 | 0.969 | 0.767 | 0.621 | 0.437 | 0.679 | 0.668 |
|  | (%) With significant differences | | 0 | 0 | 0 | 0 | 0 | 0 | 0 | 0 |
| *q*  = 3 | Phylum | Bai-Urban *vs.* Han-Urban | 0.759 | 0.439 | 0.811 | 0.848 | 0.444 | 0.559 | 0.029 | 0.920 |
|  |  | Bai-Urban *vs.* Kazakh-Urban | 0.945 | 0.592 | 0.997 | 0.988 | 0.715 | 0.379 | 0.466 | 0.993 |
|  |  | Bai-Urban *vs.* Mongol-Urban | 0.736 | 0.970 | 0.823 | 0.843 | 0.960 | 0.811 | 0.541 | 0.875 |
|  |  | Bai-Urban *vs.* Tibetan-Urban | 0.924 | 0.825 | 0.931 | 0.882 | 0.835 | 0.424 | 0.749 | 0.952 |
|  |  | Bai-Urban *vs.* Uyghur-Urban | 0.775 | 0.796 | 0.806 | 0.819 | 0.858 | 0.287 | 0.862 | 0.842 |
|  |  | Bai-Urban *vs.* Zhuang-Urban | 0.931 | 0.751 | 0.988 | 0.997 | 0.714 | 0.602 | 0.617 | 0.993 |
|  |  | Han-Urban *vs.* Kazakh-Urban | 0.880 | 0.147 | 0.822 | 0.891 | 0.250 | 0.356 | 0.010 | 0.916 |
|  |  | Han-Urban *vs.* Mongol-Urban | 0.923 | 0.364 | 0.941 | 0.909 | 0.457 | 0.493 | 0.019 | 0.897 |
|  |  | Han-Urban *vs.* Tibetan-Urban | 0.889 | 0.340 | 0.724 | 0.591 | 0.361 | 0.437 | 0.023 | 0.997 |
|  |  | Han-Urban *vs.* Uyghur-Urban | 0.920 | 0.266 | 0.872 | 0.851 | 0.337 | 0.337 | 0.018 | 0.902 |
|  |  | Han-Urban *vs.* Zhuang-Urban | 0.823 | 0.698 | 0.756 | 0.719 | 0.756 | 0.433 | 0.170 | 0.902 |
|  |  | Kazakh-Urban *vs.* Mongol-Urban | 0.867 | 0.516 | 0.857 | 0.885 | 0.609 | 0.579 | 0.100 | 0.858 |
|  |  | Kazakh-Urban *vs.* Tibetan-Urban | 0.987 | 0.770 | 0.928 | 0.879 | 0.851 | 0.778 | 0.812 | 0.960 |
|  |  | Kazakh-Urban *vs.* Uyghur-Urban | 0.879 | 0.657 | 0.814 | 0.859 | 0.771 | 0.815 | 0.268 | 0.869 |
|  |  | Kazakh-Urban *vs.* Zhuang-Urban | 0.998 | 0.404 | 0.994 | 0.977 | 0.508 | 0.751 | 0.211 | 0.994 |
|  |  | Mongol-Urban *vs.* Tibetan-Urban | 0.871 | 0.802 | 0.715 | 0.627 | 0.780 | 0.670 | 0.348 | 0.944 |
|  |  | Mongol-Urban *vs.* Uyghur-Urban | 0.972 | 0.755 | 0.925 | 0.890 | 0.757 | 0.417 | 0.477 | 0.956 |
|  |  | Mongol-Urban *vs.* Zhuang-Urban | 0.811 | 0.682 | 0.777 | 0.785 | 0.702 | 0.798 | 0.873 | 0.845 |
|  |  | Tibetan-Urban *vs.* Uyghur-Urban | 0.868 | 0.960 | 0.758 | 0.711 | 0.948 | 0.615 | 0.649 | 0.927 |
|  |  | Tibetan-Urban *vs.* Zhuang-Urban | 0.976 | 0.586 | 0.939 | 0.850 | 0.561 | 0.908 | 0.418 | 0.937 |
|  |  | Uyghur-Urban *vs.* Zhuang-Urban | 0.838 | 0.523 | 0.780 | 0.787 | 0.549 | 0.622 | 0.600 | 0.864 |
|  | (%) With significant differences | | 0 | 0 | 0 | 0 | 0 | 0 | 23.8% (5/21) | 0 |
|  | Family | Bai-Urban *vs.* Han-Urban | 0.406 | 0.990 | 0.449 | 0.513 | 0.949 | 0.041 | 0.271 | 0.529 |
|  |  | Bai-Urban *vs.* Kazakh-Urban | 0.670 | 0.978 | 0.857 | 0.961 | 0.965 | 0.479 | 0.064 | 0.983 |
|  |  | Bai-Urban *vs.* Mongol-Urban | 0.617 | 0.955 | 0.586 | 0.669 | 0.833 | 0.431 | 0.407 | 0.545 |
|  |  | Bai-Urban *vs.* Tibetan-Urban | 0.817 | 0.786 | 0.917 | 0.947 | 0.872 | 0.309 | 0.806 | 0.926 |
|  |  | Bai-Urban *vs.* Uyghur-Urban | 0.872 | 0.528 | 0.933 | 0.891 | 0.532 | 0.300 | 0.382 | 0.779 |
|  |  | Bai-Urban *vs.* Zhuang-Urban | 0.832 | 0.900 | 0.715 | 0.736 | 0.859 | 0.524 | 0.752 | 0.697 |
|  |  | Han-Urban *vs.* Kazakh-Urban | 0.215 | 0.984 | 0.349 | 0.529 | 0.984 | 0.046 | 0.004 | 0.588 |
|  |  | Han-Urban *vs.* Mongol-Urban | 0.691 | 0.947 | 0.818 | 0.734 | 0.895 | 0.045 | 0.873 | 1.000 |
|  |  | Han-Urban *vs.* Tibetan-Urban | 0.304 | 0.734 | 0.384 | 0.421 | 0.810 | 0.060 | 0.383 | 0.651 |
|  |  | Han-Urban *vs.* Uyghur-Urban | 0.565 | 0.574 | 0.550 | 0.530 | 0.590 | 0.045 | 0.101 | 0.738 |
|  |  | Han-Urban *vs.* Zhuang-Urban | 0.551 | 0.868 | 0.712 | 0.696 | 0.924 | 0.052 | 0.635 | 0.838 |
|  |  | Kazakh-Urban *vs.* Mongol-Urban | 0.366 | 0.933 | 0.524 | 0.695 | 0.886 | 0.235 | 0.008 | 0.569 |
|  |  | Kazakh-Urban *vs.* Tibetan-Urban | 0.789 | 0.817 | 0.924 | 0.993 | 0.862 | 0.215 | 0.057 | 0.926 |
|  |  | Kazakh-Urban *vs.* Uyghur-Urban | 0.526 | 0.633 | 0.796 | 0.971 | 0.607 | 0.171 | 0.100 | 0.822 |
|  |  | Kazakh-Urban *vs.* Zhuang-Urban | 0.486 | 0.891 | 0.602 | 0.746 | 0.938 | 0.265 | 0.062 | 0.744 |
|  |  | Mongol-Urban *vs.* Tibetan-Urban | 0.496 | 0.791 | 0.555 | 0.615 | 0.741 | 0.788 | 0.594 | 0.654 |
|  |  | Mongol-Urban *vs.* Uyghur-Urban | 0.777 | 0.490 | 0.642 | 0.569 | 0.631 | 0.862 | 0.177 | 0.734 |
|  |  | Mongol-Urban *vs.* Zhuang-Urban | 0.801 | 0.939 | 0.897 | 0.979 | 0.969 | 0.930 | 0.850 | 0.824 |
|  |  | Tibetan-Urban *vs.* Uyghur-Urban | 0.723 | 0.425 | 0.858 | 0.978 | 0.482 | 0.844 | 0.430 | 0.877 |
|  |  | Tibetan-Urban *vs.* Zhuang-Urban | 0.654 | 0.881 | 0.623 | 0.650 | 0.759 | 0.722 | 0.912 | 0.772 |
|  |  | Uyghur-Urban *vs.* Zhuang-Urban | 0.952 | 0.434 | 0.753 | 0.639 | 0.605 | 0.817 | 0.410 | 0.904 |
|  | (%) With significant differences | | 0 | 0 | 0 | 0 | 0 | 19.0% (4/21) | 9.5% (2/21) | 0 |
|  | Genus | Bai-Urban *vs.* Han-Urban | 0.425 | 0.500 | 0.819 | 0.825 | 0.831 | 0.434 | 0.356 | 0.976 |
|  |  | Bai-Urban *vs.* Kazakh-Urban | 0.695 | 0.705 | 0.568 | 0.620 | 0.887 | 0.627 | 0.127 | 0.586 |
|  |  | Bai-Urban *vs.* Mongol-Urban | 0.668 | 0.725 | 1.000 | 0.944 | 0.862 | 0.612 | 0.643 | 0.997 |
|  |  | Bai-Urban *vs.* Tibetan-Urban | 0.791 | 0.636 | 0.777 | 0.764 | 0.729 | 0.868 | 0.917 | 0.929 |
|  |  | Bai-Urban *vs.* Uyghur-Urban | 0.843 | 0.418 | 0.820 | 0.638 | 0.490 | 0.322 | 0.196 | 1.000 |
|  |  | Bai-Urban *vs.* Zhuang-Urban | 0.751 | 0.546 | 0.916 | 0.916 | 0.781 | 0.251 | 0.324 | 0.784 |
|  |  | Han-Urban *vs.* Kazakh-Urban | 0.266 | 0.841 | 0.325 | 0.373 | 0.938 | 0.624 | 0.100 | 0.494 |
|  |  | Han-Urban *vs.* Mongol-Urban | 0.644 | 0.730 | 0.809 | 0.569 | 0.960 | 0.613 | 0.664 | 0.945 |
|  |  | Han-Urban *vs.* Tibetan-Urban | 0.258 | 0.210 | 0.483 | 0.413 | 0.496 | 0.445 | 0.367 | 0.878 |
|  |  | Han-Urban *vs.* Uyghur-Urban | 0.564 | 0.812 | 0.584 | 0.430 | 0.611 | 0.871 | 0.276 | 0.974 |
|  |  | Han-Urban *vs.* Zhuang-Urban | 0.497 | 0.864 | 0.665 | 0.569 | 0.994 | 0.861 | 0.428 | 0.747 |
|  |  | Kazakh-Urban *vs.* Mongol-Urban | 0.382 | 0.928 | 0.436 | 0.529 | 0.979 | 0.866 | 0.099 | 0.517 |
|  |  | Kazakh-Urban *vs.* Tibetan-Urban | 0.940 | 0.467 | 0.852 | 0.891 | 0.649 | 0.491 | 0.174 | 0.718 |
|  |  | Kazakh-Urban *vs.* Uyghur-Urban | 0.510 | 0.691 | 0.663 | 0.911 | 0.571 | 0.537 | 0.355 | 0.529 |
|  |  | Kazakh-Urban *vs.* Zhuang-Urban | 0.428 | 0.972 | 0.503 | 0.530 | 0.949 | 0.304 | 0.299 | 0.654 |
|  |  | Mongol-Urban *vs.* Tibetan-Urban | 0.503 | 0.415 | 0.682 | 0.667 | 0.598 | 0.522 | 0.593 | 0.925 |
|  |  | Mongol-Urban *vs.* Uyghur-Urban | 0.839 | 0.620 | 0.749 | 0.583 | 0.560 | 0.814 | 0.254 | 1.000 |
|  |  | Mongol-Urban *vs.* Zhuang-Urban | 0.852 | 0.880 | 0.849 | 0.974 | 0.946 | 0.387 | 0.423 | 0.770 |
|  |  | Tibetan-Urban *vs.* Uyghur-Urban | 0.664 | 0.316 | 0.883 | 0.963 | 0.419 | 0.266 | 0.311 | 0.925 |
|  |  | Tibetan-Urban *vs.* Zhuang-Urban | 0.518 | 0.332 | 0.743 | 0.664 | 0.540 | 0.195 | 0.347 | 0.895 |
|  |  | Uyghur-Urban *vs.* Zhuang-Urban | 0.928 | 0.659 | 0.858 | 0.617 | 0.539 | 0.343 | 0.854 | 0.774 |
|  | (%) With significant differences | | 0 | 0 | 0 | 0 | 0 | 0 | 0 | 0 |
|  | Species | Bai-Urban *vs.* Han-Urban | 0.533 | 0.502 | 0.706 | 0.630 | 0.711 | 0.166 | 0.489 | 0.859 |
|  |  | Bai-Urban *vs.* Kazakh-Urban | 0.707 | 0.608 | 0.792 | 0.958 | 0.746 | 0.713 | 0.169 | 0.730 |
|  |  | Bai-Urban *vs.* Mongol-Urban | 0.735 | 0.513 | 0.869 | 0.711 | 0.778 | 0.426 | 0.313 | 0.907 |
|  |  | Bai-Urban *vs.* Tibetan-Urban | 0.946 | 0.602 | 0.813 | 0.734 | 0.654 | 0.764 | 0.775 | 0.870 |
|  |  | Bai-Urban *vs.* Uyghur-Urban | 0.950 | 0.398 | 0.884 | 0.840 | 0.571 | 0.717 | 0.218 | 0.945 |
|  |  | Bai-Urban *vs.* Zhuang-Urban | 0.969 | 0.472 | 0.909 | 0.855 | 0.701 | 0.208 | 0.209 | 0.726 |
|  |  | Han-Urban *vs.* Kazakh-Urban | 0.304 | 0.897 | 0.505 | 0.639 | 0.933 | 0.165 | 0.207 | 0.611 |
|  |  | Han-Urban *vs.* Mongol-Urban | 0.672 | 0.838 | 0.737 | 0.714 | 0.868 | 0.256 | 0.941 | 0.752 |
|  |  | Han-Urban *vs.* Tibetan-Urban | 0.525 | 0.729 | 0.909 | 0.983 | 0.856 | 0.160 | 0.770 | 0.962 |
|  |  | Han-Urban *vs.* Uyghur-Urban | 0.574 | 0.926 | 0.609 | 0.554 | 0.902 | 0.132 | 0.559 | 0.824 |
|  |  | Han-Urban *vs.* Zhuang-Urban | 0.489 | 0.913 | 0.538 | 0.524 | 0.909 | 0.666 | 0.235 | 0.610 |
|  |  | Kazakh-Urban *vs.* Mongol-Urban | 0.453 | 0.947 | 0.627 | 0.732 | 0.946 | 0.501 | 0.181 | 0.749 |
|  |  | Kazakh-Urban *vs.* Tibetan-Urban | 0.655 | 0.867 | 0.624 | 0.698 | 0.936 | 0.606 | 0.345 | 0.628 |
|  |  | Kazakh-Urban *vs.* Uyghur-Urban | 0.674 | 0.837 | 0.888 | 0.904 | 0.831 | 0.479 | 0.342 | 0.775 |
|  |  | Kazakh-Urban *vs.* Zhuang-Urban | 0.630 | 0.988 | 0.832 | 0.839 | 0.974 | 0.177 | 0.798 | 0.959 |
|  |  | Mongol-Urban *vs.* Tibetan-Urban | 0.747 | 0.840 | 0.875 | 0.811 | 1.000 | 0.382 | 0.651 | 0.760 |
|  |  | Mongol-Urban *vs.* Uyghur-Urban | 0.787 | 0.732 | 0.733 | 0.581 | 0.724 | 0.276 | 0.464 | 0.982 |
|  |  | Mongol-Urban *vs.* Zhuang-Urban | 0.685 | 0.917 | 0.726 | 0.796 | 0.955 | 0.316 | 0.261 | 0.777 |
|  |  | Tibetan-Urban *vs.* Uyghur-Urban | 0.994 | 0.601 | 0.657 | 0.559 | 0.700 | 0.887 | 0.430 | 0.800 |
|  |  | Tibetan-Urban *vs.* Zhuang-Urban | 0.973 | 0.758 | 0.639 | 0.686 | 0.932 | 0.193 | 0.295 | 0.571 |
|  |  | Uyghur-Urban *vs.* Zhuang-Urban | 0.968 | 0.805 | 0.951 | 0.708 | 0.755 | 0.132 | 0.538 | 0.763 |
|  | (%) With significant differences | | 0 | 0 | 0 | 0 | 0 | 0 | 0 | 0 |
